# Metabolic and Biochemical Responses of Heirloom and Hybrid Tomato (*Solanum lycopersicum*) Under Flooding, Specialist, and Generalist Insect Herbivory, and their Stress Combination

**DOI:** 10.1007/s10886-026-01703-9

**Published:** 2026-04-02

**Authors:** Michael Somerville, Emma Cartelli, Minxing Zhu, Esther N. Ngumbi

**Affiliations:** Department of Entomology, University of Illinois Urbana-Champaign, Urbana, IL 61801 USA

**Keywords:** Metabolites, Tomato, Volatile organic compounds, Flooding, Stress combination, Insect herbivory

## Abstract

This study investigated how heirloom (Cherokee Purple) and hybrid (New Girl) tomato (*Solanum lycopersicum* L.) plant varieties respond metabolically and biochemically to flooding and specialist (*Manduca sexta*) and generalist (*Spodoptera exigua*) insect herbivory both individually and in combination. Using LC-MS/MS based untargeted metabolite profiling of foliar tissue and solid phase microextraction and GC-MS, we characterized and quantified metabolome and aboveground headspace volatile organic compounds (VOCs) changes in response to individual and combined stressors. We additionally measured aboveground and belowground biomass in each treatment. We hypothesized that (1) flooding would dominate metabolomic reprogramming and VOC emissions; (2) specialist and generalist herbivores would elicit distinct metabolic and VOC responses; (3) single and combined stresses would produce distinct metabolite and VOC profiles; and (4) VOC emissions would differ between heirloom and hybrid tomato varieties. Flooding was the primary driver of metabolic reprogramming, VOC emission, and reduced root biomass in tomato, with accumulated metabolites linked to oxidative stress mitigation. Differences between generalist and specialist herbivory were most evident in secondary sulfur metabolites. In addition, in the heirloom tomato variety, the generalist altered many more metabolites than the specialist, a trend that was reversed in the hybrid variety. Under combined stress, flooding-induced metabolic changes were largely preserved or additive, including the accumulation of indole metabolites associated with multifunctional roles including priming plant defenses against biotic stressors, whereas herbivore-driven metabolic changes were attenuated. Monoterpenes increased under flooding and combined stress, especially in the heirloom variety. Finally, we documented varietal differences in metabolic responses reflected in increased accumulation of several metabolites in the heirloom tomato variety compared with the hybrid. Together, our results reveal that flooding, herbivory by specialist and generalist caterpillar species and their interactions can dynamically change metabolites, volatile organic compounds, and plant growth patterns and suggest that breeding history shapes plants metabolic response and resillience under multi-stress combinations.

**Supplementary Information:**

The online version contains supplementary material available at 10.1007/s10886-026-01703-9.

## Introduction

Tomato (*Solanum lycopersicum* L.) is an economically important vegetable crop, with global tomato production exceeding 192 million tons in 2023 (FAO 2023). Tomato plants are subjected to intensive selective breeding to improve fruit quality, size, flavor, and yield, which may come at the cost of reduced resistance to abiotic and biotic stressors (Tieman et al. 2017; Paudel et al. 2019; Safavi-Rizi et al. 2020b; Yin et al. 2023). Tomato is also an excellent model crop system with many accessions that represent broad geographic, phenotypic, and genetic diversity, including wild relatives, mutants, and heirloom and hybrid varieties shaped by domestication and modern crop breeding, offering valuable natural allelic variants useful for genetic and metabolic analyses and subsequent breeding applications (Mata-Nicolas et al. 2020). Tomato production is threatened by abiotic and biotic stressors, including flooding and insect herbivory, which can occur individually, in quick succession, or simultaneously (Suzuki et al. 2014; Kissoudis et al. 2015, 2016; Pandey et al. 2017; Zandalinas et al. 2022; Ngumbi et al. 2022; Mittler et al. 2025). To resist these stressors, tomato plants use multiple strategies, including reprogramming their physiology, metabolism, and biochemistry, along with measurable changes in metabolite accumulation patterns and volatile organic compounds (VOCs) emissions (Steinbrenner et al. 2011; Bailey-Serres et al. 2012; Schuman and Baldwin 2015; Zhou et al. 2015; De Ollas et al. 2021; Ngumbi et al. 2022; Luo et al. 2023; Zandalinas et al. 2024; Bulut et al. 2025). Once quantified using metabolite and chemistry profiling techniques, insights, including the identification of metabolites that consistently increase, decrease, or remain stable when crops are exposed to simultaneously occurring stressors can guide efforts to enhance the resilience of tomato plants amid changing climate conditions (Fabregas and Fernie 2019; De Ollas et al. 2021; Kissoudis et al. 2015; Manghwar et al. 2024; Mittler et al. 2025).

Flooding depletes oxygen, an essential substrate for many biochemical reactions. As a result, it significantly affects plant physiological processes, promotes the buildup of harmful reactive oxygen species (ROS), and triggers extensive reprogramming of the metabolome and metabolic pathways, including those involved in sugar, carbon, nitrogen, lipid, and amino acid metabolism, as well as oxidative stress mitigation and defense mechanisms (e.g., phenylpropanoid, isoprenoid, and flavonoid pathways) (Bailey-Serres and Voesenek 2008; Bailey-Serres et al. 2012; Coutinho et al. 2018; Lothier et al. 2020; De Ollas et al. 2021). For example, short-term (6 h) and long-term (48 h) flooding stress exposure in tomatoes (Money Maker) led to reprogramming of carbon and amino acid metabolism (Safavi-Rizi et al. 2020b). In another study involving four tomato genotypes, two commercial cultivars, Lukullus and Ailsa Craig, and their two isogenic ABA-deficient lines, *notabilis* and *flacca*, De Ollas et al. (2021) showed that flooding caused a genotype-specific reorganization of the metabolome. Furthermore, they showed that flooding reduced the accumulation of metabolites in groups such as fatty acids, phenylpropanoids, oxylipins, and terpenoids. Similarly, Umićević et al. (2024) observed significant genotypic variation in the accumulation patterns of several metabolites after flooding, including 5-o-caffeoylquinic acid (a phenolic acid) and rutin (a steroidal glycoalkaloid). Flooding-driven metabolome reprogramming is primarily mediated by ethylene, often interacting with other signaling molecules and hormones such as abscisic acid, auxin, and gibberellins (Bailey-Serres and Voesenek 2020; Vidoz et al. 2010; Sasidharan and Voesenek 2015; Nguyen et al. 2016a, b; De Ollas et al. 2021; Safavi-Rizi et al. 2020a). Moreover, multiple studies across various crop species, including tomato, have demonstrated that flooding increases the emission of volatile organic compounds (Block et al. 2019; Ngumbi et al. 2022; Mleziva and Ngumbi 2024; Ngumbi et al. 2025). Collectively, previous studies have revealed that the metabolic responses to flooding in tomatoes are variable and shaped by the genotype/variety used. In addition, these studies suggest that flooding generally decreases the accumulation of defense-related metabolites. This documented variability highlights the need for additional studies using different tomato varieties to identify broad patterns in metabolite accumulation patterns. Once identified, these insights can guide efforts to develop flooding-resilient crops with metabolites as breeding targets.

Herbivory by both specialist and generalist leaf-chewing insects, including *Manduca sexta* and *Spodoptera exigua*, also triggers significant reprogramming of plants primary and secondary metabolism, and these changes are detectable soon after damage at the whole-plant level (Kessler and Baldwin 2002; Schwachtje and Baldwin 2008; Diezel et al. 2009; Gomez et al. 2012; Steinbrenner et al. 2011; Zhou et al. 2015). In tomato plants, the metabolic pathways reprogrammed following insect herbivory include those associated with carbon and nitrogen mobilization and the biosynthesis of diverse secondary metabolites such as rutin, quercetin, solasodine, α-tomatine, acyl sugars, and terpenoids (Steinbrenner et al. 2011; Gomez et al., 2012; Rivero et al. 2021). These compounds are known to deter various herbivores, including whiteflies, leaf-mining insects, and chewing herbivores (Barbour and Kennedy 1991; Kowalski et al. 1999; Steinbrenner et al. 2011; Errard et al. 2016; Su et al. 2025; Zhang et al. 2025). These metabolic adjustments enable plants to tolerate herbivory while minimizing impacts on fitness traits (Kerchev et al. 2012). For instance, Steinbrenner et al. (2011) demonstrated changes in tomato metabolism in response to feeding by a generalist herbivore (*Helicoverpa zea*) and a specialist (*Manduca sexta*). The generalist herbivore altered more metabolites than the specialist and strongly affected the concentrations of defense-related metabolites, including precursor amino acids and simple phenolics, indicating an enhanced response of tomatoes to *H. zea*. By contrast, the specialist herbivore primarily influenced metabolites related to nitrogen and carbon transport. In another study, Gomez et al. (2012) showed that herbivory by the specialist tomato herbivore *M. sexta* increased concentrations of primary metabolites. Unlike flooding stress, the metabolic reprogramming induced by insect herbivory, particularly from chewing insects, is driven by the jasmonic acid signaling pathway, which often acts synergistically and in crosstalk with other hormones, including salicylic acid, ethylene, abscisic acid, and cytokinin (Schwachtje and Baldwin 2008; Nguyen et al. 2016a, b a). Additionally, these hormone interactions, which strongly influence herbivore-induced metabolic reprogramming and resistance outcomes, vary depending on the plant species, herbivore identity and specialization, and salivary elicitors (Nguyen et al. 2016a, b b; Diezel et al. 2009). Overall, previous studies have argued that generalist species trigger broader metabolic reprogramming, whereas specialist insects tend to suppress defense signaling strategies, subsequently reducing crops’ metabolic responses (Diezel et al. 2009; Voelckel et al. 2004; Steinbrenner et al. 2011; Gomez et al. 2012). These distinct herbivore-specific metabolic responses set the stage for complex, potentially non-additive reactions when insect herbivory occurs simultaneously with flooding, an area that remains to be fully understood.

Additionally, in response to herbivore damage, tomato plants emit an induced blend of volatile organic compounds that serve various ecological roles, including attracting parasitoids and predators, mediating communication within and between plants, directly repelling ovipositing herbivores, and inhibiting the growth and performance of their offspring (Rodriguez-Saona et al. 2005; Silva et al. 2017; Catola et al. 2018; Paudel et al. 2019; Ayelo et al. 2021). The herbivore-induced VOC blend can vary significantly depending on factors such as crop species, genotype, domestication and breeding history, development stage, the attacking herbivore species, its feeding guild, diet breadth, and density of attacking herbivores (Rowen and Kaplan 2016; Paudel et al. 2019). Because crops’ overall response to insect herbivory can be influenced by multiple factors, including their genotype and breeding history, more studies are needed using various crop varieties currently grown in modern production systems.

In nature and during a growing season, crops often face a combination of two stressors (Mittler 2006; Suzuki et al. 2014; Nguyen et al. 2016a, b; Havko et al. 2020; Ngumbi et al. 2022; Pawar et al. 2025). Despite the availability of many tomato varieties with diverse breeding histories, including wild relatives, heirlooms, and modern hybrids, our understanding of how their biochemical and metabolic responses to stress combinations differ remains limited. Although still limited, results from studies of stress combinations show that when biotic and abiotic stressors occur simultaneously, their effects can be additive, synergistic, antagonistic, or neutral. In some cases, the influence of one stress can become dominant, and in others, crops may prioritize responding to one stress over the other (Zhou et al. 2017; Sewelam et al. 2020). These complex, variable, and unpredictable outcomes highlight the need for more experiments involving multiple stress combinations, such as flooding and insect herbivory. These studies should use tools such as metabolomics to uncover general and unique responses to concurrent stressors. The results and insights from these experiments can guide efforts to breed crop varieties that tolerate multiple stresses.

A crop’s metabolic and chemical response to stress combinations can be further shaped, in part, by breeding. Over the past 100 years, human-directed crop breeding has produced dramatic and remarkable genotypic and phenotypic changes in many crops, along with shifts in physiological, metabolic, and defensive traits (Blanca et al. 2015; Paudel et al. 2019). Previous studies have shown that, in tomato, breeding for larger fruit size, firmness, and quality, early ripening, disease resistance, and higher yields, for example, qualities important for long-term storage, shipping, and appearance, comes at the expense of altering the response to abiotic and biotic stressors and potential combinations (Tieman et al. 2017; Paudel et al. 2019; Yin et al. 2023).

Although tomato has been widely used as a model crop to study the effects of individual biotic and abiotic stressors on the plant’s metabolome and induced responses, including volatile emissions, only a few studies have examined how these responses change when stressors occur simultaneously (Nguyen et al. 2016a, b a; Catola et al. 2018; Havko et al. 2020; Ngumbi et al. 2022). These studies have confirmed that outcomes from combinations of stressors are unique and cannot be predicted from responses to individual stressors. For example, Havko et al. (2020) investigated the combined stress of elevated temperature and herbivory by *M. sexta* in cultivated tomatoes (cv. Castlemart). They showed that the combination resulted in an antagonistic response, with insect herbivory suppressing leaf cooling responses to elevated temperature. In another study on *Solanum dulcamara*, Nguyen et al. (2016a, b) demonstrated that flooding, combined with herbivory, suppressed herbivore-induced processes and responses. Ngumbi et al. (2022) studied two heirloom tomato varieties (Cherokee Purple and Striped German) and found that the combined stress of flooding and herbivory elicited significantly different transcriptomic responses than plants exposed to flooding or herbivory alone. Additionally, studies examining how combinations of stress affect VOC emissions have yielded mixed results, with some reporting additive effects and others showing non-additive or neutral effects (Catola et al. 2018; Ngumbi et al. 2022). Given these findings, which indicate that stress combinations produce distinct outcomes, along with the fundamentally different physiological challenges posed by flooding and insect herbivory, and considering the influence of crop variety, breeding history, and herbivore specialization, there remains a clear need for additional research. Specifically, studies should explicitly and comprehensively investigate how biotic and abiotic stressors interact to reprogram plant metabolism and chemistry.

Metabolomics, including both targeted and non-targeted profiling, has proven to be a powerful tool that plays an invaluable role in revealing system-wide and systematic changes in plant metabolism in response to biotic and abiotic stressors and their potential combinations (Allwood et al. 2021; De Ollas et al. 2021; Rivero et al. 2022; Kumaraswamy et al. 2025). Integrating metabolomics with chemical profiling can lead to a more comprehensive understanding of how plants respond dynamically and adaptively to individual and combined stressors.

Therefore, in this study, we performed untargeted metabolite profiling to measure metabolomic changes in two tomato varieties (heirloom and hybrid) in response to flooding, herbivory by a specialist (*Manduca sexta*), a generalist (*Spodoptera exigua*), and their combined stress. We also examined changes in volatile chemistry (VOCs) and assessed the effects of treatments on their below-and-above ground growth parameters. Given the fundamental physiological challenges posed by flooding (depriving plants of oxygen, which is essential for all metabolic processes) and insect herbivory (affecting photosynthesis and subsequently carbon allocation patterns), along with the hormone cross-talk that drives and supports metabolic and induced responses, we hypothesized that (1) flooding would be the dominant stressor, increasing VOC emissions and altering metabolite accumulation patterns, increasing metabolites with stress-protective functions and decreasing defense-related metabolites; (2) the generalist herbivore would induce stronger metabolic reprogramming and higher accumulation of defense metabolites compared to the specialist, which has evolved the ability to suppress or tolerate jasmonic acid-driven defenses; (3) the combination of flooding and insect herbivory would produce metabolite accumulation and VOC emission patterns different from those documented under single stress, reflecting additive, synergistic, subtractive (antagonistic), or prioritization of the dominant stressor effect; (4) metabolic reprogramming and VOC emission patterns would be more pronounced in the heirloom tomato variety compared to the hybrid, reflected in increased abundance of putatively identified metabolites and total VOC emissions, revealing how breeding history influences crop resilience.

## Methods and Materials

### Plant Material

We used two organic tomato (*Solanum lycopersicum* L.) varieties: an heirloom, Cherokee Purple (CP) and a hybrid, New Girl (NG). Cherokee Purple was selected as it is among several highly valued and economically important heirloom crops utilized by farmers (Watson 1996; Goldman et al. 2008). The New Girl variety is a popular F1 hybrid of the Early Girl parent line (Johnny’s Seeds, Winslow, Maine, USA) and has extended shelf life and deep red coloration in fruit due to selective breeding (Kitagawa et al. 2005). Seeds were obtained from Johnny’s seeds (Johnny’s Seeds, Winslow, Maine, USA). Seeds were germinated in seed trays (1020 planting trays, in 72-cell trays; 25 × 51 cm) containing a soil propagation mix (Berger BM2 Seed Germination & Propagation Mix; Berger, Saint-Modeste, Quebec, CA). They were maintained at the greenhouse facilities at the University of Illinois at Urbana Champaign plant care facility (PCF) at 25 °C ± 5 °C, 50 ± 5% relative humidity and 14 L:10D photoperiod. Two weeks old tomato seedlings were transplanted into individual plastic pots (12 cm x 14 cm dia.) (Hummert International, Earth City, Missouri, USA) containing 80:20 field soil: sand mixture. Thereafter, plants were grown for three weeks at 25 ℃ ± 5 ℃, 50 ± 5% relative humidity and 14 L:10D photoperiod.

### Insect Herbivores

Two insect herbivores were used in this study: a specialist, *Manduca sexta*, and a generalist, *Spodoptera exigua*. The specialist caterpillar was obtained from an internal colony at UIUC maintained by James Nardi (Department of Entomology, UIUC). *M. sexta* larvae were raised at UIUC on a laboratory-prepared diet (Yamamoto 1969). The generalist caterpillars were purchased from Benzon Research (Carlisle, Pennsylvania, USA). Third instar *M. sexta* and *S. exigua* caterpillars were used for the study. Caterpillars were maintained in a constant temperature incubator at 26 °C ± 2 °C, and 16 L:8D photoperiod until used for the experiments.

### General Methods

Five weeks old plants were randomly assigned into six treatment groups as follows: (1) no flooding (control), (2) no flooding + herbivory by *S. exigua*, (3) no flooding + herbivory by *M. sexta*, (4) flooding, (5) flooding + herbivory by *S. exigua*, and (6) flooding + herbivory by *M. sexta*. Each treatment group had five biological replicates of each variety reserved per treatment group (*n* = 5) for untargeted metabolite sampling. To impose flooding, pots containing tomato plants were placed in a secondary larger plastic bucket. For all treatments involving flooding, buckets were filled with water up to 5 cm above the soil surface. This water level was maintained for the duration of the flooding period, ensuring that the soil remained completely waterlogged, so the roots were under constant flooding conditions. For floooding treatments, flooding was implemented for a duration of five days which offers an adequate time frame to document dynamic changes in tomato plants physiology, metabolism and biochemistry following stress exposure (Renziehausen et al. 2024). Moreover, previous studies and preliminary studies on flooding have used this time frame (Ide et al. 2022; Ngumbi et al. 2022). For treatments involving herbivory, both insect species were first starved for 24 h, followed by 16 h of uninterrupted feeding. Feeding on plants was initiated at the same time as with stress combination treatments. For combinatorial treatments, herbivory was initiated promptly after the three days (approximately 72 h) of flooding stress and allowed to feed for the same duration as herbivory treatment while the plants were still flooding. One insect was placed onto the third and fourth most apical lateral shoot of each plant. These individual leaves, along with the respective insect, were contained inside of sterilized nylon mesh bags (11.5 cm x 9 cm). Observationally, *M. sexta* insects were more voracious than *S. exigua* insects, and both herbivores produced chewing-type damage in the leaf tissue of their hosts. Subsequently, 60 mg of fresh leaf tissue was collected from the leaflet that had been subjected to active feeding for untargeted metabolite profiling. Leaf tissues were collected with a 7 mm cork-borer, and they were immediately placed in 2 ml tubes, snap-frozen in liquid nitrogen and stored in -80 ℃ until the day of analysis.

### Untargeted Metabolite Profiling and Analysis

First, 60 mg samples of tomato foliar tissue were homogenized in 1 ml of Acetonitrille: IPA: Water (3:3:1 v/v) using Fisherbrand™ Bead Mill 4 Mini Homogenizer (5 min at max speed) and centrifuged for 15 min at 20,000 RCF. Next, 100uL of supernatant were transferred into glass vials, spiked with 5uL of internal standard (4-Chloro-DL-phenylalanine, 25ug/mL). Additionally, 50uL of supernatant from each sample were pulled together, 100ul were spiked with internal standard and used as a QC group. Leaf tissue was extracted and processed by the Metabolomics Unit of the Roy J. Carver Biotechnology Center (University of Illinois Urbana-Champaign, Urbana, IL, USA), following the protocol of Elolimy et al. (2019). Samples were analyzed with the Q-Exactive MS system (Thermo. Bremen, Germany). Xcalibur 4.1.31.9 software was used for data acquisition. The Dionex Ultimate 3000 series HPLC system (Thermo, Germering, Germany) equipped with a degasser, an autosampler and a binary pump. The LC separation performed on a Kinetex C18 100 A column (100 × 4.6 mm, 2.6u) (Phenomenex, USA) with mobile phase A (H2O with 0.1% formic acid) and mobile phase B (acetonitrile with 0.1% formic acid). The flow rate was 0.25 mL/min. The linear gradient was: 0–3 min, 100% A; 20–30 min, 100% A at 31–36 min. The autosampler was set to 5 °C and injection volume was 20 µL. Mass spectra were acquired under both positive and negative electrospray ionization modes. Data dependent acquisition (DDA) was performed. All the LCMS raw data files were performed using MS-DIAL ver.4.80 software for data collection, peak detection, alignment, adduct, and identification (Tsugawa et al. 2015). All acquired high resolution LC-MS/MS spectra were compared with those from mzCloud, mzVault and Chem Spider mass spectral libraries. The detailed parameter setting was as follows: MS1 tolerance, 0.005 Da; MS2 tolerance, 0.01 Da; minimum peak height, 10,000 amplitude; mass slice width, 0.05 Da; smoothing method, linear weighted moving average; smoothing level, 3 scans; minimum peak width, 5 scans. [M-H]-, [M-H2O-H]-, [2 M-H]- and [M + H]+, [2 M + H]+, [M + NH4] +, [M + Na] + were included in adduct ion setting for negative and positive mode, respectively. Positive and negative mode data were combined and replicate identifications removed. Compounds were annotated by m/z and MS/MS spectra against the MassBank of North America (MoNA) and NIST20 libraries. MS-DIAL makes identifications and calculates a total score by taking into account MS/MS similarity, MS1 similarity, and isotope ratio similarity. From the MS-DIAL results file, all detected features/metabolites were removed if (sample max)/(blank average) < 10. Manual inspections of remaining spectra MSI level 2 features (putatively annotated compounds) were conducted (Sumner, 2007). Features that were unlikely to be present were reassigned to ‘unknown,’ as their identification was unlikely to be identified correctly. All sample peak heights (semi-quantitative) were normalized to the metabolite total ion chromatogram (mTIC). MS/MS spectra and retention time of putative biomarkers were confirmed by injection of authentic standards.

### Plant Headspace Volatiles Collection and Analysis

Six replicates from each treatment group (*n* = 6) were reserved to measure plant headspace volatiles collected from the treatment combinations (no flooding (control), no flooding + herbivory by *S. exigua*, no flooding + herbivory by *M. sexta*, flooding, flooding + herbivory by *S. exigua*, and flooding + herbivory by *M. sexta*) using solid phase microextraction (SPME) technique. This technique follows protocols utilized by Ngumbi et al. (2022). Briefly, plants were wrapped with an odor blocking oven bag (Arcadia INTL, El Monte, California, USA). Once wrapped, they stayed sealed for one hour to allow for volatile compound accumulation. After one hour, the SPME fiber (65 μm polydimethyl dimethylsiloxane-divinylbenzene (PDMS/DVB) fused silica, and the stainless-steel fiber (Millipore Sigma^®^, Milwaukee, Wisconsin, USA) were inserted into the bag and remained there for 40 min, following which the SPME fiber was withdrawn and immediately inserted into an Agilent 7890B gas chromatography (GC) system equipped with a fused silica DB-5ms phase (30 m x 0.250 mm x 0.25 μm) column and interfaced to an Agilent 5977 A MSD (Agilent Technologies Inc., Santa Clara, CA, USA). Samples were run through coupled gas chromatography-mass spectrophotometry. The column was programmed from 40 ℃/2 min, 5 ℃/min to 200 ℃, for a total of 40 min. The injector and transfer line temperatures were set at 200 ℃. Peaks were identified using the NIST 98 library (Anastasaki et al. 2015). Peak identification was done using the NIST 98 library and by comparing published GC profiles of tomato headspace volatiles. Only peaks with a match greater than 90% in the NIST library were included and reported. The structures of the identified compounds were further validated using commercially available synthetic standards from Millipore Sigma (St. Louis, Missouri, USA). Peaks with less than 90% match or those not confirmed with synthetic standards were excluded from analysis.

### Plant Growth Determination

Shoots and roots were harvested immediately following the tissue sampling conducted on day 5 after the flooding was initiated. Fresh root and shoot weights were recorded in grams; shoot and root length were measured in centimeters for all treatments in both varieties. Shoots and roots were thereafter placed in brown paper bags, oven dried at 70 ℃ for 3 days and the dry weights were recorded.

### Statistical Analysis

Univariate and multivariate analyses of untargeted metabolomics data were conducted using MetaboAnalyst 6.0 (https://www.metaboanalyst.ca.) (Pang et al. 2021). For all analysis, missing values were replaced by 1/5 of the minimum value of their corresponding variable. For multivariate analysis, Principal Component Analysis (PCA) of log and auto-scaled data were performed to discriminate each two-group comparison. Univariate analysis was performed on log transformed data using Student’s t-tests for each two-group comparison. Adjustment for multiple hypothesis testing was performed according to the Benjamini–Hochberg method at a false discovery rate (FDR) of 5% (Benjamini and Hochberg 1995). Statistical analysis of all plant chemistry and growth data were performed using R studio statistical software (Version 4.2.0) (R Core Team 2022). Figures were generated using the ggplot2 package (Wickham et al. 2016). To visualize the effect of variety, flooding, and herbivory on the emission VOCs, non-metric dimensional scaling (NMDS) analysis was performed using a Bray-Curtis’s dissimilarity matrix using the metaMDS function from the vegan package (version 2.6-2) (Oksanen et al. 2022). To assess the influence of tomato variety, flooding, herbivory by a specialist and generalist caterpillar species on VOCs emissions and plant growth characteristics, three-way analyses of variance (ANOVAs) were used. VOCs that strongly influenced differences between treatments were identified and ranked by the Random Forest algorithm, using the randomForest function (package ‘randomForest’ version 4.7.1.1) (Liaw and Wiener 2002; Ranganathan and Borges, 2010; McCormick et al. 2012). To determine variable selection, models underwent 1000 iterations. The percent increase in mean square error was used to rank the compound importance; greater values indicate higher importance.

## Results

### Overall Heirloom (CP) and Hybrid (NG) Metabolism Responses Under Flooding, Generalist (*S. exigua*) or Specialist (*M. sexta*) Herbivory, and their Interaction

**Fig. 1:**
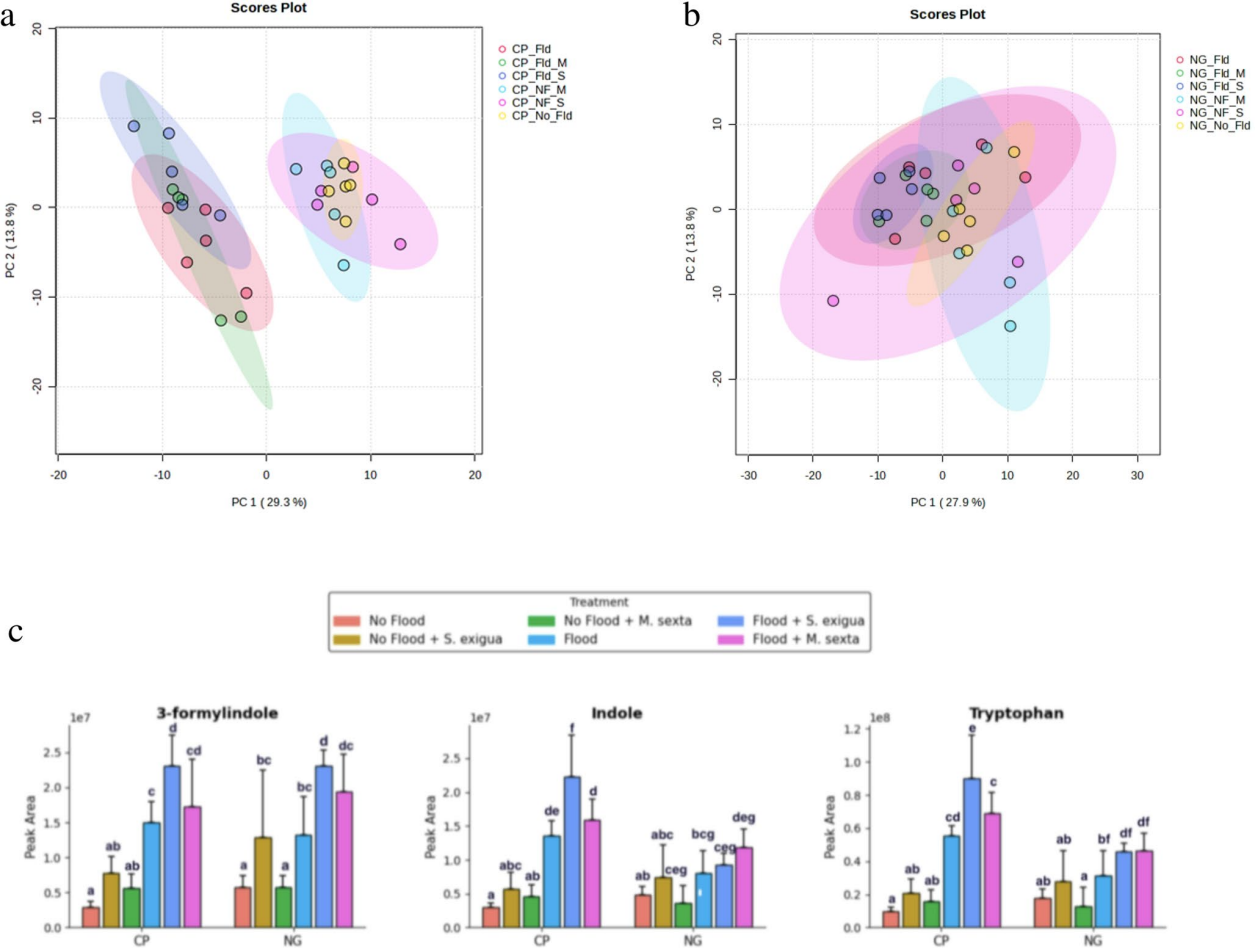

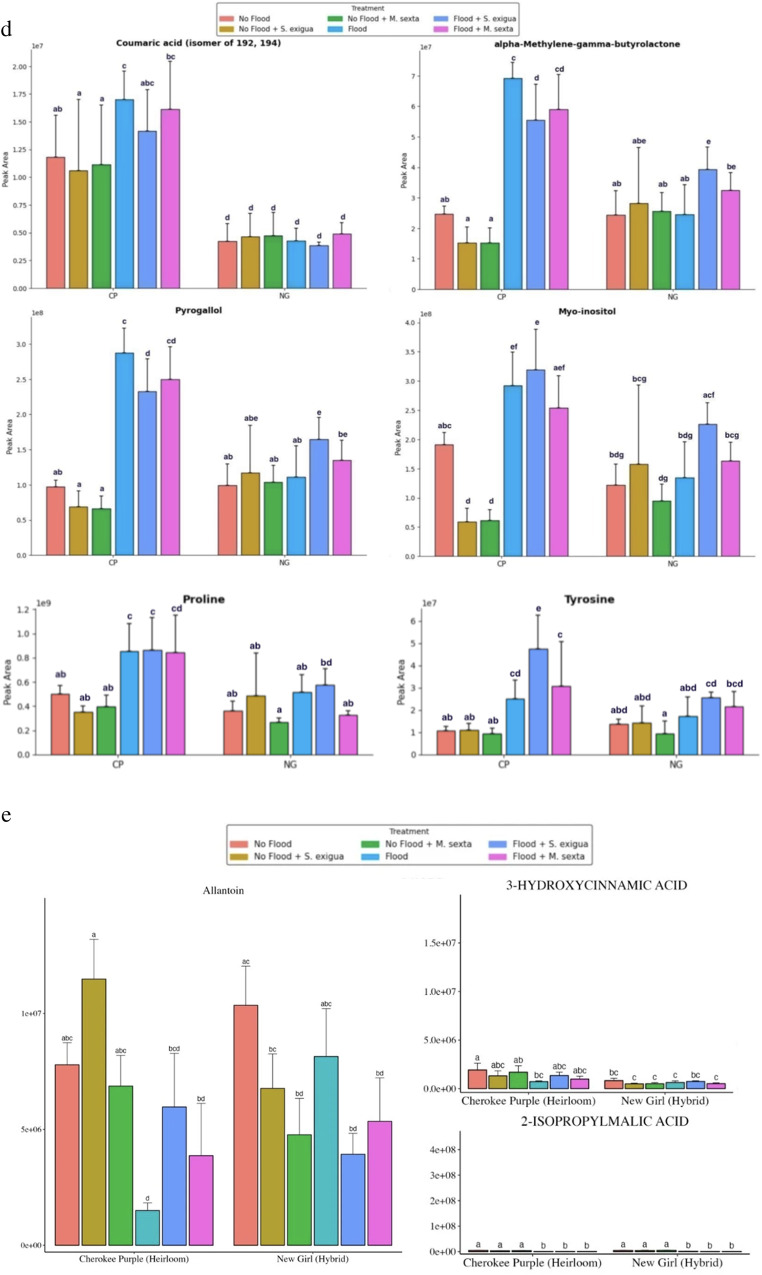

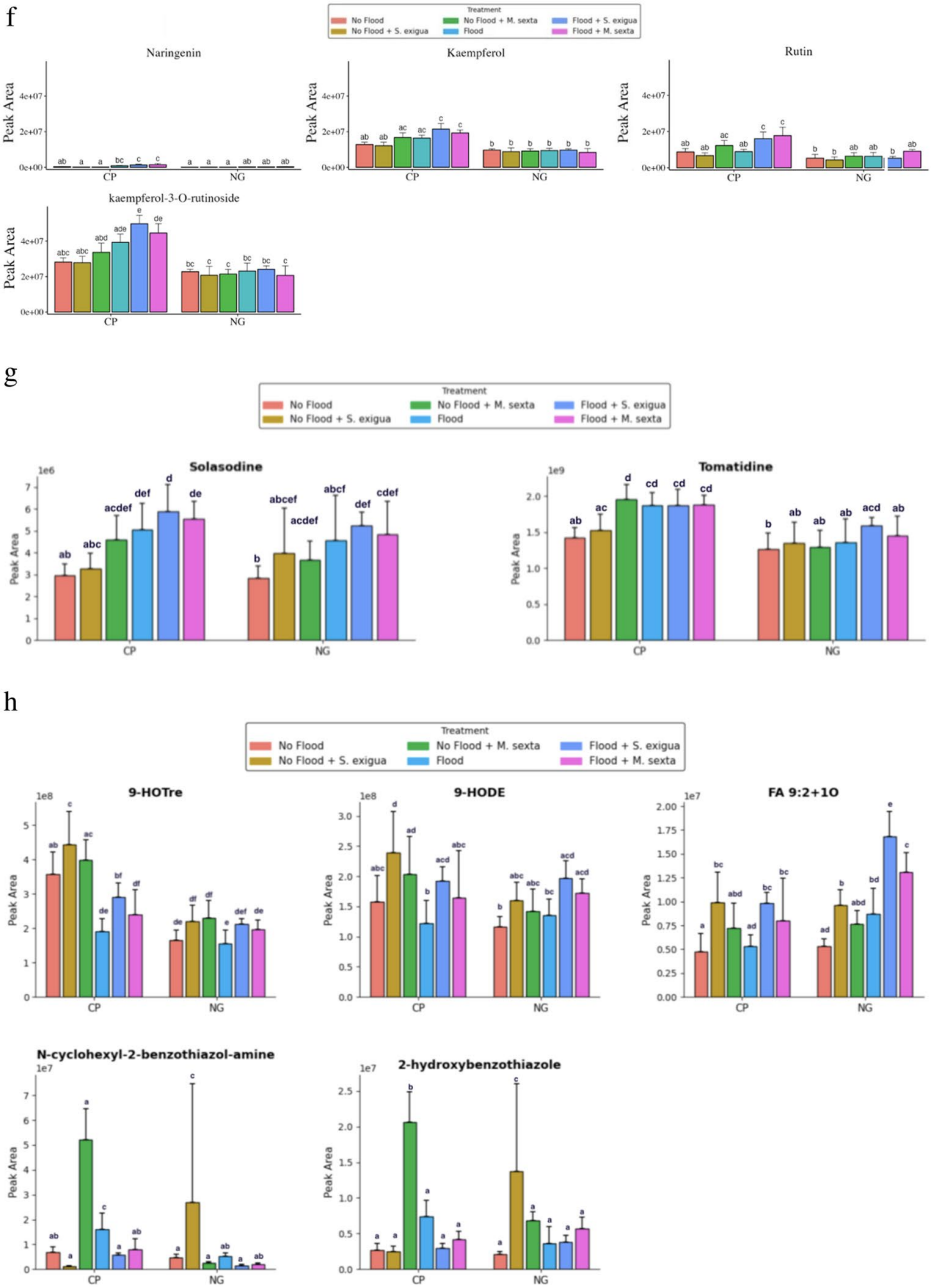
**a.** Principal component analysis (PCA) scores plots indicating the general grouping patterns between the different treatments comprising of heirloom tomato (Cherokee Purple) plants under flooding, herbivory by the specialist (*M. sexta*) and generalist (*S. exigua*) caterpillar species and their combination. The first two principal components PC1 (29.3%) and PC2 (13.8%) explain 43.1% of the total variance. All three clusters of CP_FLD (Cherokee Purple flooding treatments) demonstrate a significant divergence from the other three clusters representing CP_NF (Cherokee Purple no-flooding treatments). **b.** Principal component analysis (PCA) scores plots indicating the general grouping patterns between the different treatments comprising of hybrid (NG) tomato plants under flooding (NG_FLD), herbivory by the specialist (*M. sexta*) (NG_NF_MS) and generalist (*S. exigua*) (NG_NF_SE) caterpillar species and their combination (NG_FLD_MS, NG_FLD_SE). The first two principal components PC1 (27.9%) and PC2 (13.8%) explain 41.7% of the total variance. **c**. Accumulation patterns of metabolites contributing to differences between flooding and non-flooding treatments involving heirloom and hybrid tomato varieties subjected to flooding, herbivory by the specialist (*M. sexta*) and generalist (*S. exigua*) caterpillar species and their combination (*n* = 5) with standard error bars. Letters indicate significantly different treatments from three-way ANOVA using Tukey’s honestly significant difference tests (*P* < 0.05). Indole, tryptophan, and 3-formylindole contributed to significant (*P* < 0.05) differences between flooding and non-flooded treatments. **d**. Accumulation patterns of metabolites contributing to differences between heirloom and hybrid tomato varieties subjected to flooding, herbivory by the specialist (*M. sexta*) and generalist (*S. exigua*) caterpillar species and their combination (*n* = 5) with standard error bars. Letters indicate significantly different treatments from three-way ANOVA using Tukey’s honestly significant difference tests (*P* < 0.05). Coumaric acid (isomer of 192, 194), pyrogallol, α-methylene-γ-butyrolactone, proline, tyrosine, and myo-inositol contributed to significant (*P* < 0.05) differences between hybrid and heirloom tomato treatments. **e**. Accumulation patterns of metabolites contributing to differences between flooding and non-flooding treatments involving heirloom and hybrid tomato varieties subjected to flooding, herbivory by the specialist (*M. sexta*) and generalist (*S. exigua*) caterpillar species and their combination (*n* = 5) with standard error bars. Letters indicate significantly different treatments from three-way ANOVA using Tukey’s honestly significant difference tests (*P* < 0.05). 3-hydroxycinnamic acid, allantoin, and 2-isopropylmallic acid contributed to significant (*P* < 0.05) differences between flooding and non-flooded treatments. **f.** Accumulation patterns of metabolites contributing to significant (*P* < 0.05) differences between single and stress combination treatments heirloom and hybrid tomato varieties subjected to flooding, herbivory by the specialist (*M. sexta*) and generalist (*S. exigua*) caterpillar species and their combination (*n* = 5) with standard error bars. Letters indicate significantly different treatments from three-way ANOVA using Tukey’s honestly significant difference tests (*P* < 0.05). Kaempferol, kaempferol 3-rutinoside, naringenin, and rutin contributed to significant (*P* < 0.05) differences between single and stress combination treatments. **g.** Accumulation patterns of metabolites contributing to significant (*P* < 0.05) differences between single and stress combination treatments heirloom and hybrid tomato varieties subjected to flooding, herbivory by the specialist (*M. sexta*) and generalist (*S. exigua*) caterpillar species and their combination (*n* = 5) with standard error bars. Letters indicate significantly different treatments from three-way ANOVA using Tukey’s honestly significant difference tests (*P* < 0.05). Solasodine and tomatidine contributed to significant (*P* < 0.05) differences between single and stress combination treatments. **h**. Accumulation patterns of metabolites contributing to differences between herbivory by a specialist and generalist caterpillar species in heirloom and hybrid tomato varieties subjected to flooding, herbivory by the specialist (*M. sexta*) and generalist (*S. exigua*) caterpillar species and their combination (*n* = 5) with standard error bars. Letters indicate significantly different treatments from three-way ANOVA using Tukey’s honestly significant difference tests (*P* < 0.05). 9-HOTrE, 9-HODE, and FA 9:2 + 1O produced significant (*P* < 0.05) differences between herbivory by a specialist (*M. sexta*) vs. generalist (*S. exigua*) caterpillar species. 2-hydroxybenzothiazole and N-Cyclohexyl-2-benzothiazole-amine produced significant (*P* < 0.05) divergence in their accumulation patterns in response to herbivory by generalist and specialist caterpillar species in heirloom and hybrid tomato plants

A total of 168 putative metabolites, including primary and secondary metabolites, were identified among the treatments used in this study. The identified metabolites consisted of carboxylic acids and derivatives, flavonoids, organooxygen compounds, fatty acyls, benzothiazoles, steroids and steroid derivatives, keto acids and derivatives, cinnamic acids and derivatives, quinolines and derivatives, quinoline carboxylic acids, phenols, lactones, piperidines, indoles and derivatives, hydroxycinnamic acids, ketones, diazines, pyrimidine nucleosides, benzene and substituted derivatives, prenol lipids, and hydroxy acids and derivatives (Supplemental Table 1). Our results revealed that flooding was by far the major factor shaping reprogramming of heirloom tomato plant non-volatile metabolomes. Principal component analysis revealed that leaf metabolomes of heirloom tomato plants exposed to flooding versus those that were not flooded were compositionally different, evident by separate clustering (Fig. 1a). Treatment differences were more pronounced in the heirloom variety (Fig. 1a), with the first two principal components PC1 (29.3%) and PC2 (13.8%) explaining 43.1% of the total variance. On the contrary, we did not get clear and distinct treatment pattern differences in hybrid tomato metabolome accumulation patterns (Fig. 1b). Treatment differences were largely overlapping in the hybrid variety (Fig. 1b), with the first two principal components PC1 (27.9%) and PC2 (13.8%) explaining 41.7% of the total variance.

### Metabolic Differences between Flooded and Non-flooded Treatments

Flooding stress significantly reshaped the metabolite profiles (*P* < 0.05), resulting in significant elevated accumulation of several metabolites including indole, tryptophan and 3-formyl indole (indoles and derivatives), pyrogallol (phenols), alpha-glucose-1-phosphate (organooxygen compounds/carbohydrates and carbohydrate conjugates), mannose, α-methylene-γ-butyrolactone, 4-hydoxyquinoline, glutamic acid, aconitic acid, naringenin, P-hydroxycoumaric acid, and cuminyl alcohol relative to non-flooded conditions (Fig. 1c) (Fig. 1d) (Supplementary Table 1) (Supplementary Table 2). We additionally documented significant reduction in the accumulation of several metabolites by flooding including allantoin (azoles), 2-isopropyl malic acid, beta-alanine, citrate, allantoic acid, and 3-hydroxycinnamic acid, and caffeic acid hexoside (Fig. 1e) (Supplementary Table 1). Finally, a few defense-related metabolites were significantly reduced by flooding and these included D-(-)-quinic acid, 3-hydroxycinnamic acid, fatty acyls 9-HOTrE, 9-HODE, (10E,15Z)-9,12,13-trihydroxyoctadeca-10,15-dienoic acid, (Z)-5,8,11-trihydroxyoctadec-9-enoic acid and Fa 18:4 + 10 (Fig. 1h) (Supplementary Table 1) (Supplementary Table 2). Other notable metabolites also significantly impacted by flooding included spermidine (polyamines) (Supplementary Table 1).

### Metabolic Differences between *Spodoptera exigua* (Generalist) and *Manduca sexta* (Specialist) Herbivory Treatments

Several metabolites contributed to significant (*P* < 0.05) differences between herbivory by the specialist *Manduca sexta* and the generalist *Spodoptera exigua*. Most notable were the secondary sulfur-containing metabolites including 2-hydroxybenzothiazole and N-cyclohexyl-2-benzothiazol-amine (Fig. 1h). Herbivory by the specialist caterpillar species *Manduca sexta* significantly elevated these two metabolites in the heirloom tomato. On the contrary, these metabolites were significantly elevated by the generalist caterpillar species *Spodoptera exigua* in the hybrid tomato (Fig. 1h). Other metabolites that were differentially accumulated in response to herbivory by specialist and generalist herbivore species, particularly in the heirloom tomato variety also included FA 9:2 + 10 (Keto acids and derivatives), glycerate, betaine, taurine, tomatidine, aconitic acid, Dicyclohexylamine, 3-Hydroxyphenylacetic acid (phenol) which were significantly elevated in response to the specialist and not generalist in the heirloom tomato (Fig. 1g) (Supplementary Table 1) (Supplementary Table 2).

Additionally, when analyzed separately to determine if *M. sexta* triggered more metabolites than the generalist, we found that in CP, compared to control, and at 1-fold change threshold, herbivory by the specialist led to significant up-accumulation of 86 metabolites and down-accumulation of 82 metabolites (Supplementary Fig. 4), whereas, herbivory by the generalist, resulted in the up-accumulation of 101 metabolites and the down-accumulation of 67 metabolites (Supplementary Fig. 5). With respect to the hybrid variety, specialist herbivory resulted in the up accumulation of 90 metabolites and down-accumulation of 78 metabolites (Supplementary Fig. 6), whereas herbivory by the generalist resulted in the up accumulation of 44 metabolites and the down accumulation of 124 metabolites (Supplementary Fig. 7).

### Metabolic Differences between Single and Stress Combination Treatments

The simultaneous presence of flooding and herbivory by specialist and generalist caterpillar species resulted in metabolic responses that were different from those documented in single stresses alone. These modified metabolic responses either reflected a synergistic (metabolic response in stress combination treatments was greater than the expected sum of individual stress responses), additive (metabolic response in stress combination treatments equaled to the sum of responses documented in individual treatments) antagonistic (metabolic response in stress combination treatments was less than that documented in individual treatments, with one treatment cancelling out or weakening the effect of the other treatment), neutral (no significant difference between individual and stress combination metabolic response) or metabolic response being dominated by one stressor (metabolic response in stress combination treatment closely resemble the response of the dominant stress).

Metabolites exhibiting significant synergistic metabolic responses under stress combination (*P* < 0.05) included indole, 3-formylindole, tryptophan, abscisic acid, 4-hydroxyquinoline, tyrosine, and rutin (Fig. 1c) (Fig. 1f) (Supplementary Table 1). In contrast, antagonistic metabolic responses were documented for 2-hydroxybenzothizole and N-cyclohexyl-2-benzothiazole-amine (Fig. 1h). Metabolites showing an additive metabolic response included naringenin and kaempferol-3-O-rutinoside (flavonoids), and 9-HOTrE, 9-HODE, FA 18:4 + 10, (Z)-5,8,11-trihydroxyoctadec-9-enoic acid (Fatty acyls), and solasodine and tomatidine (steroidal glycoalkaloids) (Fig. 1g) (Fig. 1h) (Supplementary Table 1). Finally, metabolites reflecting a dominant stress metabolic response included alpha-Methylene-gamma-butyrolactone, glyceraldehyde, myo-inositol, pyrogallol, cuminyl alcohol, proline, and epigallocatechin (Fig. 1c) (Supplementary Table 2).

### Heirloom Cultivar Produced Greater Stress Induced Differences in Comparison to the Hybrid on Average

Based on metabolic response analyses, we found that the levels of several putatively identified metabolites, particularly in flooding and insect herbivory stress combination treatments were significantly higher in the heirloom tomato variety compared to the hybrid. These metabolites included coumaric acid (isomer of 192, 194), caffeic acid hexoside, p-coumaric acid, tyrosine, indole, tryptophan, solasodin, FA 18:4 + 10, kaempferol-3-O-rutinoside, quercetin, rutin, L-Sorbose (Fig. 1c) (Fig. 1d) (Fig. 1f) (Fig. 1g) (Supplementary Table 2). For single stress treatments, specifically herbivory by specialist and generalist caterpillar species metabolites that were significantly higher in heirloom and not hybrid included (Z)-5, 8,11-trihydroxyoctadec-9-enoic acid, 9-HOTrE, 9-HODE, 8-[3-oxo-2-[E-pent-2-enyl] cyclopenten-1-yl] octanoic acid (Fig. 1f) (Fig. 1h) (Supplementary Table 1) (Supplementary Table 2). With respect to single stress of flooding, metabolites that were significantly higher in heirloom and not hybrid included pyrogallol [phenols], α-methylene-γ-butyrolactone [lactones], myo-inositol, proline and tomatidine (Fig. 1c) (Fig. 1g).

### Plant Chemistry (Volatile Organic Compounds)

A total of 23 Volatile Organic Compounds (VOCs) were detected, identified, and quantified using GC-MS from aboveground headspace of tomato plants (Supplemental Fig. 2). These included 2 green leaf volatiles (hexanal and 2-hexanal), 12 monoterpenes (α-pinene, o-cymene, β-pinene, (+)-4-carene, α-terpinene, β-phellandrene, trans-β-ocimene, p-cymene, β-ocimene, γ-terpinene, α-terpinolene, 2-carene), 1 aldehyde (nonanal), 1 benzoate ester (methyl salicylate) and 7 sesquiterpenes (δ-elemene, copaene, β-elemene, caryophyllene, δ-elemene, humulene, germacrene).

**Fig. 2:**
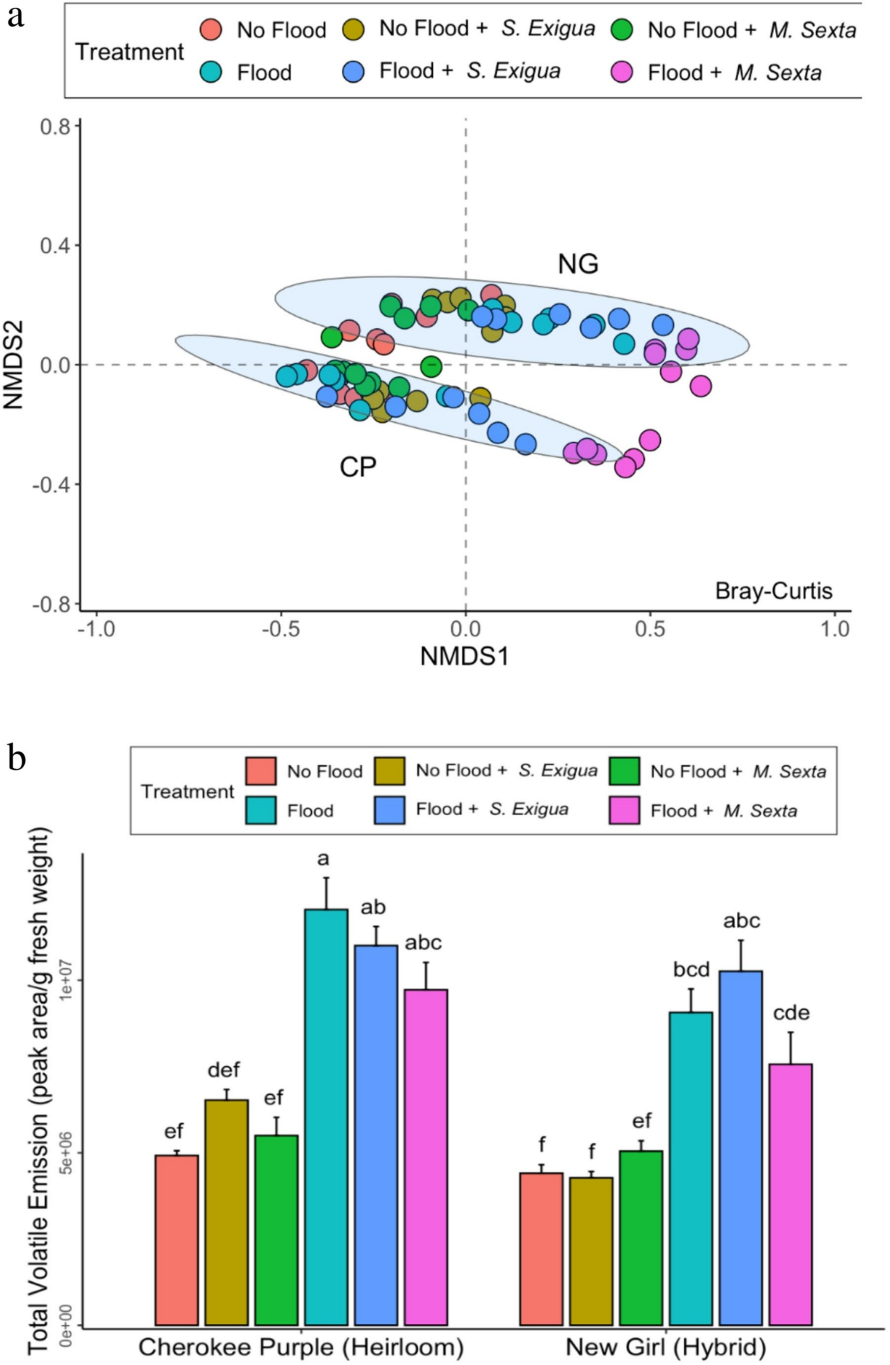
**a.** Non-metric dimensional scaling (NMDS) ordination plot of total volatile organic compound emission in peak area per g fresh weight (*n* = 6) of the heirloom and hybrid tomato varieties using Bray-Curtis dissimilarity matrix from the vegan package. Clusters of VOC emissions of the heirloom and hybrid are represented by “CP” and “NG” respectively. Circles indicate multivariate t-distribution data ellipses generated using the stat_ellipse() function of the ggplot2 package in R. **b**. Total headspace volatile organic compound emission of heirloom and hybrid tomato varieties subjected to flooding, herbivory by the specialist (*M. sexta*) and generalist (*S. exigua*) caterpillar species and their combination expressed in peak area per g fresh weight (*n* = 6) with standard error bars. Letters indicate significantly different treatments from three-way ANOVA using Tukey’s honestly significant difference testes (*P* < 0.05)

Three-way ANOVA analysis of variance on total VOC emissions revealed significant differences for variety, flooding, and flooding x herbivory interaction (Table 1). Flooding alone is responsible for significant increases in the total volatile emission of both heirloom and hybrid variety (*P* < 2E-16). The combination of flooding and herbivory significantly increased the total VOC emissions of both varieties (*P* = 0.0142), and heirloom variety emitted significantly greater quantities of volatiles than the hybrid variety across all treatments (*P* = 6.54E-05) (Fig. 2b) (Table 1). In addition to quantitative difference, the two varieties differ in the composition of their volatile blends, as evident by the separate clustering in the non-metric dimensional scaling (NMDS) bi-dimensional plot (Fig. 2a). Further, to understand which VOCs contribute to the compositional difference, Random Forest Model Analysis was performed which identified that α-terpinolene, α-terpinene, and γ-terpinene and seven other terpene molecules shaped the uniqueness of the volatile blend of the heirloom variety. On the other hand, trans-β-ocimene, β-pinene, β-phellandrene and seven others were the most important VOCs contributing to the volatile blend of the hybrid variety.

**Table 1:** Three-way analysis of variance (ANOVA) (*P* < 0.05) table showing the effects of variety, flooding, herbivory, and their interaction on the total volatile emissions of heirloom (Cherokee Purple) and hybrid (New Girl) tomato. (*n* = 6)

Factors	Degrees of freedom	Sum of squares	*R* ^2^	F-value	*P*-value
Variety	1	4.14E + 13	4.14E + 13	18.429	**6.54E-05**
Flooding	1	4.21E + 14	4.21E + 14	187.119	**<2e-16**
Herbivory	1	1.37E + 13	6.83E + 12	3.039	0.0553
Variety: Flooding	1	3.58E + 12	3.58E + 12	1.591	0.212
Variety: Herbivory	2	5.91E + 11	2.96E + 11	0.132	0.877
Flooding: Herbivory	2	2.05E + 13	1.03E + 13	4.57	**0.0142**
Variety: Flooding: Herbivory	2	1.34E + 13	6.69E + 12	2.977	0.0585
Residuals	60	1.35E + 14	2.25E + 12		

### Plant Above and Belowground Growth Characteristics

**Fig. 3:**
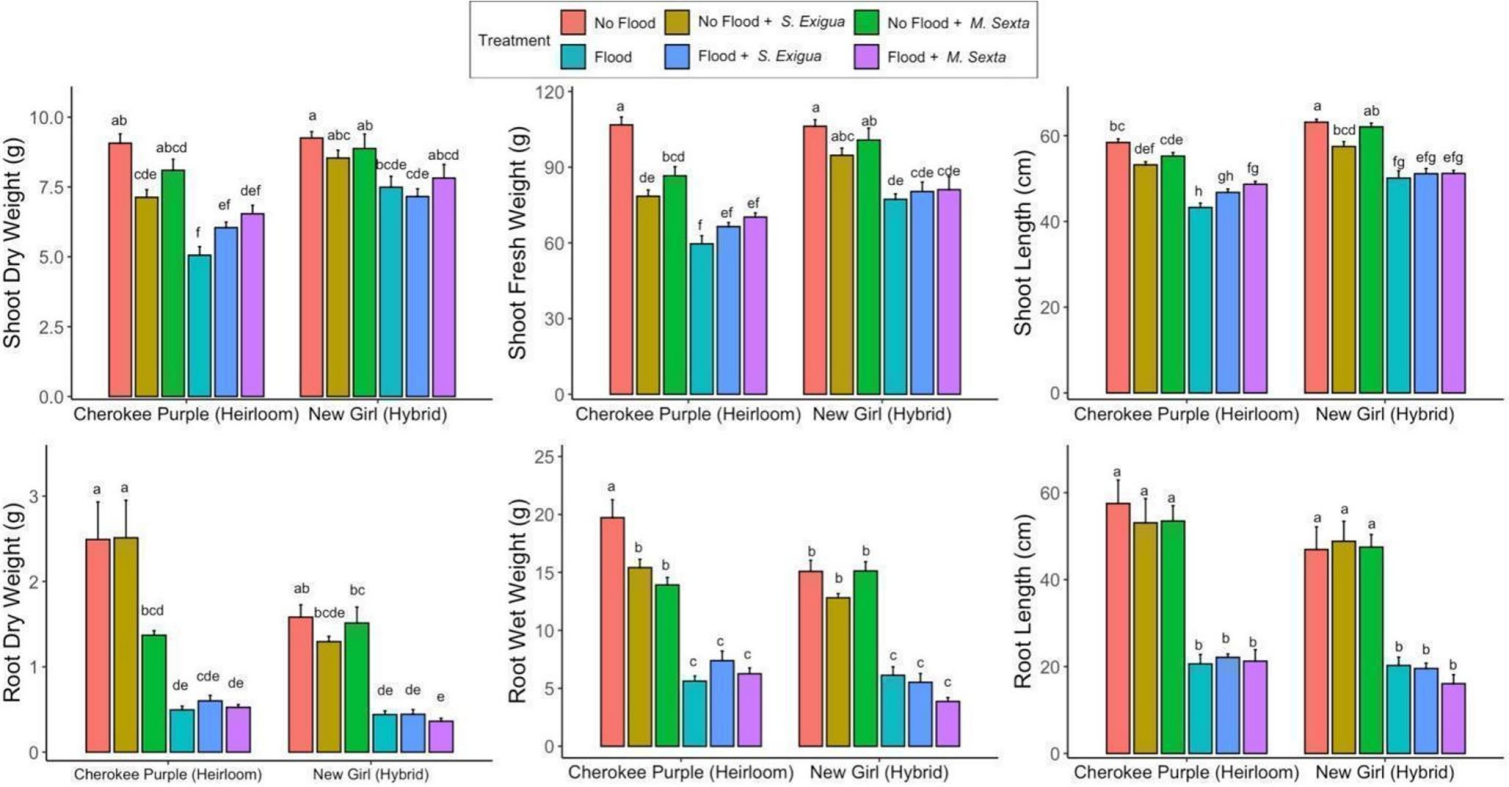
Plant growth parameters (Shoot dry and fresh weight in grams, shoot length in centimeters, root dry and fresh weight in grams, root length in centimeters) of heirloom (Cherokee Purple) and hybrid (New Girl) exposed to flooding, herbivory by the specialist (*M. sexta*) and generalist (*S. exigua*) caterpillar species and their combination. Letters indicate significantly different treatments from three-way ANOVA using Tukey’s honestly significant difference testes (*P* < 0.05). *n* = 6

Aboveground, in the heirloom tomato variety, flooding, alone or combined with insect herbivory significantly (*P* < 0.05) reduced shoot fresh and dry weight and shoot length (Fig. 3). On the contrary, we did not document a significant reduction of shoot fresh and dry weight in the hybrid variety. Belowground, in both the heirloom and hybrid tomato variety, both isolated flooding and the stress flooding and insect herbivory in combination significantly reduced root dry and fresh weight and root length. Additionally, herbivory by *Manduca sexta* significantly reduced root dry weight in the heirloom variety. In the heirloom variety, herbivory by both *Spodoptera exigua* and *Manduca sexta* significantly decreased root fresh weight (Fig. 3).

## Discussion

This study assessed the individual and combined effects of flooding and herbivory by specialist (*M. sexta*) and generalist (*S. exigua*) insects on the metabolome, VOC emissions, and growth of two tomato varieties: heirloom (Cherokee Purple) and hybrid (New Girl). Insect herbivory and flooding impose distinct physiological and metabolic constraints on plants, and we therefore expected to document differences in metabolite accumulation and VOC emission patterns when these stressors occur individually versus simultaneously, with these responses further being shaped by plant variety and breeding history (Suzuki et al. 2014; Zhou et al. 2015; Schuman and Baldwin^2015^; Paudel et al. 2019; Havko et al. 2020; FAO 2023; Bailey-Serres et al. 2008; Zandalinas et al. 2022). Overall, our results revealed that flooding was the dominant factor, reprogramming the tomato metabolome and significantly increasing VOC emissions. Flooding also reduced belowground root biomass. In the heirloom tomato variety, the generalist altered many more metabolites than the specialist, a trend that was reversed in the hybrid variety. The most notable differences in metabolite accumulation between generalist- and specialist-induced changes were observed in secondary sulfur-containing metabolites. We further documented significant differences in metabolite accumulation patterns between single-stress and stress-combination treatments, with both synergistic, additive, and antagonistic metabolic response outcomes. Finally, across treatments, several of the putatively identified metabolites accumulated in greater amounts in the heirloom variety. Notable differences were also documented in VOC emission patterns across varieties and single- and stress-combination treatments, with monoterpenes including β-pinene, trans-β-ocimene, and β-phellandrene shaping VOC differences among treatments (Table 2).

**Table 2:** Importance ranking of the top 10 volatile organic compounds (VOCs) emitted from the heirloom (Cherokee Purple) and hybrid (New Girl) ranked by their percent (%) increase in mean square error (MSE) from the Random Forest algorithm

Rank	Cherokee Purple-volatile compounds	%IncMSE	New Girl-volatile compounds	%IncMSE
1	α-terpinolene	11.43	trans-β-ocimene	10.45
2	α-terpinene	10.71	β-pinene	10.41
3	γ-terpinene	10.59	β-phellandrene	9.19
4	β-phellandrene	10.15	2-carene	9.04
5	α-pinene	9.61	γ-terpinene	7.19
6	2-carene	8.67	α-terpinolene	7.07
7	4-carene	8.44	β-ocimene	6.04
8	caryophyllene	3.88	o-cymene	5.9
9	p-cymene	3.19	4-carene	5.78
10	nonanal	2.58	α-terpinene	5.31

Flooding deprives plants of oxygen, forcing a shift from aerobic to anaerobic respiration and results in a significant reorganization of the metabolome to maintain basic energy needs during oxygen deprivation (Bailey-Serres et al. 2008; Voesenek and Bailey-Serres 2015; Safavi-Rizi et al. 2020b; De Ollas et al. 2021; Zandalinas et al. 2022). Given the severe constraints flooding imposes them and based on previous flooding studies, we hypothesized that flooding would be the dominant stressor driving metabolic reprogramming. We further expected it to increase the accumulation of metabolites with stress-protective functions while decreasing the abundance of defense-related metabolites (Zandalinas et al. 2022). Consistent with our hypothesis, flooding was the primary driver of metabolic reprogramming in this study. Flooding strongly increased the abundance of indole-derived metabolites, including indole, 3-formylindole, tryptophan, pyrogallol, 4-hydroxyquinoline, p-hydoxycoumaric acid and naringenin. De Ollas et al. (2021) profiled the metabolic responses of tomato to flooding stress and reported the accumulation of several metabolites in tomato leaves, including tryptophan, and our results agree with this previous study. Indole-derived metabolites have been reported in previous studies to play diverse stress-mitigating functions, such as priming, activation of their immune system and defense signaling, and to confer increased defenses against ecological stressors (Erb et al. 2015; Stahl et al. 2016; Ye et al. 2019; Hu et al. 2019). Moreover, previous studies have also reported that these metabolites play other stress-mitigating roles, including redox buffering, regulation of antioxidant enzyme activity, detoxification of activated harmful oxygen species, acting as precursors for important stress-mitigating compounds such as auxin and melatonin, and providing protection against pathogens (Loewus and Murthy 2000; Ramani and Chelliah 2007; Roepke et al. 2017; Mehle et al. 2012; Marta et al. 2016; Dey et al. 2020; Zandalinas et al. 2022; Manzoor et al. 2023). Flooding also increased levels of α-methylene-γ-butyrolactone, a metabolite previously reported to have antifungal properties (He et al. 2023; Chen et al. 2024). Although we did not directly determine the exact functions of these metabolites elevated under flooding, their above well-documented roles in previous studies allow us to speculate they are performing similar functions. Flooding also reduced the accumulation of several defense-related metabolites, such as allantoin, 3-hydroxycinnamic acid, fatty acyls 9-HOTrE and 9-HODE, and spermidine, and previous flooding studies have also reported decreased accumulation of metabolites (Fig. 1e) (Coutinho et al. 2018; De-Ollas et al., 2021; Umicevic et al., 2024). These dynamic changes in metabolite accumulation patterns documented in our study reflect significant metabolic reprogramming following flooding, a pattern previously reported in previous studies including some involving tomato (Safavi-Rizi et al. 2020b; De-Ollas et al., 2021; Umicevic et al., 2024). Uncovering these dynamic changes is a foundational step that paves the way for future studies to comprehensively determine the implications of increases or decreases in certain metabolites while establishing the metabolic pathways that underpin flooding tolerance.

In our study, we used a specialist and a generalist caterpillar species. Based on previous studies showing that herbivore characteristics, including dietary specialization, can shape plant metabolic responses, we hypothesized that the generalist caterpillar species would alter more metabolites and lead to higher accumulation of herbivore-triggered metabolites than the specialist, which has evolved mechanisms to suppress or tolerate defensive metabolites (Steinbrenner et al. 2011; Ali and Agrawal 2012). We found differences in metabolic responses triggered by the two caterpillar species, with the most notable differences between the specialist *Manduca sexta* and the generalist *Spodoptera exigua* documented for sulfur-containing secondary metabolites, including 2-hydroxybenzothiazole and *N*-cyclohexyl-2-benzothiazol-amine (Fig. 1h). These metabolites have been previously reported to have antimicrobial, antifungal, and antibacterial activities and to be involved in defense priming. Although we did not functionally validate their role in our study, future studies will aim to determine their ecological relevance, including whether they also differentially suppress the growth and performance of these caterpillars (Kessler and Baldwin 2002; Bosch et al. 2014; Gandhi et al. 2020; Simonova et al. 2005; Hu et al. 2009; Mei et al. 2019; Zou et al. 2023). In addition, our results revealed that the generalist *S. exigua* drove most induced metabolic responses, consistent with our hypothesis and prior reports that generalist herbivores often elicit broader plant chemical responses than specialists (Steinbrenner et al. 2011). Furthermore, our results also revealed that the generalist *S. exigua* induced greater accumulation of fatty acyl oxylipin precursors 9-HOTrE and 9-HODE. These fatty acid derivatives are established precursors to oxylipins, including jasmonic acid, a central regulator of plant responses to both herbivory and abiotic stress (Andreou et al. 2009; Reinbothe et al. 2009; Woldemariam et al. 2018; Deboevere et al. 2020; Wang et al. 2021). Surprisingly, in our study, we did not document significant differences between control and specialist and generalist herbivore treatments with respect to metabolites associated with carbon and nitrogen mobilization and several secondary metabolites associated with anti-herbivore defense in tomato, such as rutin, quercetin, and solasodine, as has been reported in previous studies (Fig. 1g) (Steinbrenner et al. 2011; Schwachtje et al. 2006). Several potential reasons could explain this discrepancy, including the number of herbivores used in our study and the time of tissue sampling for metabolite profiling. Future untargeted and targeted metabolite profiling studies that incorporate different densities of specialist and generalist caterpillars and sample tissues at several time points after insect herbivory will help resolve these differences.

In agricultural environments, crops are often exposed to multiple stressors simultaneously (Suzuki et al. 2014; Havko et al. 2020; Ngumbi et al. 2022; Mittler et al. 2025). When biotic and abiotic stressors that impose contrasting physiological and metabolic constraints co-occur, their metabolic responses are often markedly different from those observed under single stresses, and the resulting response can be additive, synergistic, or antagonistic (Martinez et al. 2018; Havko et al. 2020; Vescio et al. 2022; Zandalinas et al. 2020). Consistent with these expectations, we found that although flooding and herbivory each induced distinct metabolic changes, when both stresses co-occurred, metabolites that were often increased by flooding, including tryptophan, tyrosine, naringenin, kaempferol-3-O-rutinoside (flavonoids), and 9-HOTrE, 9-hode, FA 18:4 + 10, and (Z)-5,8,11-trihydroxyoctadec-9-enoic acid (fatty acyls), showed additive, synergistic, or neutral responses. These synergistic and additive metabolic responses suggest that combining stresses may amplify plants’ metabolic responses. Although the functional significance of the enhanced accumulation of indole-derived metabolites, flavonoids, and fatty acyls remains to be established, previous studies have demonstrated that these metabolites are multifunctional and promote stress resistance by playing versatile roles, including scavenging harmful reactive oxygen species, regulating plant growth, enhancing the antioxidant system, increasing herbivore resistance, and priming defenses against other potential biotic threats such as pathogens (Erb et al. 2015; Shen et al. 2018; Ye et al. 2019). In contrast, metabolites such as 2-hydroxybenzothizole and N-cyclohexyl-2-benzothiazole-amine, which were significantly elevated under herbivory alone, exhibited antagonistic responses when herbivory occurred in combination with flooding. This was reflected in the downregulation of these metabolites, suggesting that flooding may compromise plant metabolic responses to insect herbivory when the two stresses co-occur. Antagonistic responses when flooding and insect herbivory co-occur have been reported in several previous studies involving crops such as maize and *Solanum dulcamara* (Nguyen et al. 2016a, b; Block et al. 2020; Lee et al. 2020; Ngumbi et al. 2022). Nguyen et al. (2016a, b), for example, showed that in *Solanum dulcamara*, flooding repressed various defense responses before and after insect herbivory and suggested that flooding-induced suppression could be due to the lack of detectable jasmonic acid levels in flooded crops, a finding that has been reported in other flooding studies (Lee et al. 2020). It is also possible that this antagonistic response is driven by ethylene, which is elevated during flooding (Lee et al. 2020; Xiong et al. 2026). Previous studies have shown that ethylene can suppress the jasmonic acid pathway, reducing anti-herbivore capacity (Lee et al. 2020). Ultimately, future studies are needed to determine whether this antagonistic response is driven by hormonal crosstalk and to assess the subsequent ecological implications of the additive, synergistic, and antagonistic metabolic responses documented in our study.

Cultivar variety and breeding history can further influence the strategies crops use to cope with individual and simultaneously occurring biotic and abiotic stress, including metabolic reprogramming. Previous studies have overwhelmingly shown that breeding for improved yields and fruit quality often involves trade-offs with defense and stress mitigation strategies in cultivated crops (Zhu et al. 2018; Paudel et al. 2019; Dady et al. 2023). We therefore expected to find differences in metabolic responses between heirloom and hybrid tomato varieties representing two breeding histories. Consistent with our expectations, our results showed that the heirloom tomato variety exhibited more distinct metabolic reprogramming than the hybrid variety. The most notable trend was the increased accumulation of several metabolites, including coumaric acid, α-methylene-γ-butyrolactone, proline, myo-inositol, and pyrogallol, in the heirloom tomato variety compared with the hybrid (Fig. 1d). Although we did not functionally validate the biological functions of these metabolites, previous studies have revealed that metabolites such as proline and myo-inositol play versatile roles in mitigating biotic and abiotic stressors, including drought, cold stress, and flooding, through multiple mechanisms, including scavenging harmful reactive oxygen species, stress signaling, stabilizing proteins and enzymes, protecting protein integrity, and helping to maintain redox balance (Aloni and Rosenshtein 1982; Ashraf and Foolad 2007; Szabados 2010; Alok et al. 2022; Ikehara et al. 2025). For example, Aloni and Rosenshtein (1982) found a positive relationship between proline accumulation and flooding stress tolerance in a study of 8 tomato varieties. Varietal differences in metabolic accumulation patterns are likely driven in part by genetic variation arising from breeding and the trade-offs that come along with it (Gerszberg and Hnatuszko-Konka 2017; Tieman et al. 2017; Zhu et al. 2018; Paudel et al. 2019; Rezk et al. 2021). Overall, we conclude that breeding has modified plants’ metabolic response to abiotic and biotic stress and their combinations, and future studies are needed to validate the biological and stress mitigation implications of the elevated accumulation of metabolites.

Individual and simultaneously occurring biotic and abiotic plant stress factors, such as insect herbivory and drought and flooding, are known to increase or decrease the emission of volatile organic compounds (Holopainen and Gershenzon 2010; Paudel et al. 2019; Dady et al. 2023). Furthermore, herbivore dietary breadth, plant variety, and breeding history can influence the composition, quantity, and quality of emitted VOCs (Rowen and Kaplan 2016; Paudel et al. 2019; Dady et al. 2023). Once emitted, VOCs provide stress relief through several mechanisms, including directly repelling insect herbivores, recruiting natural enemies of insect herbivores, or alleviating oxidative stress (Holopainen and Gershenzon 2010; Ayelo et al. 2021; Dady et al. 2023). We therefore expected to document an increase in VOC emissions when tomatoes were exposed to the individual stresses of herbivory by generalist *S. exigua* and specialist *M. sexta*, and to flooding. Additionally, we expected that VOC emission patterns in stress combination treatments would reflect an additive, antagonistic, or stress-prioritization response, in which the most severe stress has the strongest impact on VOC emissions. Finally, based on previous tomato studies, we predicted that heirloom tomato varieties would have significantly higher total VOC levels than hybrid varieties (Dady et al. 2023). Our results revealed that tomato variety, flooding, and the interaction between flooding and herbivory, and not herbivory by either *M. sexta* or *S. exigua*, influenced volatile emissions. Flooding significantly increased VOC emissions, whereas herbivory suppressed them. This was true for all 23 detected volatiles. Additionally, when flooding occurred simultaneously with insect herbivory, results suggested that crops prioritized flooding, as total VOCs in flooding and insect-herbivory stress combination treatments did not differ significantly from flooding alone. Consistent with previous studies in both tomato and other crop species, including corn, flooding generally increases volatile emissions (Block et al. 2020; Ngumbi et al. 2022, 2025; Ngumbi and Ugarte 2021; Mleziva and Ngumbi 2024), and future studies are urgently needed to determine the physiological mechanisms that underpin this increase in VOC emissions, alongside studies to determine the ecological implications of flooding-induced increases in VOC emissions.

Notably, cultivar variety had a strong effect on VOC emissions, as evidenced by the separate clustering of heirloom and hybrid tomato varieties. We identified several terpenes, including beta-pinene, beta-ocimene, and phellandrene, as the most important VOCs contributing to these significant treatment differences. Our results, showing a strong varietal effect on VOC emission patterns in heirloom and hybrid tomatoes, align with previous studies reporting that, compared with hybrid tomatoes, heirloom tomato varieties such as Cherokee Purple emit significantly higher VOCs (Dady et al. 2023; Ngumbi et al. 2022, 2025). Varietal differences in volatile emission patterns have been reported in tomatoes and other crop species, and many studies suggest that breeding may contribute to these differences (Degen et al. 2004, 2012; Paudel et al. 2019; Dady et al. 2023). Breeding tomatoes for commercially relevant traits such as fruit size and shape, as well as other storage-related traits, often comes at a cost, manifested by decreased inducibility of plant defense and stress-mitigation traits, including volatiles (Rowen and Kaplan 2016; Tieman et al. 2017; Paudel et al. 2019). Therefore, the differences in VOC emissions recorded in our study between hybrid and heirloom tomatoes could have been attributed to breeding.

Moreover, several terpenoids, including nonanal, α-pinene, β-phellandrene, and trans-β-ocimene amongst other monoterpenoids were identified as compounds that may contribute to varietal and individual differences in VOC emission patterns, as well as to differences observed during treatment. Broadly, terpenoids play important biological, physiological, and ecological roles, including repelling insect herbivores, suppressing herbivore growth and performance (Shrivastava et al. 2015; Silva et al. 2017), attracting natural enemies (Ayelo et al. 2021), and relieving plants of oxidative stress (Zhou et al. 2023; Zhou et al. 2025). Although our current study did not validate the ecological significance of these terpenoids, future studies will seek to determine their functional importance, which was significantly elevated under flooding and stress combination treatments.

Compared with control treatments, insect herbivory did not increase total VOCs, even though herbivory is expected to increase VOC emissions. Our results are unsurprising, as previous studies have reported that specialist and generalist herbivores suppress volatile emissions in their hosts (Sarmento et al. 2011; Rowen and Kaplan 2016; De Lange et al. 2020). Furthermore, *Manduca sexta*, a specialist herbivore, has been reported to evade or suppress induced plant defense (Bosch et al. 2014; Gandhi et al. 2020). The mechanisms underlying the suppression observed in our study remain to be determined. However, evidence from previous studies suggests that elicitors and other compounds present in insect regurgitant and saliva, as well as in insect-associated gut bacteria, are involved (Sarmento et al. 2011; Jones et al. 2019).

To cope with biotic and abiotic stresses, plants reallocate resources from growth to defense and stress mitigation, creating trade-offs between these processes (Herms and Mattson 1992; He et al. 2019; Verslues 2025; Zrimec et al. 2025). Moreover, modern crops, including hybrid tomato, have been bred to maximize yields and growth-related traits at the expense of defense, which can create a dichotomy in how different tomato varieties’ growth characteristics are affected by biotic and abiotic stressors (Ferrero et al. 2020; Gao et al. 2023; Zrimec et al. 2025). Therefore, we expected to document significant changes in below- and above-ground growth characteristics, particularly under flooding and in stress combinations involving flooding, along with differences between hybrid and heirloom responses. In accordance with our expectation, flooding and the stress combination of flooding and insect herbivory significantly reduced belowground dry and fresh weights of roots in both varieties. These results agree with previous flooding studies in tomato and crop species including corn (Ngumbi and Ugarte 2021; Ngumbi et al. 2022, 2025; Mleziva and Ngumbi 2024). Reduction in root biomass by flooding is expected because roots are the first organs to suffer from oxygen depletion, which hinders aerobic respiration and cripples root growth and sustaining processes, including nutrient uptake (Luo et al. 2024; Daniel and Hartman 2024). Contrasting the patterns documented in belowground root biomass, aboveground, the hybrid tomato variety displayed greater tolerance to flooding, reflected in no significant impact on the reduction of both shoot wet and dry weight compared with the heirloom variety. This could potentially be due to the ecological trade-offs associated with the process of domestication and crop breeding (Whitehead et al. 2017). Modern and hybrid varieties, including tomato, are often bred to maintain and prioritize growth over defense, and it is possible that the hybrid variety prioritized growth (Whitehead et al. 2017). Alternatively, the hybrid tomato variety maintained higher aboveground biomass because it has superior and effective resource allocation traits (Zhou et al. 2023). Future experiments using an expanded number of varieties representing putatively different stages of tomato domestication, breeding history, and breeding program priorities are worth pursuing to comprehensively establish how abiotic and biotic stressors and their combinations influence the reallocation of resources from growth to defense and the trade-offs that exist.

Our results, showing that flooding and its interaction with specialist and generalist insect herbivory drive changes in metabolite accumulation and VOC emission patterns, have several ecological implications. Increased VOC emissions, particularly monoterpenes, can serve to repel and deter herbivores, attract and recruit natural enemies of insect herbivores, and prime neighboring plants for enhanced responses (Zhou et al. 2023; Ayelo et al. 2021; Raghava et al. 2010). Similarly, increased metabolites with anti-insect activity can suppress the growth and performance of insect herbivores (Altesor et al. 2014; Paudel et al. 2019). Additionally, the accumulation of metabolites associated with antioxidant activity and increased VOC emissions can directly and indirectly mitigate abiotic stress (Holopainen and Gershenzon 2010; Brilli et al. 2019). Collectively, these changes in metabolites and VOCs can relieve tomatoes of ongoing stress and contribute to overall fitness.

In summary, our study established that heirloom and hybrid tomato varieties exhibit distinct metabolic and biochemical responses to the unique stressors of flooding, herbivory, and their combination. Tomato varieties shaped by combinations of breeding, flooding, and stress showed significant changes in their metabolic responses and biochemical responses. Our findings complement previous work that has investigated how abiotic and biotic stress, individually and in combination, affect metabolites, volatile organic compounds, and plant growth patterns, and how these changes are further shaped by breeding (Paudel et al. 2019; Ngumbi et al. 2022; Dady et al. 2023). Notably, our findings lay the groundwork for future studies to determine the ecological consequences of metabolite reprogramming and elevated VOC emissions documented under flooding and under combined flooding and insect herbivory. Understanding the roles that metabolites and VOCs play in stress tolerance and integrating metabolic and ecological data would deepen our knowledge and improve our understanding of how crops respond to complex stress combinations. These insights can inform breeding crops capable of withstanding simultaneous abiotic and biotic stressors, thereby supporting sustainable agriculture under changing environmental conditions (Zhou et al. 2023; Chen et al. 2023).

## Electronic Supplementary Material

Below is the link to the electronic supplementary material.

Supplementary Material 1 Supplementary Fig. 1. Figures of all individual compounds emitted in heirloom and hybrid tomato varieties.

Supplementary Material 2 Supplementary Fig. 2. GC-MS chromatograms of heirloom and hybrid tomato varieties under different treatments and Mass Spectrums of major compounds identified in samples.

Supplementary Material 3 Supplementary Fig. 3. Pictures of plants under different treatments and the overall experimental scheme.

Supplementary figure 4 Supplementary figure 4 (PNG 2.08 MB)

Number of up-and down-accumulated metabolites in Cherokee Purple in response to feeding by the specialist caterpillar, *Manduca sexta* High Resolution Image (TIF 244 KB)

Supplementary Material 5 Supplementary Fig. 5. Number of up-and down-accumulated metabolites in Cherokee Purple in response to feeding by the generalist caterpillar, *Spodoptera exigua*

Supplementary Material 6 Supplementary Fig. 6. Number of up-and down-accumulated metabolites in New Girl in response to feeding by the specialist caterpillar, *Manduca sexta*

Supplementary Material 7 Supplementary Fig. 7. Number of up-and down-accumulated metabolites in New Girl in response to feeding by the generalist caterpillar, *Spodoptera exigua*

Supplementary Material 8 Supplementary Table 1. List of all identified metabolites and the parent groups identified metabolites belong to (LC-MS/MS analysis data)

Supplementary Material 9 Supplementary Table 2. List of significantly (*P* < 0.05) altered metabolites. Data were analyzed on MetaboAnalyst 6.0 software.

## Data Availability

Available upon request.

## Code Availability

Available upon request.
