# Supplementary material for: Metabolic and Biochemical Responses of Heirloom and Hybrid Tomato (Solanum lycopersicum) Under Flooding, Specialist, and Generalist Insect Herbivory, and their Stress Combination
Source: J Chem Ecol. 2026 Apr 2;52(2):32. doi: 10.1007/s10886-026-01703-9 (PMC13046590; doi:10.1007/s10886-026-01703-9)
Supplement: Supplementary file 1 — Supplementary Fig. 1. Figures of all individual compounds emitted in heirloom and hybrid tomato varieties. [file 10886_2026_1703_MOESM1_ESM.pdf]

Supplementary Fig 1.
individual volatile emission

Headspace Volatile Organic Compounds (VOCs) emission of 23 compounds categorized into green leaf volatiles, monoterpene, benzoate easter, and sesquiterpenes collected from the two tested tomato varieties:
CP:Cherokee Purple (Heirloom), NG:New Girl (Hybrid)
VOCs emission expressed in peak area per gram of fresh weight

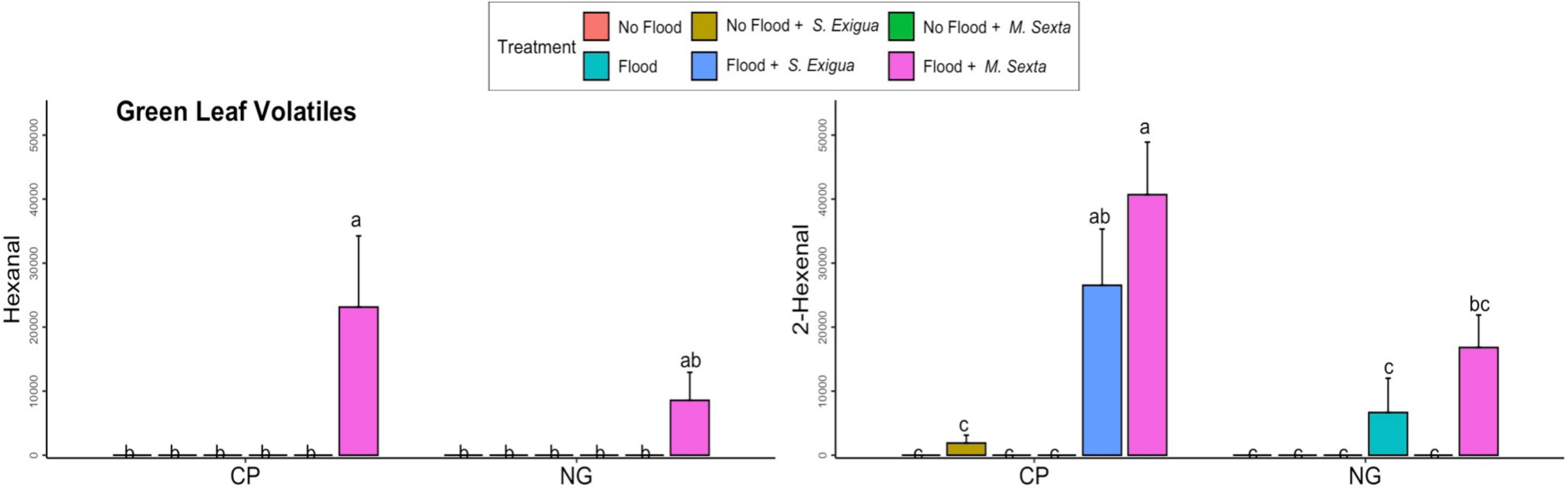
Treatment
No Flood
No Flood + S. Exigua
No Flood + M. Sexta
Flood
Flood + S. Exigua
Flood + M. Sexta
Green Leaf Volatiles
Hexanal
2-Hexenal
CP
NG
a
ab
bc
c

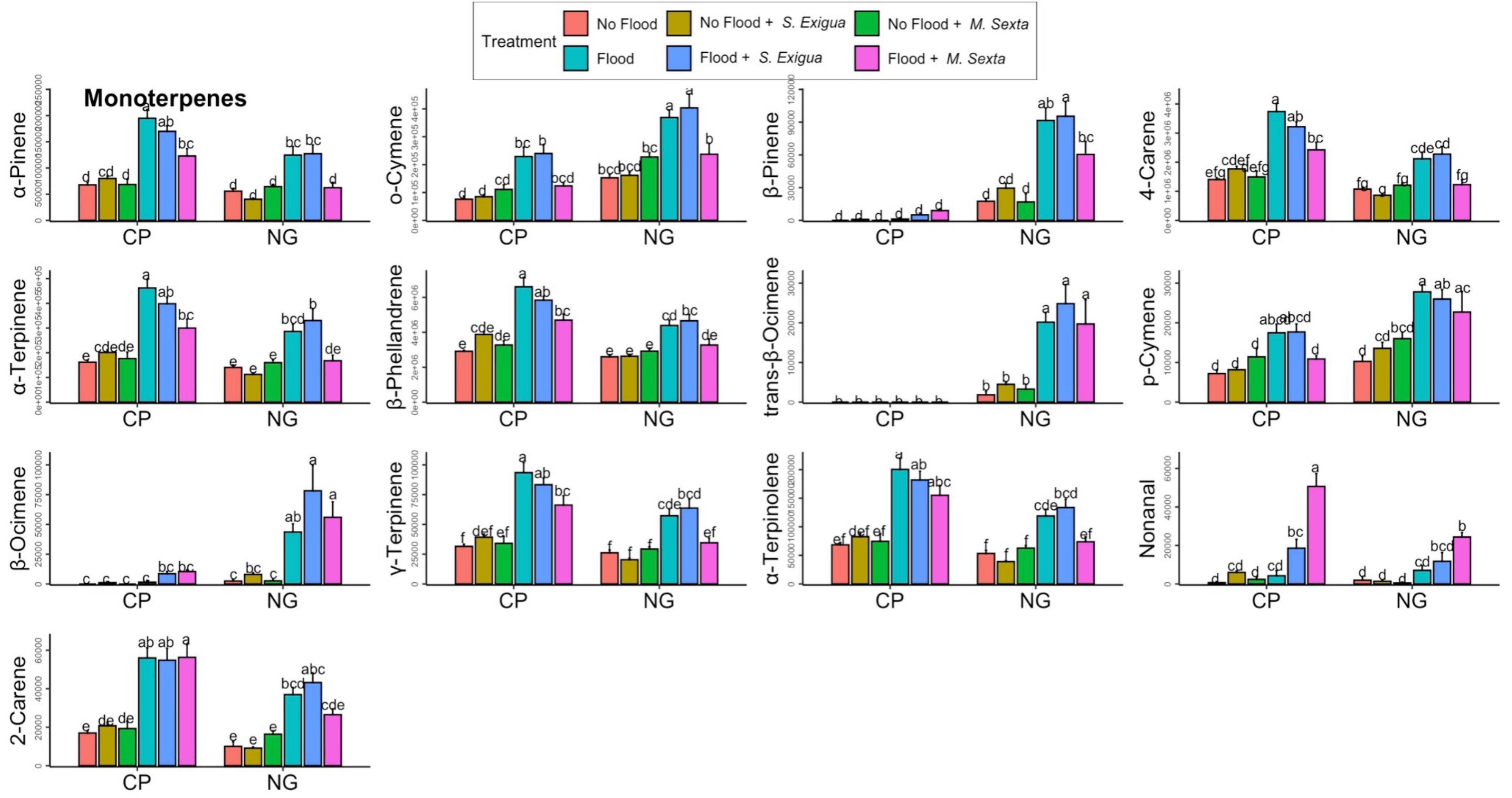
Treatment
No Flood
No Flood + S. Exigua
No Flood + M. Sexta
Flood
Flood + S. Exigua
Flood + M. Sexta
Monoterpenes
α-Pinene
o-Cymene
β-Pinene
4-Carene
α-Terpinene
β-Phellandrene
trans-β-Ocimene
p-Cymene
β-Ocimene
γ-Terpinene
α-Terpinolene
Nonanal
2-Carene
CP
NG

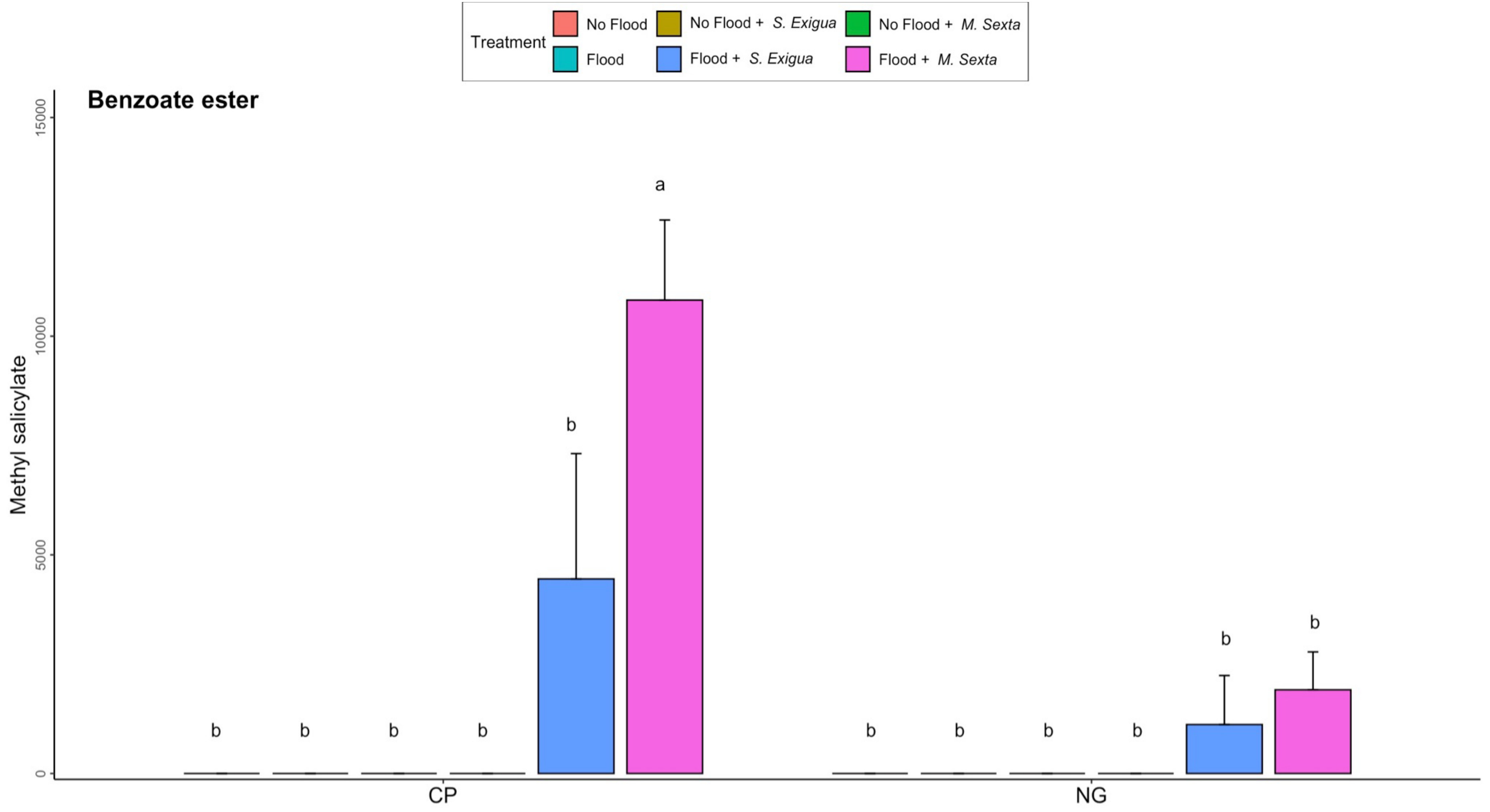
Treatment
No Flood
Flood
No Flood + S. Exigua
Flood + S. Exigua
No Flood + M. Sexta
Flood + M. Sexta
Benzoate ester
Methyl salicylate
15000
10000
5000
0
a
b
CP
NG

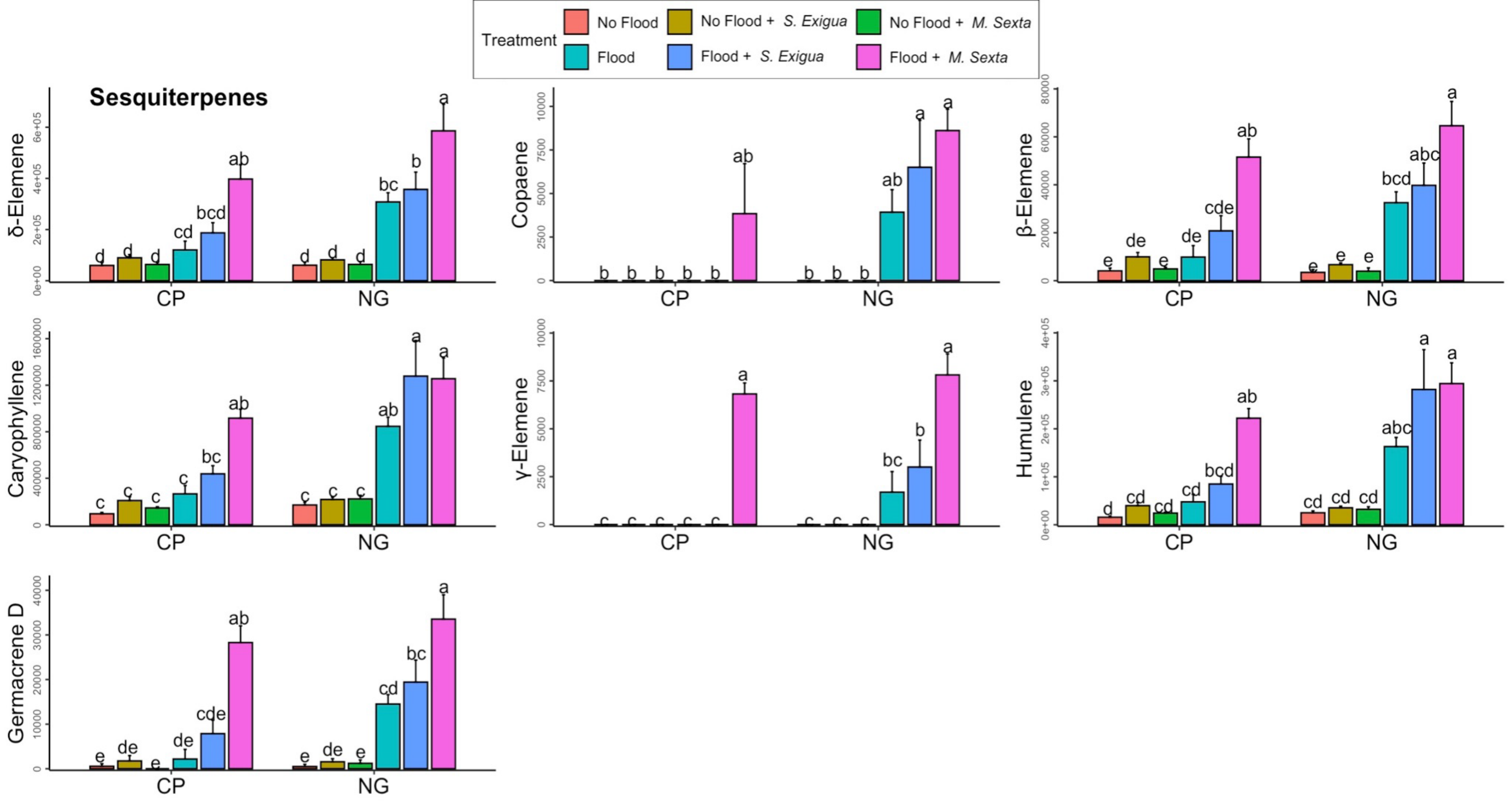
Treatment
No Flood
No Flood + S. Exigua
No Flood + M. Sexta
Flood
Flood + S. Exigua
Flood + M. Sexta
Sesquiterpenes
δ-Elemene
Copaene
β-Elemene
Caryophyllene
γ-Elemene
Humulene
Germacrene D
CP
NG
