# Supplementary material for: Metabolic and Biochemical Responses of Heirloom and Hybrid Tomato (Solanum lycopersicum) Under Flooding, Specialist, and Generalist Insect Herbivory, and their Stress Combination
Source: J Chem Ecol. 2026 Apr 2;52(2):32. doi: 10.1007/s10886-026-01703-9 (PMC13046590; doi:10.1007/s10886-026-01703-9)
Supplement: Supplementary file 2 — Supplementary Fig. 2. GC-MS chromatograms of heirloom and hybrid tomato varieties under different treatments and Mass Spectrums of major compounds identified in samples. [file 10886_2026_1703_MOESM2_ESM.pdf]

Supplementary Figure 2

-GC-MS chromatograms of heirloom and hybrid tomato varieties under different treatments

-Mass Spectrums of major compounds

Cherokee Purple (heirloom)
No Flood
No Herbivory

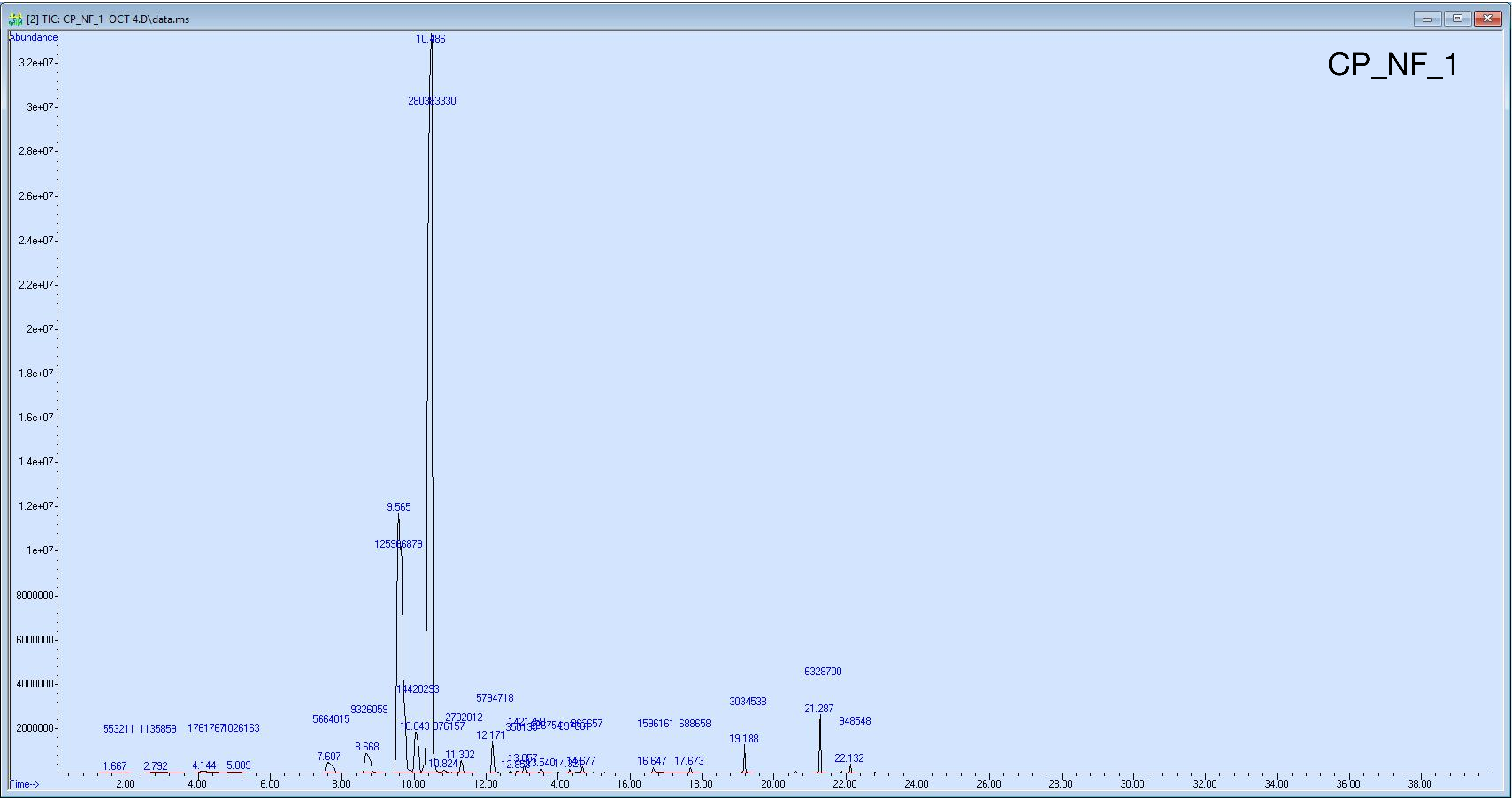

[2] TIC: CP_NF_1 OCT 4.D\data.ms
CP_NF_1
Abundance
Time-->
10.486
280383330
9.565
125986879
14420293
5794718
2702012
6328700
3034538
21.287
948548
19.188
22.132
1596161
688658
16.647
17.673
553211
1135859
1.667
2.792
4.144
5.089
5664015
9326059
7.607
8.668
12.171
11.302

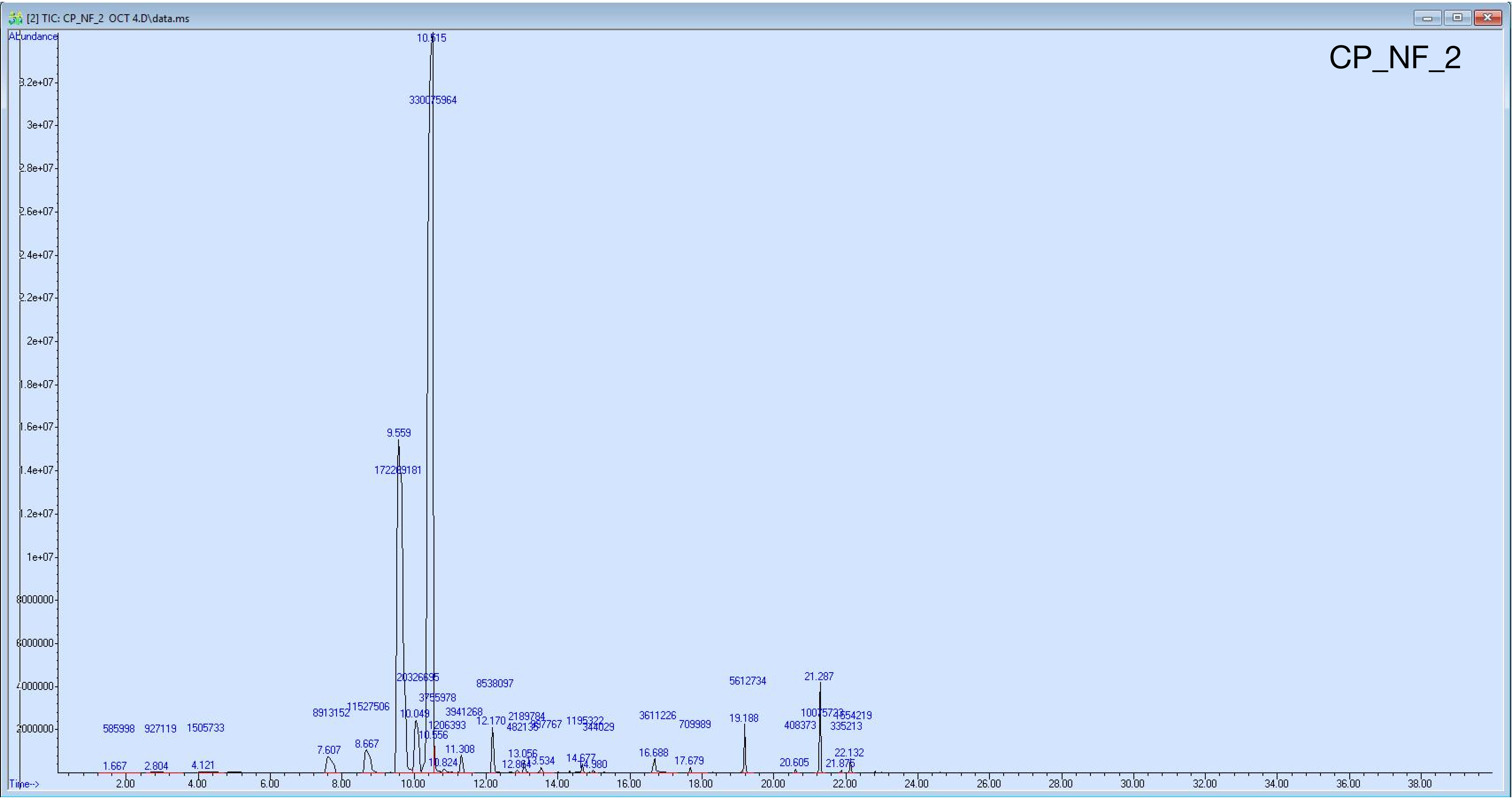
[2] TIC: CP_NF_2 OCT 4.D\data.ms
CP_NF_2
Abundance
Time-->
10.515
330075964
9.559
172289181
21.287
19.188
5612734
12.170
8538097
16.688
3611226

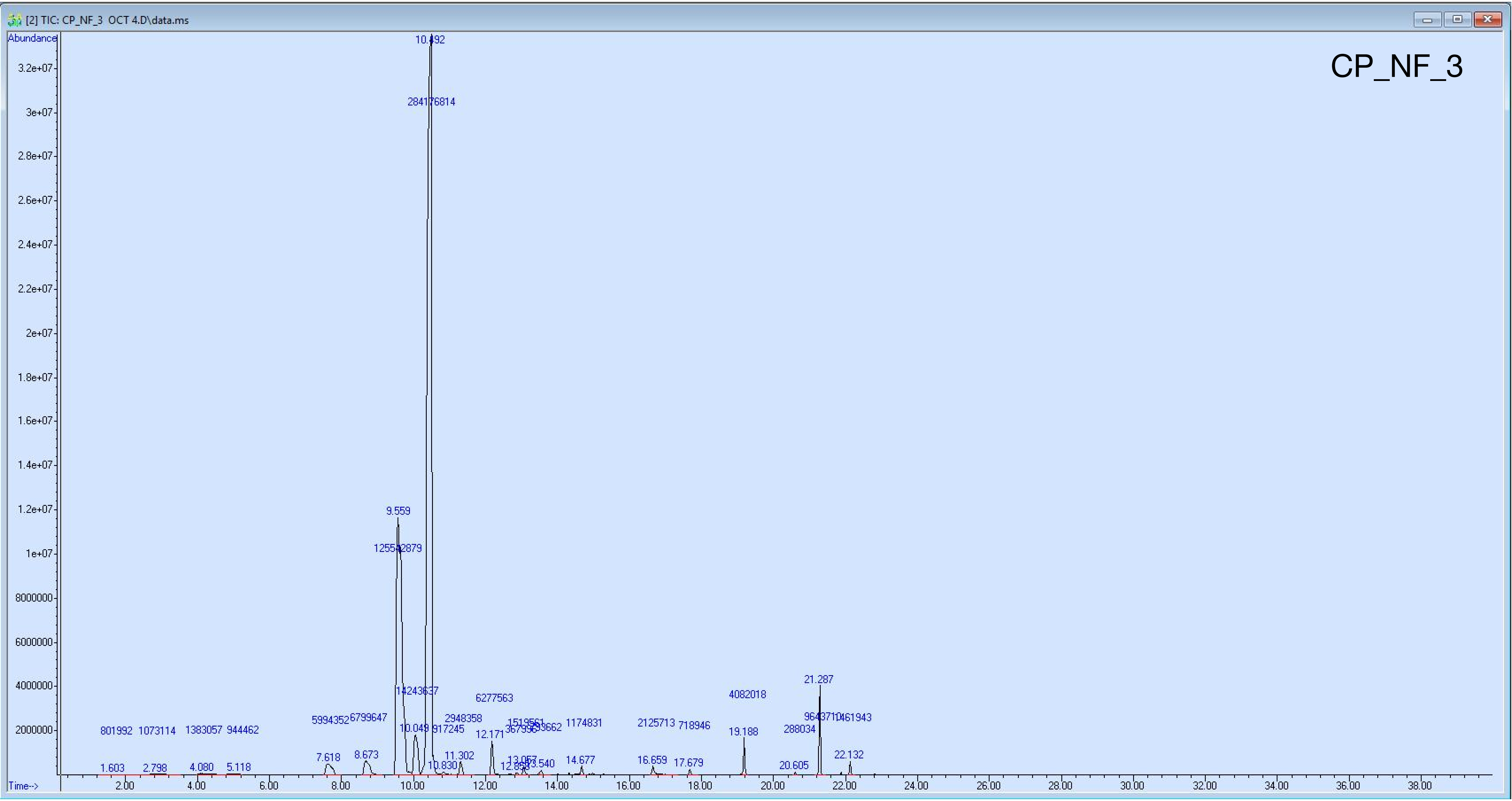
[2] TIC: CP_NF_3 OCT 4.D\data.ms
CP_NF_3
Abundance
Time-->
10.492
284176814
9.559
12554287
14243637
10.049
917245
2948358
10.830
11.302
6277563
12.171
1174831
14.677
2125713
16.659
718946
17.679
4082018
19.188
21.287
288034
20.605
22.132
7.618
5994352
8.673
6799647
1.603
801992
2.798
1073114
4.080
1383057
5.118
944462
2.00
4.00
6.00
8.00
10.00
12.00
14.00
16.00
18.00
20.00
22.00
24.00
26.00
28.00
30.00
32.00
34.00
36.00
38.00

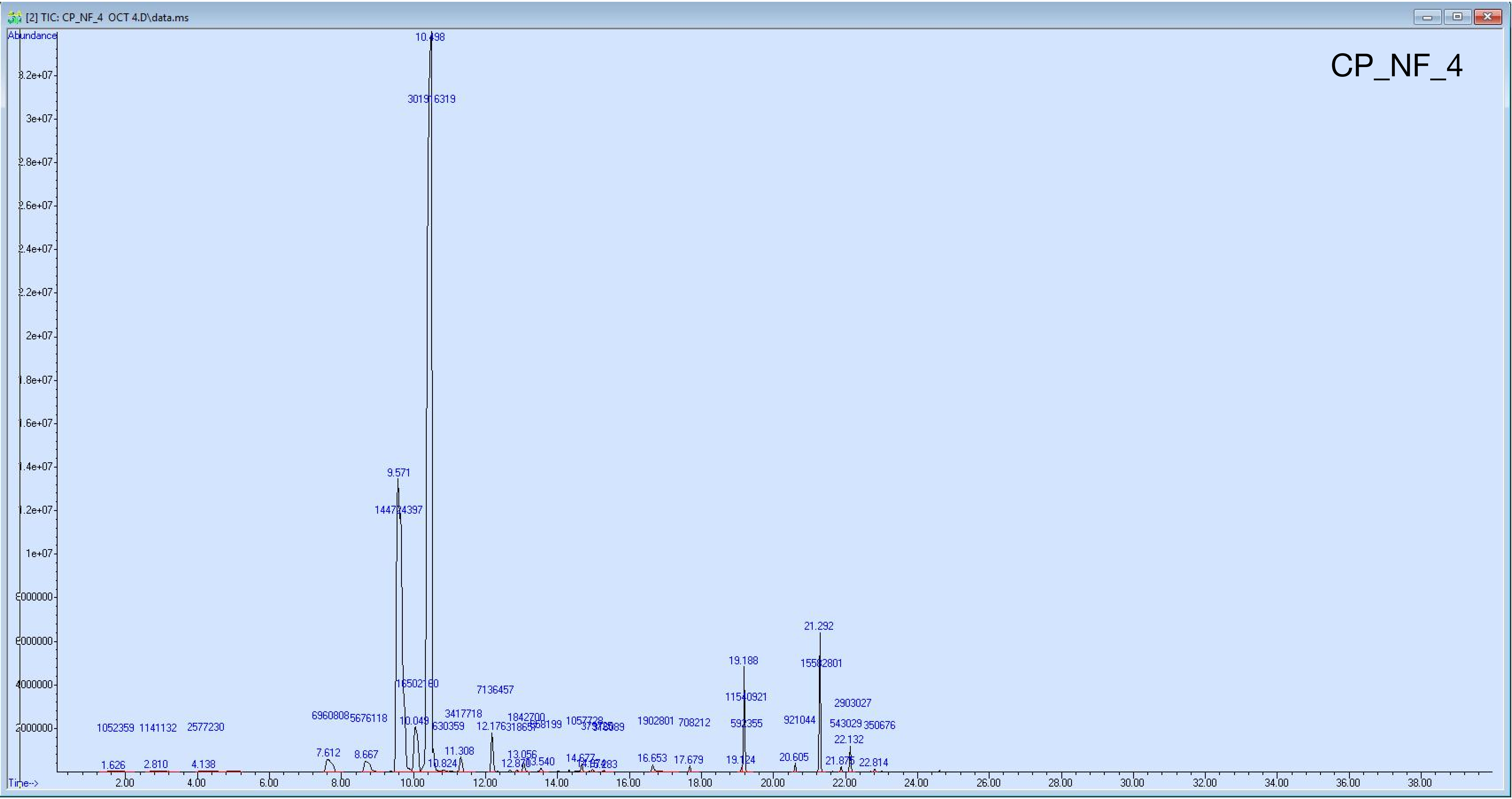
[2] TIC: CP_NF_4 OCT 4.D\data.ms
CP_NF_4
Abundance
Time-->
10.498
9.571
19.188
21.292

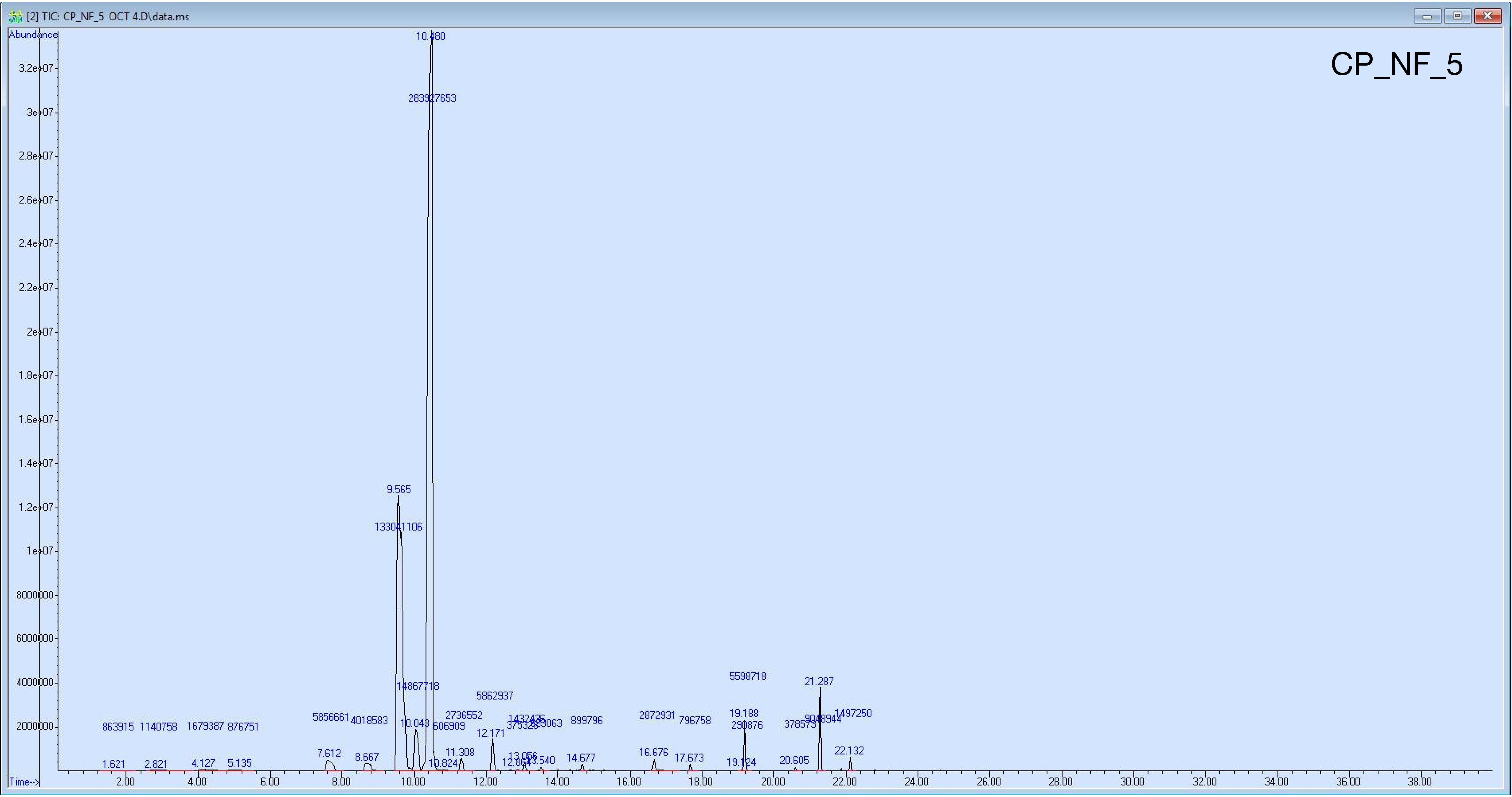
[2] TIC: CP_NF_5 OCT 4.D\data.ms
CP_NF_5
Abundance
Time-->
10.480
283927653
9.565
133041106
14867718
10.043
606909
2736552
5862937
12.171
11.308
10.824
7.612
5856661
8.667
4018583
1.621
863915
2.821
1140758
4.127
1679387
5.135
876751
899796
14.677
16.676
2872931
17.673
796758
5598718
19.188
290876
19.124
20.605
378573
21.287
1497250
22.132

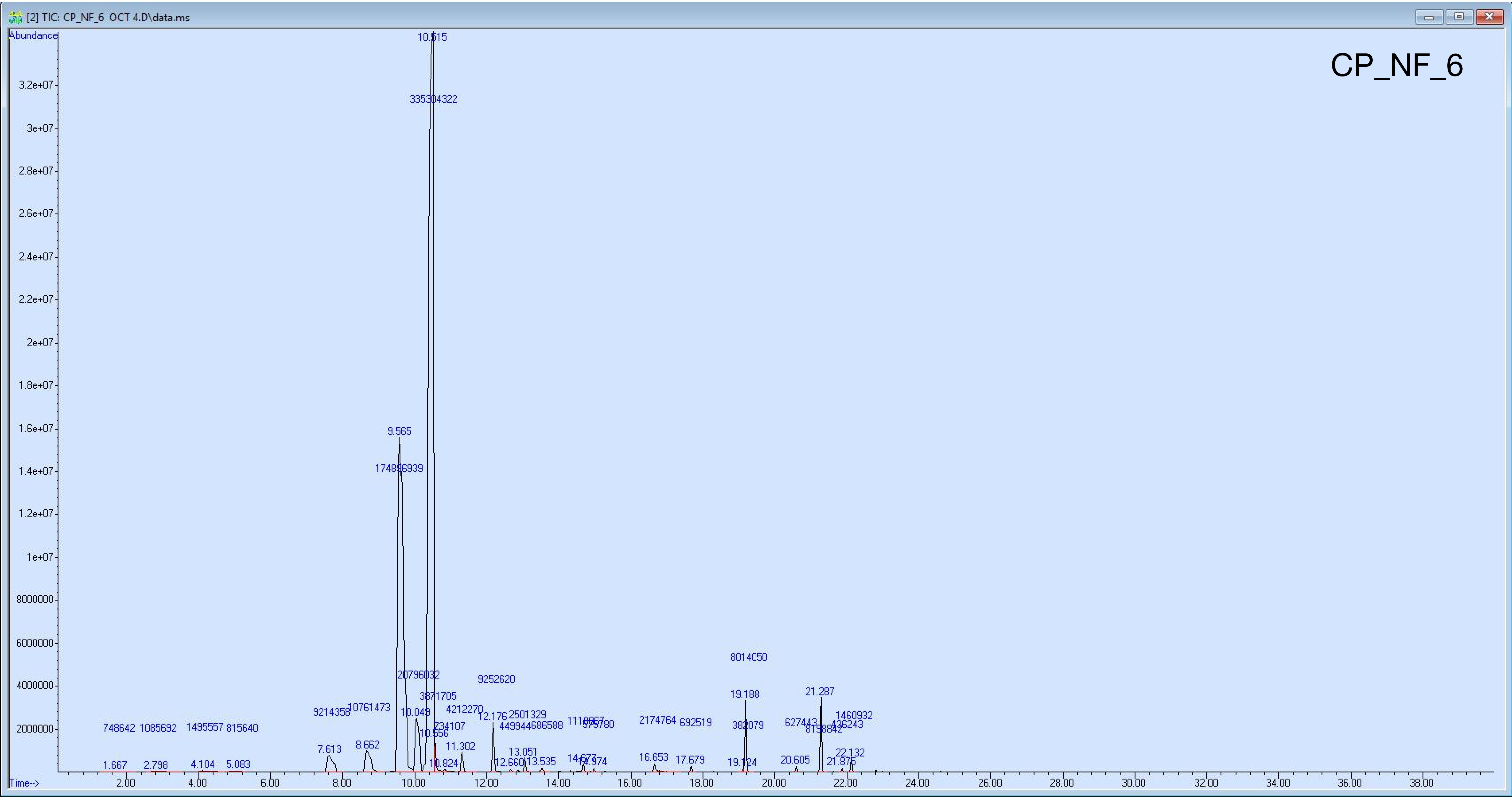
[2] TIC: CP_NF_6 OCT 4.D\data.ms
CP_NF_6
Abundance
Time-->
10.515
335304322
9.565
174856939
8014050
19.188
21.287
9252620
20796032
3871705
4212270

Cherokee Purple (heirloom)
No Flood
Spodptera exigua-damaged

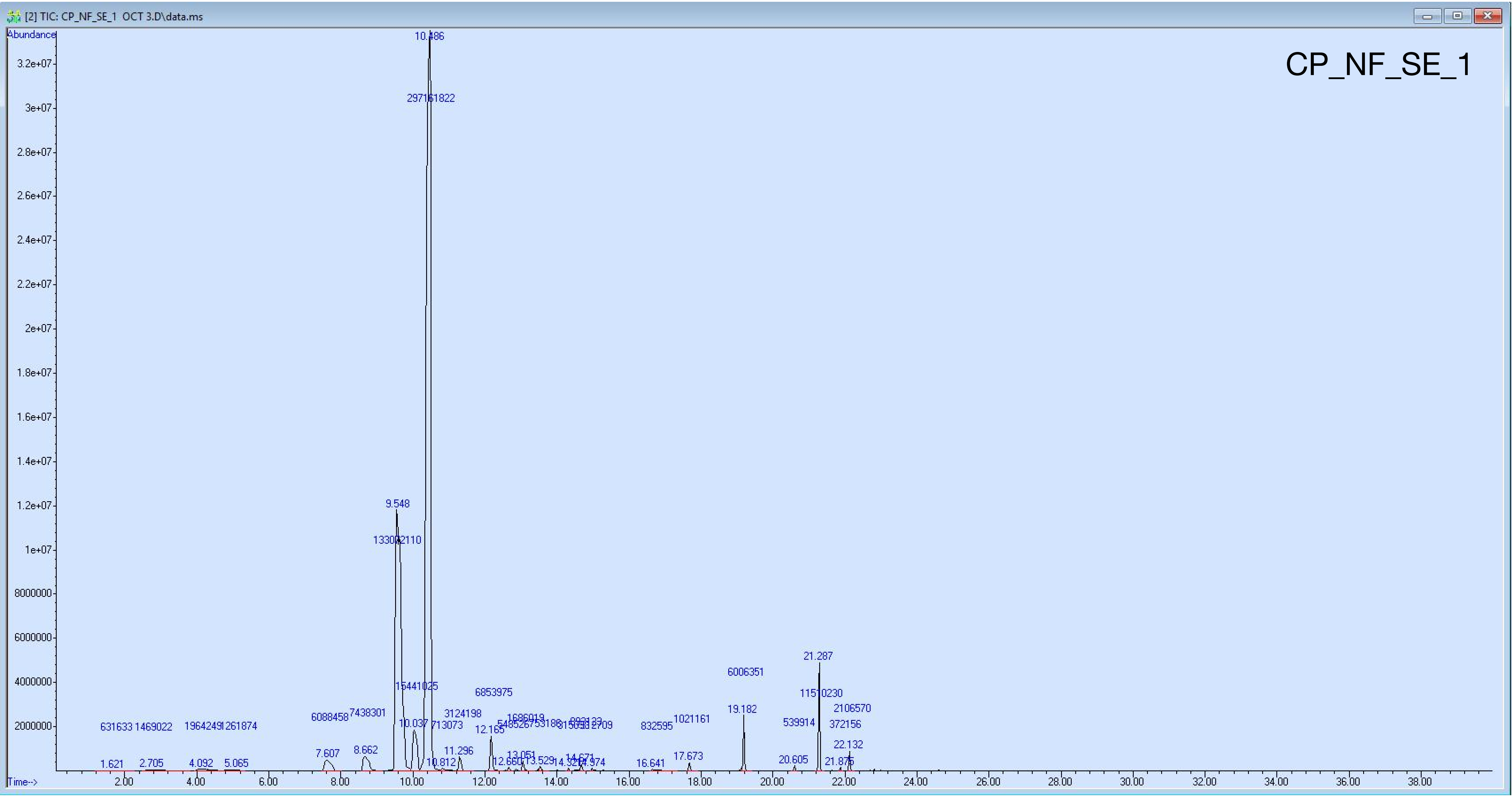
[2] TIC: CP_NF_SE_1 OCT 3.D\data.ms
CP_NF_SE_1
Abundance
Time-->
10.486
29716182
9.548
13302110
21.287
6006351
11510230
19.182
2106570
372156
22.132
539914
20.605
21.875
15441025
6853975
3124198
12.165
11.296
10.812
10.037
713073
6088458
7438301
7.607
8.662
631633
1469022
1964249
1261874
1.621
2.705
4.092
5.065
832595
1021161
16.641
17.673
13.051
13.529
14.974

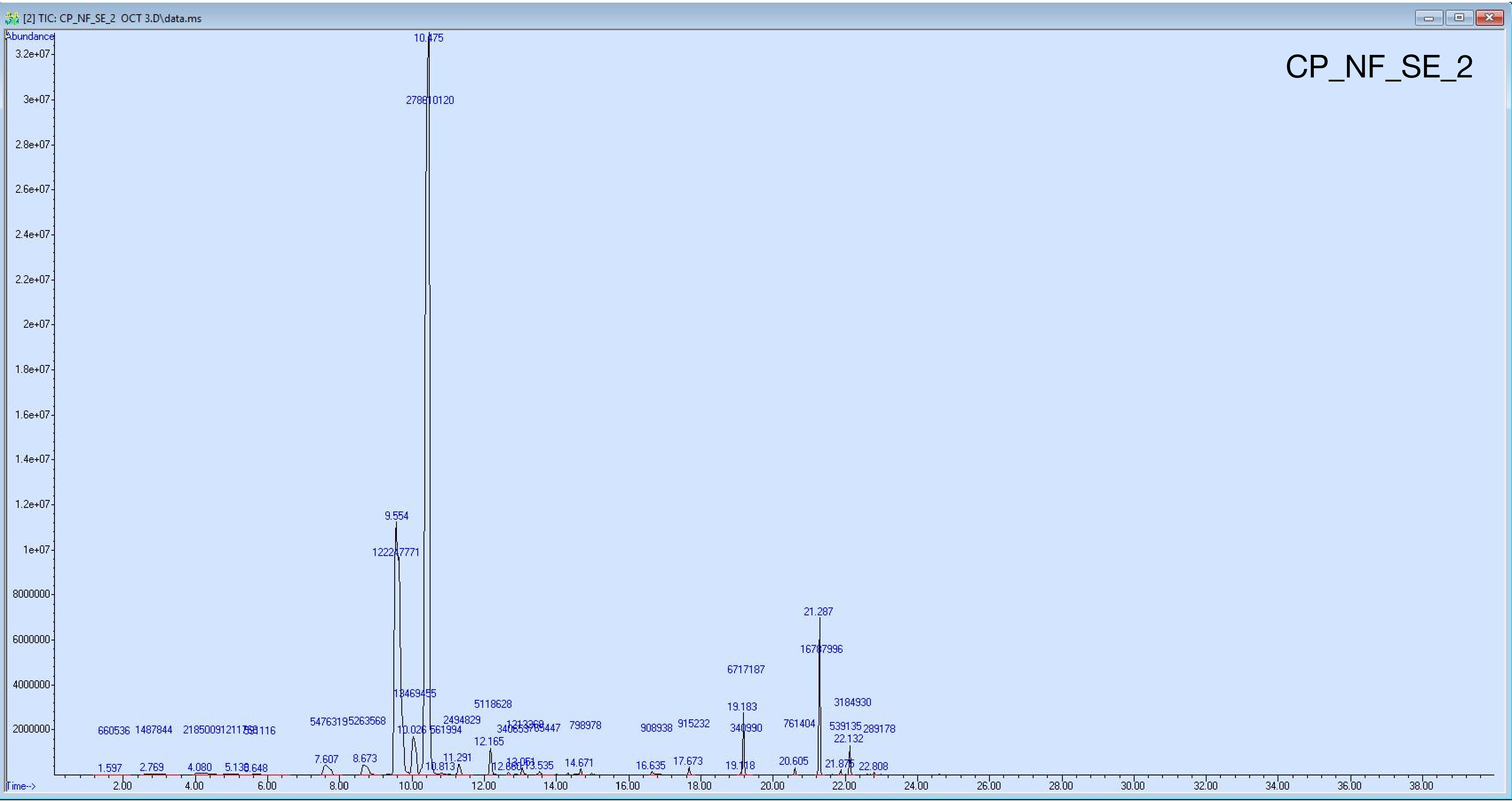
[2] TIC: CP_NF_SE_2 OCT 3.D\data.ms
CP_NF_SE_2
Abundance
Time-->
10.475
278810120
9.554
122247771
21.287
16787996
19.183
6717187

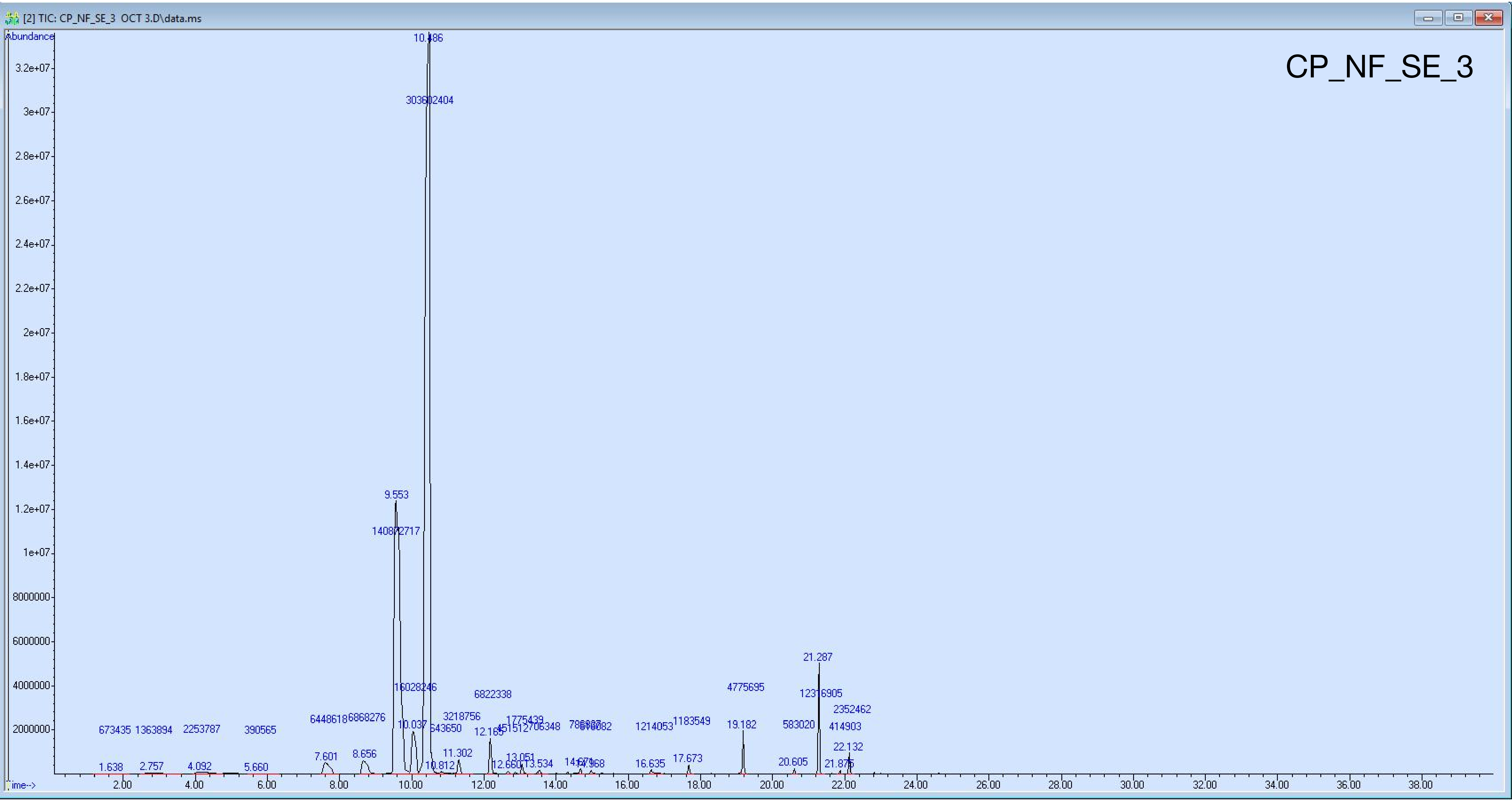
[2] TIC: CP_NF_SE_3 OCT 3.D\data.ms
CP_NF_SE_3
Abundance
Time-->
10.486
303602404
9.553
21.287
12316905
4775695
19.182
6822338
12.165
3218756
11.302
2352462
22.132

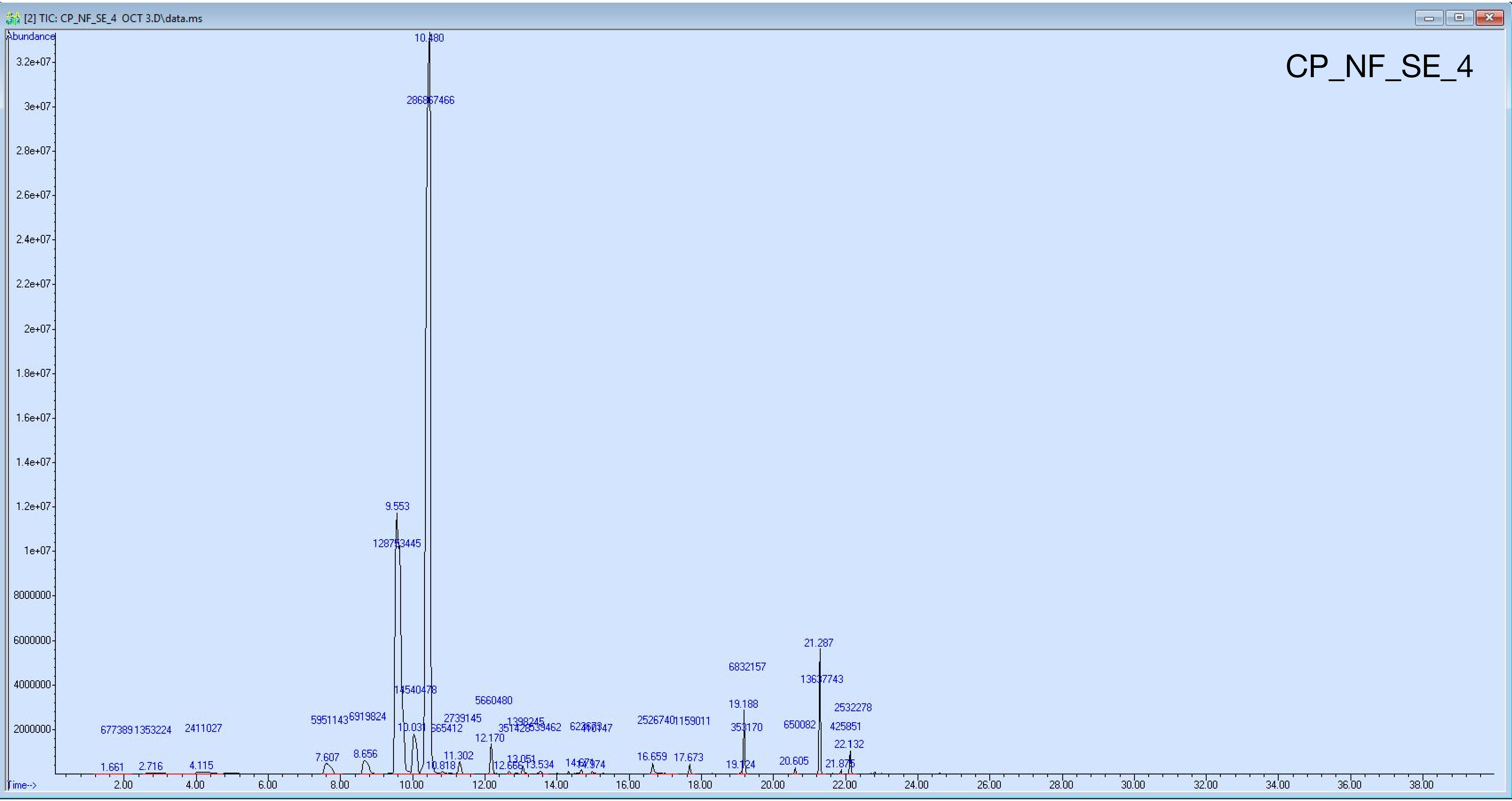
[2] TIC: CP_NF_SE_4 OCT 3.D\data.ms
CP_NF_SE_4
Abundance
Time-->
10.480
28667466
9.553
12753445
21.287
13637743
19.188
6832157
353170
5660480
12.170
14540478
10.031
2739145
11.302
22.132
2532278
425851
650082
20.605
21.875
19.124
16.659
2526740
17.673
1159011
7.607
5951143
8.656
6919824
1.661
2.716
4.115
2411027
10.818
13.534

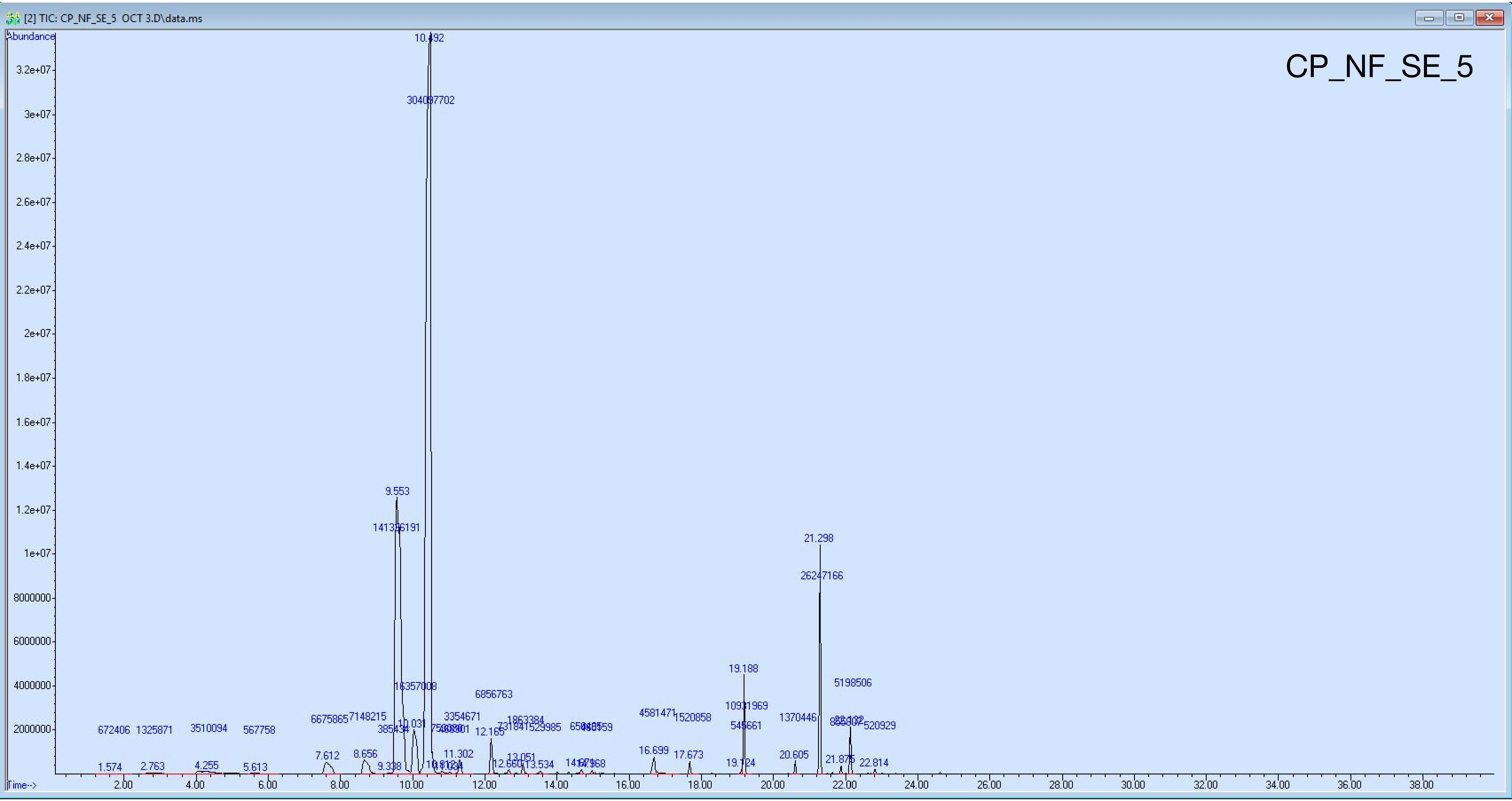

[2] TIC: CP_NF_SE_5 OCT 3.D\data.ms
CP_NF_SE_5
Abundance
Time-->
3.2e+07
3e+07
2.8e+07
2.6e+07
2.4e+07
2.2e+07
2e+07
1.8e+07
1.6e+07
1.4e+07
1.2e+07
1e+07
8000000
6000000
4000000
2000000
2.00
4.00
6.00
8.00
10.00
12.00
14.00
16.00
18.00
20.00
22.00
24.00
26.00
28.00
30.00
32.00
34.00
36.00
38.00
9.553
21.298
26247166
19.188
5198506
6856763
4581471
1520858
1370446
520929
672406
1325871
3510094
567758
6675865
7148215
3354671
1.574
2.763
4.255
5.613
7.612
8.656
10.031
11.302
12.165
13.051
13.534
16.699
17.673
20.605
22.814

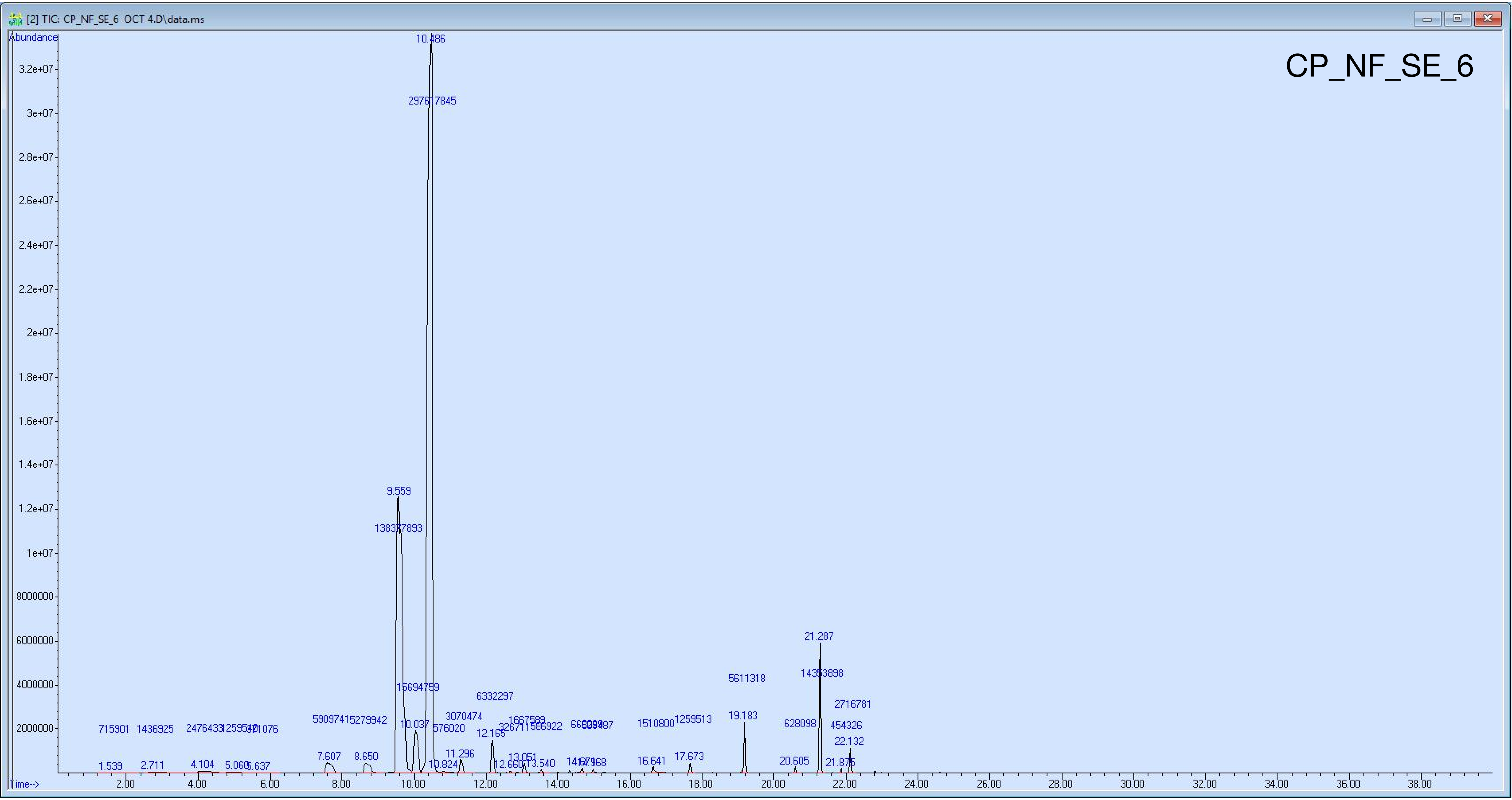

[2] TIC: CP_NF_SE_6 OCT 4.D\data.ms
CP_NF_SE_6
Abundance
Time-->
10.486
29767845
9.559
13837893
21.287
14353898
19.183
5611318
15694759
10.037
6332297
12.165
3070474
576020
11.296
10.824
2716781
454326
22.132
21.875
628098
20.605
1510800
16.641
1259513
17.673
715901
1.539
1436925
2.711
2476433
4.104
5.060
5.637
7.607
8.650
13.051
12.660
13.540

Cherokee Purple (heirloom)
No Flood
Manduca sexta-damaged

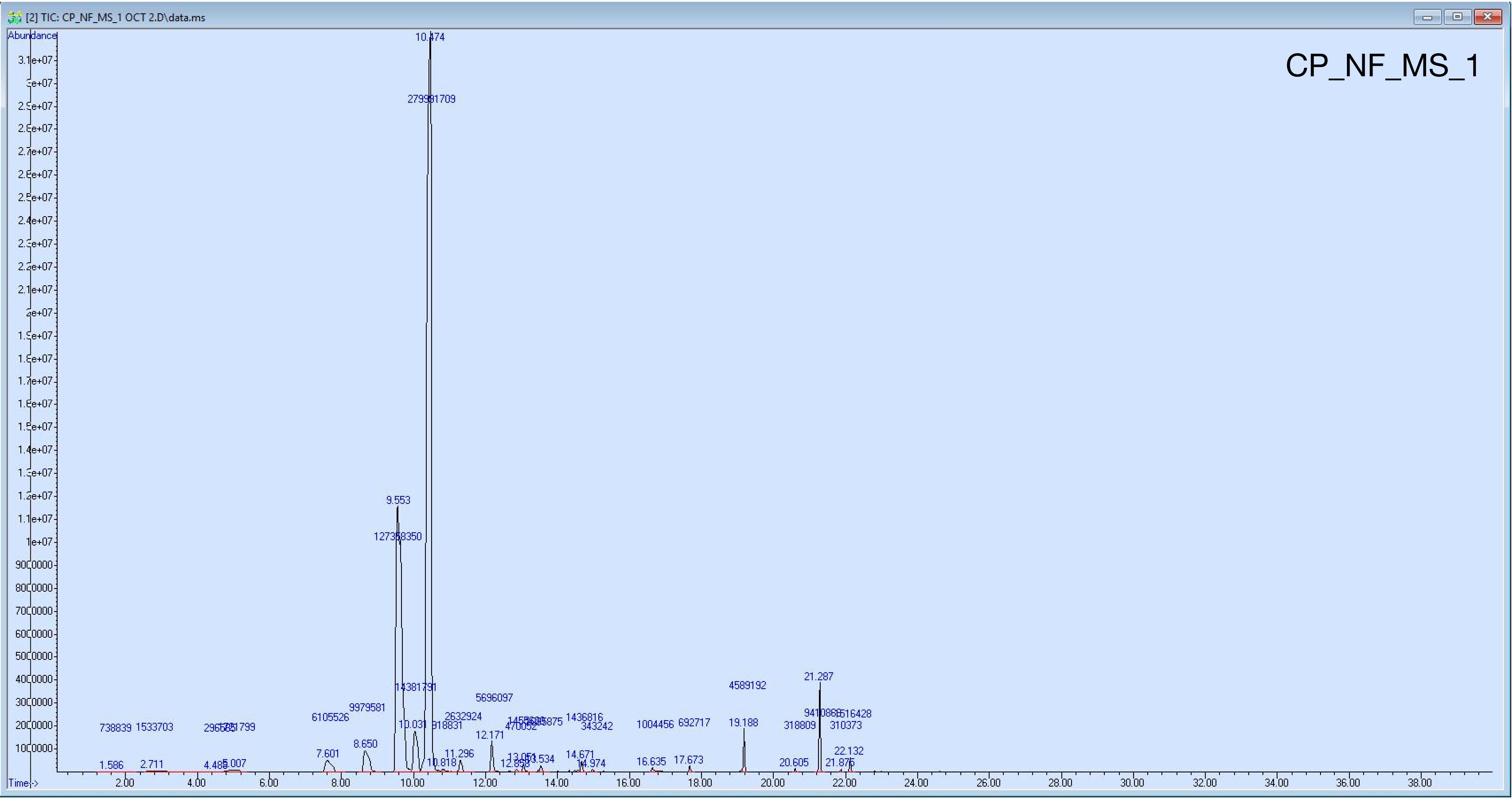
[2] TIC: CP_NF_MS_1 OCT 2.D\data.ms
CP_NF_MS_1
Abundance
Time-->
10.474
279981709
9.553
127358350
21.287
4589192
19.188
5696097
12.171
6105526
7.601
9979581
8.650
10.031
2632924
11.296
1436816
14.671
1004456
16.635
692717
17.673
22.132
1.586
2.711
738839
1533703

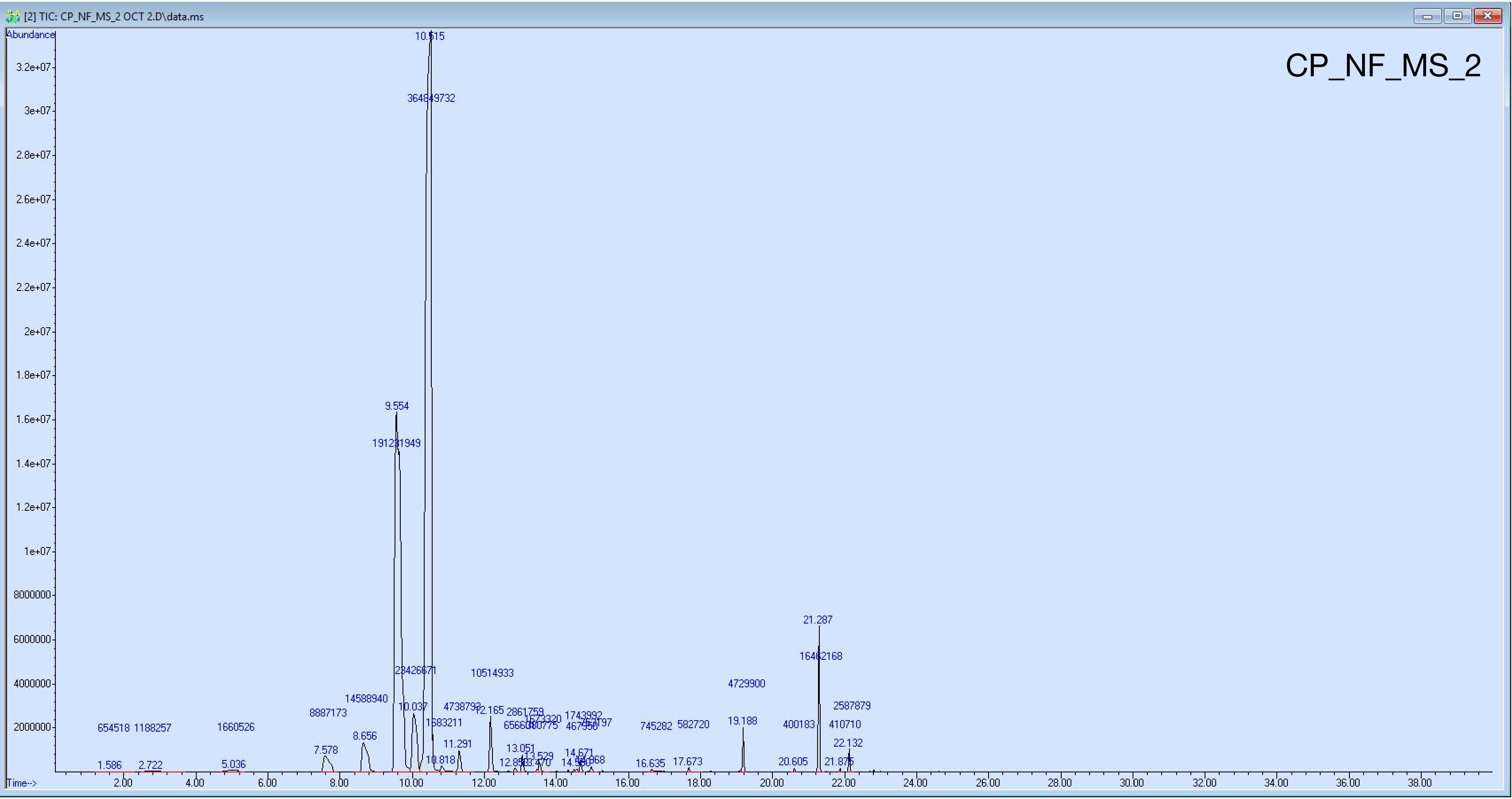

[2] TIC: CP_NF_MS_2 OCT 2.D\data.ms
CP_NF_MS_2
Abundance
Time-->
10.515
364849732
9.554
191231949
21.287
16462168

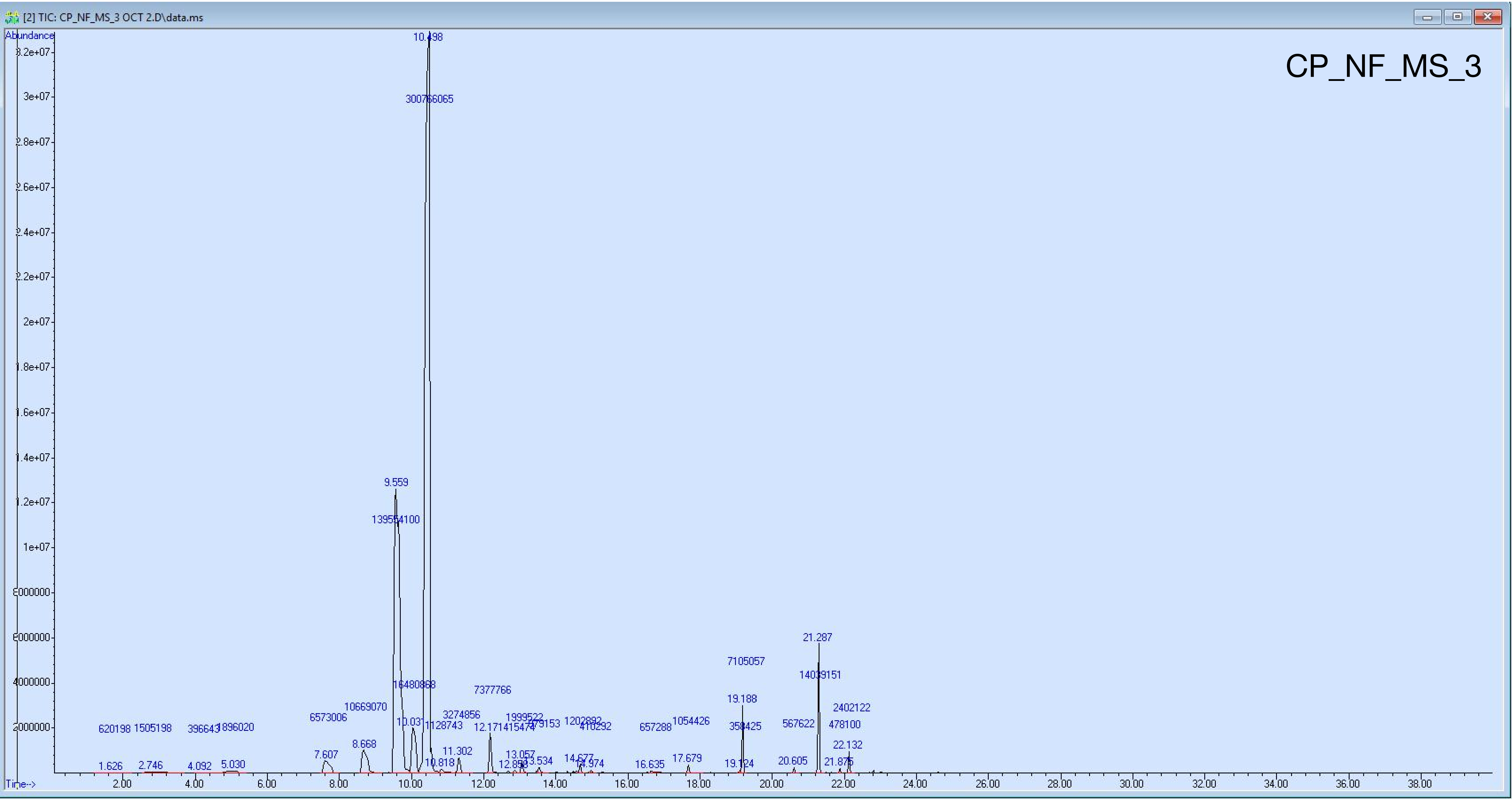
[2] TIC: CP_NF_MS_3 OCT 2.D\data.ms
CP_NF_MS_3
Abundance
3.2e+07
3e+07
2.8e+07
2.6e+07
2.4e+07
2.2e+07
2e+07
1.8e+07
1.6e+07
1.4e+07
1.2e+07
1e+07
4000000
2000000
Time-->
2.00
4.00
6.00
8.00
10.00
12.00
14.00
16.00
18.00
20.00
22.00
24.00
26.00
28.00
30.00
32.00
34.00
36.00
38.00
10.498
9.559
21.287
7105057
19.188
7377766
3274856
10669070
6573006
2402122
22.132
620198
1505198
1896020
657288
1054426
567622
478100
1128743
1.626
2.746
4.092
5.030
7.607
8.668
11.302
10.818
13.057
13.534
14.677
16.635
17.679
19.124
20.605

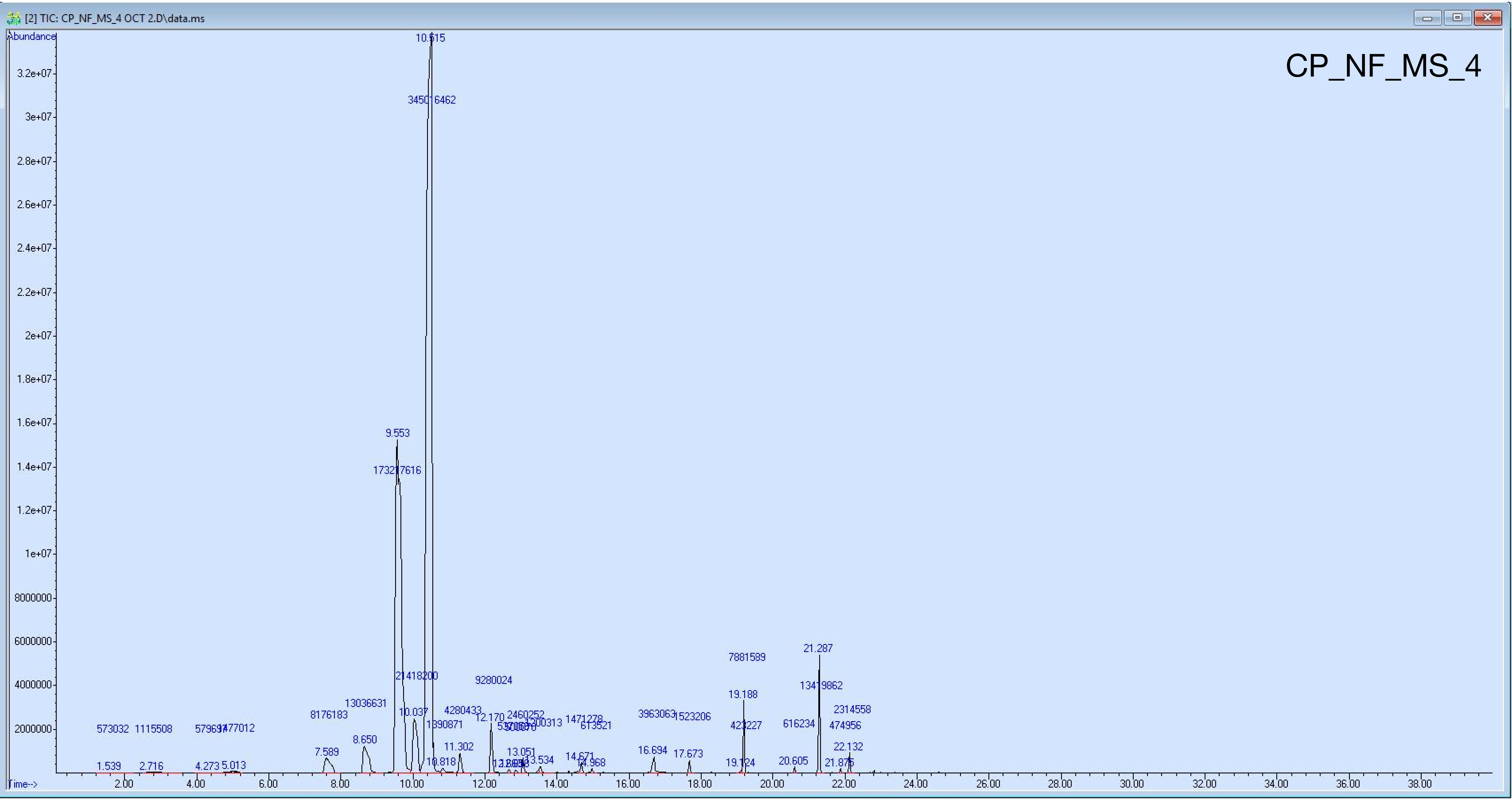
[2] TIC: CP_NF_MS_4 OCT 2.D\data.ms
CP_NF_MS_4
Abundance
Time-->
10.515
345016462
9.553
173217616
21.287
13419862
19.188
7881589

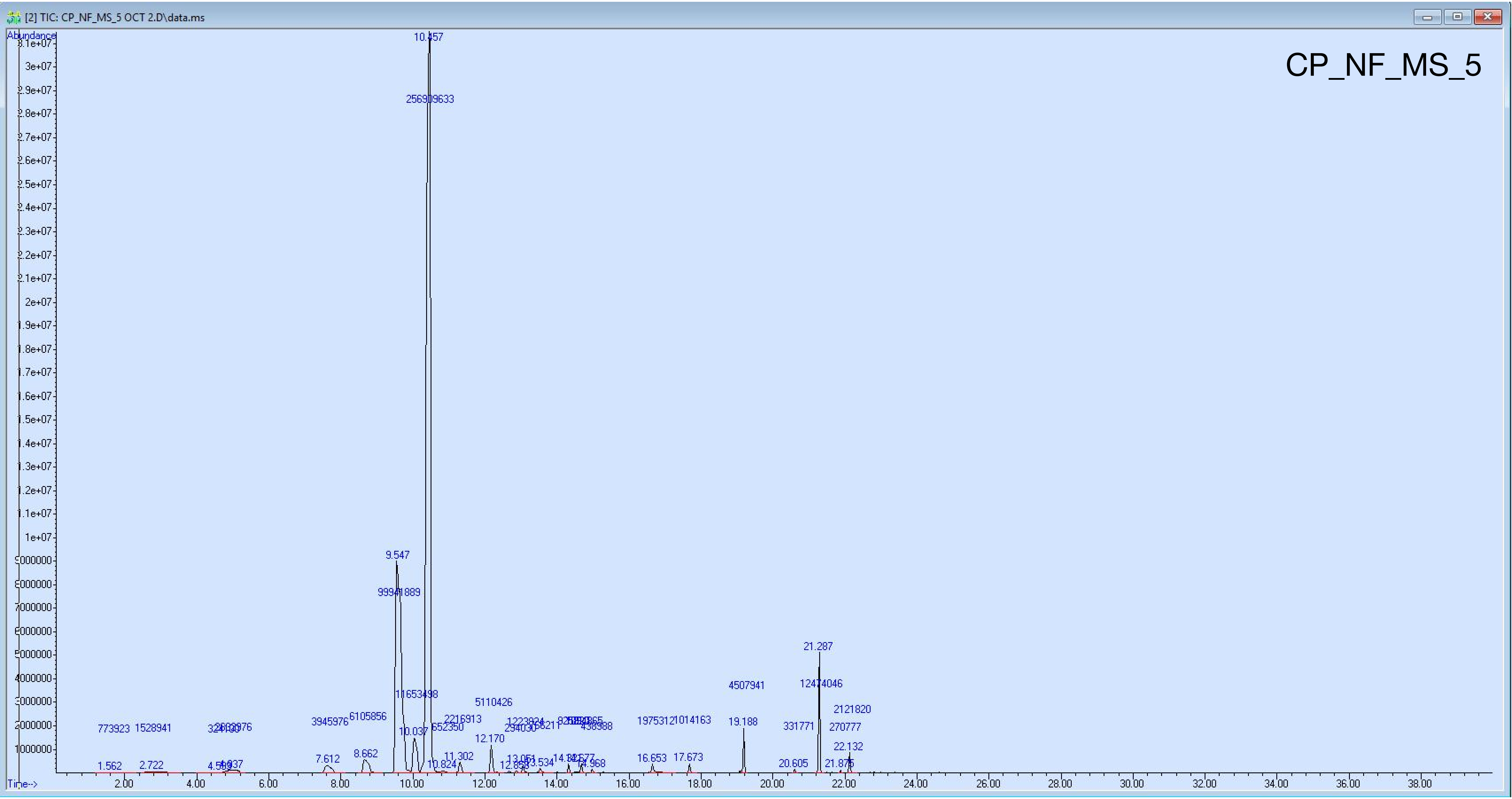

[2] TIC: CP_NF_MS_5 OCT 2.D\data.ms
CP_NF_MS_5
Abundance
Time-->
10.457
25699633
9.547
99941889
21.287
12474046
19.188
4507941

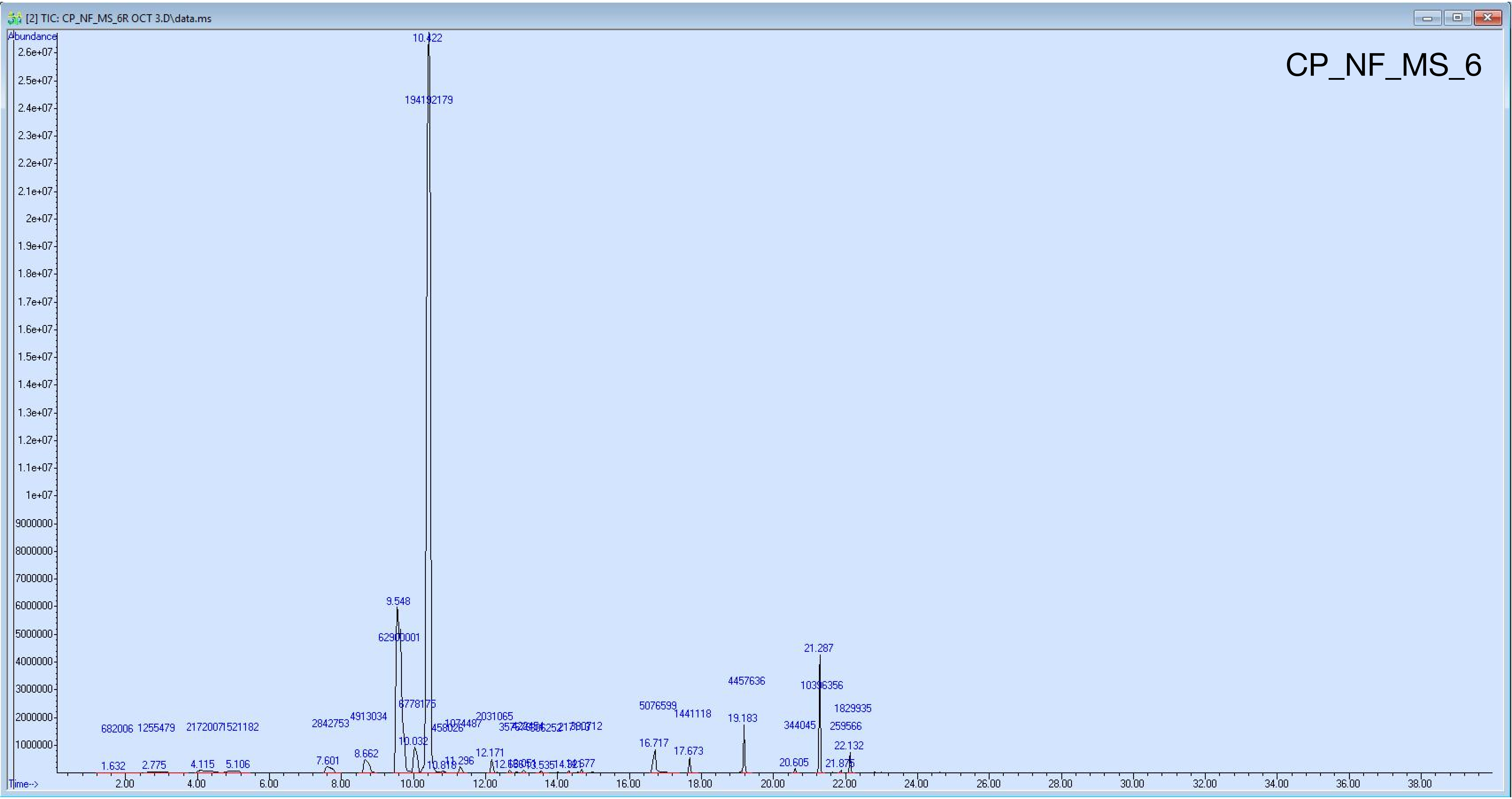

[2] TIC: CP_NF_MS_6R OCT 3.D\data.ms
CP_NF_MS_6
Abundance
Time-->
10.422
194192179
9.548
62900001
21.287
10396356
19.183
4457636
16.717
5076599
17.673
1441118
22.132
1829935

Cherokee Purple (heirloom)
Flooding
No Herbivory

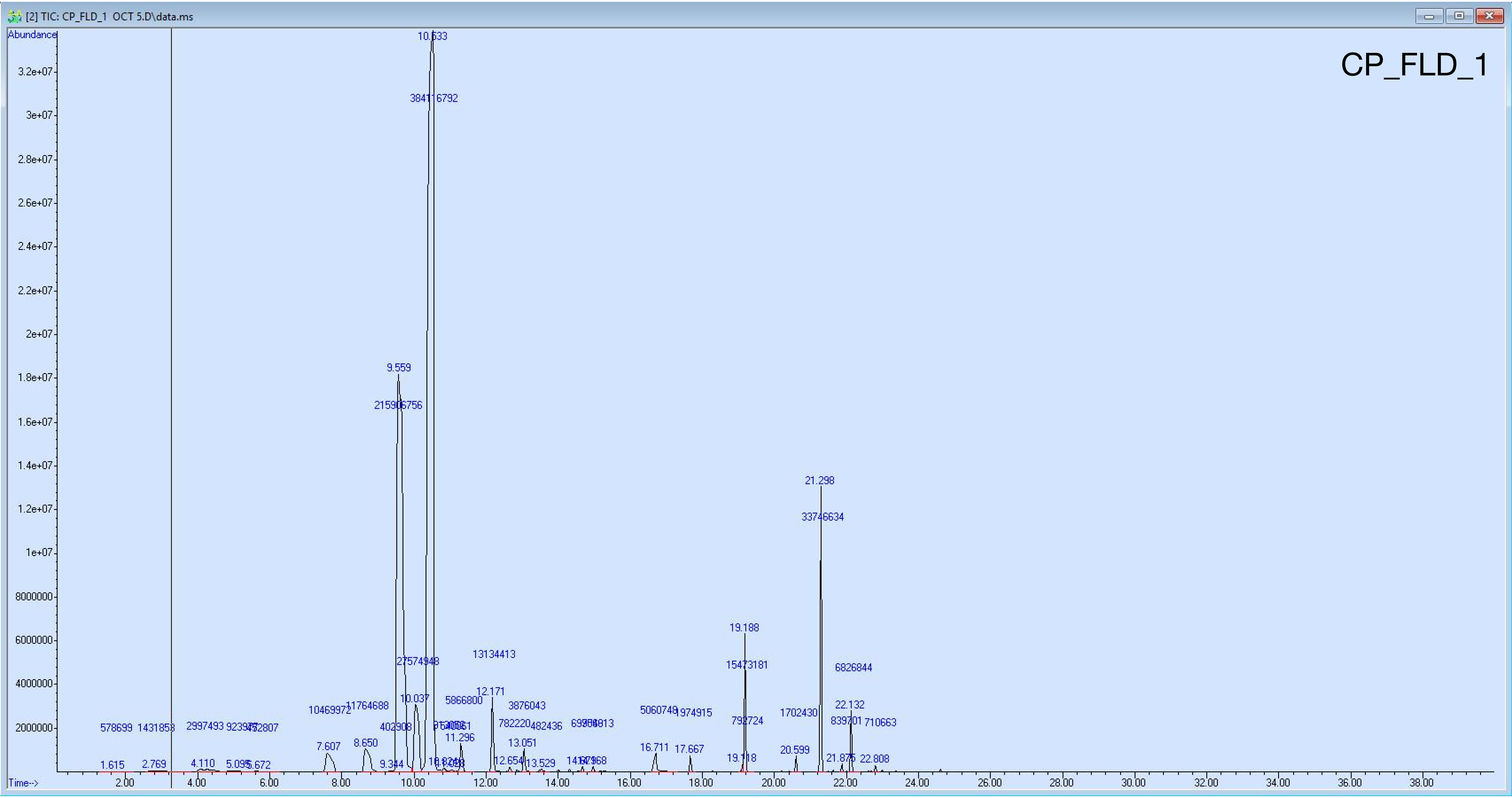

[2] TIC: CP_FLD_1 OCT 5.D\data.ms
CP_FLD_1
Abundance
3.2e+07
3e+07
2.8e+07
2.6e+07
2.4e+07
2.2e+07
2e+07
1.8e+07
1.6e+07
1.4e+07
1.2e+07
1e+07
8000000
6000000
4000000
2000000
Time-->
2.00
4.00
6.00
8.00
10.00
12.00
14.00
16.00
18.00
20.00
22.00
24.00
26.00
28.00
30.00
32.00
34.00
36.00
38.00
10.533
9.559
21.298
33746634
19.188
15473181
6826844
13134413
12.171
5866800
3876043
10.037
22.132
11.296
13.051
16.711
17.667
20.599
7.607
8.650
9.344
1.615
2.769
4.110
5.672
12.654
13.529
19.118
22.808
578699
1431853
2997493
10469972
11764688
782220
482436
5060748
1974915
792724
1702430
710663

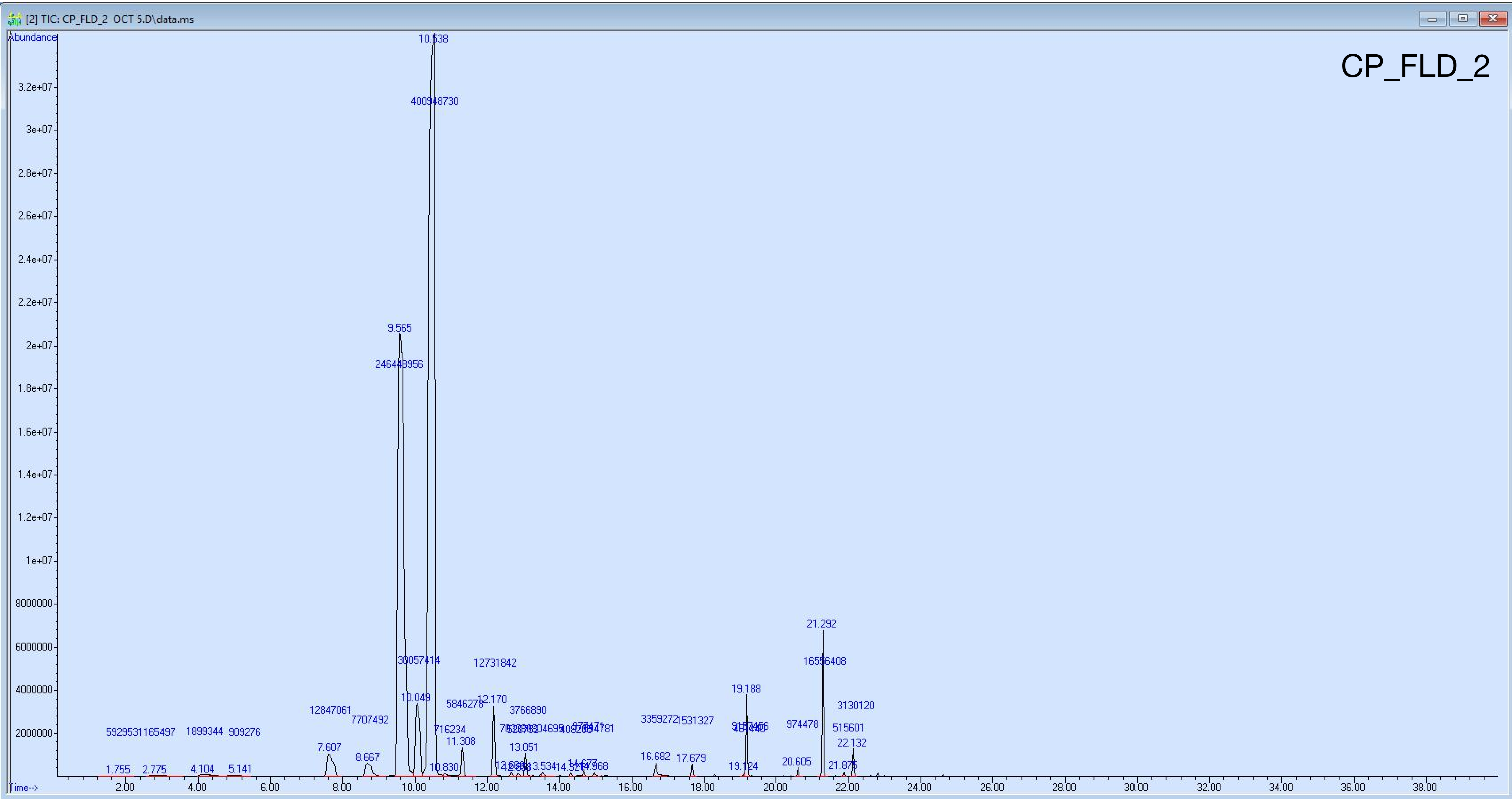
[2] TIC: CP_FLD_2 OCT 5.D\data.ms
CP_FLD_2
Abundance
Time-->
10.538
400948730
9.565
246443956
30057414
10.049
12731842
5846278
12.170
3766890
11.308
716234
10.830
12847061
7.607
7707492
8.667
13.051
16.682
3359272
17.679
1531327
19.188
19.124
21.292
16556408
974478
20.605
3130120
515601
22.132
1.755
2.775
4.104
5.141
1899344
909276

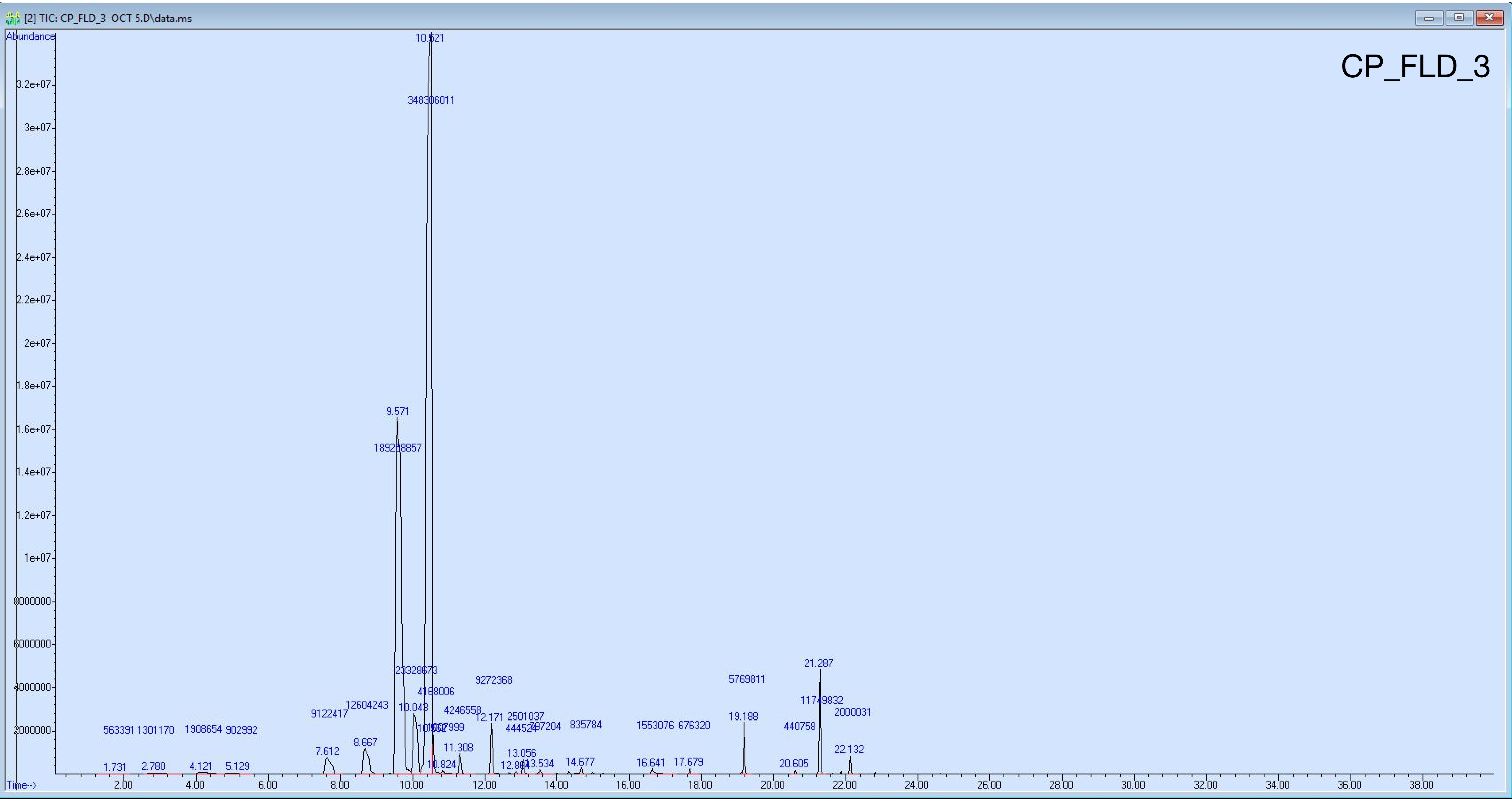
[2] TIC: CP_FLD_3 OCT 5.D\data.ms
CP_FLD_3
Abundance
Time-->
10.521
348306011
9.571
189258857
23328673
4168006
9272368
5769811
21.287
11749832
2000031
12604243
9122417
10.043
4246558
12.171
2501037
797204
835784
1553076
676320
19.188
440758
563391
1301170
1908654
902992
8.667
7.612
11.308
13.056
22.132
1.731
2.780
4.121
5.129
10.824
13.534
14.677
16.641
17.679
20.605

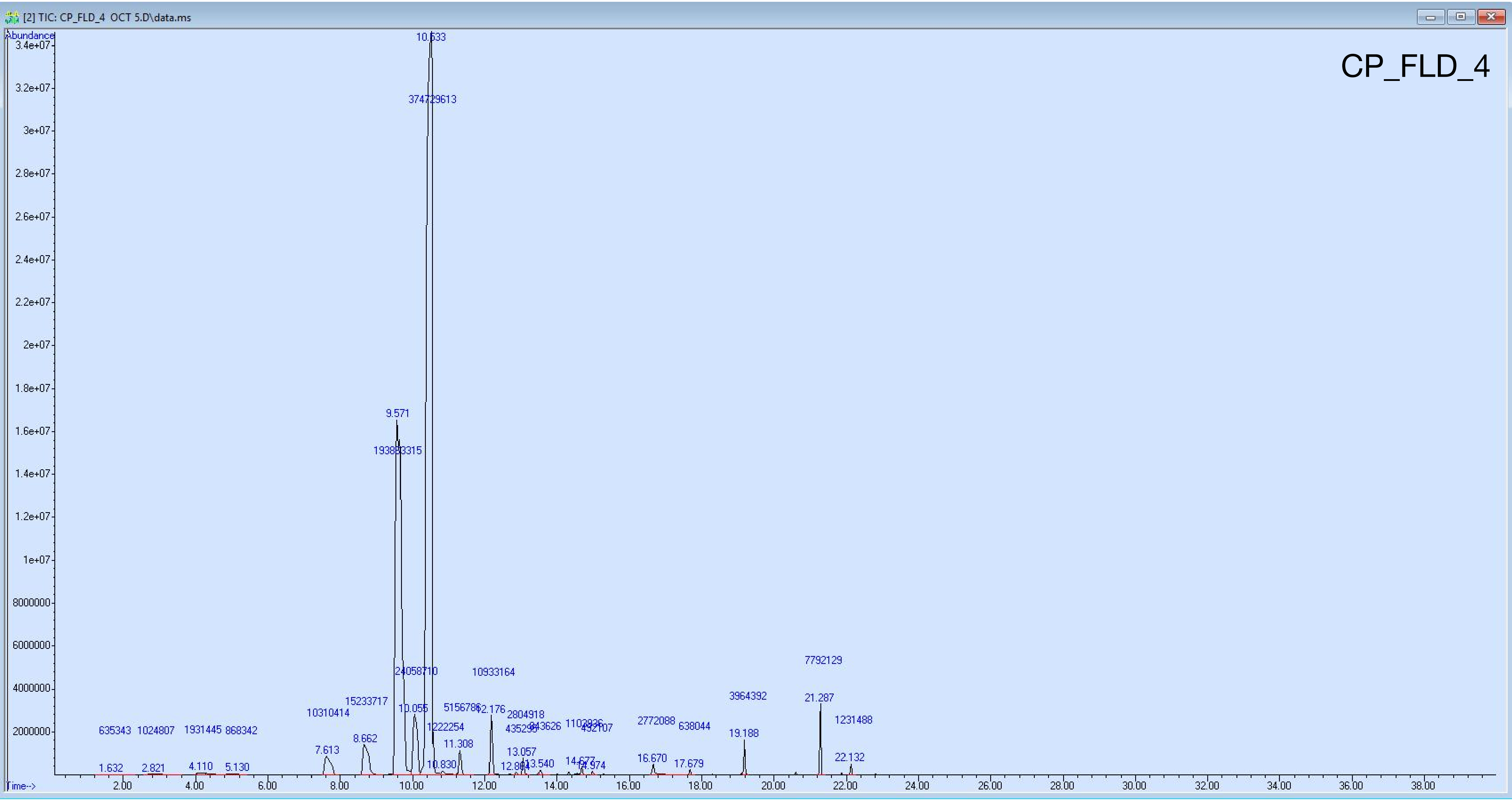

[2] TIC: CP_FLD_4 OCT 5.D\data.ms
CP_FLD_4
Abundance
Time-->
10.533
374729613
9.571
24058710
10933164
7792129
3964392
21.287
19.188
1231488
22.132
15233717
10310414
8.662
7.613
10.055
5156786
1222254
11.308
10.830
2804918
13.057
13.540
2772088
638044
16.670
17.679
635343
1024807
1931445
868342
1.632
2.821
4.110
5.130

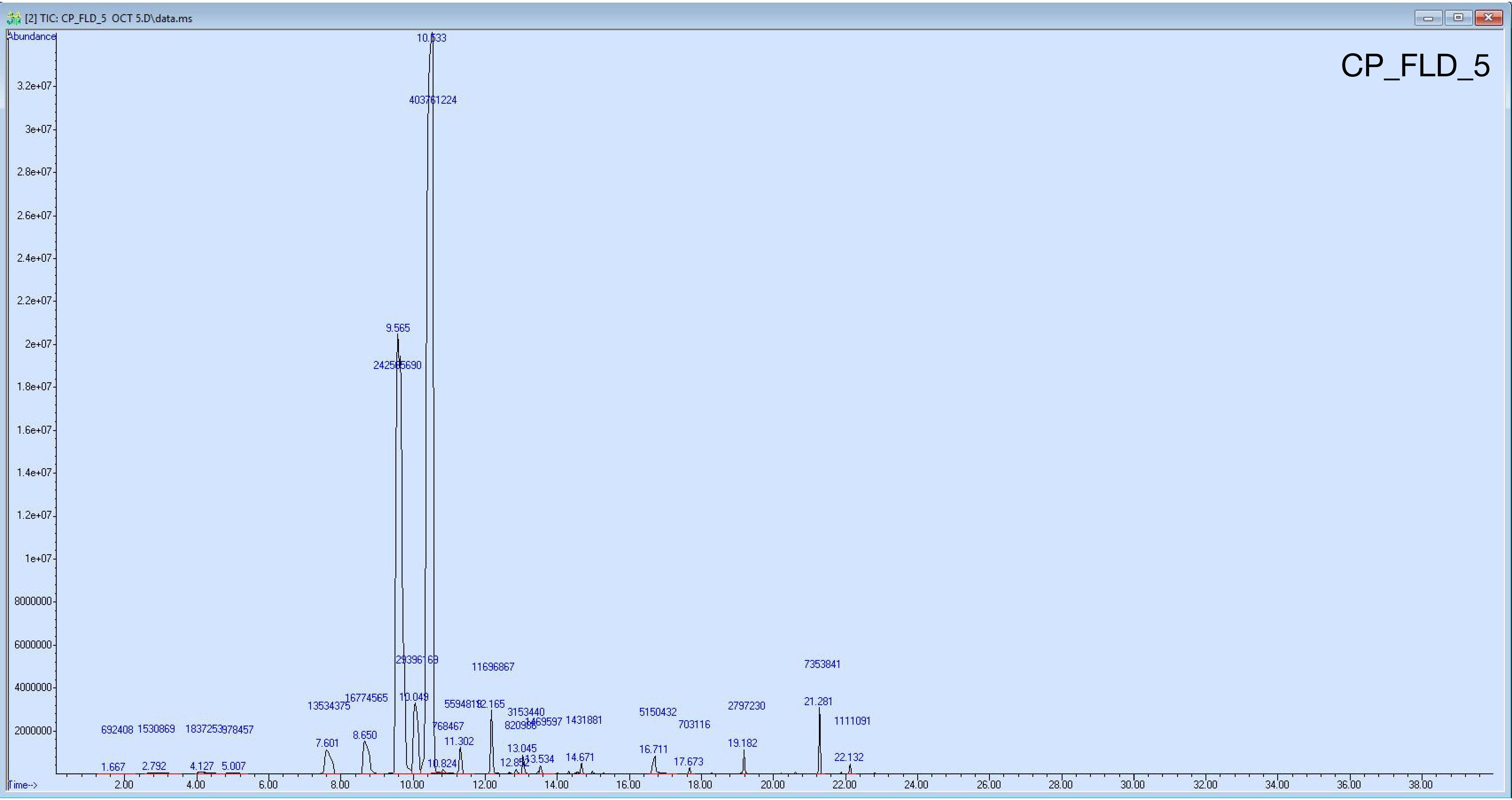

[2] TIC: CP_FLD_5 OCT 5.D\data.ms
CP_FLD_5
Abundance
3.2e+07
3e+07
2.8e+07
2.6e+07
2.4e+07
2.2e+07
2e+07
1.8e+07
1.6e+07
1.4e+07
1.2e+07
1e+07
8000000
6000000
4000000
2000000
Time-->
2.00
4.00
6.00
8.00
10.00
12.00
14.00
16.00
18.00
20.00
22.00
24.00
26.00
28.00
30.00
32.00
34.00
36.00
38.00
403761224
9.565
11696867
7353841
13534375
16774565
10.049
3153440
1431881
5150432
2797230
21.281
692408
1530869
978457
768467
703116
1111091
7.601
8.650
11.302
13.045
13.534
14.671
16.711
17.673
19.182
1.667
2.792
4.127
5.007
10.824
22.132

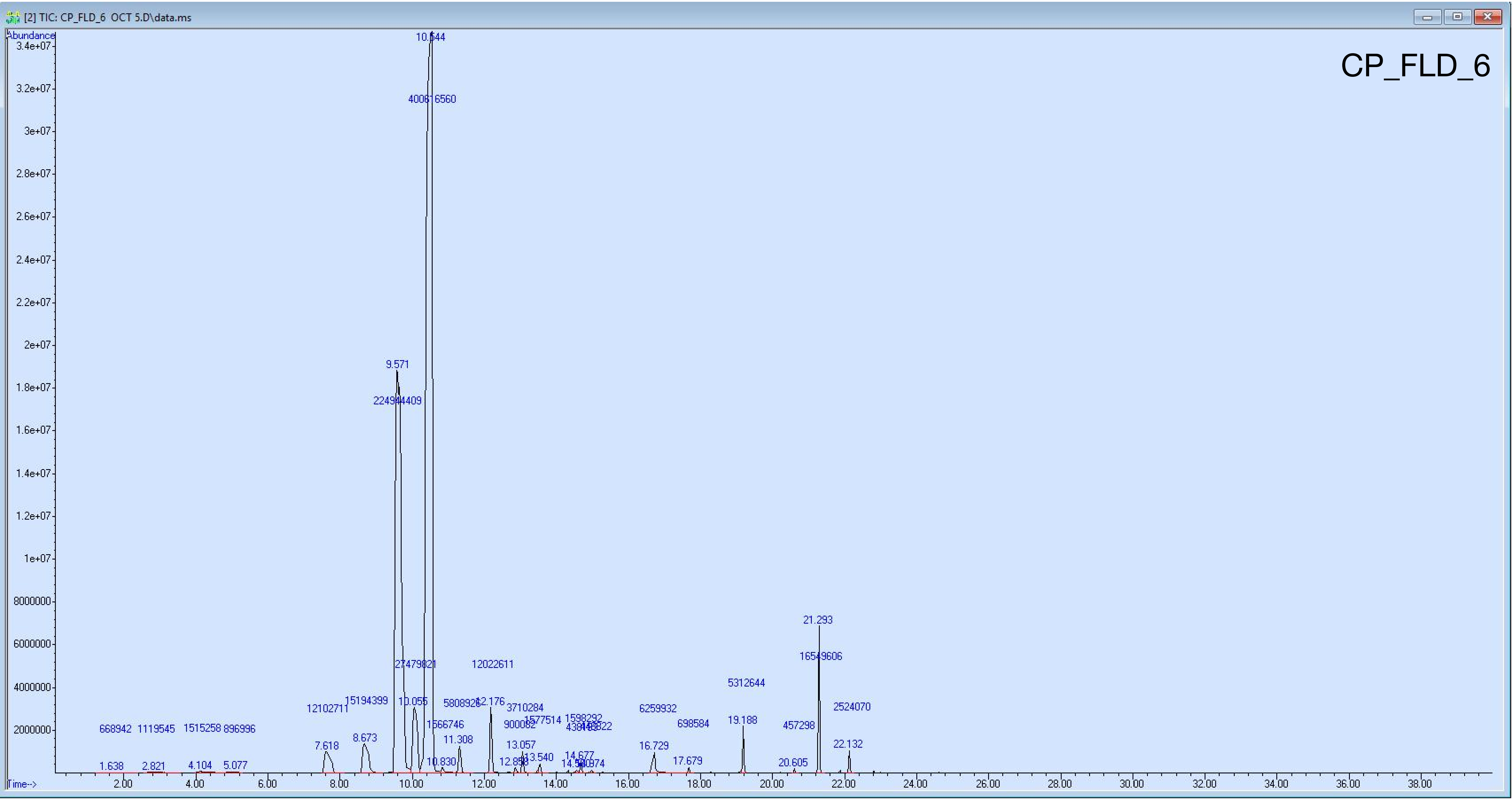

[2] TIC: CP_FLD_6 OCT 5.D\data.ms
CP_FLD_6
Abundance
Time-->
10.544
9.571
21.293
16549606
12022611
5312644
2524070
6259932
698584
457298
19.188
16.729
17.679
20.605
22.132
12.176
11.308
10.830
10.055
8.673
7.618
1.638
2.821
4.104
5.077
668942
1119545
1515258
896996
12102711
15194399
1566746
3710284
1577514
13.057
13.540
14.677

Cherokee Purple (heirloom)
Flooding
Spodptera exigua-damaged

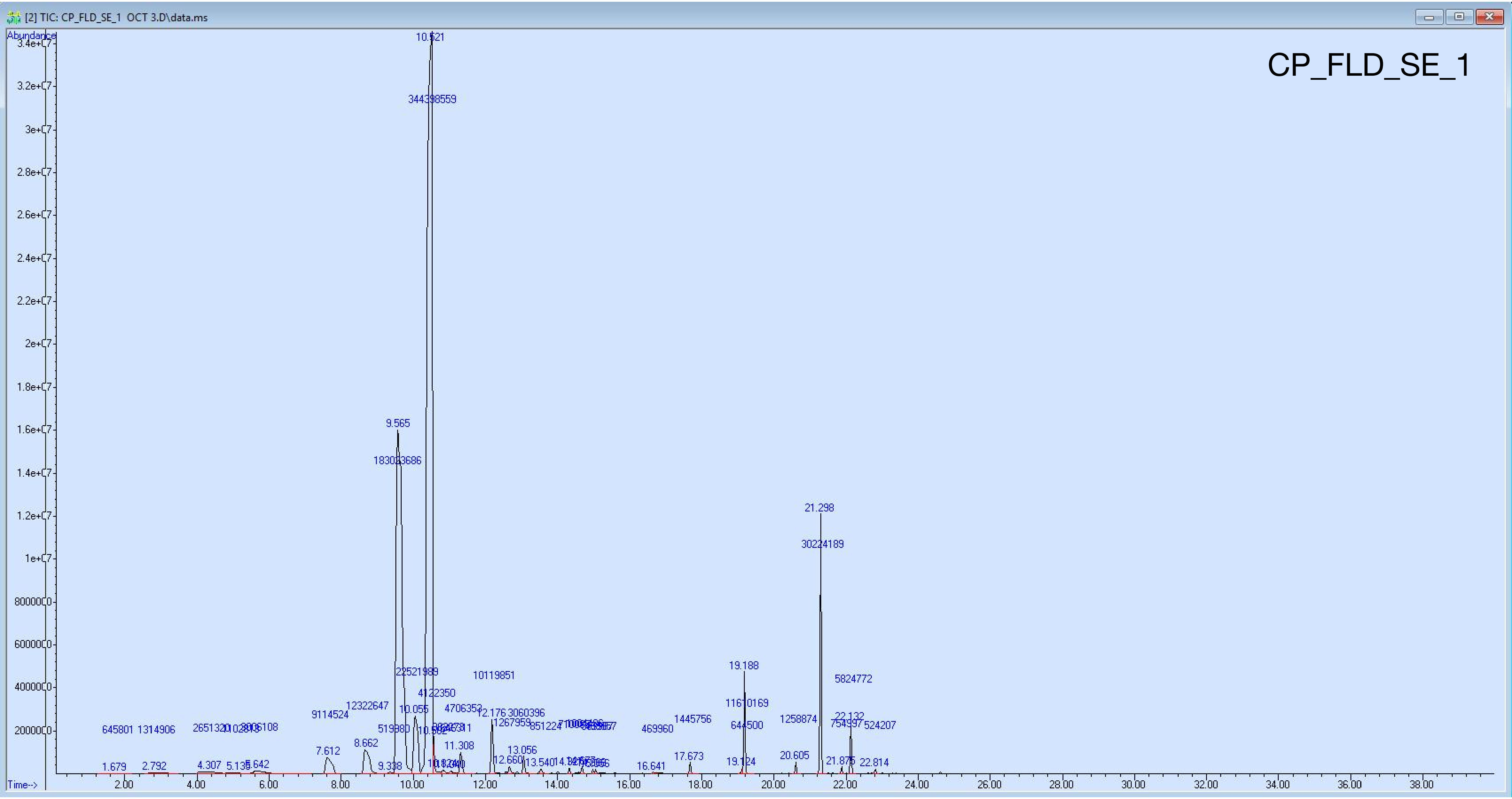
[2] TIC: CP_FLD_SE_1 OCT 3.D\data.ms
CP_FLD_SE_1
Abundance
Time-->
10.521
344398559
9.565
183023686
21.298
30224189
19.188
11610169

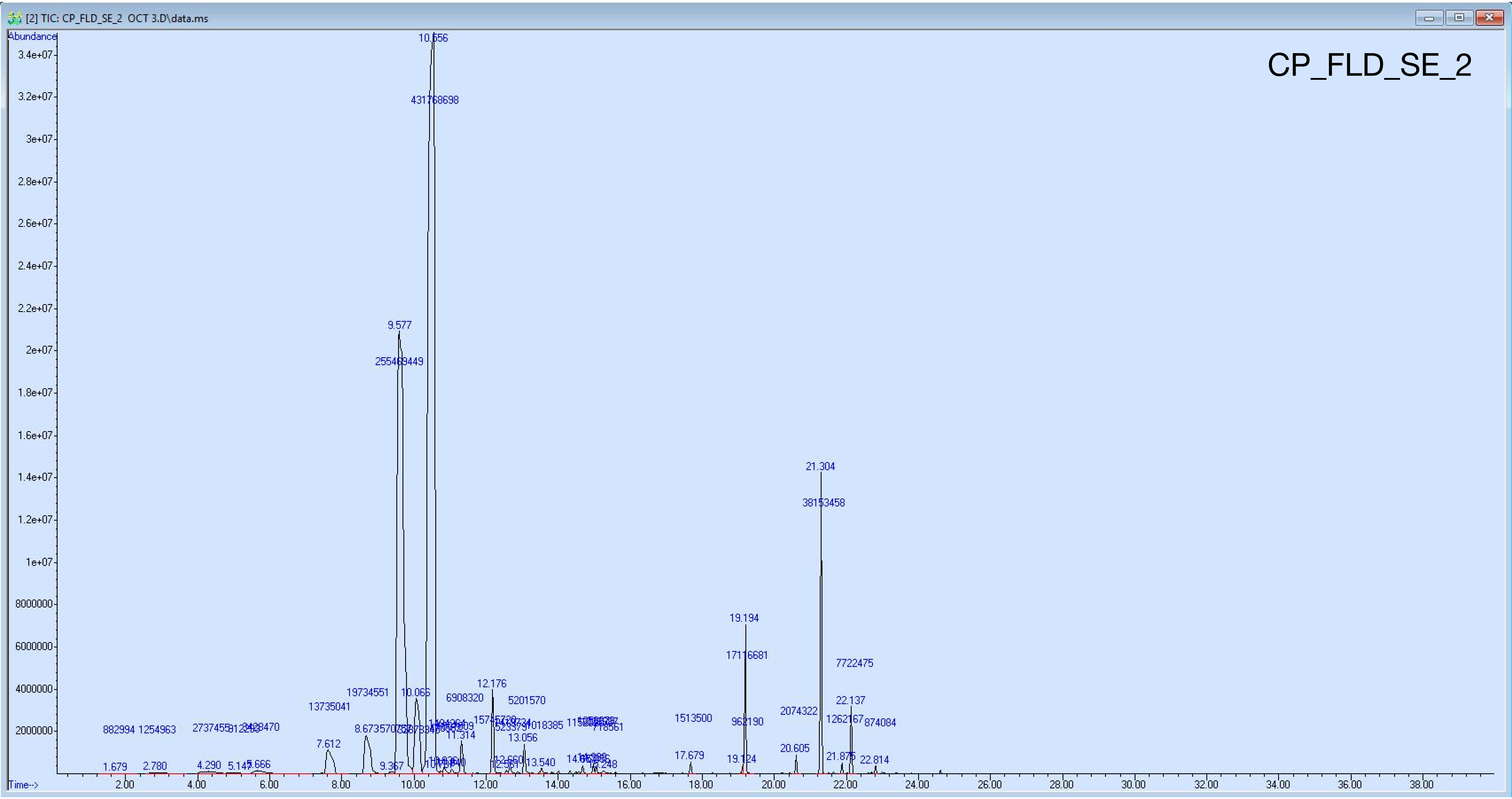
[2] TIC: CP_FLD_SE_2 OCT 3.D\data.ms
CP_FLD_SE_2
Abundance
Time-->
10.556
431768698
9.577
255469449
21.304
38153458
19.194
17116681
7722475
22.137
12.176
5201570
6908320
19734551
10.066
13735041
1513500
962190
2074322
1262167
874084
13.056
11.314
7.612
17.679
19.124
20.605
21.875
22.814
13.540
1.679
2.780
4.290
5.147
5.666
9.367
882994
1254963
2737455
3428470

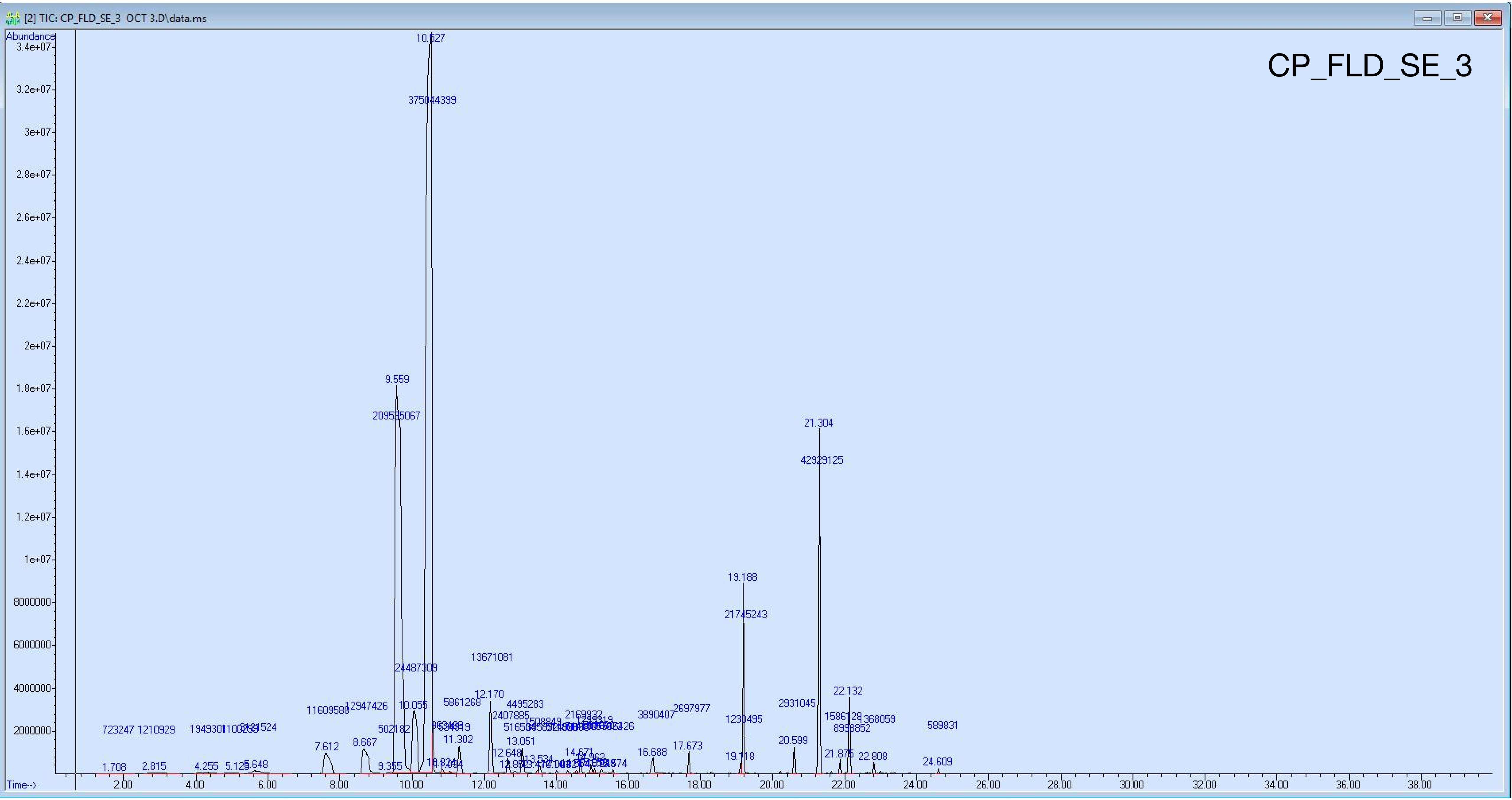
[2] TIC: CP_FLD_SE_3 OCT 3.D\data.ms
CP_FLD_SE_3
Abundance
3.4e+07
3.2e+07
3e+07
2.8e+07
2.6e+07
2.4e+07
2.2e+07
2e+07
1.8e+07
1.6e+07
1.4e+07
1.2e+07
1e+07
8000000
6000000
4000000
2000000
Time-->
2.00
4.00
6.00
8.00
10.00
12.00
14.00
16.00
18.00
20.00
22.00
24.00
26.00
28.00
30.00
32.00
34.00
36.00
38.00
10.627
375044399
9.559
209525067
21.304
42929125
19.188
21745243
24487309
13671081
12.170
22.132
10.055
11609588
12947426
5861268
4495283
2407885
2931045
1230495
3890407
2697977
1586128
8993852
1368059
589831
723247
1210929
502182
11.302
13.051
7.612
8.667
9.355
12.648
16.688
17.673
20.599
19.118
21.875
22.808
24.609
1.708
2.815
4.255
5.125
5.648

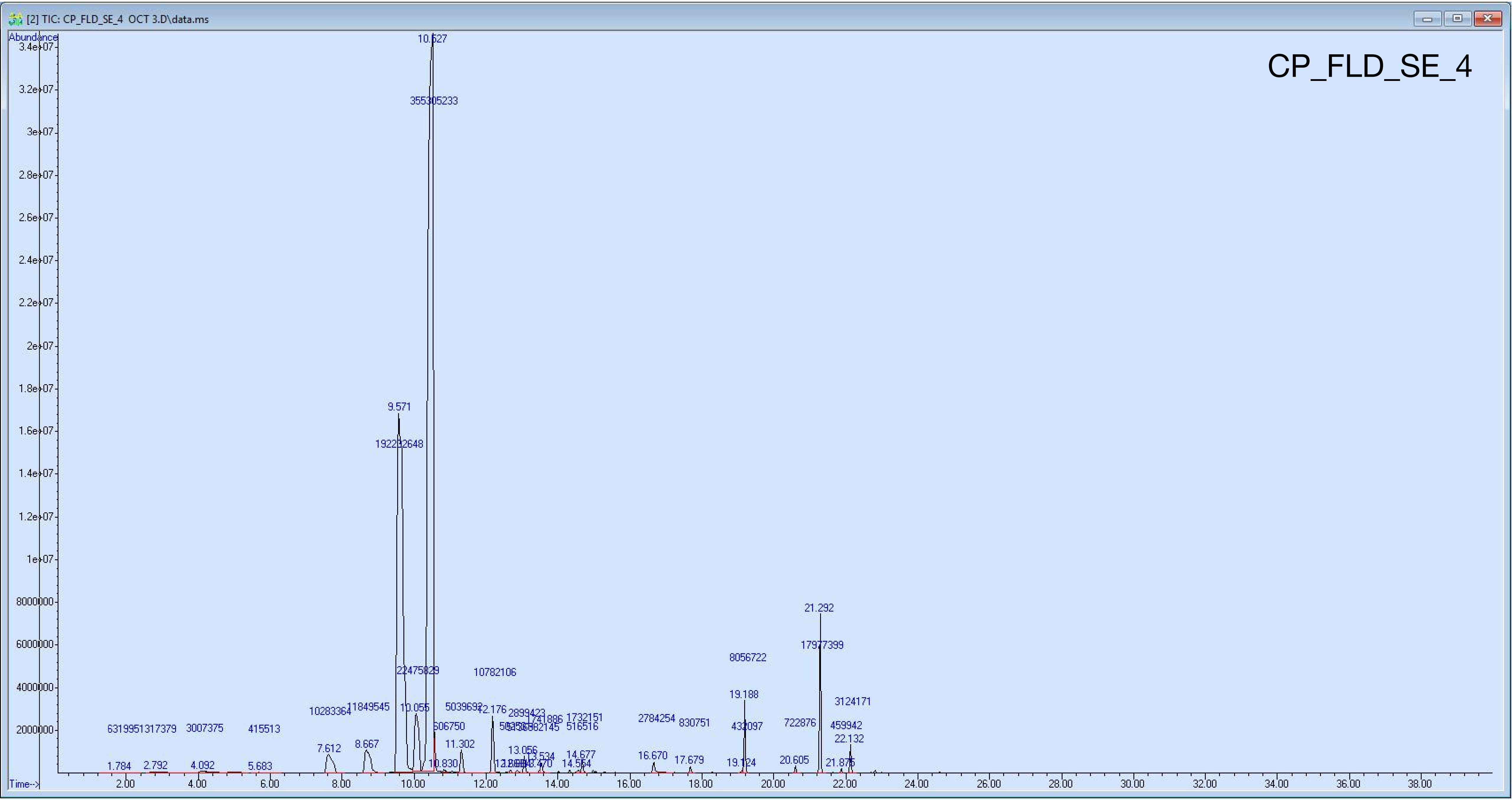
[2] TIC: CP_FLD_SE_4 OCT 3.D\data.ms
CP_FLD_SE_4
Abundance
Time-->
9.571
10.527
21.292
19.188

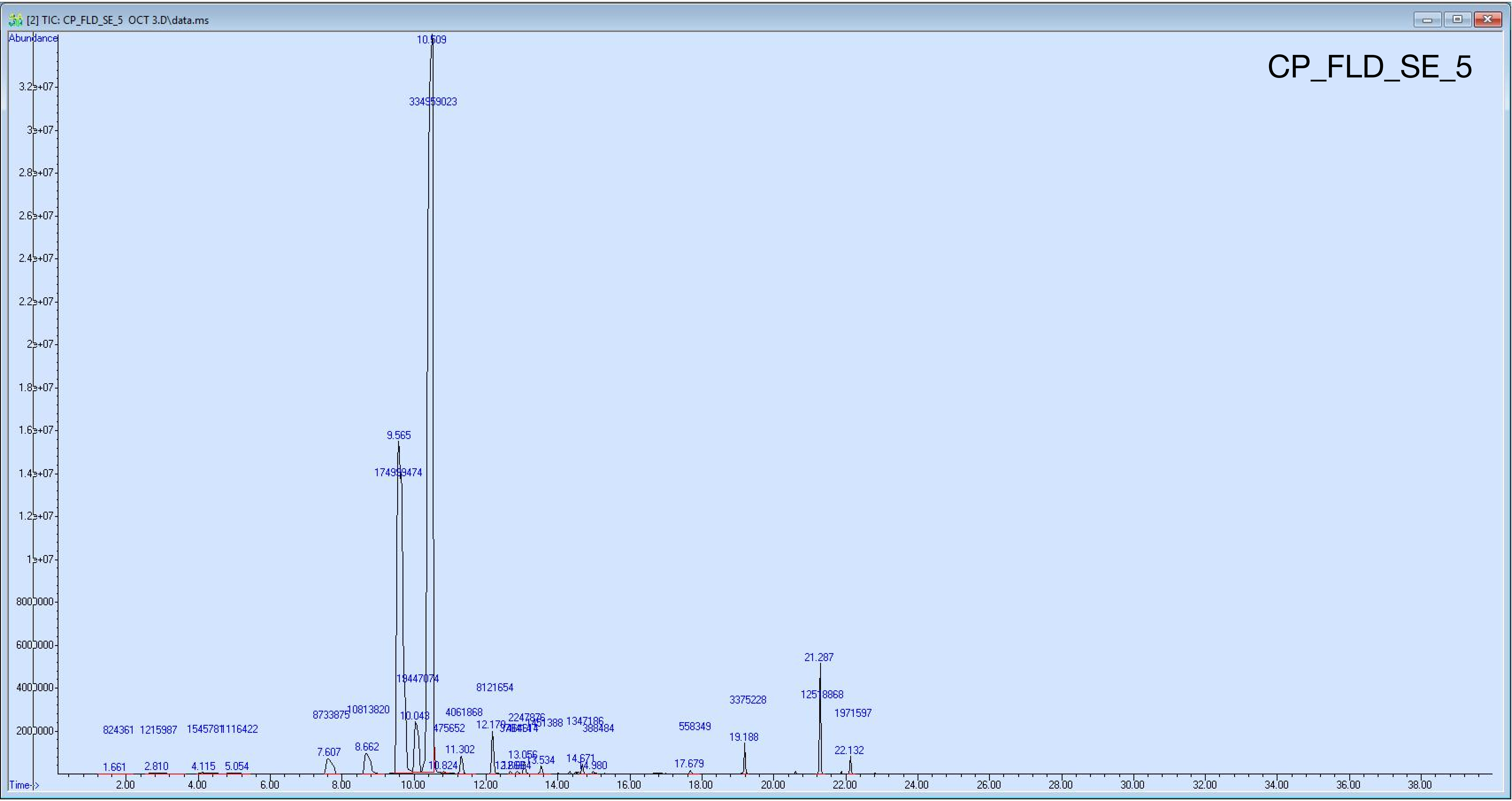
[2] TIC: CP_FLD_SE_5 OCT 3.D\data.ms
CP_FLD_SE_5
Abundance
Time-->
10.509
334959023
9.565
174959474
21.287
12518868
19.188
3375228
22.132
1971597
8121654
12.179
10.043
19447074
11.302
4061868
7.607
8733875
8.662
10813820
17.679
558349
1.661
824361
2.810
1215987
4.115
1545781
5.054
1116422

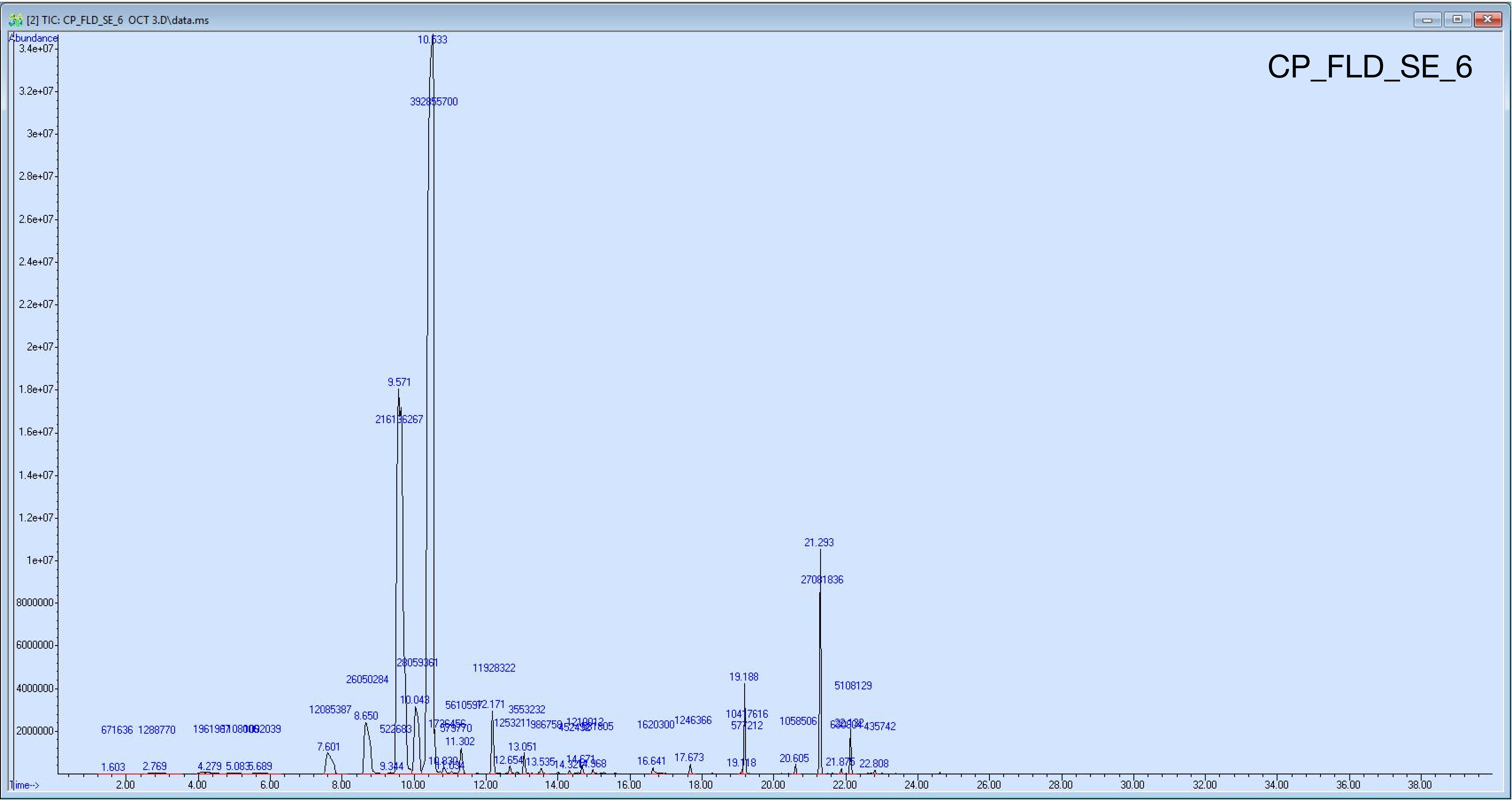
[2] TIC: CP_FLD_SE_6 OCT 3.D\data.ms
CP_FLD_SE_6
Abundance
Time-->
10.533
9.571
21.293
27081836
19.188
1.603
2.769
4.279
5.083
5.689
7.601
8.650
9.344
10.043
11.302
12.171
12.654
13.051
13.535
16.641
17.673
19.118
20.605
21.875
22.808
671636
1288770
26050284
12085387
11928322
3553232
1620300
1246366
10417616
577212
1058506
5108129
435742

Cherokee Purple (heirloom)
Flooding
Manduca sexta-damaged

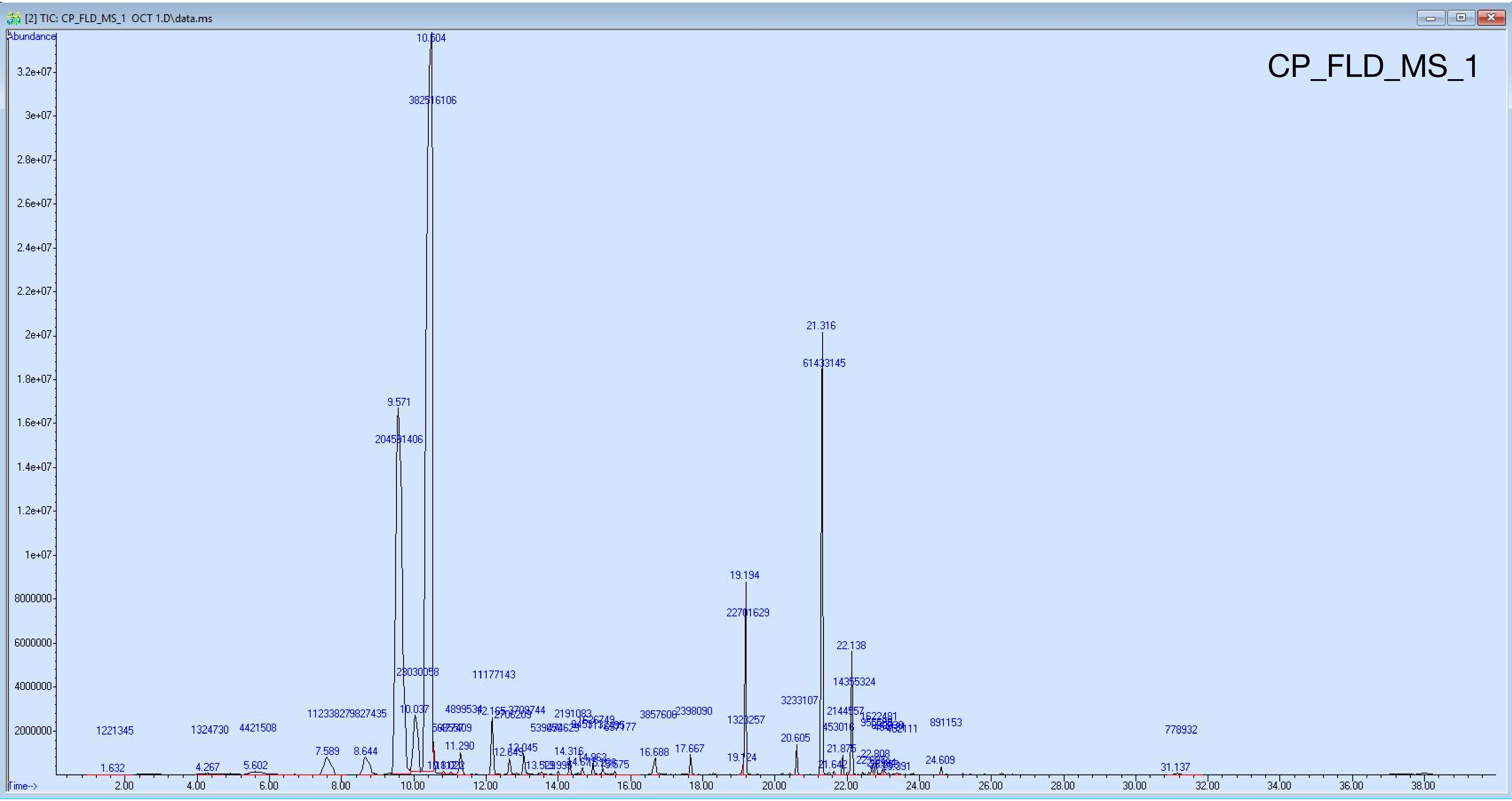

[2] TIC: CP_FLD_MS_1 OCT 1.D\data.ms
CP_FLD_MS_1
Abundance
Time-->
3.2e+07
3e+07
2.8e+07
2.6e+07
2.4e+07
2.2e+07
2e+07
1.8e+07
1.6e+07
1.4e+07
1.2e+07
1e+07
8000000
6000000
4000000
2000000
2.00
4.00
6.00
8.00
10.00
12.00
14.00
16.00
18.00
20.00
22.00
24.00
26.00
28.00
30.00
32.00
34.00
36.00
38.00
10.504
382516106
9.571
204591406
21.316
61433145
19.194
22701629
22.138
14355324
23030058
11177143
10.037
1.632
1221345
4.267
1324730
5.602
4421508
7.589
8.644
11.290
12.649
13.045
14.316
16.688
3857606
17.667
2398090
19.124
1323257
20.605
3233107
21.875
22.808
24.609
891153
31.137
778932

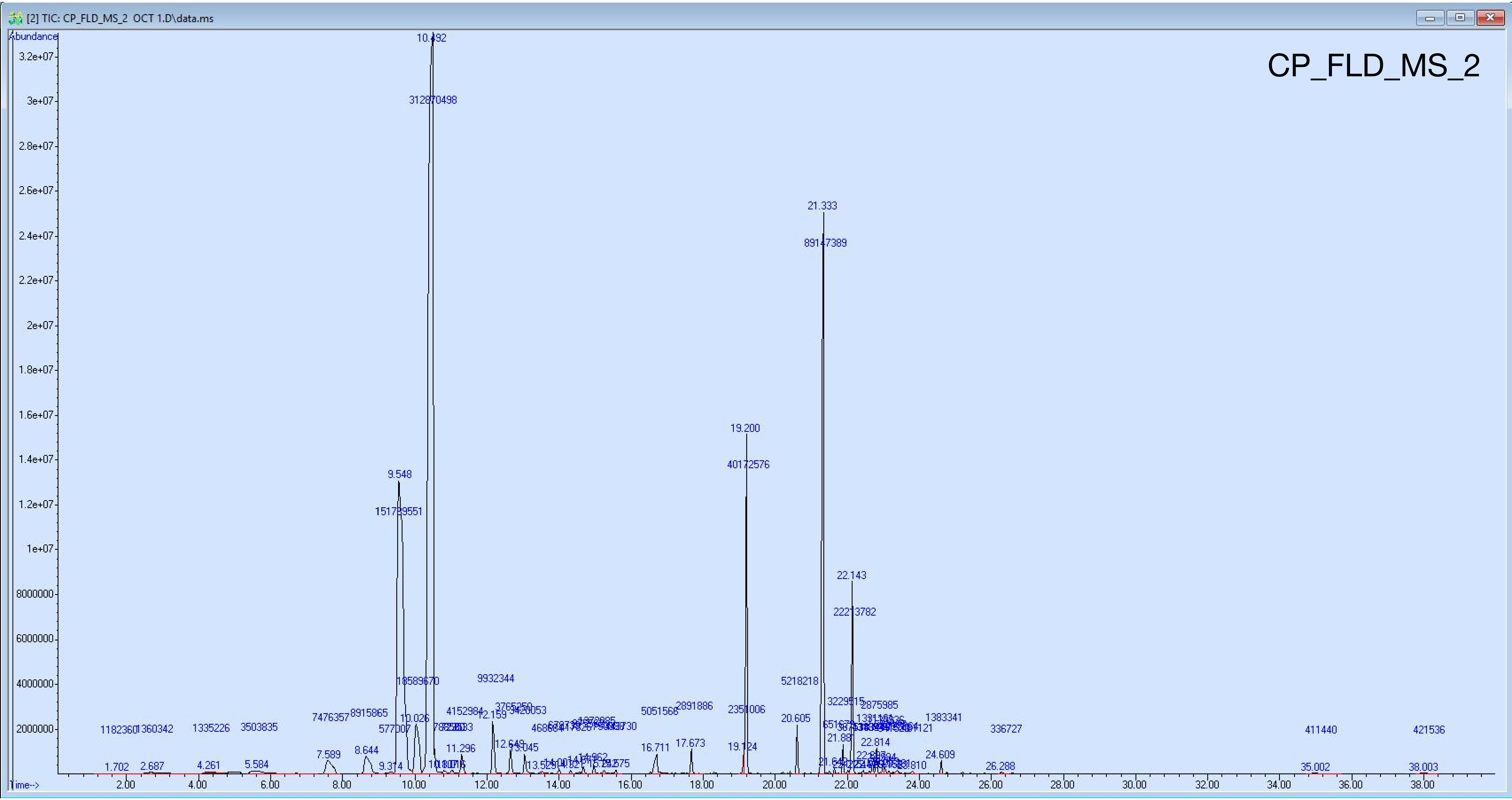
[2] TIC: CP_FLD_MS_2 OCT 1.D\data.ms
CP_FLD_MS_2
Abundance
Time-->
3.2e+07
3e+07
2.8e+07
2.6e+07
2.4e+07
2.2e+07
2e+07
1.8e+07
1.6e+07
1.4e+07
1.2e+07
1e+07
8000000
6000000
4000000
2000000
2.00
4.00
6.00
8.00
10.00
12.00
14.00
16.00
18.00
20.00
22.00
24.00
26.00
28.00
30.00
32.00
34.00
36.00
38.00
312870498
21.333
89147389
19.200
40172576
9.548
151729551
22.143
22213782
18589670
9932344
5218218
1.702
2.687
4.261
5.584
7.589
8.644
9.374
10.026
11.296
12.159
16.711
17.673
19.124
20.605
22.814
24.609
26.288
35.002
38.003
1182360
1360342
1335226
3503835
7476357
8915865
577007
4152984
5051566
2891886
2351006
2875985
1383341
336727
411440
421536

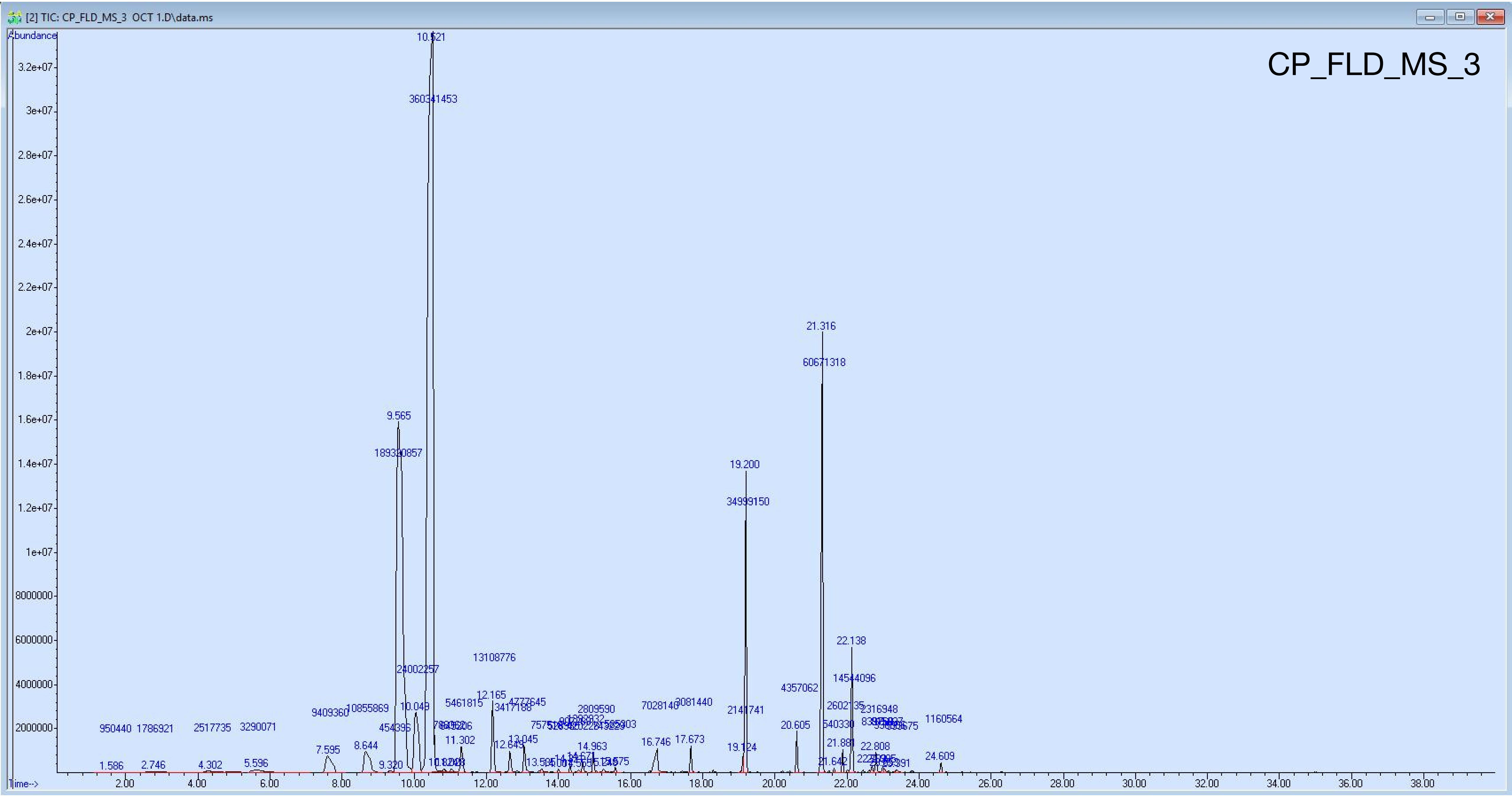
[2] TIC: CP_FLD_MS_3 OCT 1.D\data.ms
CP_FLD_MS_3
Abundance
Time-->
3.2e+07
3e+07
2.8e+07
2.6e+07
2.4e+07
2.2e+07
2e+07
1.8e+07
1.6e+07
1.4e+07
1.2e+07
1e+07
8000000
6000000
4000000
2000000
2.00
4.00
6.00
8.00
10.00
12.00
14.00
16.00
18.00
20.00
22.00
24.00
26.00
28.00
30.00
32.00
34.00
36.00
38.00
10.521
360341453
9.565
189320857
19.200
34999150
21.316
60671318
22.138
14544096
13108776
24002257
10.049
12.165
3417188
4777645
5461815
11.302
12.643
13.045
14.963
2809590
16.746
7028140
17.673
3081440
19.124
2141741
20.605
4357062
2602135
540330
21.881
21.642
2316948
22.808
24.609
1160564
7.595
9409360
8.644
10855869
9.320
454396
1.586
950440
2.746
1786921
4.302
2517735
5.596
3290071

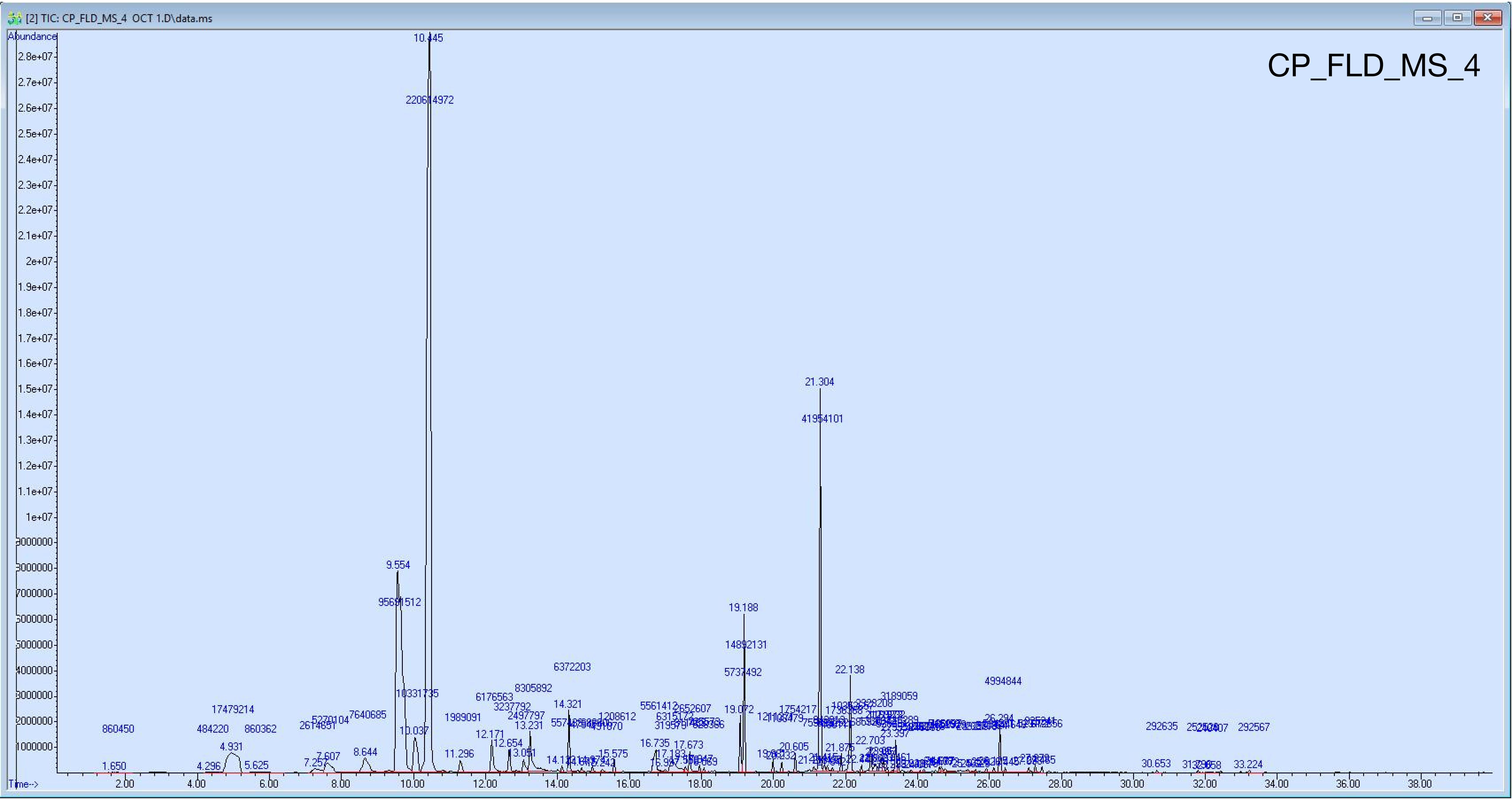
[2] TIC: CP_FLD_MS_4 OCT 1.D\data.ms
CP_FLD_MS_4
Abundance
Time-->
10.445
220614972
9.554
21.304
41954101
19.188
14892131
5737492
22.138
4994844
6372203
3189059
17479214
4.931
8.644
12.171
12.654
14.321
16.735
17.673
20.605
11.296
1.650
30.653
33.224

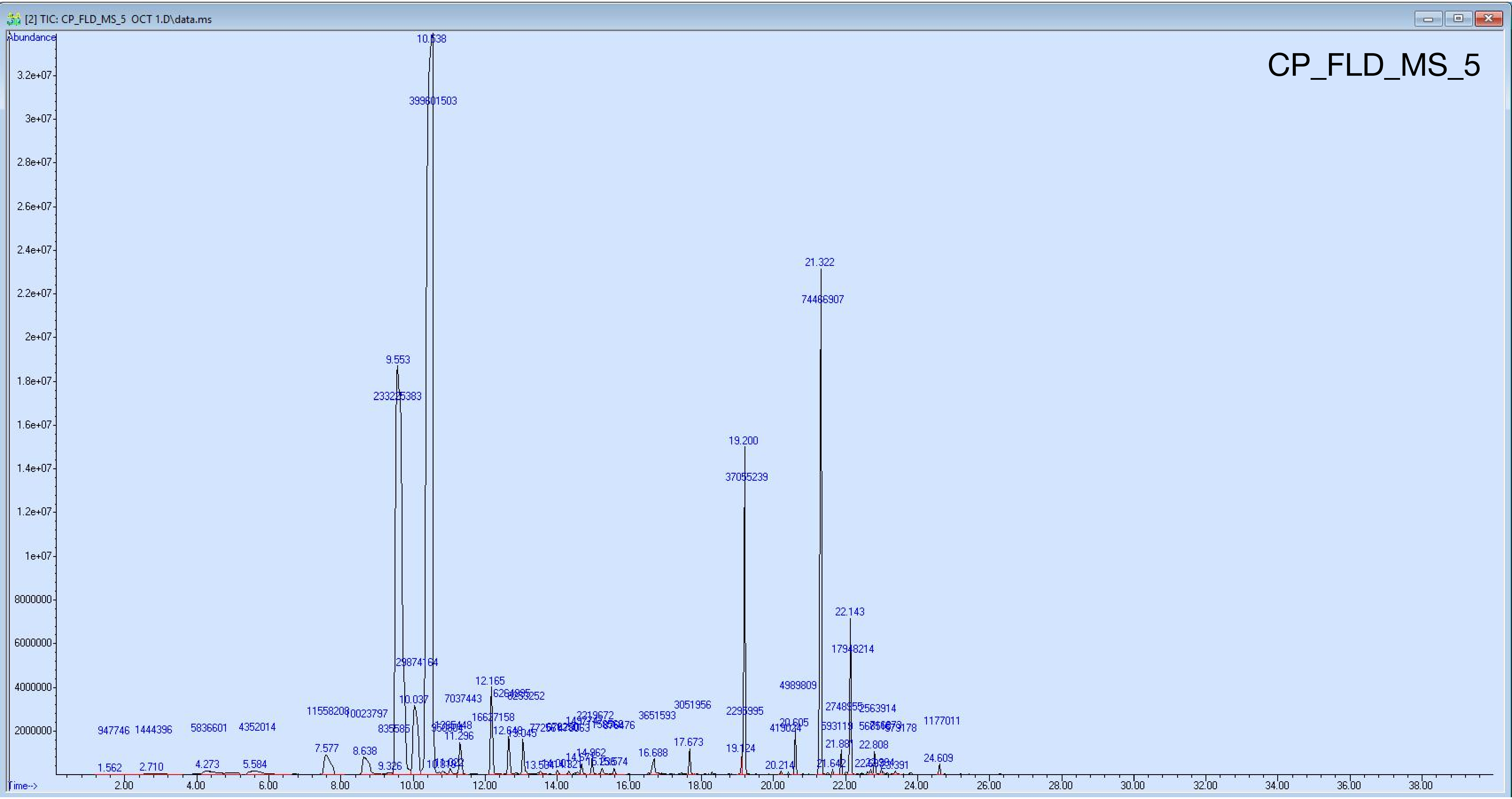
[2] TIC: CP_FLD_MS_5 OCT 1.D\data.ms
CP_FLD_MS_5
Abundance
Time-->
3.2e+07
3e+07
2.8e+07
2.6e+07
2.4e+07
2.2e+07
2e+07
1.8e+07
1.6e+07
1.4e+07
1.2e+07
1e+07
8000000
6000000
4000000
2000000
2.00
4.00
6.00
8.00
10.00
12.00
14.00
16.00
18.00
20.00
22.00
24.00
26.00
28.00
30.00
32.00
34.00
36.00
38.00
21.322
74486907
19.200
37055239
22.143
17948214
9.553
1.562
947746
2.710
1444396
4.273
5836601
5.584
4352014
7.577
11558208
8.638
10.037
7037443
12.165
11.296
16627158
16.688
3651593
17.673
3051956
19.124
2296995
20.214
20.605
4989809
21.881
22.808
24.609
1177011

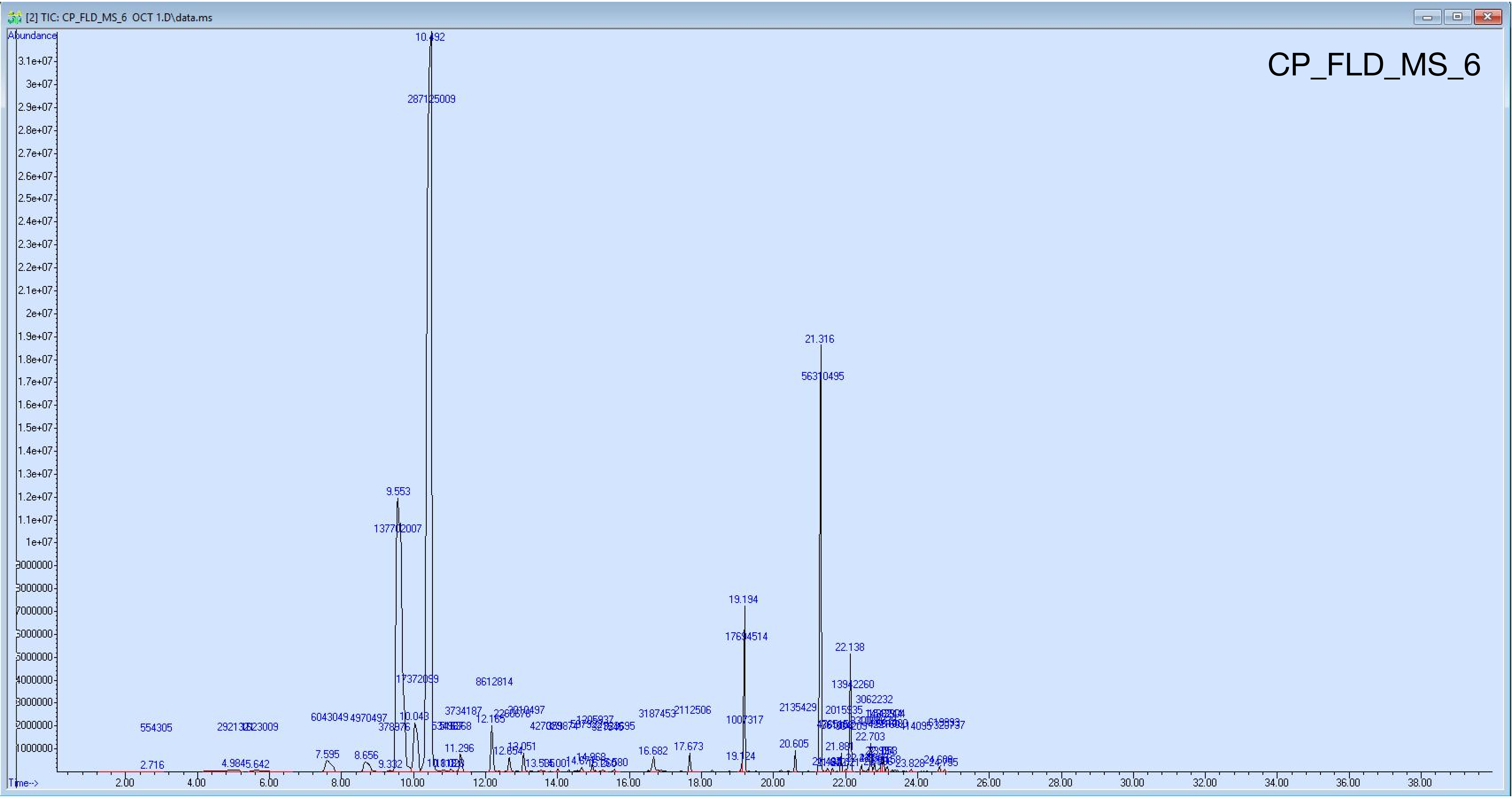
[2] TIC: CP_FLD_MS_6 OCT 1.D\data.ms
CP_FLD_MS_6
Abundance
Time-->
10.492
28712509
9.553
13770007
19.194
17694514
21.316
56310495
22.138
13942260

New Girl (hybrid)
No Flood
No Herbivory

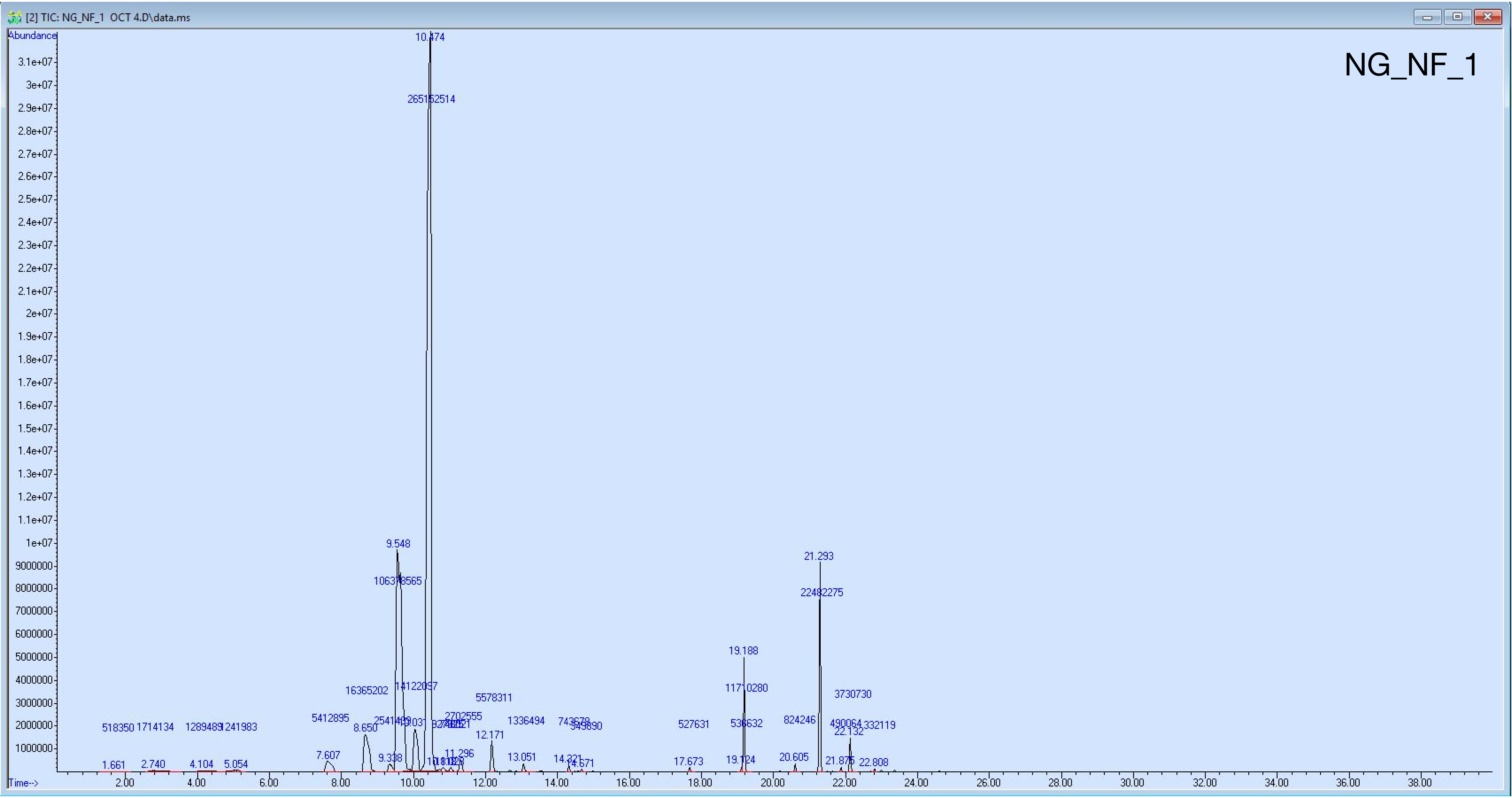
[2] TIC: NG_NF_1 OCT 4.D\data.ms
NG_NF_1
Abundance
Time-->
10.474
265152514
9.548
106378565
21.293
22482275
19.188
11710280
3730730
22.132
16365202
14122097
5578311
12.171
5412895
8.650
7.607
9.338
11.296
2702555
1336494
13.051
14.321
14.571
527631
17.673
536632
19.124
824246
20.605
21.875
22.808
332119
518350
1714134
1289489
1241983
1.661
2.740
4.104
5.054

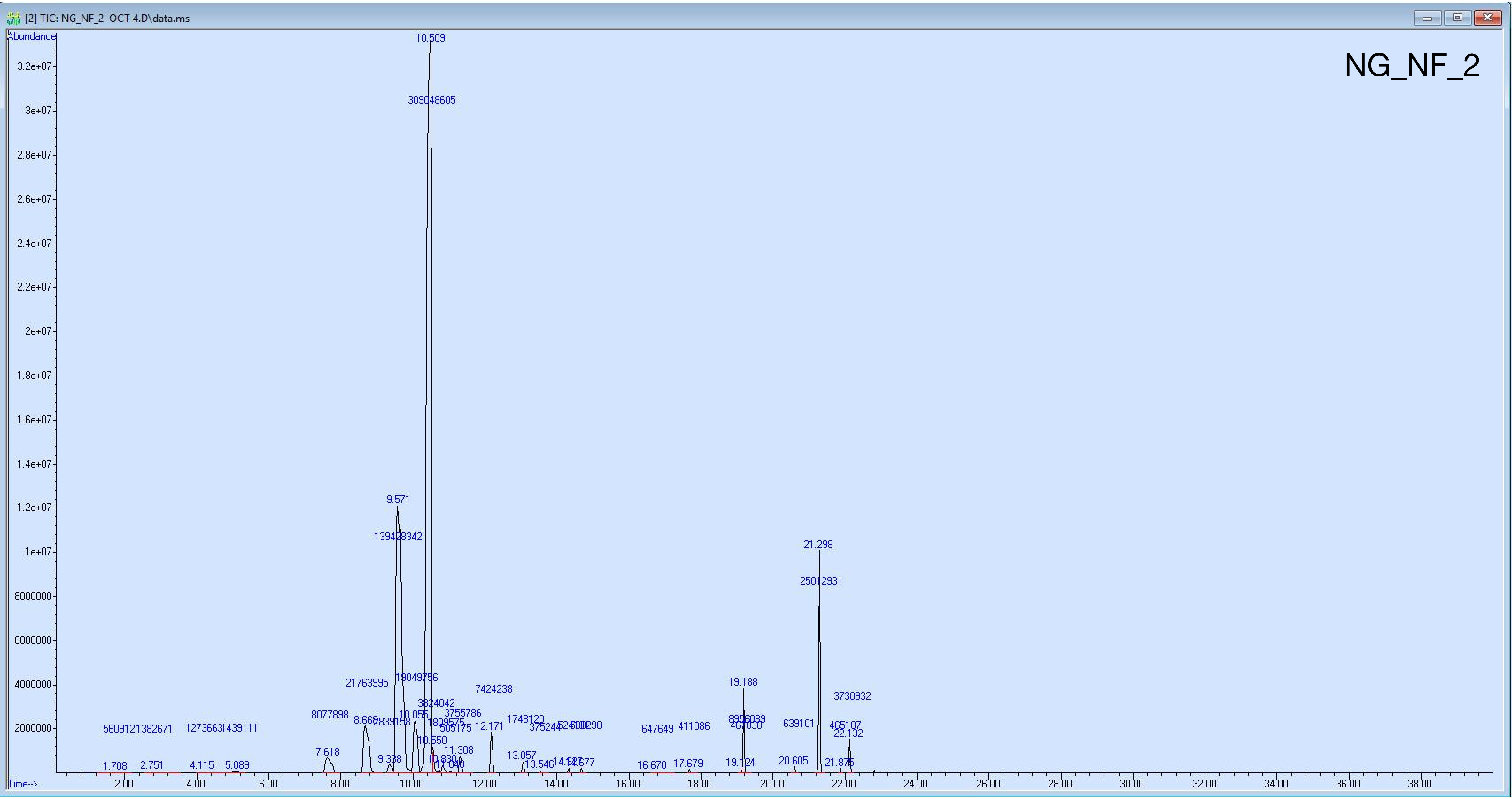

[2] TIC: NG_NF_2 OCT 4.D\data.ms
NG_NF_2
Abundance
Time-->
10.509
309048605
9.571
139428342
21.298
25012931
19.188
3730932
7424238
21763995
8077898
22.132

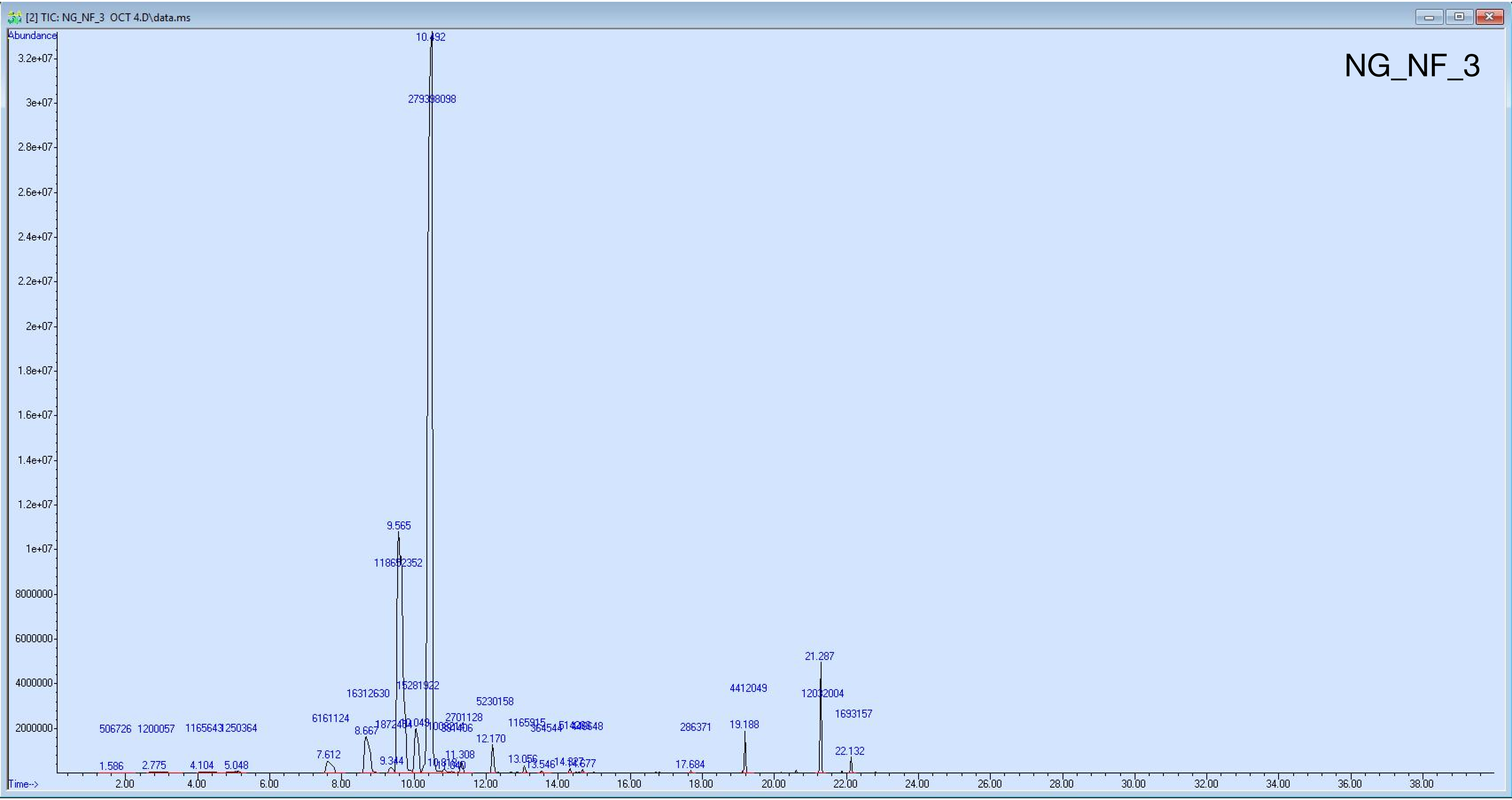
[2] TIC: NG_NF_3 OCT 4.D\data.ms
NG_NF_3
Abundance
Time-->
10.492
279398098
9.565
21.287
19.188
22.132
12.170
8.667
7.612

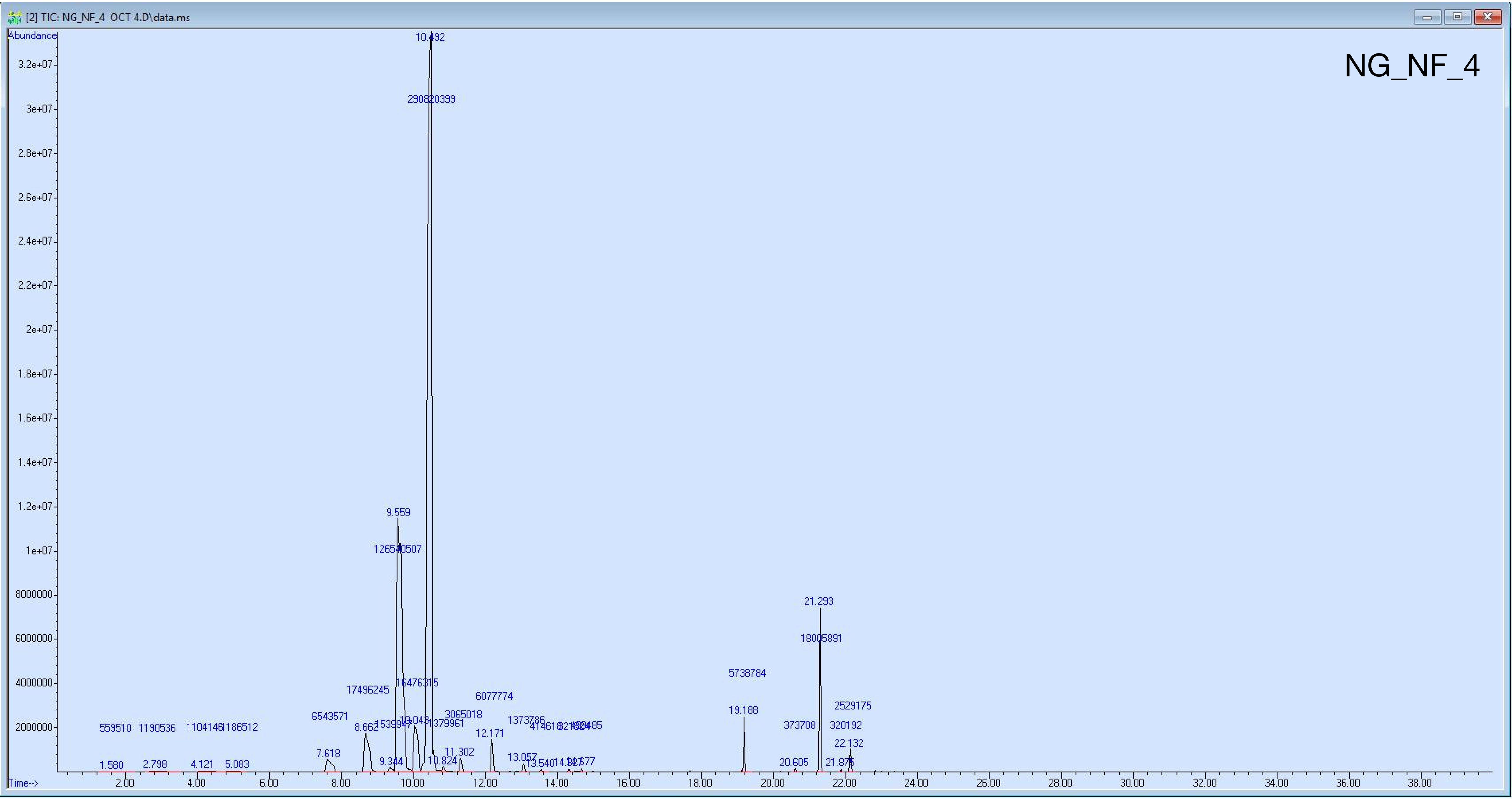

[2] TIC: NG_NF_4 OCT 4.D\data.ms
NG_NF_4
Abundance
Time-->
10.492
290820399
9.559
126540507
21.293
18005891
19.188
5738784
12.171
6077774
17496245
16476315
8.662
7.618
6543571

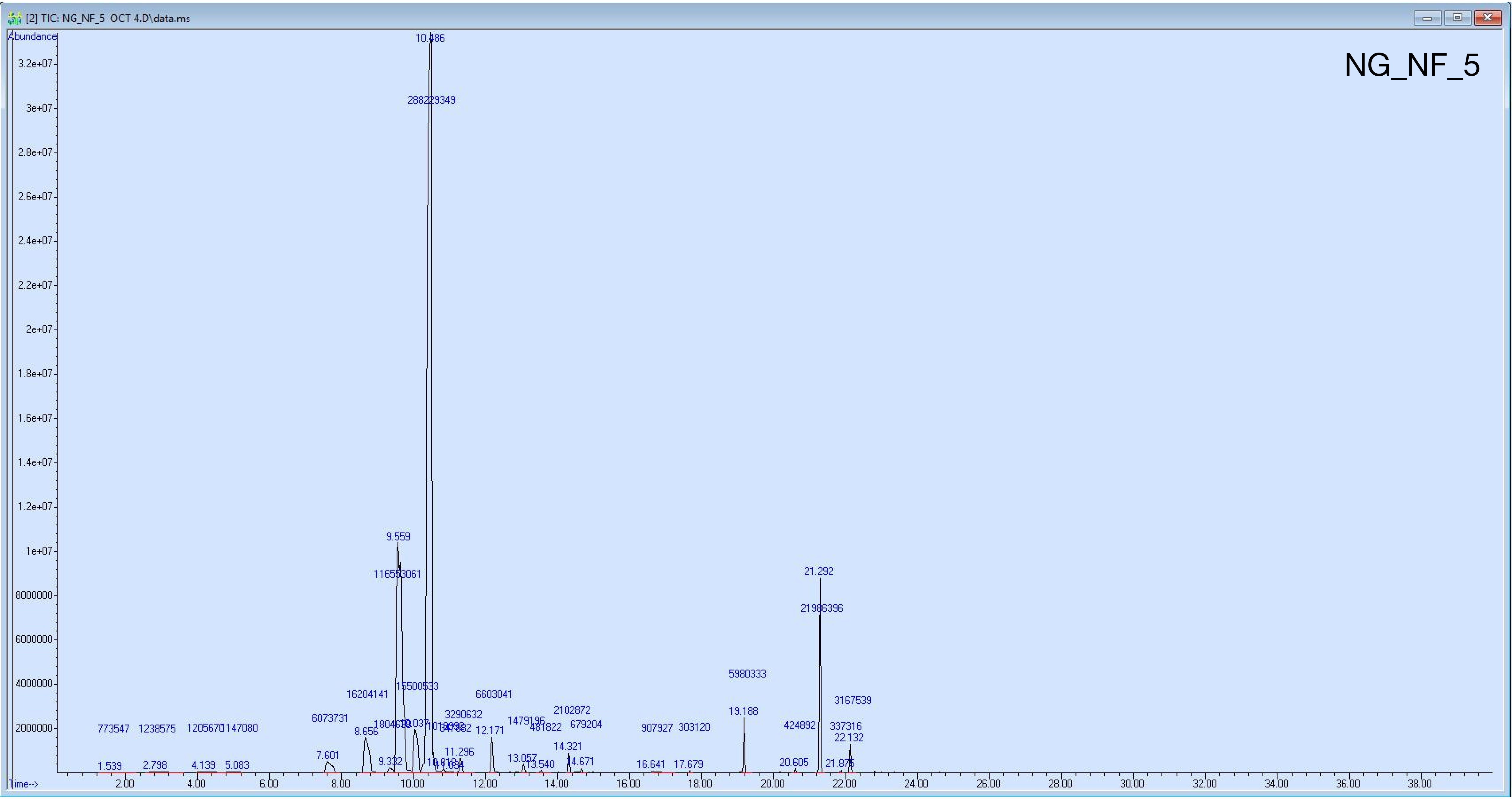
[2] TIC: NG_NF_5 OCT 4.D\data.ms
NG_NF_5
Abundance
Time-->
10.486
288229349
9.559
116553061
21.292
21986396
19.188
5980333
12.171
6603041
8.656
16204141
7.601
6073731
22.132
3167539
337316
14.321
2102872
14.671
679204
20.605
424892
21.875
13.057
1479196
13.540
481822
16.641
907927
17.679
303120
11.296
3290632
1.539
773547
2.798
1238575
4.139
1205670
5.083
1147080
9.332
15500533

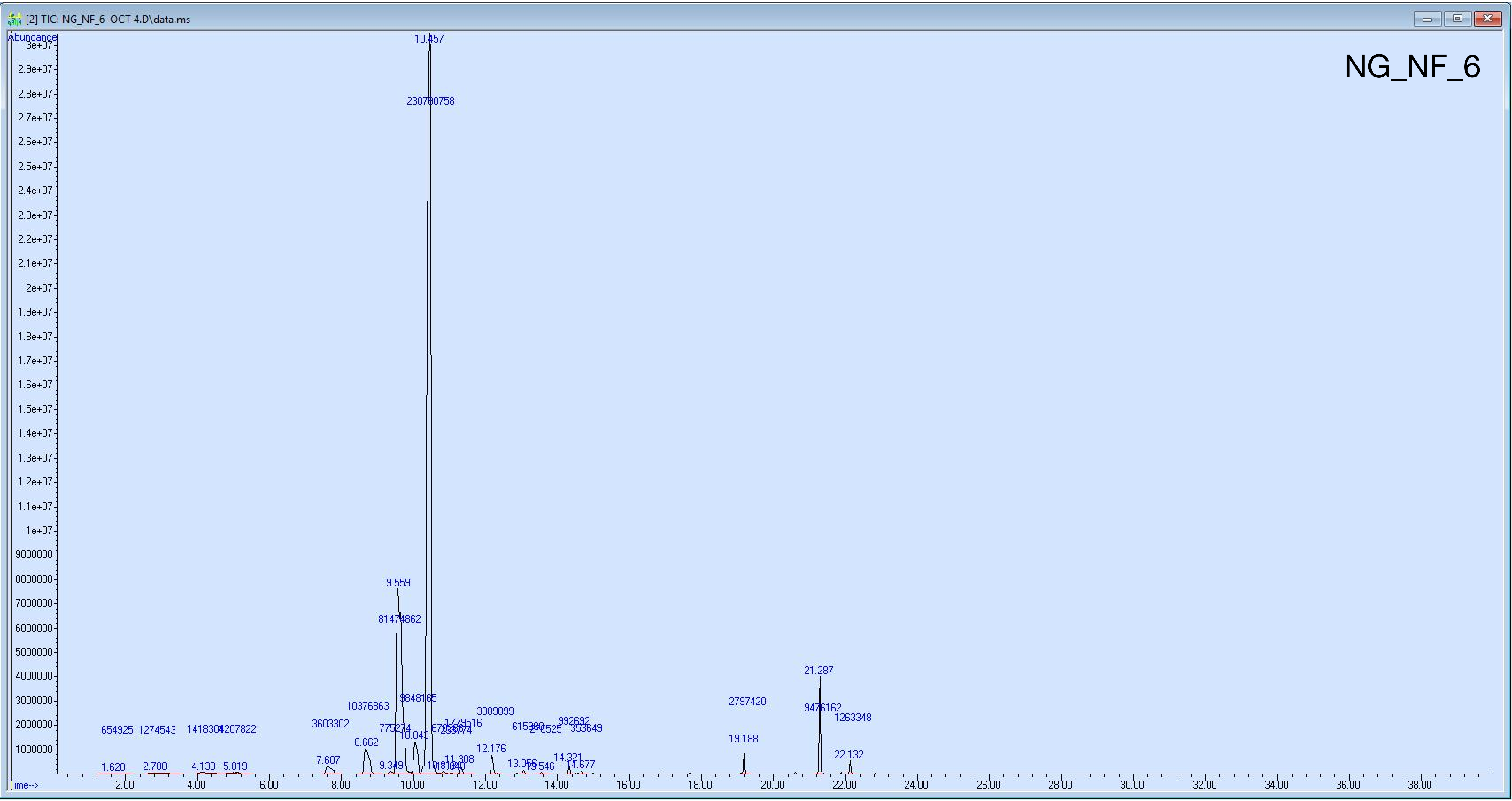
[2] TIC: NG_NF_6 OCT 4.D\data.ms
NG_NF_6
Abundance
Time-->
10.457
230730758
9.559
81474862
21.287
9476162
19.188
2797420
22.132
1263348
12.176
3389899
10.043
9848165
8.662
10376863
7.607
3603302
1.620
654925
2.780
1274543
4.133
5.019
1207822
9.349
775274
11.308
1779516
13.056
13.546
270525
14.321
992692
14.677
353649

New Girl (hybrid)
No Flood
Spodptera exigua-damaged

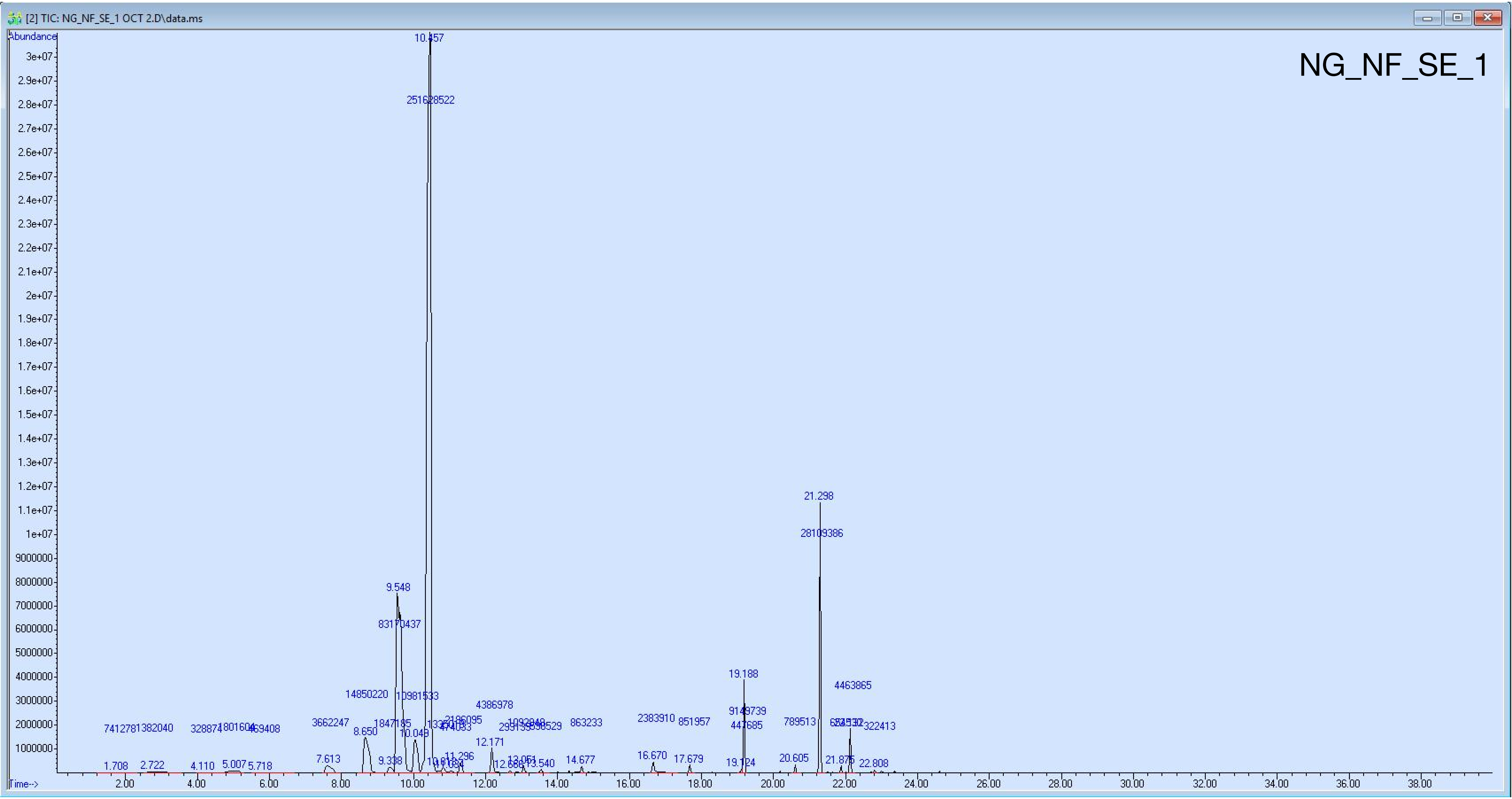
[2] TIC: NG_NF_SE_1 OCT 2.D\data.ms
NG_NF_SE_1
Abundance
Time-->
10.457
251628522
21.298
28109386
9.548
83170437
19.188
9149739
447685
4463865
14850220
10981533
4386978
3662247
8.650
10.049
12.171
7.613
9.338
11.296
14.677
16.670
17.679
19.124
20.605
21.875
22.808
1.708
2.722
4.110
5.007
5.718
863233
2383910
851957
789513
322413
1847185
2186095
13.540

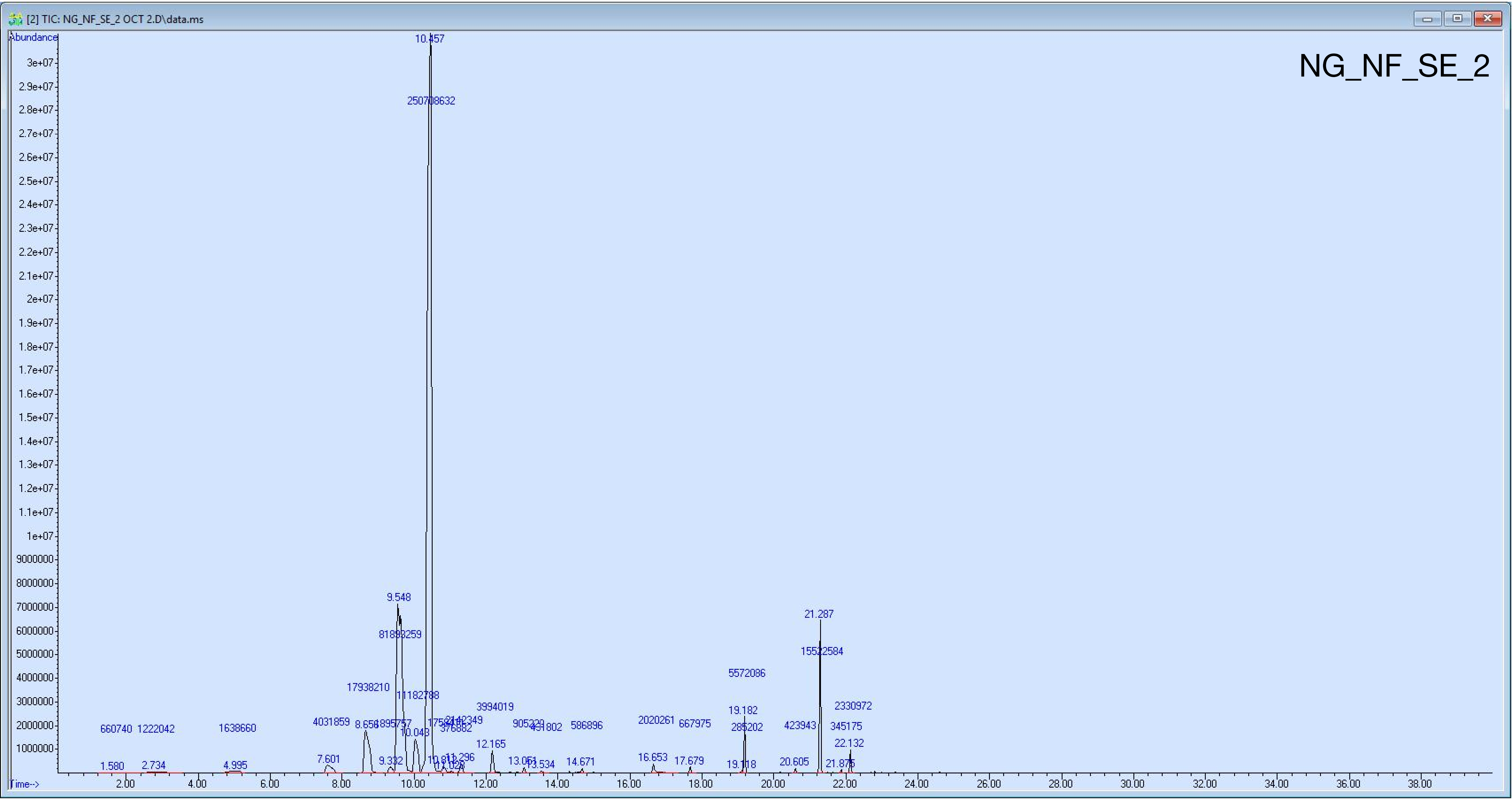
[2] TIC: NG_NF_SE_2 OCT 2.D\data.ms
NG_NF_SE_2
Abundance
Time-->
10.457
250708632
9.548
21.287
15522584
5572086
19.182
285202
2330972
22.132
345175
423943
17938210
11182788
3994019
12.165
4031859
7.601
660740
1222042
1638660
1.580
2.734
4.995
9.332
10.043
16.653
2020261
667975
17.679
586896
14.671
13.534
20.605
21.875
19.118

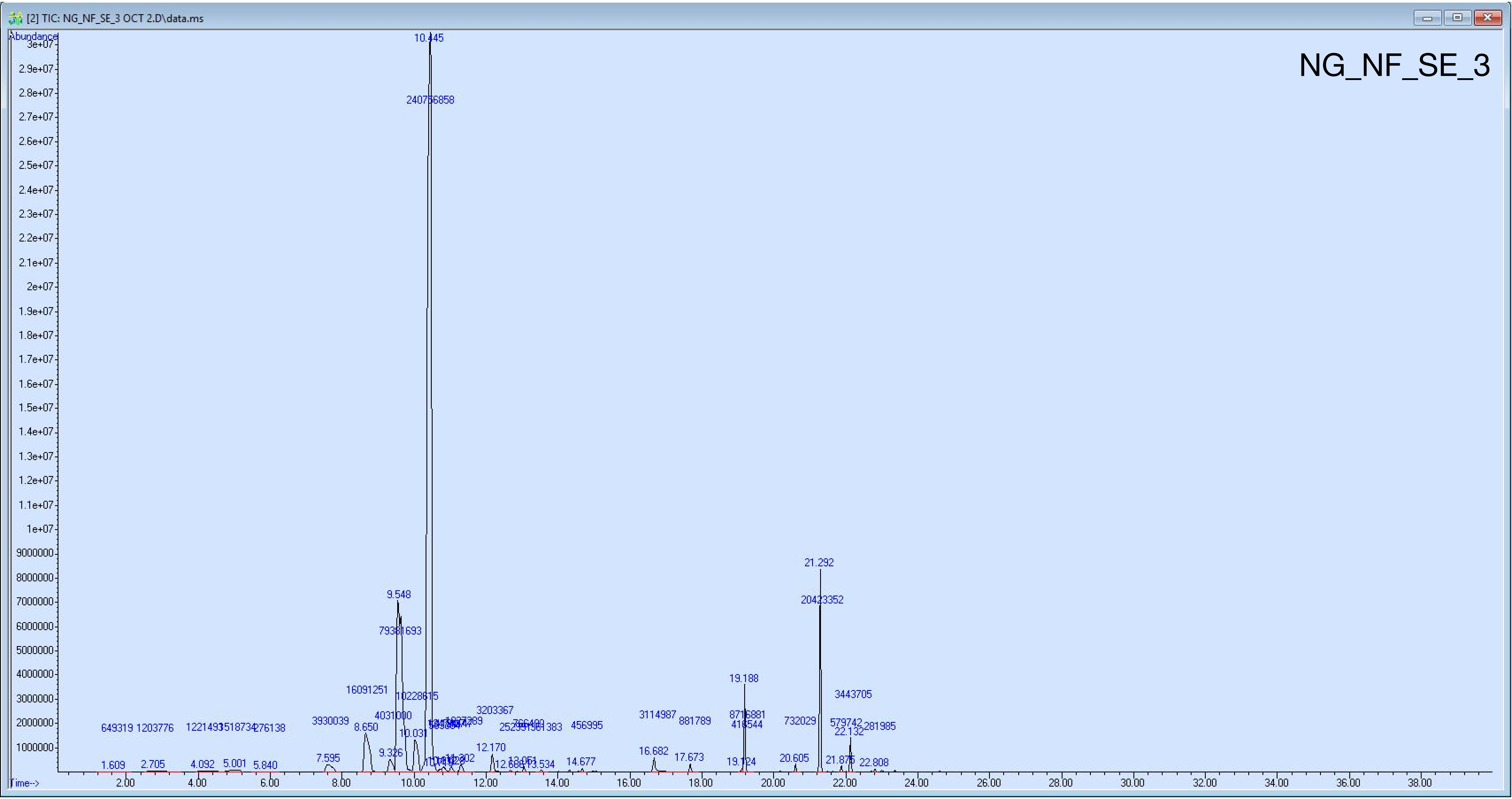
[2] TIC: NG_NF_SE_3 OCT 2.D\data.ms
NG_NF_SE_3
Abundance
Time-->
10.445
240756858
9.548
79381693
21.292
20423352
19.188
3443705
16091251
10228615
3203367
3114987
881789
8716881
732029
579742
281985
22.132
649319
1203776
3930039
8.650
4031000
10.031
456995
12.170
1.609
2.705
4.092
5.001
5.840
7.595
9.326
14.677
16.682
17.673
19.124
20.605
21.875
22.808

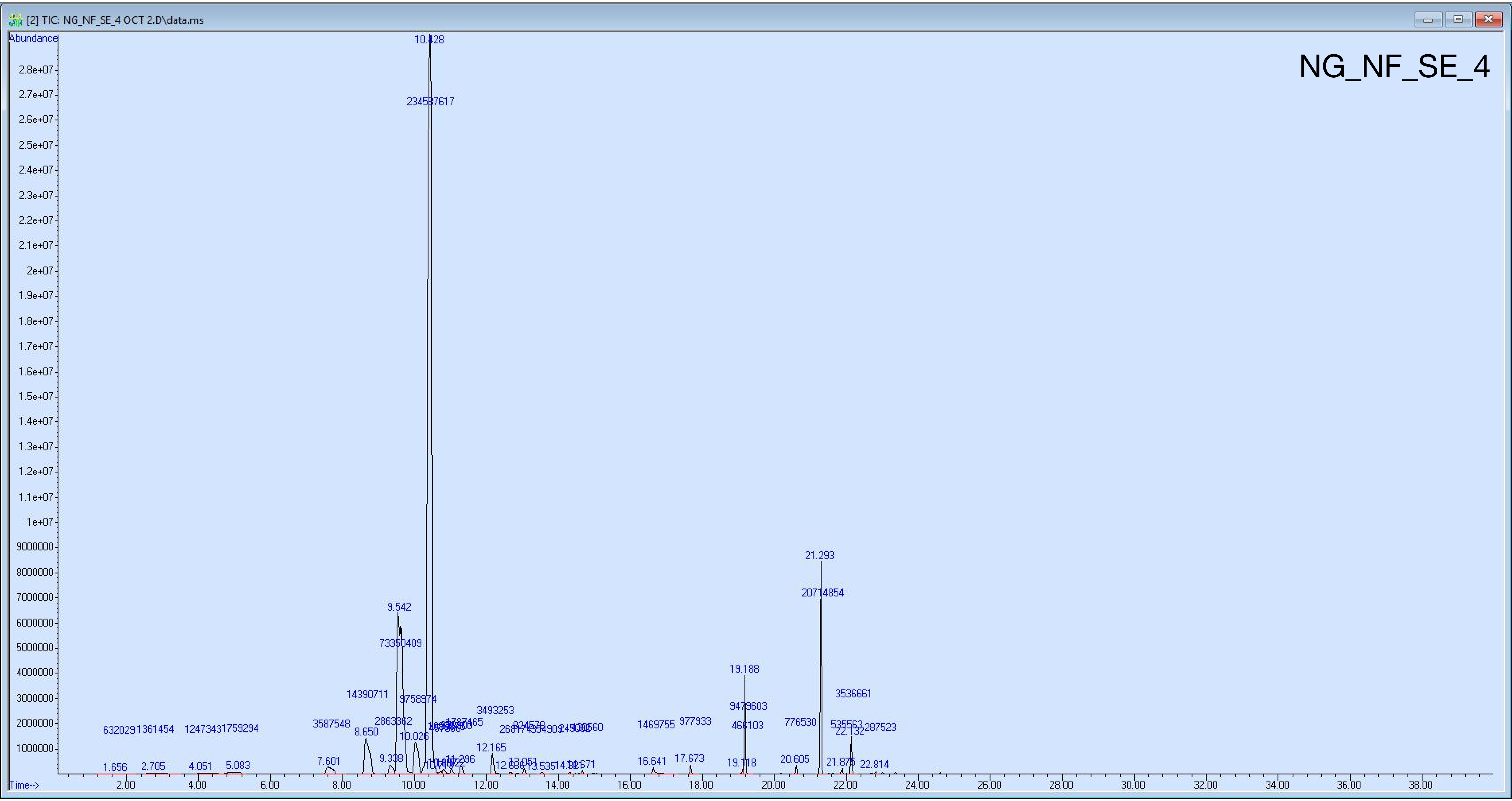
[2] TIC: NG_NF_SE_4 OCT 2.D\data.ms
NG_NF_SE_4
Abundance
Time-->
10.428
234537617
9.542
73350409
14390711
9758974
2863362
3587548
8.650
9.338
10.026
7.601
632029
1361454
1247343
1759294
1.656
2.705
4.051
5.083
3493253
12.165
11.296
1469755
977933
16.641
17.673
19.188
9479603
466103
19.118
21.293
20714854
776530
20.605
3536661
535563
287523
22.132
21.875
22.814
2.8e+07
2.7e+07
2.6e+07
2.5e+07
2.4e+07
2.3e+07
2.2e+07
2.1e+07
2e+07
1.9e+07
1.8e+07
1.7e+07
1.6e+07
1.5e+07
1.4e+07
1.3e+07
1.2e+07
1.1e+07
1e+07
9000000
8000000
7000000
6000000
5000000
4000000
3000000
2000000
1000000
2.00
4.00
6.00
8.00
10.00
12.00
14.00
16.00
18.00
20.00
22.00
24.00
26.00
28.00
30.00
32.00
34.00
36.00
38.00

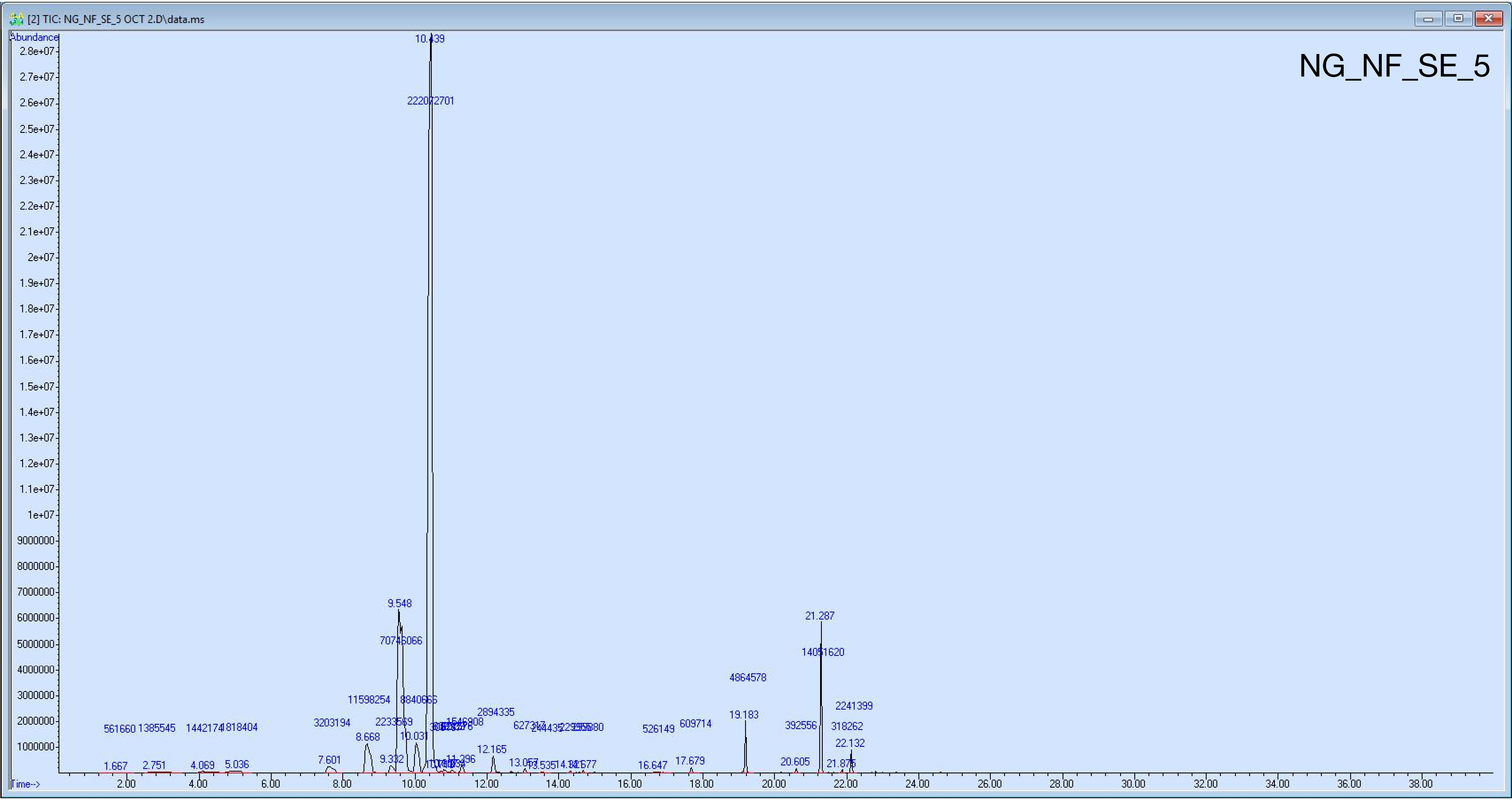
[2] TIC: NG_NF_SE_5 OCT 2.D\data.ms
NG_NF_SE_5
Abundance
Time-->
10.439
222072701
9.548
70746066
21.287
14051620
4864578
19.183
11598254
2241399
2894335
3203194
8.668
10.031
12.165
22.132
392556
318262
526149
609714
561660
1385545
1.667
2.751
4.069
5.036
7.601
9.332
13.057
16.647
17.679
20.605

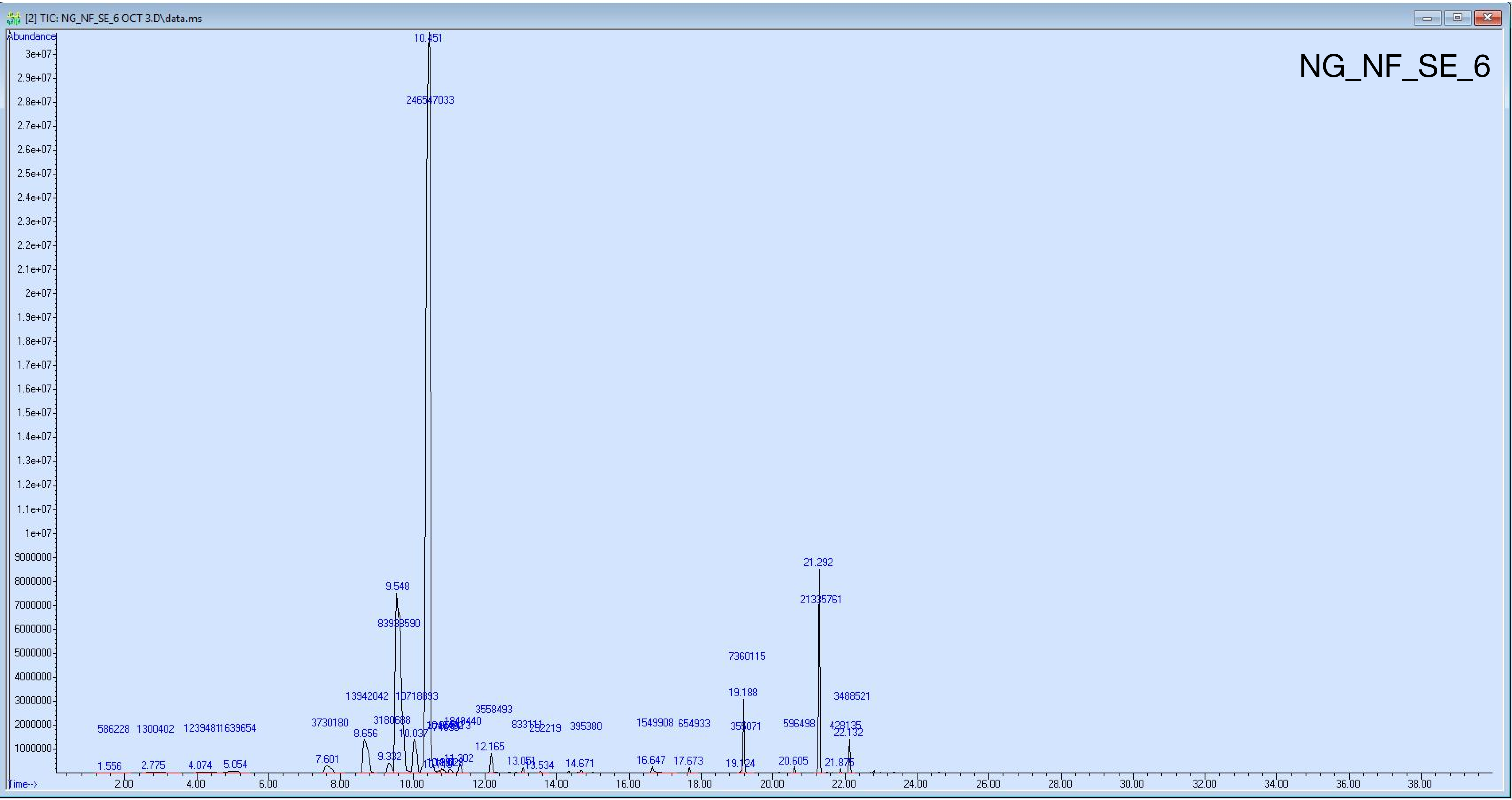
[2] TIC: NG_NF_SE_6 OCT 3.D\data.ms
NG_NF_SE_6
Abundance
Time-->
10.451
246547033
9.548
83933590
21.292
21335761
7360115
19.188
3488521
13942042
10718893
3558493
3730180
586228
1300402
1239481
1639654
8.656
7.601
12.165
16.647
17.673
20.605
22.132

New Girl (hybrid)
No Flood
Manduca sexta-damaged

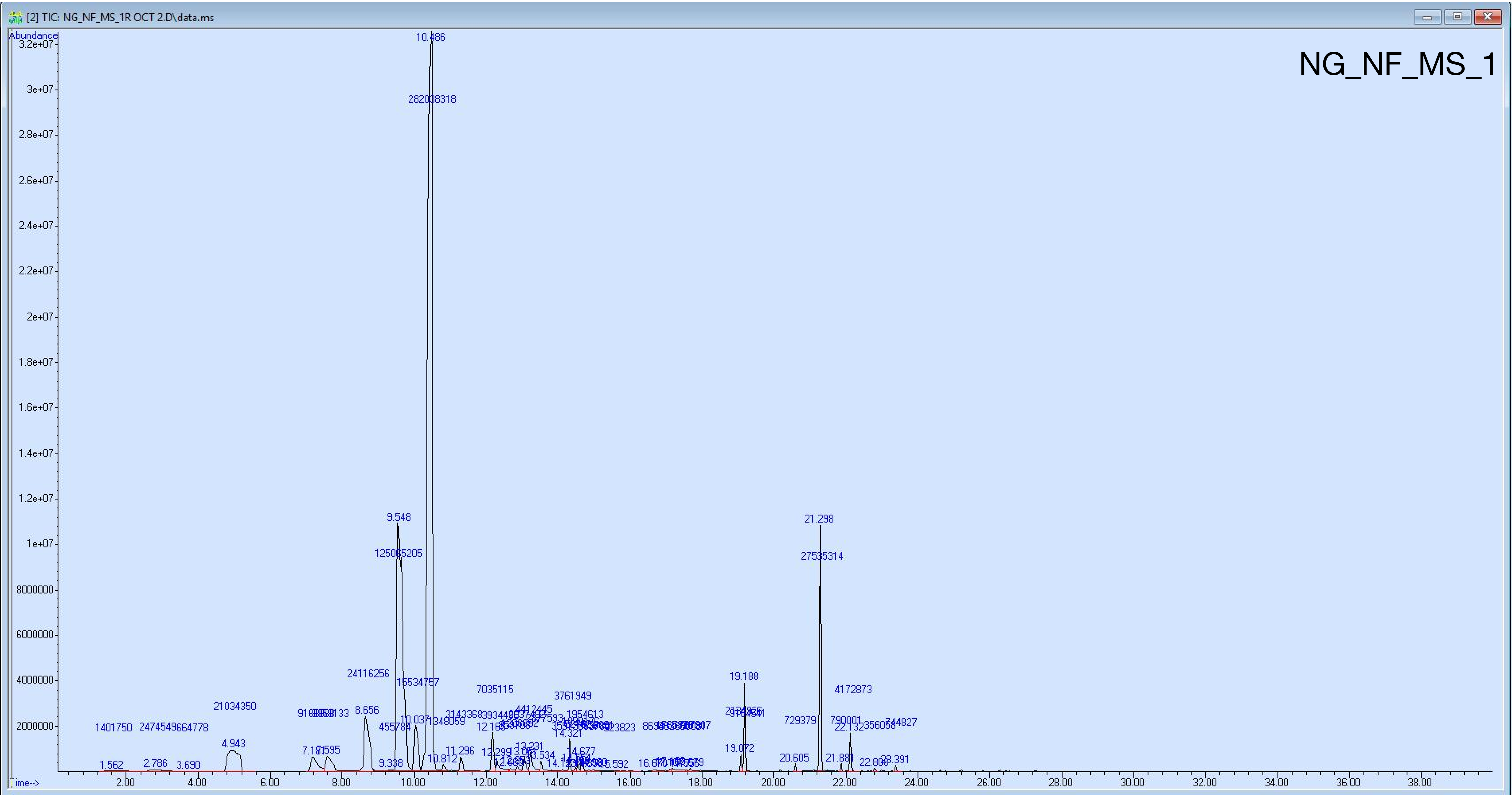
[2] TIC: NG_NF_MS_1R OCT 2.D\data.ms
NG_NF_MS_1
Abundance
Time-->
10.486
282038318
9.548
125085205
21.298
27535314
19.188
4.943
21034350
8.656
24116256
4172873
3761949
7035115
1401750
1.562
2.786
3.690
20.605
729379
22.132
744827
23.391

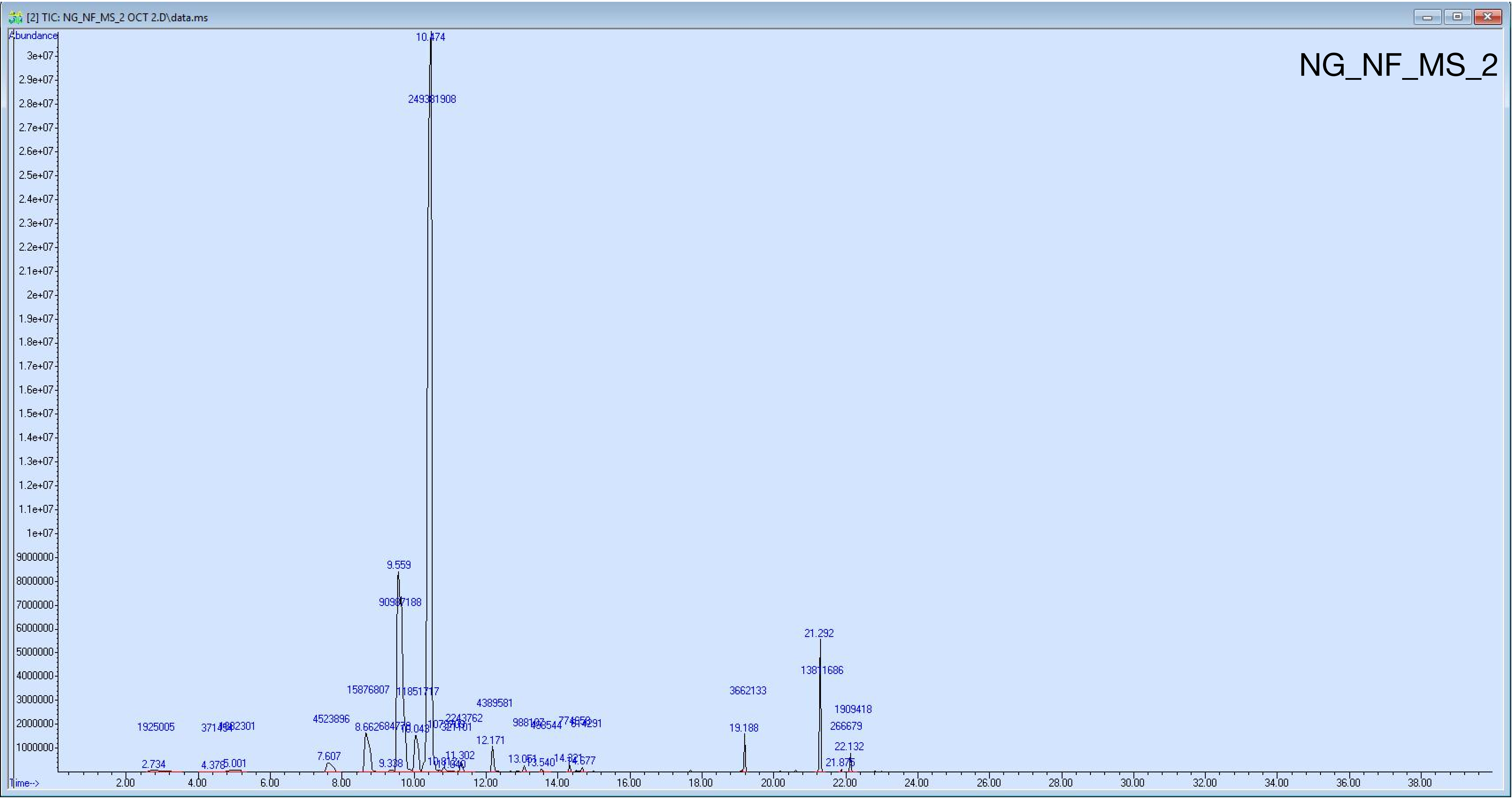
[2] TIC: NG_NF_MS_2 OCT 2.D\data.ms
NG_NF_MS_2
Abundance
Time-->
10.474
249381908
9.559
90987188
21.292
13811686
19.188
3662133
15876807
11851717
4389581
4523896
1925005
2.734
4.378
5.001
7.607
12.171
22.132
1909418
266679
21.875
11.302
2243762

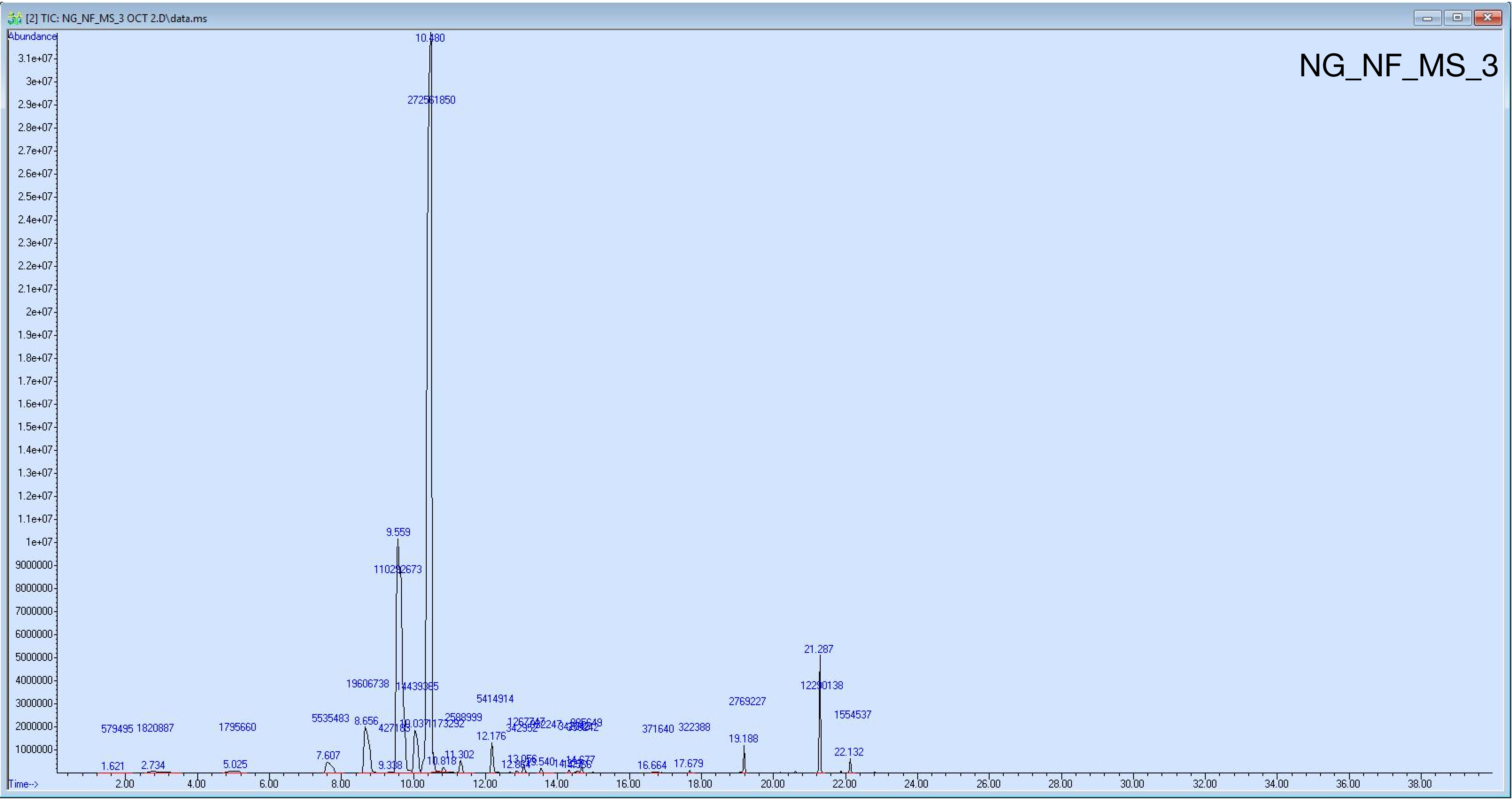

[2] TIC: NG_NF_MS_3 OCT 2.D\data.ms
NG_NF_MS_3
Abundance
Time-->
10.480
272561850
9.559
110292673
21.287
12290138
19.188
2769227
22.132
1554537
12.176
5414914
8.656
19606738
7.607
5535483
5.025
1795660
1.621
579495
2.734
1820887
16.664
371640
17.679
322388

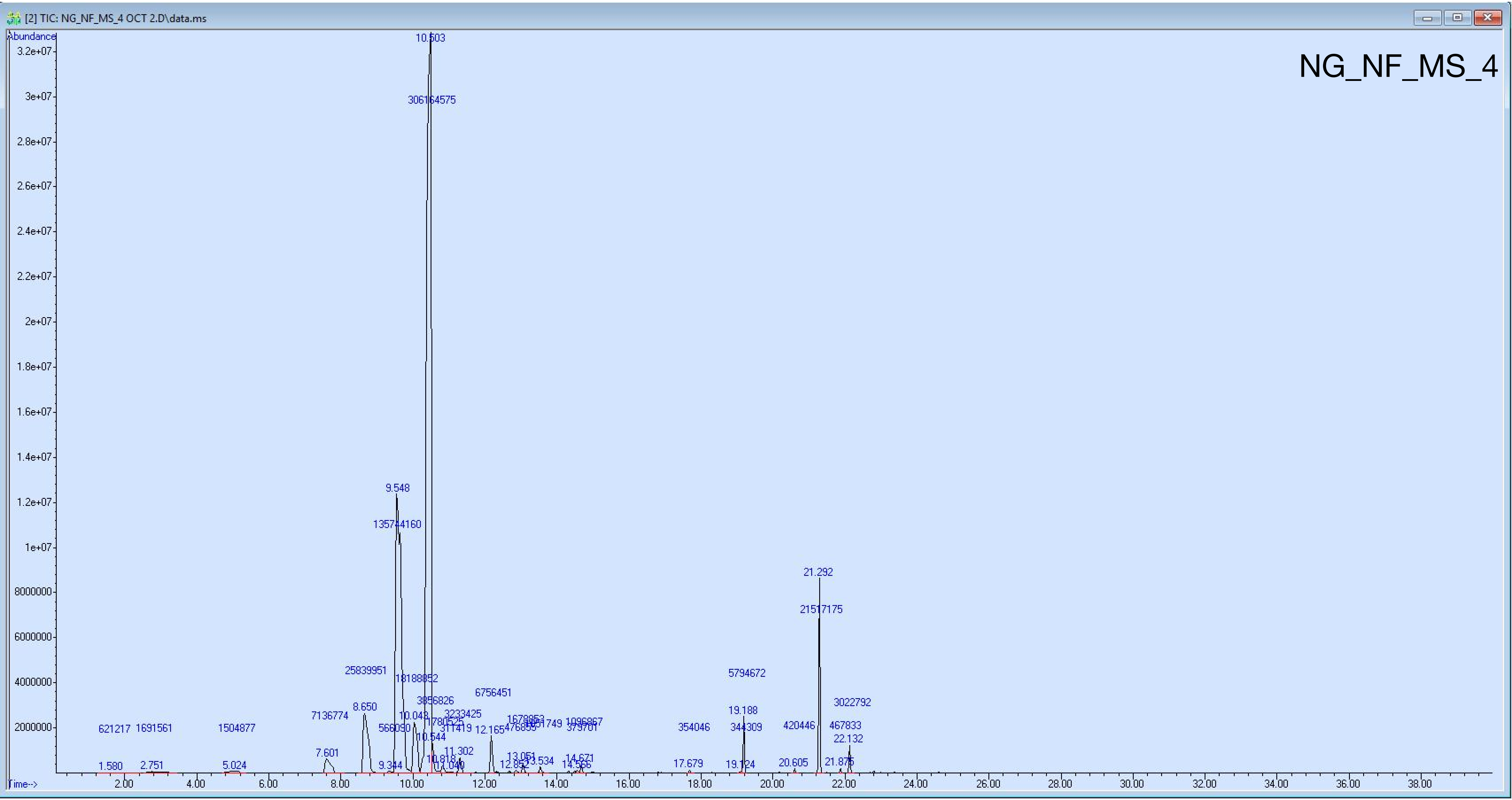
[2] TIC: NG_NF_MS_4 OCT 2.D\data.ms
NG_NF_MS_4
Abundance
Time-->
10.503
306164575
9.548
135744160
21.292
21517175
25839951
8.650
7136774
7.601
5794672
19.188
344309
3022792
467833
22.132
6756451
12.165
621217
1691561
1504877
1.580
2.751
5.024
354046
17.679
420446
20.605
19.124
21.875

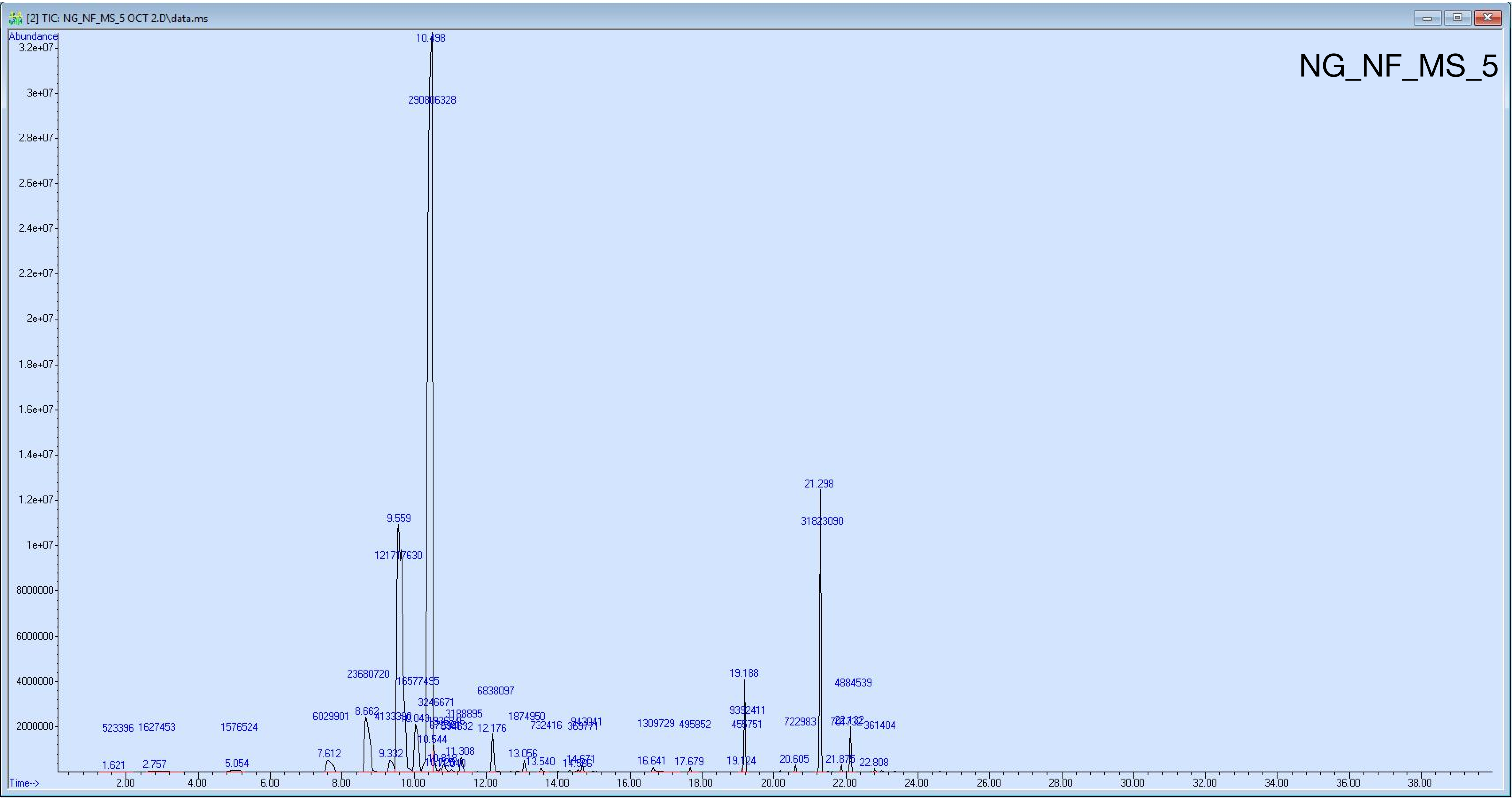
[2] TIC: NG_NF_MS_5 OCT 2.D\data.ms
NG_NF_MS_5
Abundance
Time-->
10.498
290806328
9.559
121717630
21.298
31823090
19.188
4884539
23680720
6838097
8.662
12.176
7.612
9.332
13.056
13.540
16.641
17.679
19.124
20.605
21.875
22.808
1.621
2.757
5.054
523396
1627453
1576524
6029901
3246671
3188895
1874950
732416
1309729
495852
9352411
455751
722983
361404
10.544
11.308
14.671

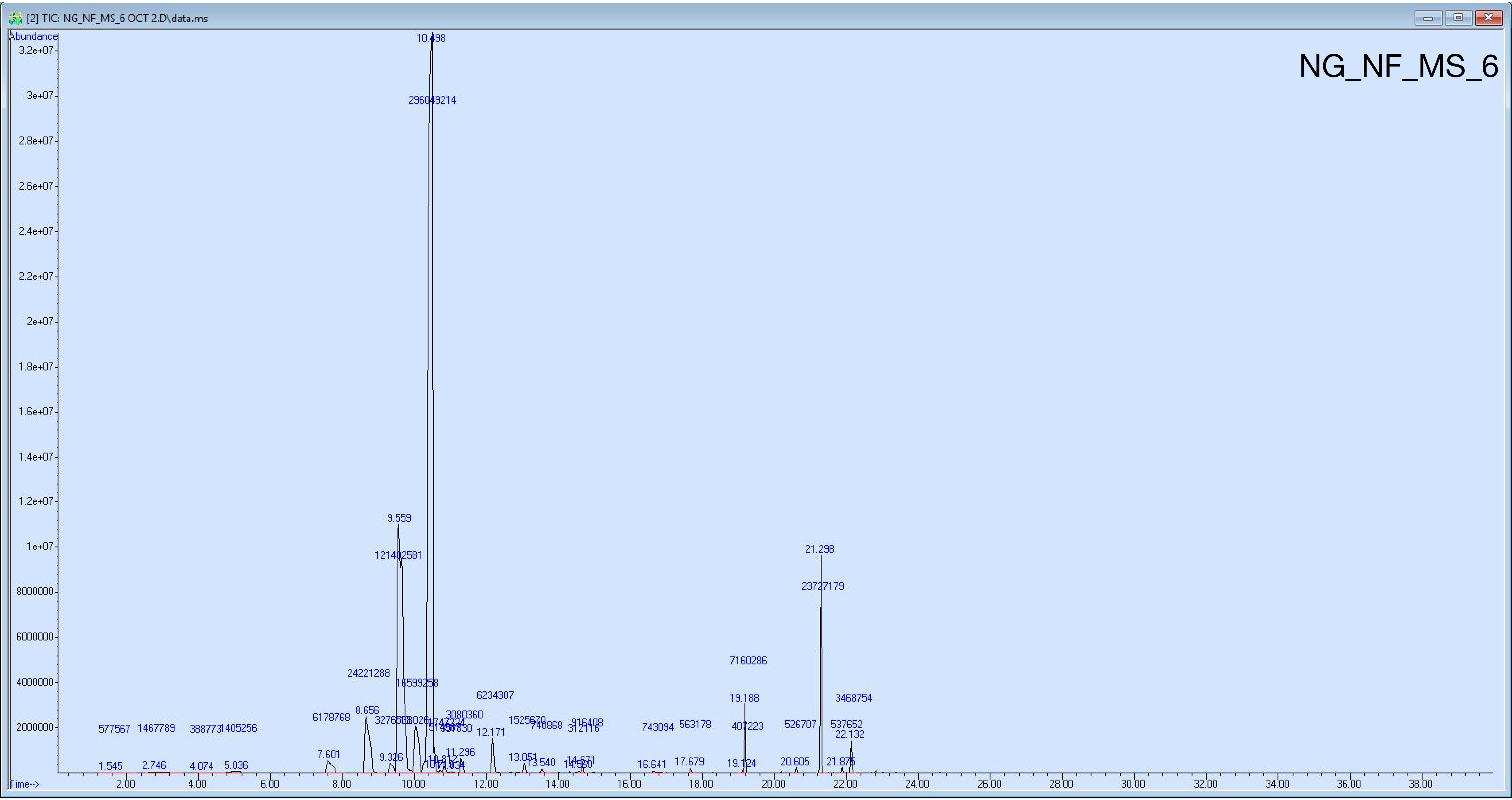
[2] TIC: NG_NF_MS_6 OCT 2.D\data.ms
NG_NF_MS_6
Abundance
Time-->

New Girl (hybrid)
Flooding
No Herbivory

[2] TIC: NG_FLD_1 OCT 4.D\data.ms

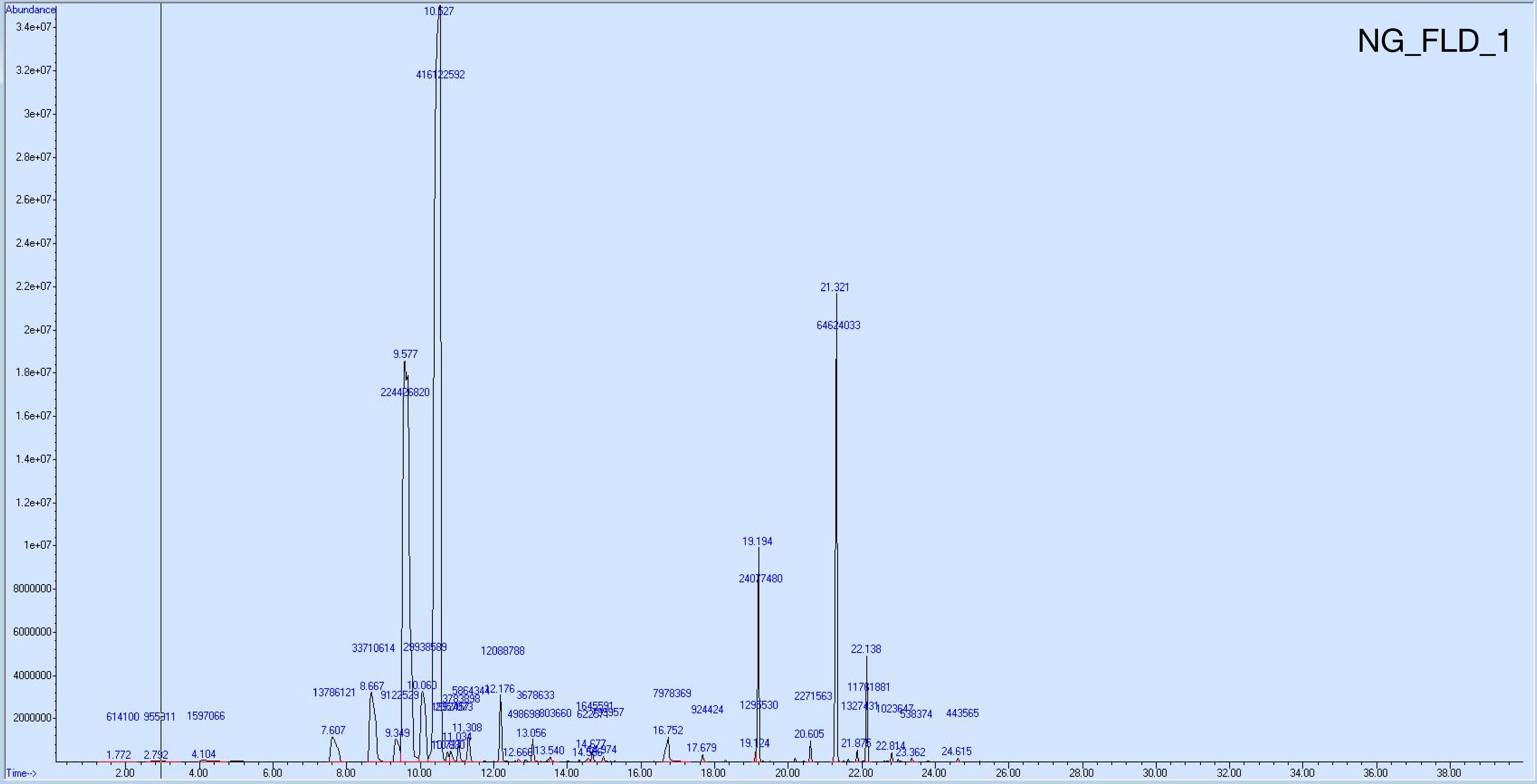

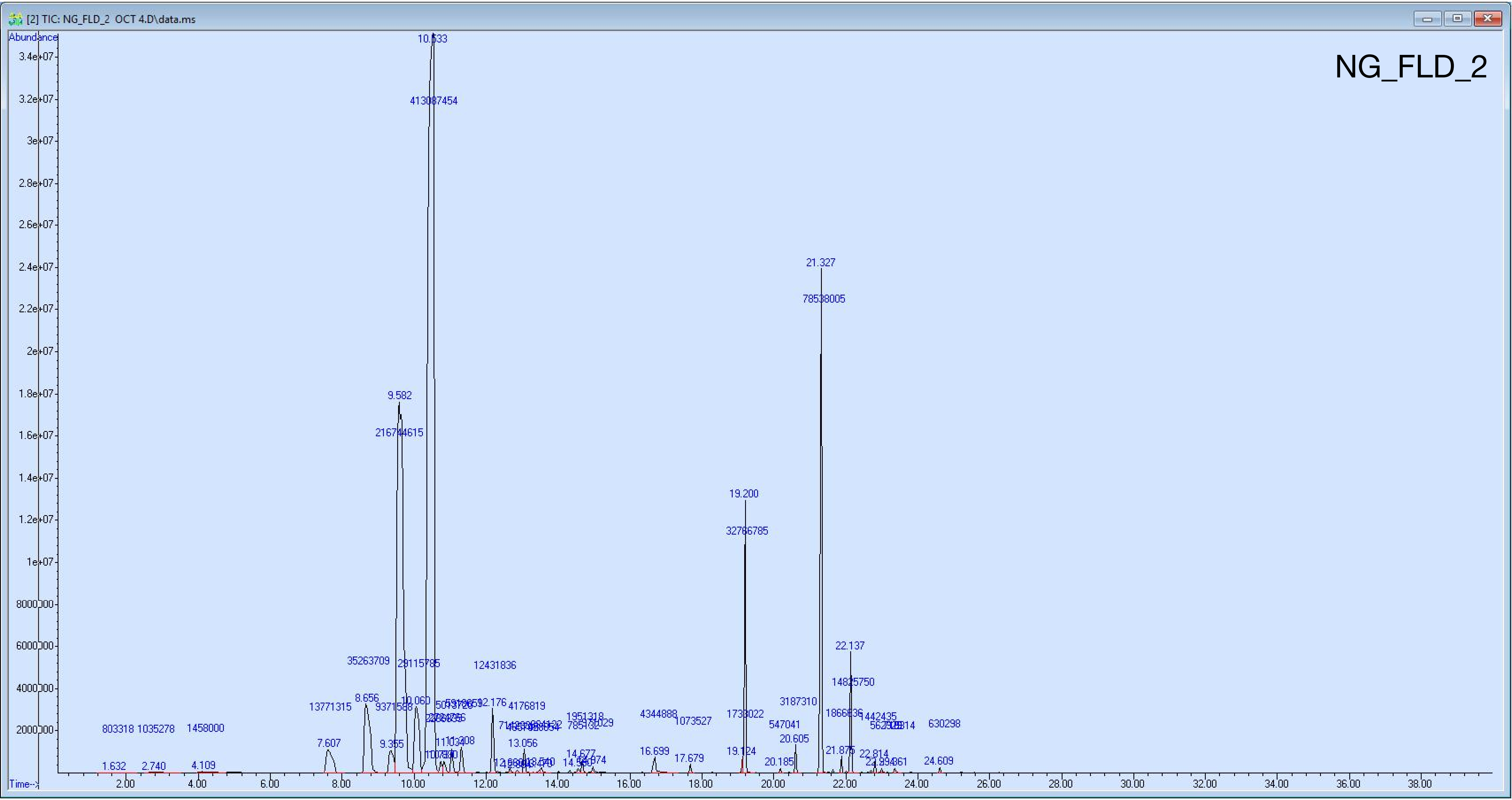
[2] TIC: NG_FLD_2 OCT 4.D\data.ms
NG_FLD_2
Abundance
Time-->
10.533
413087454
21.327
78538005
9.582
216744615
19.200
32766785
22.137
14825750
35263709
29115785
12431836
13771315
8.656
9371588
10.060
12.176
4176819
3187310
4344888
1073527
1733022
547041
1866636
1442435
630298
803318 1035278
1458000
7.607
9.355
13.056
16.699
17.679
19.124
20.605
20.185
21.875
22.814
24.609
1.632
2.740
4.109
3.4e+07
3.2e+07
3e+07
2.8e+07
2.6e+07
2.4e+07
2.2e+07
2e+07
1.8e+07
1.6e+07
1.4e+07
1.2e+07
1e+07
8000000
6000000
4000000
2000000
2.00
4.00
6.00
8.00
10.00
12.00
14.00
16.00
18.00
20.00
22.00
24.00
26.00
28.00
30.00
32.00
34.00
36.00
38.00

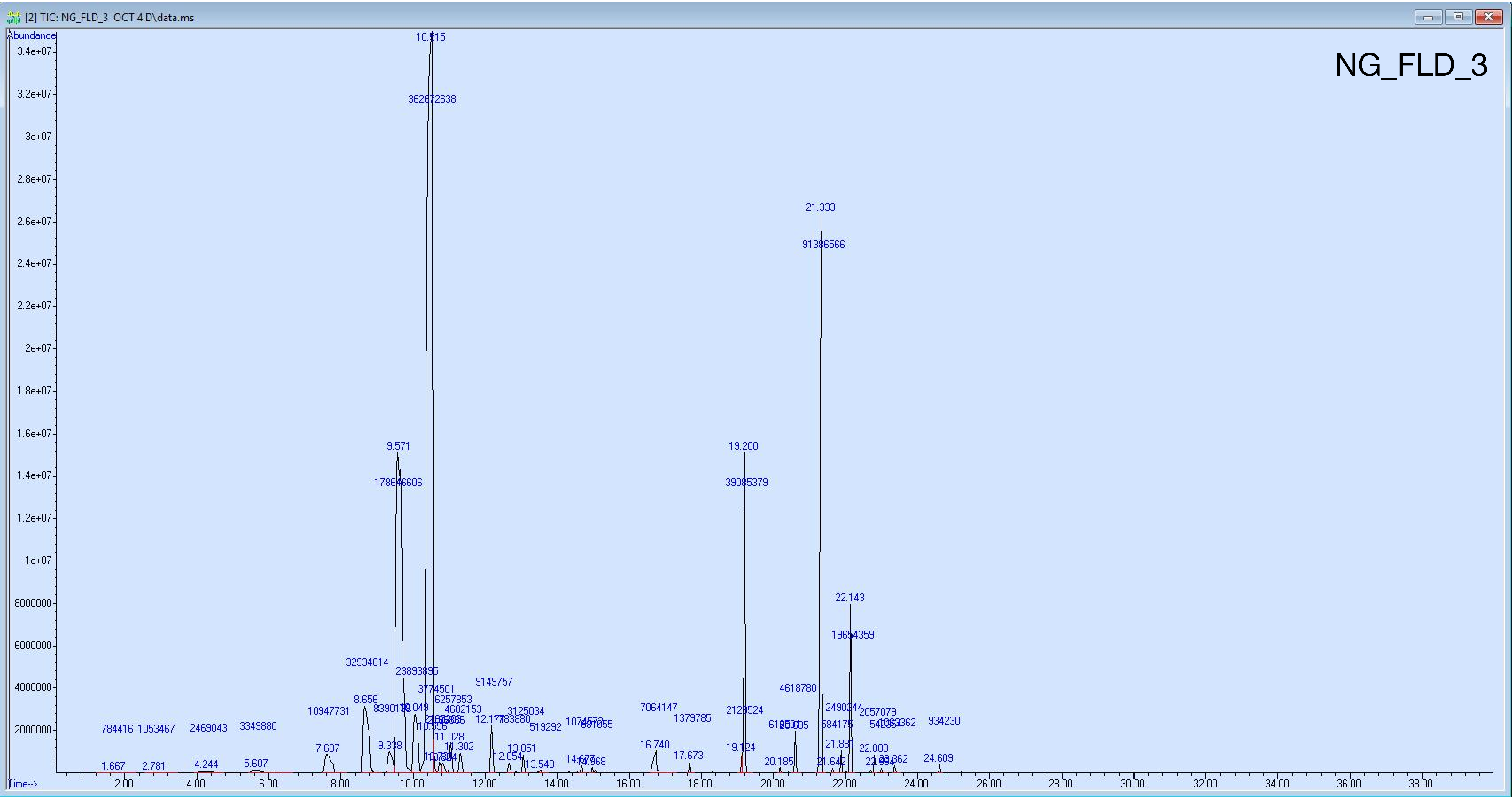
[2] TIC: NG_FLD_3 OCT 4.D\data.ms
NG_FLD_3
Abundance
Time-->
10.515
9.571
19.200
21.333
22.143
8.656
7.607
9.338
11.028
16.740
17.673
19.124
20.185
24.609
22.808
13.051
13.540
12.654
1.667
2.781
4.244
5.607
784416
1053467
2469043
3349880
10947731
32934814
9149757
7064147
1379785
4618780
19654359
39085379
934230
2057079
3125034
519292
3774501
6257853
4682153

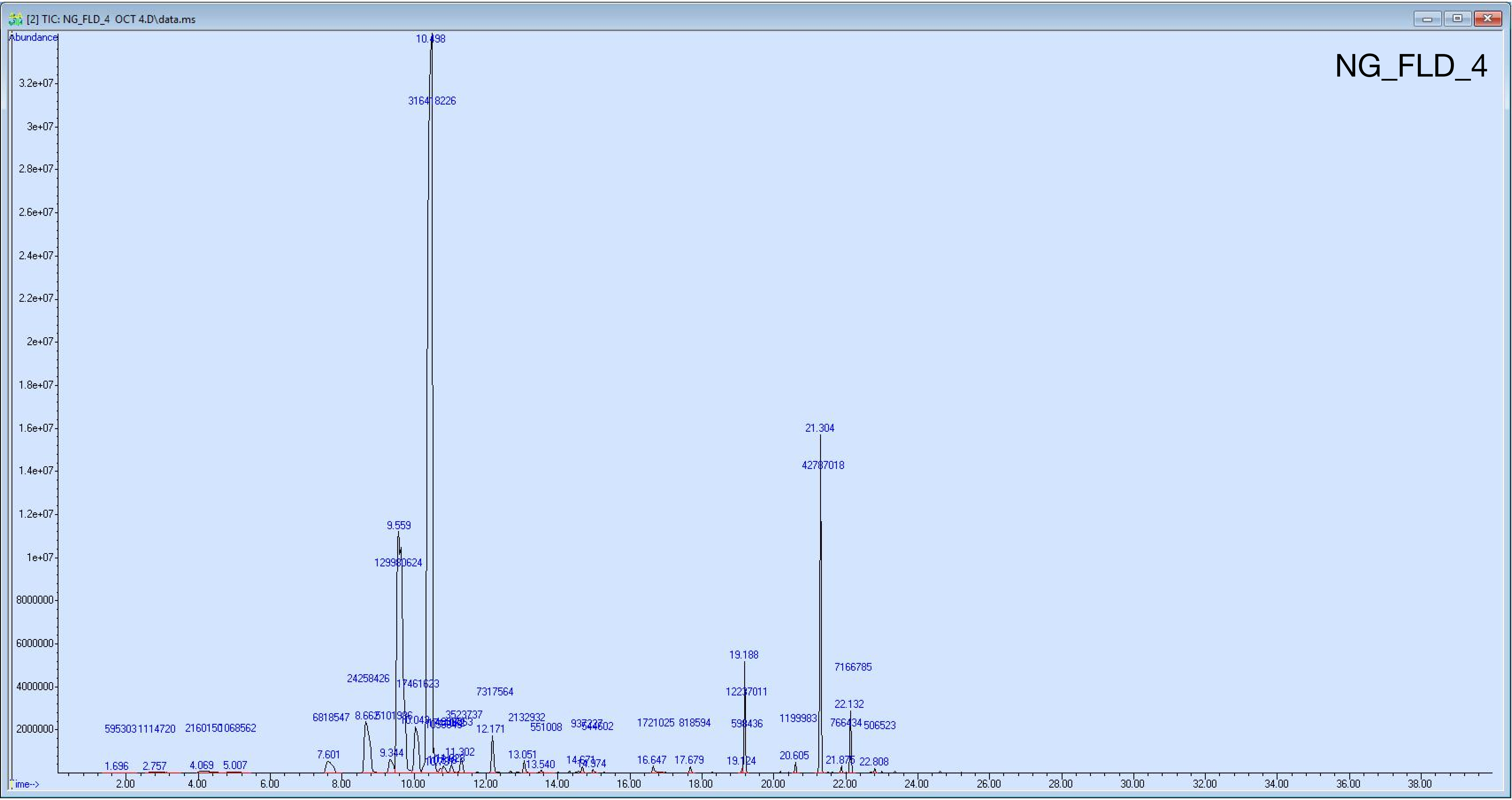
[2] TIC: NG_FLD_4 OCT 4.D\data.ms
NG_FLD_4
Abundance
Time-->
10.498
21.304
19.188
9.559
22.132
12.171

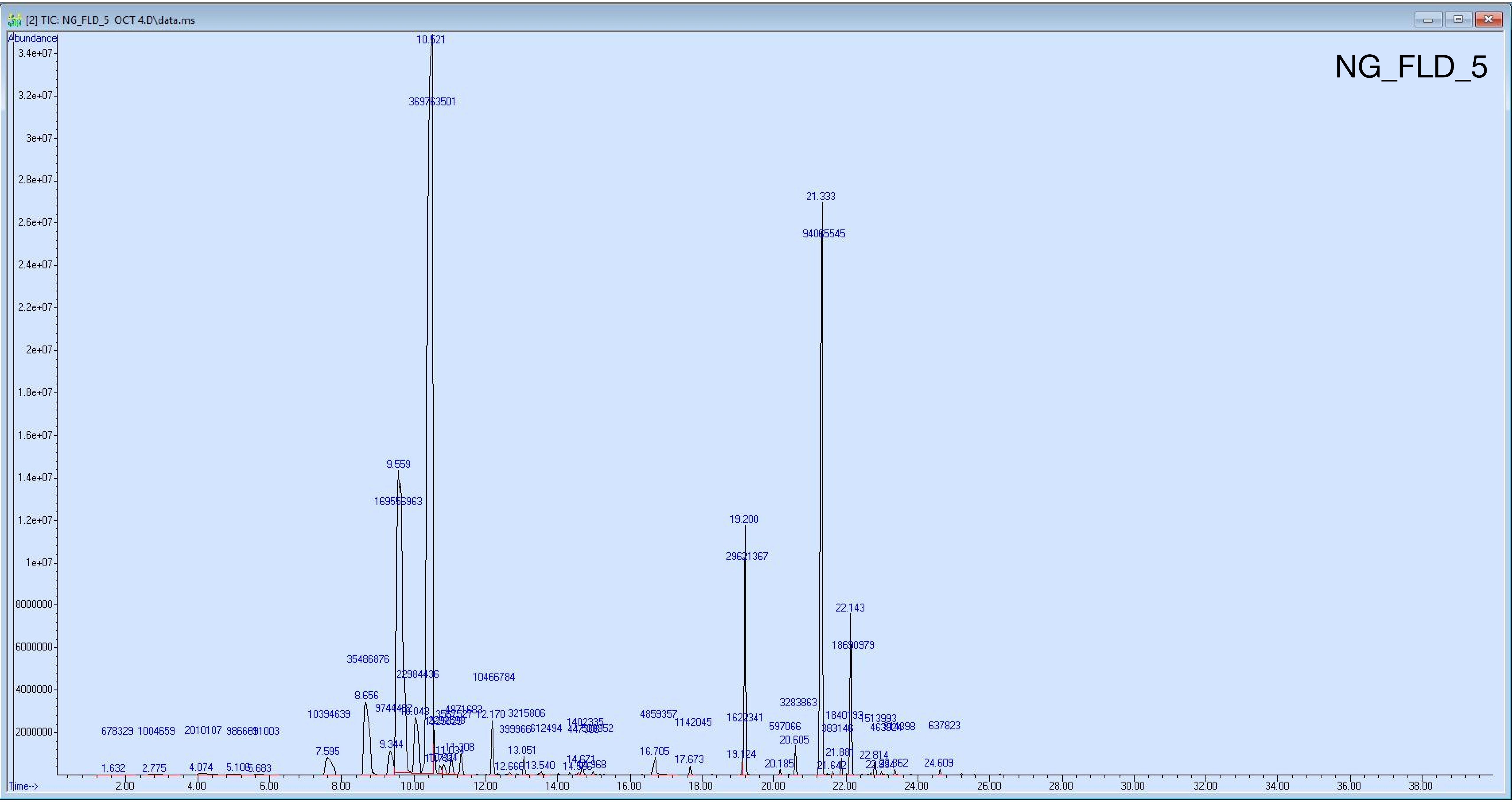

[2] TIC: NG_FLD_5 OCT 4.D\data.ms
NG_FLD_5
Abundance
3.4e+07
3.2e+07
3e+07
2.8e+07
2.6e+07
2.4e+07
2.2e+07
2e+07
1.8e+07
1.6e+07
1.4e+07
1.2e+07
1e+07
8000000
6000000
4000000
2000000
Time-->
2.00
4.00
6.00
8.00
10.00
12.00
14.00
16.00
18.00
20.00
22.00
24.00
26.00
28.00
30.00
32.00
34.00
36.00
38.00
21.333
94065545
9.559
19.200
29621367
22.143
35486876
10466784
8.656
10394639
7.595
9.344
678329
1004659
2010107
1.632
2.775
4.074
3215806
13.051
13.540
16.705
4859357
17.673
1142045
19.124
1622341
597066
20.605
20.185
3283863
22.814
24.609
637823

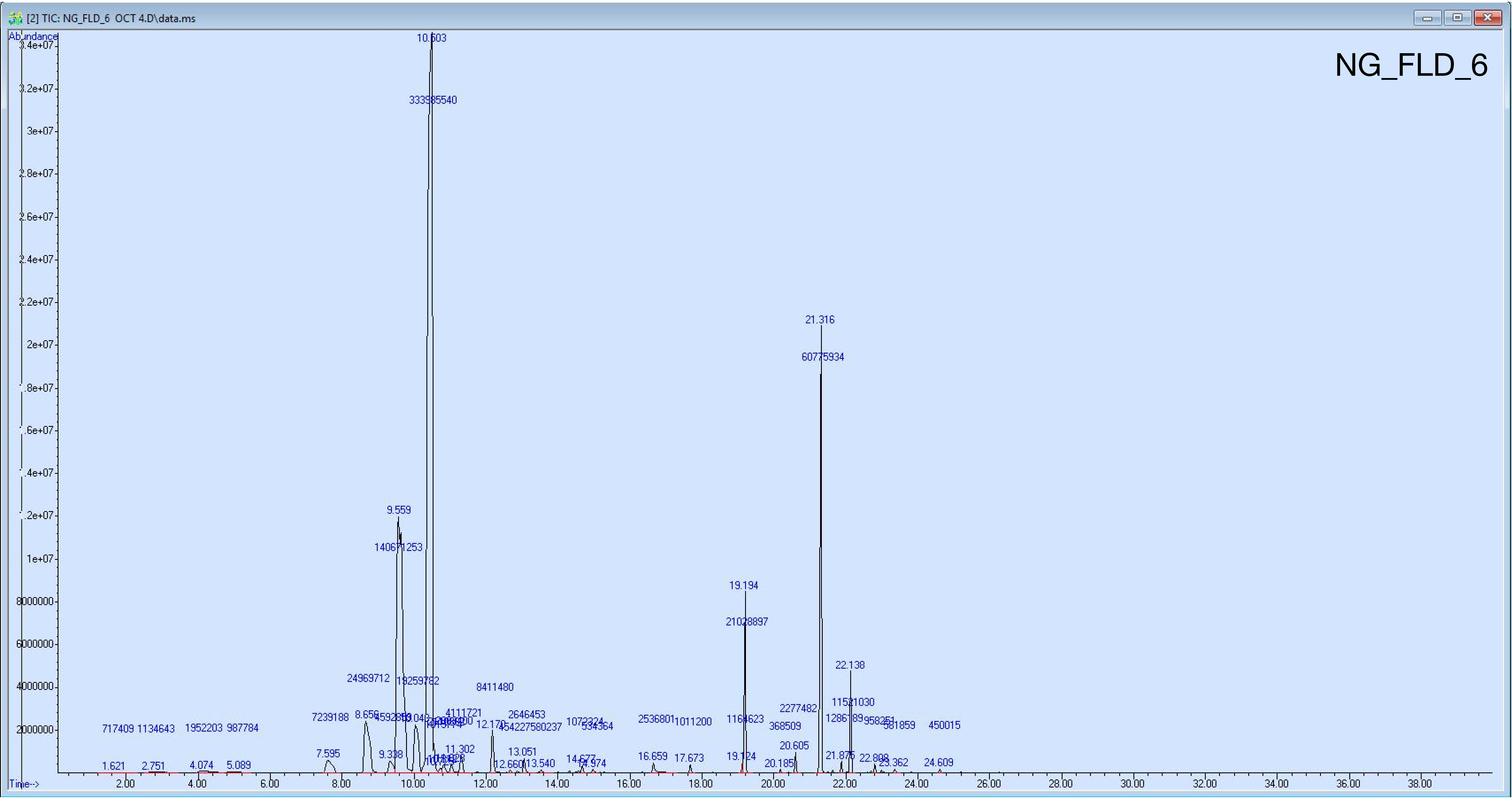
[2] TIC: NG_FLD_6 OCT 4.D\data.ms
NG_FLD_6
Abundance
Time-->

New Girl (hybrid)
Flooding
Spodptera exigua-damaged

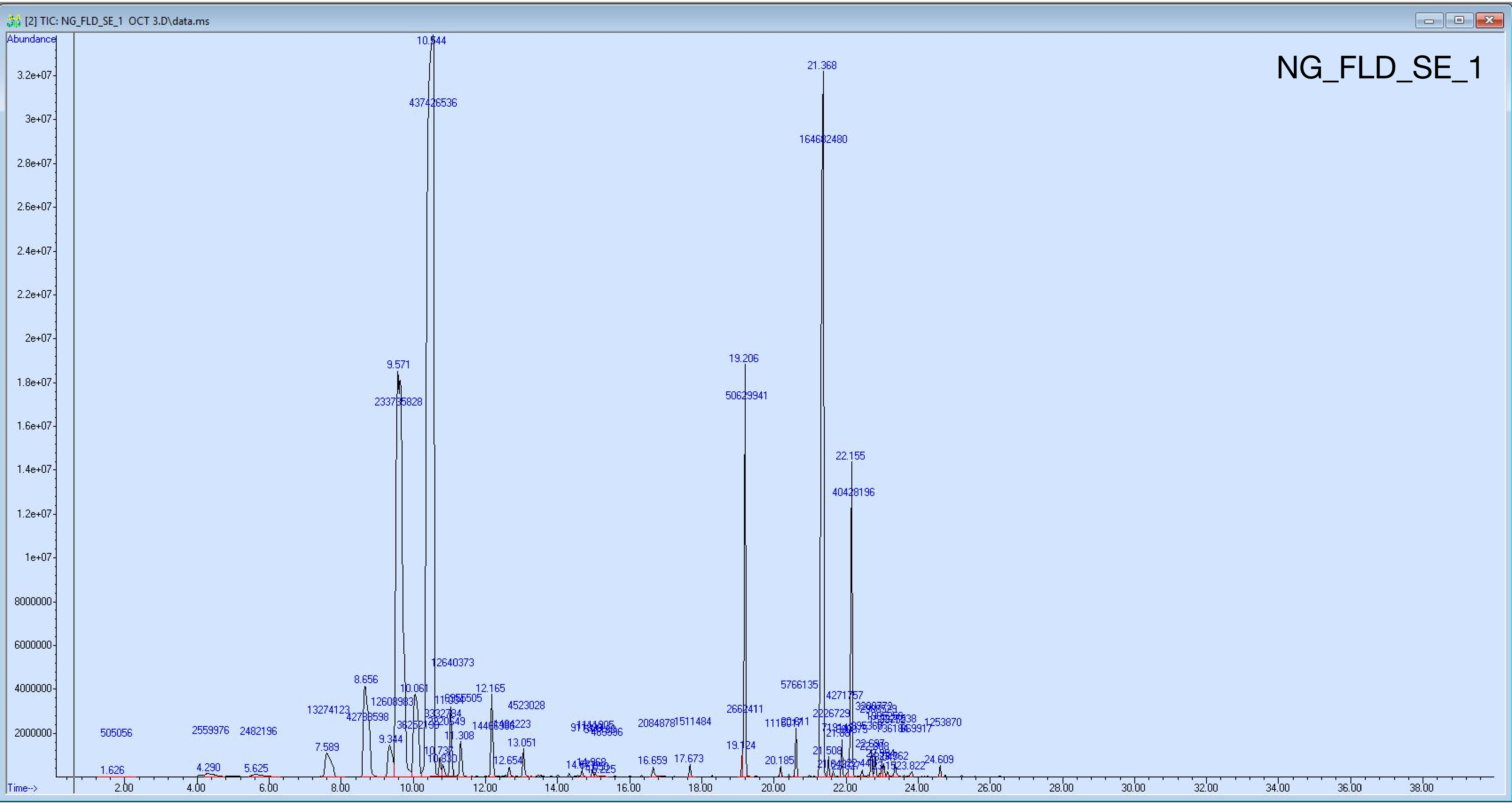
[2] TIC: NG_FLD_SE_1 OCT 3.D\data.ms
NG_FLD_SE_1
Abundance
3.2e+07
3e+07
2.8e+07
2.6e+07
2.4e+07
2.2e+07
2e+07
1.8e+07
1.6e+07
1.4e+07
1.2e+07
1e+07
8000000
6000000
4000000
2000000
Time-->
2.00
4.00
6.00
8.00
10.00
12.00
14.00
16.00
18.00
20.00
22.00
24.00
26.00
28.00
30.00
32.00
34.00
36.00
38.00
10.544
437426536
21.368
164682480
9.571
19.206
50629941
22.155
40428196
12640373
8.656
13274123
7.589
9.344
10.061
12.165
4523028
11.308
13.051
12.654
505056
1.626
2559976
4.290
2482196
5.625
2084878
16.659
1511484
17.673
2662411
19.124
20.185
5766135
4271757
2226729
1253870
24.609
23.822

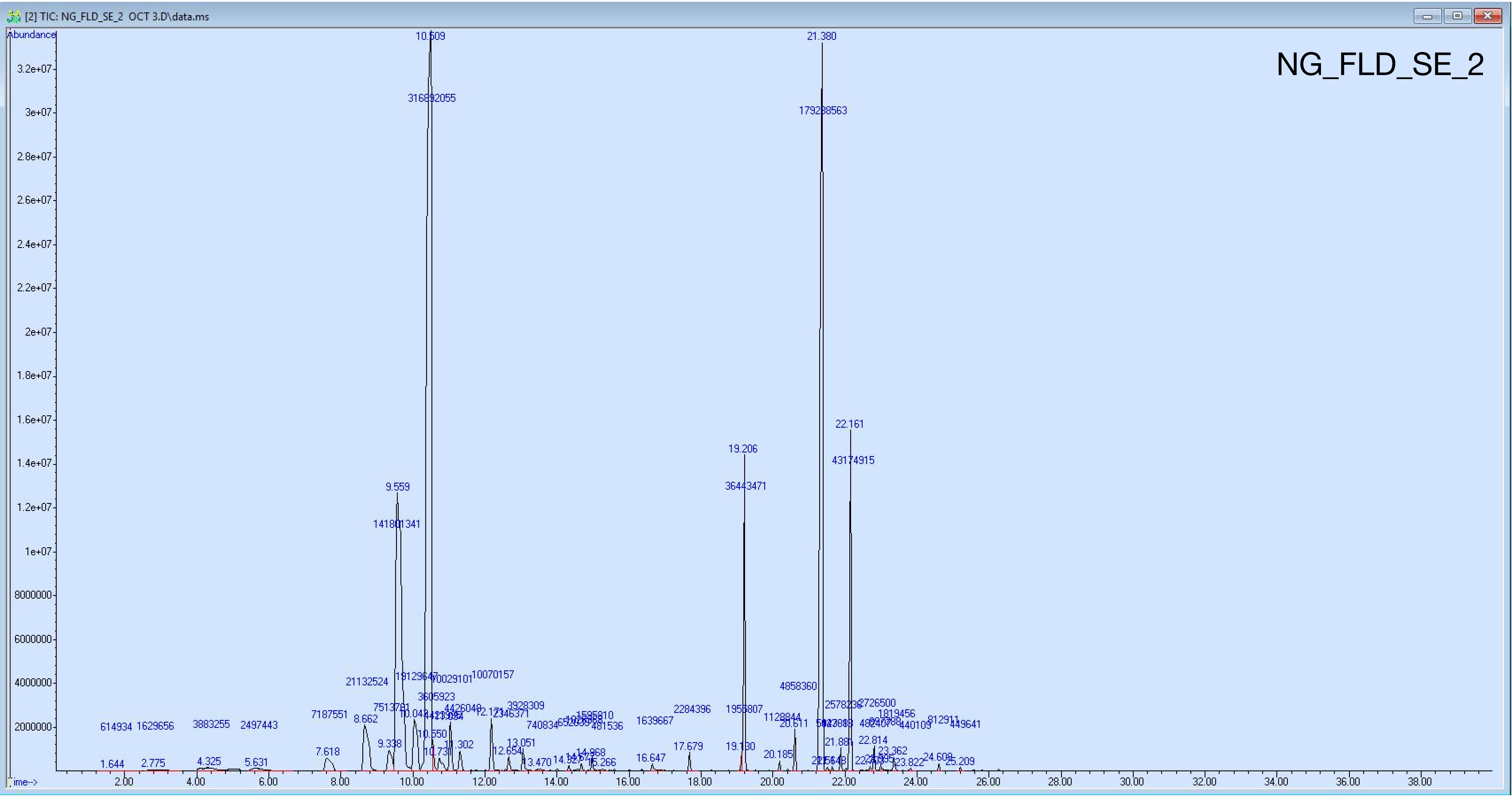
[2] TIC: NG_FLD_SE_2 OCT 3.D\data.ms
NG_FLD_SE_2
Abundance
Time-->
10.509
316892055
21.380
179238563
22.161
43174915
19.206
36443471
9.559
141801341

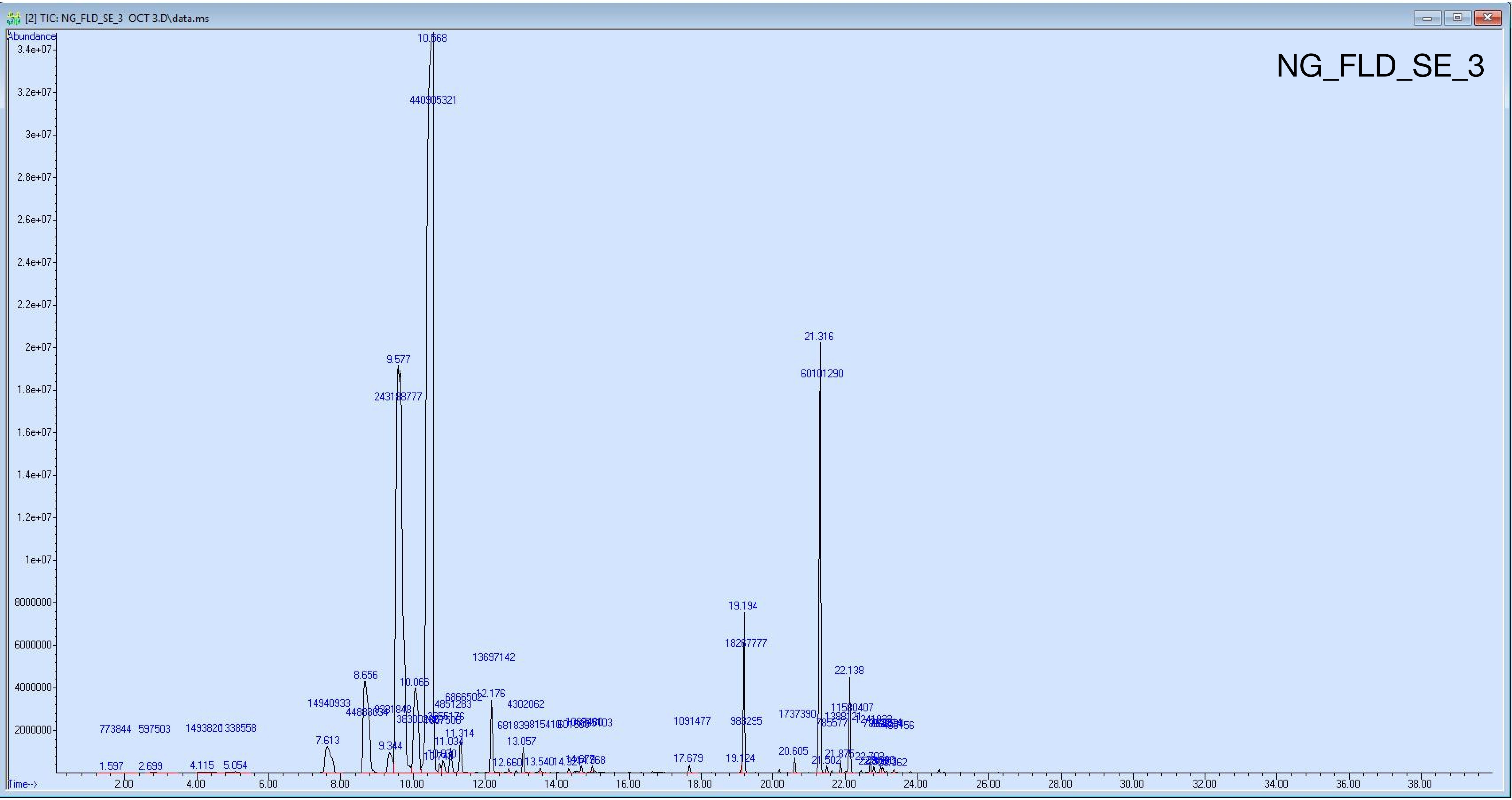
[2] TIC: NG_FLD_SE_3 OCT 3.D\data.ms
NG_FLD_SE_3
Abundance
Time-->

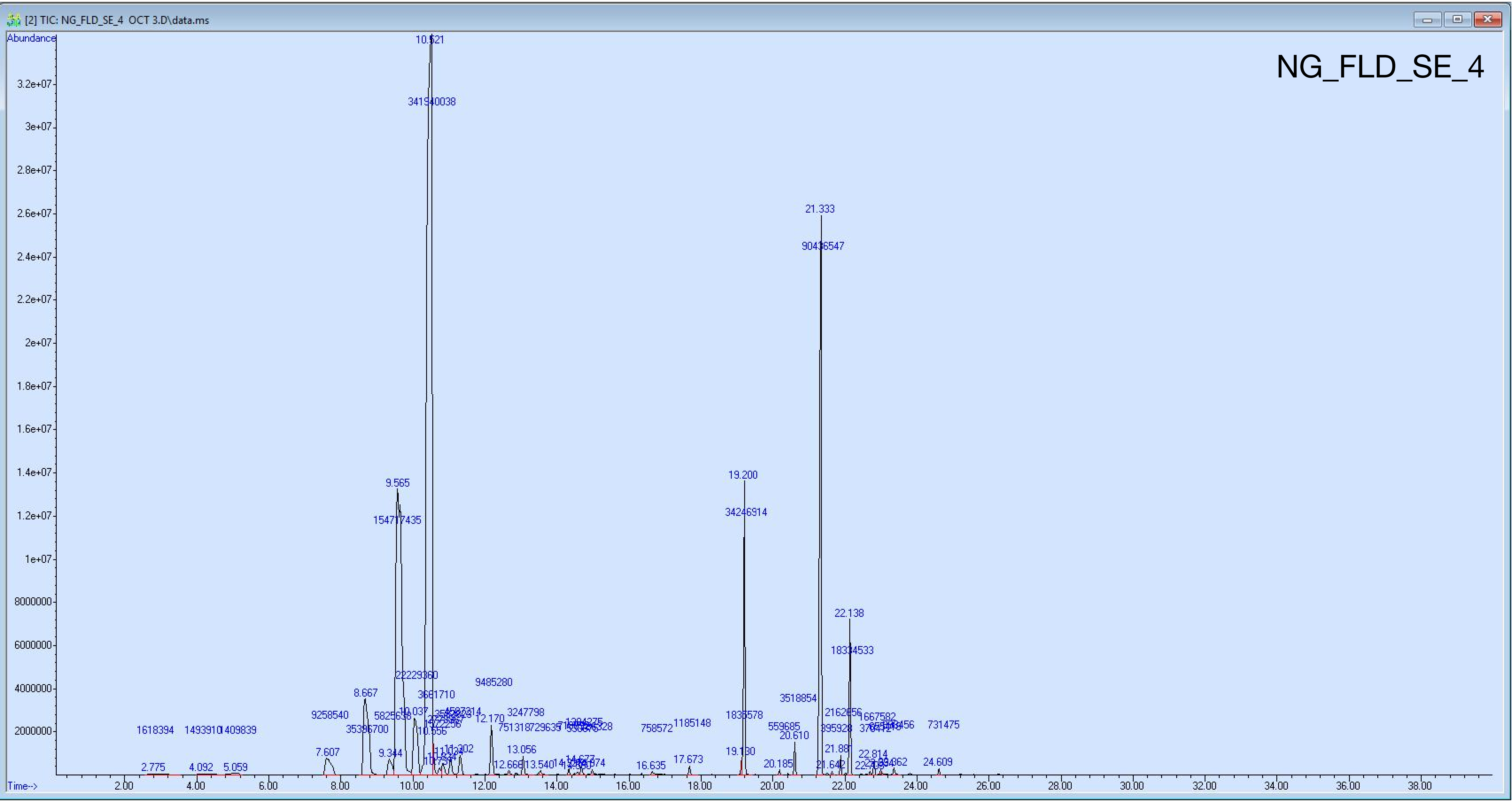
[2] TIC: NG_FLD_SE_4 OCT 3.D\data.ms
Abundance
NG_FLD_SE_4
3.2e+07
3e+07
2.8e+07
2.6e+07
2.4e+07
2.2e+07
2e+07
1.8e+07
1.6e+07
1.4e+07
1.2e+07
1e+07
8000000
6000000
4000000
2000000
Time-->
2.00
4.00
6.00
8.00
10.00
12.00
14.00
16.00
18.00
20.00
22.00
24.00
26.00
28.00
30.00
32.00
34.00
36.00
38.00
9.565
19.200
21.333
22.138
8.667
7.607
9.344
12.170
13.056
16.635
17.673
20.185
20.610
22.814
24.609
2.775
4.092
5.059
1618394
1493910
1409839
9258540
9485280
3247798
758572
1185148
3518854
559685
731475

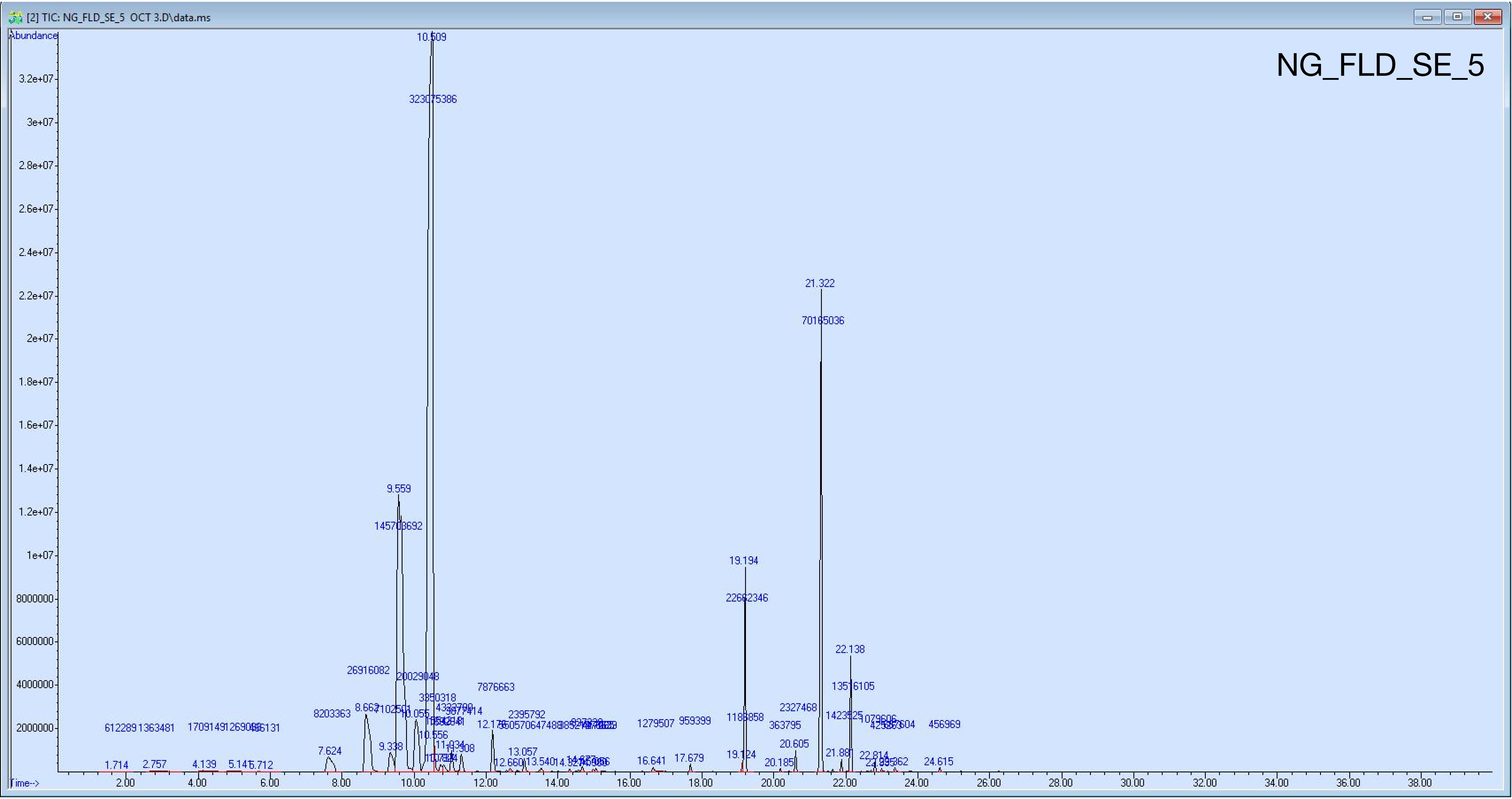
[2] TIC: NG_FLD_SE_5 OCT 3.D\data.ms
NG_FLD_SE_5
Abundance
Time-->
10.509
323075386
21.322
70165036
9.559
145703692
19.194
22662346
22.138
13516105

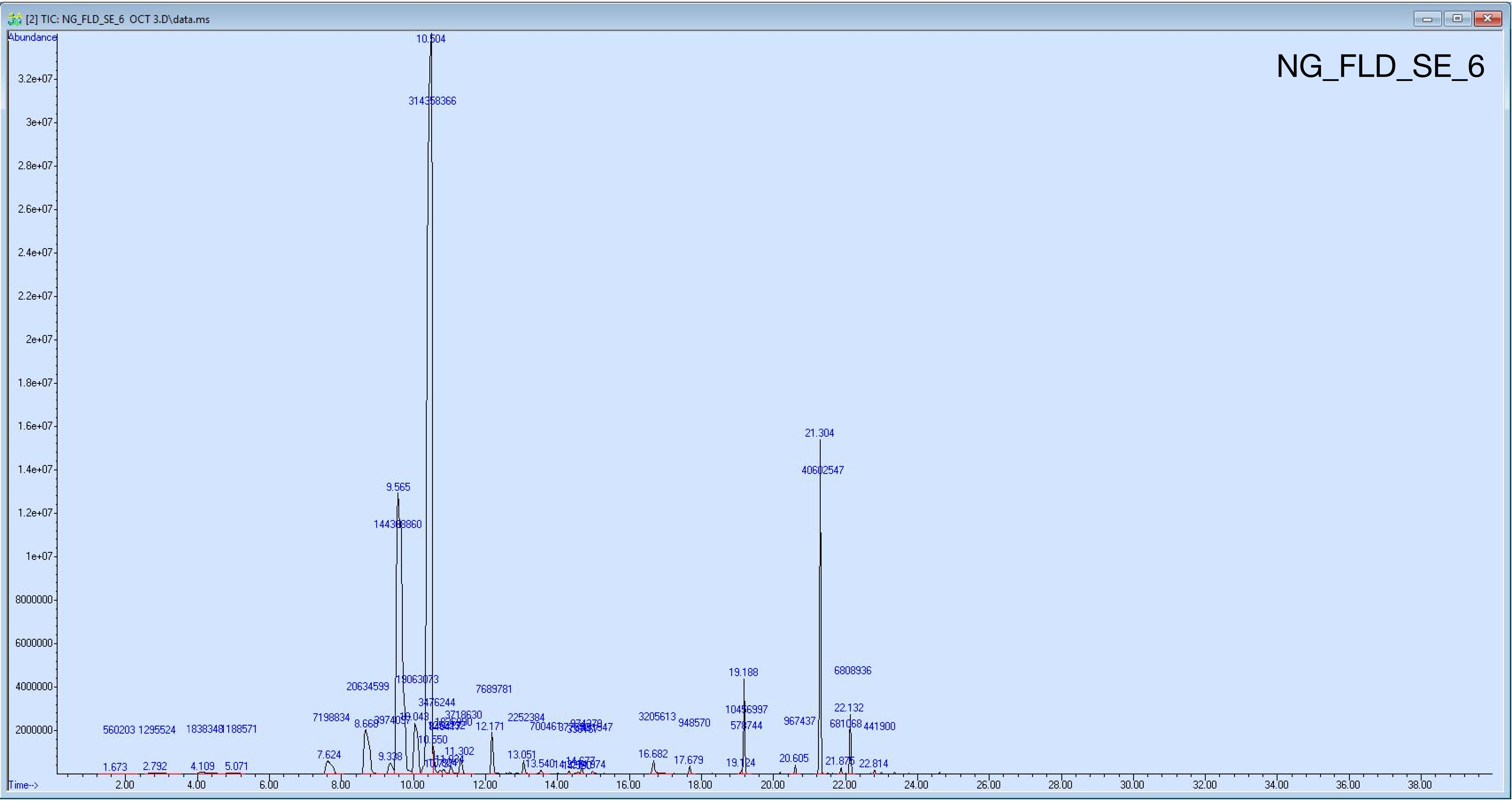
[2] TIC: NG_FLD_SE_6 OCT 3.D\data.ms
NG_FLD_SE_6
Abundance
Time-->
10.504
314358366
9.565
21.304
40602547
19.188
10456997
22.132
6808936
20634599
7198834
7689781
3205613
948570
967437
2252384
441900

New Girl (hybrid)
Flooding
Manduca sexta-damaged

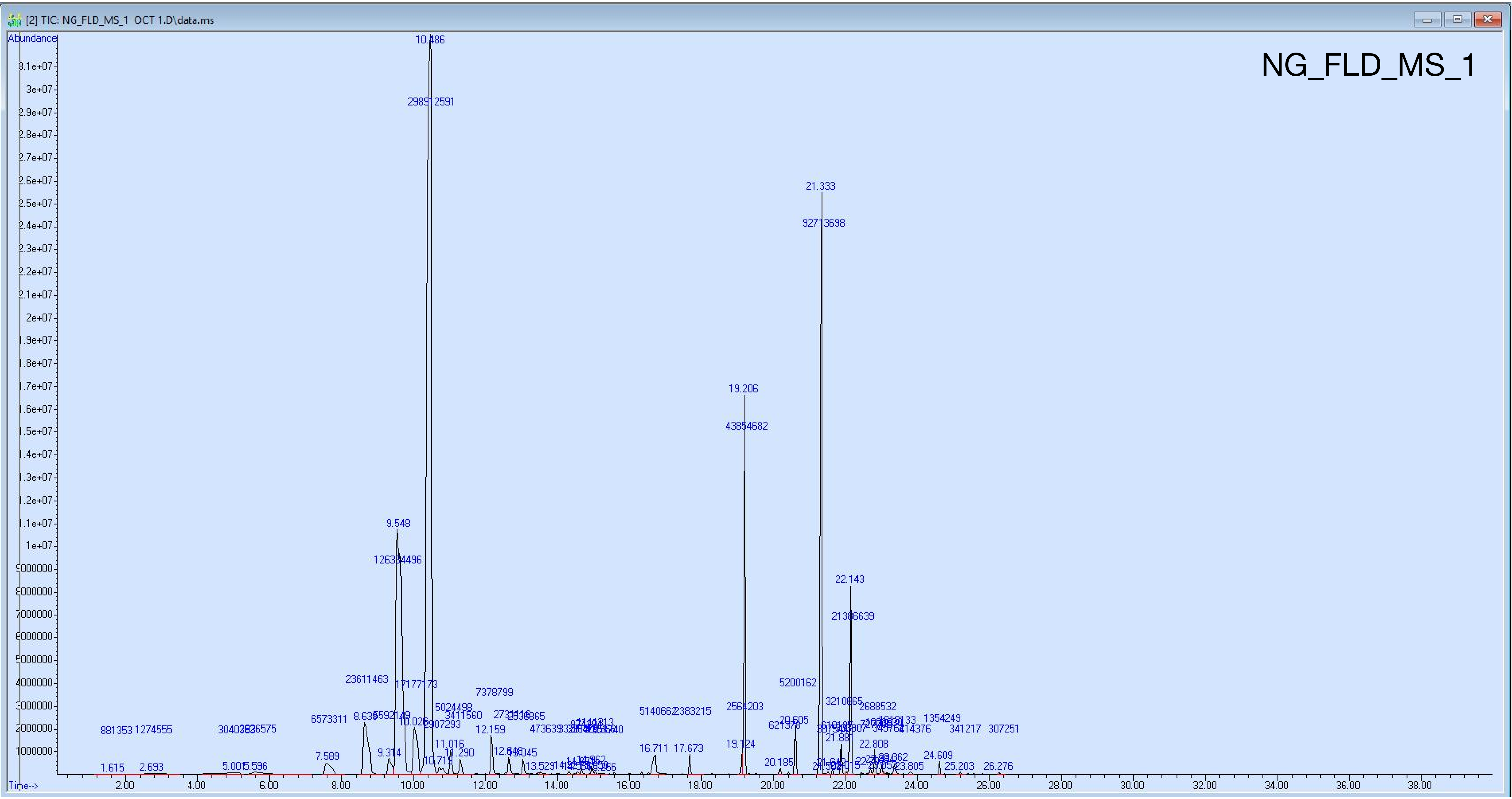

[2] TIC: NG_FLD_MS_1 OCT 1.D\data.ms
NG_FLD_MS_1
Abundance
Time-->
10.486
21.333
92713698
19.206
43854682
9.548
22.143
21386639
23611463
7378799
5024498
3411560
5140662
2383215
2564203
5200162
3210665
2688532
1354249
341217
307251
881353
1274555
6573311
8.635
7.589
9.314
12.159
16.711
17.673
19.124
20.185
20.605
621378
22.808
24.609
25.203
26.276
1.615
2.693
5.001
5.596
11.016
11.290
13.529

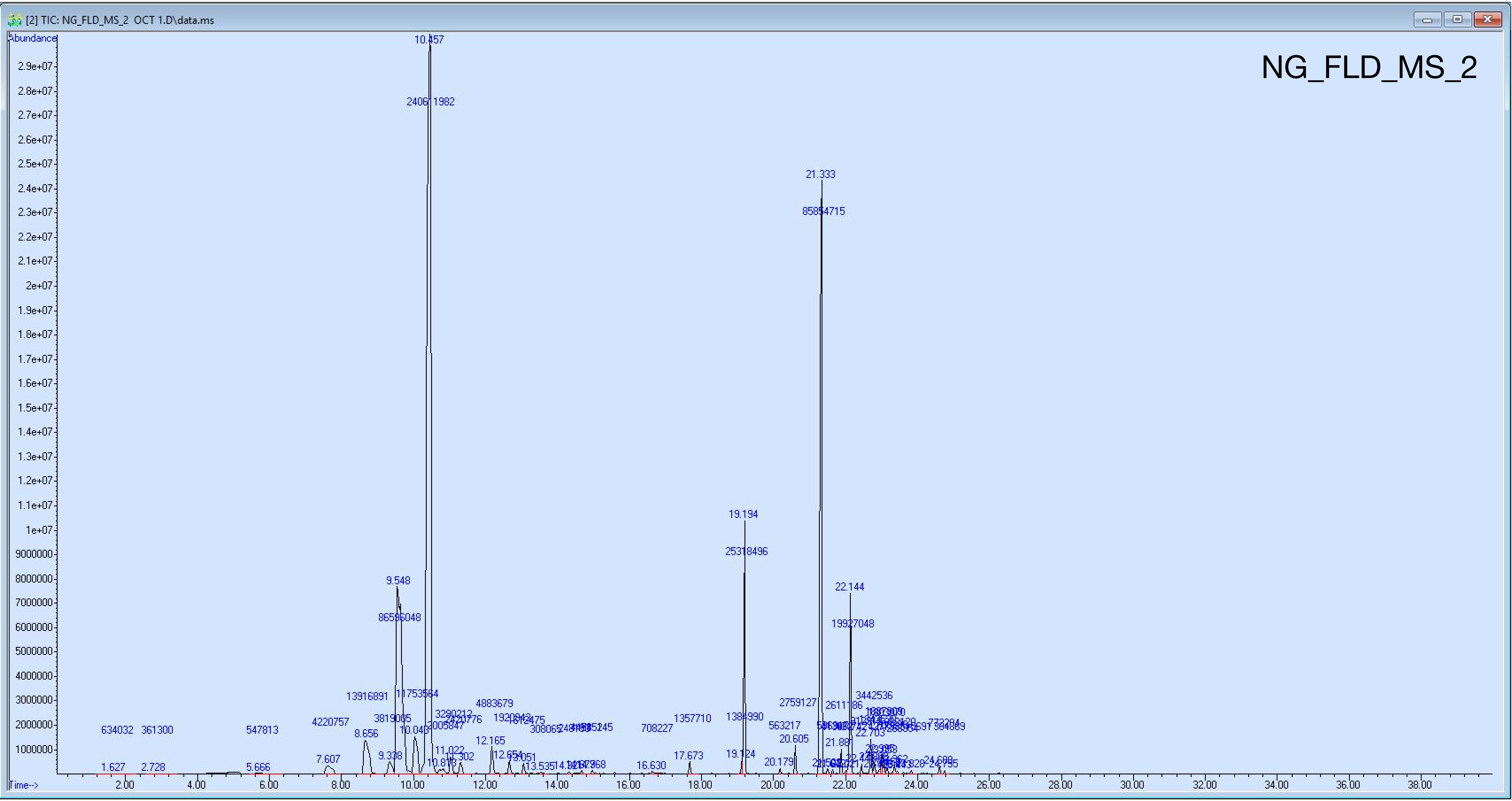
[2] TIC: NG_FLD_MS_2 OCT 1.D\data.ms
NG_FLD_MS_2
Abundance
Time-->
10.457
21.333
19.194
9.548
22.144
8.656
7.607
12.165
17.673
20.605
16.630
5.666
2.728
1.627
13916891
4220757
634032
361300
547813
4883679
708227
1357710
2759127
563217
3442536
25318496
19927048

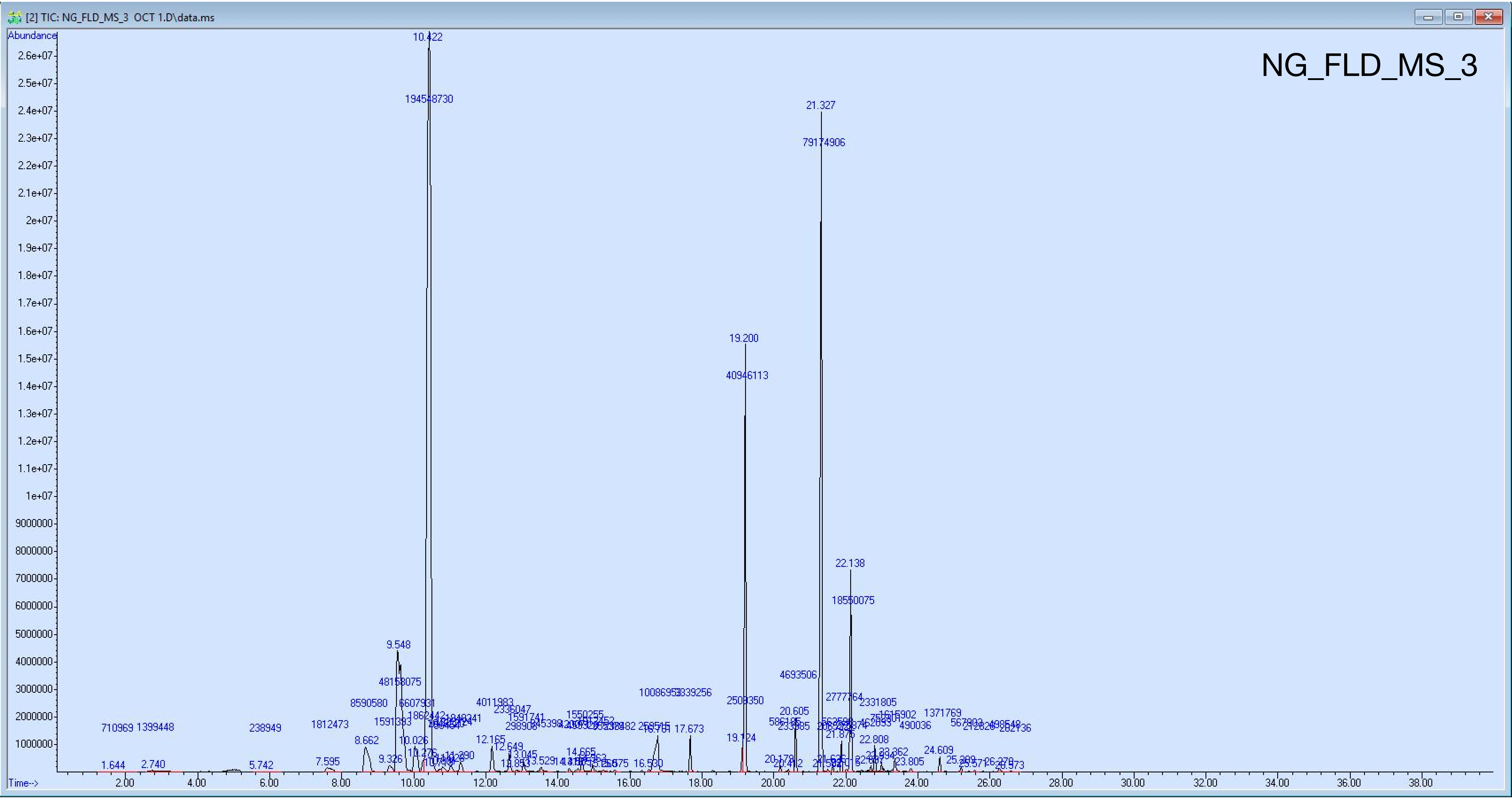
[2] TIC: NG_FLD_MS_3 OCT 1.D\data.ms
NG_FLD_MS_3
Abundance
Time-->
10.422
194548730
21.327
79174906
19.200
40946113
22.138
18550075
9.548
48158075
4693506
2777764
2331805
8590580
1591393
710969
1399448
238949
1812473
4011983
2336047
1008695
2509350
20.605
19.124
21.875
22.808
24.609
1371769
490036
8.662
10.026
12.165
12.649
14.665
16.530
17.673
1.644
2.740
5.742
7.595
9.326
23.805
2.6e+07
2.5e+07
2.4e+07
2.3e+07
2.2e+07
2.1e+07
2e+07
1.9e+07
1.8e+07
1.7e+07
1.6e+07
1.5e+07
1.4e+07
1.3e+07
1.2e+07
1.1e+07
1e+07
9000000
8000000
7000000
6000000
5000000
4000000
3000000
2000000
1000000
2.00
4.00
6.00
8.00
10.00
12.00
14.00
16.00
18.00
20.00
22.00
24.00
26.00
28.00
30.00
32.00
34.00
36.00
38.00

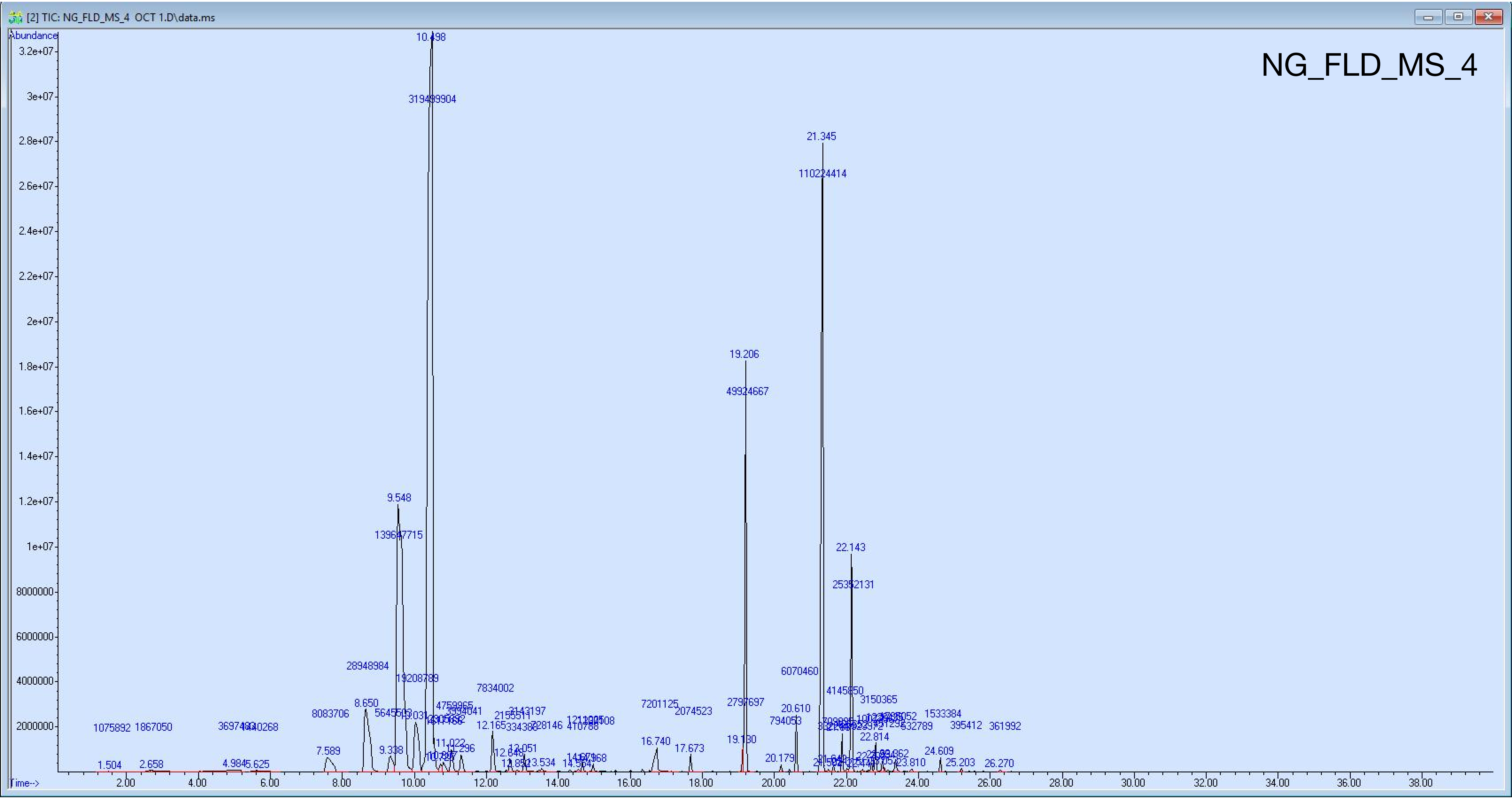

[2] TIC: NG_FLD_MS_4 OCT 1.D\data.ms
NG_FLD_MS_4
Abundance
Time-->
10.498
319499904
21.345
110224414
19.206
49924667
9.548
139647715
22.143
25352131

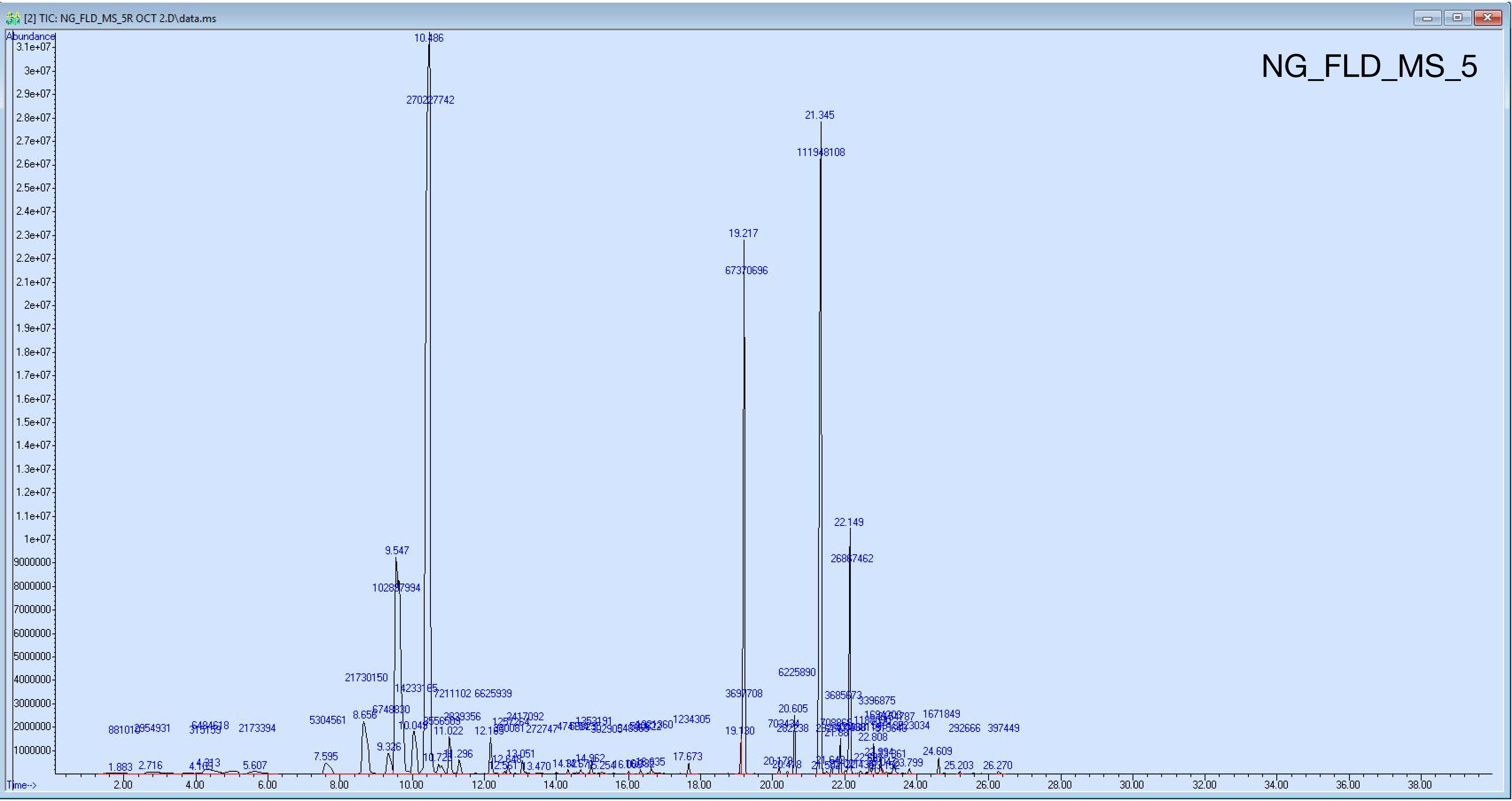
[2] TIC: NG_FLD_MS_5R OCT 2.D\data.ms
NG_FLD_MS_5
Abundance
Time-->
10.486
270227742
21.345
111948108
19.217
67370696
22.149
26887462
9.547
6225890
3697708
21730150
5304561
8.656
7211102 6625939
1234305
1671849
292666
397449
20.605
19.130
17.673
22.808
24.609
25.203
26.270
7.595
9.326
11.022
12.189
5.607
2173394
1.883
2.716
13.051
13.470
23.799
10.048
2839356
2417092
3396875

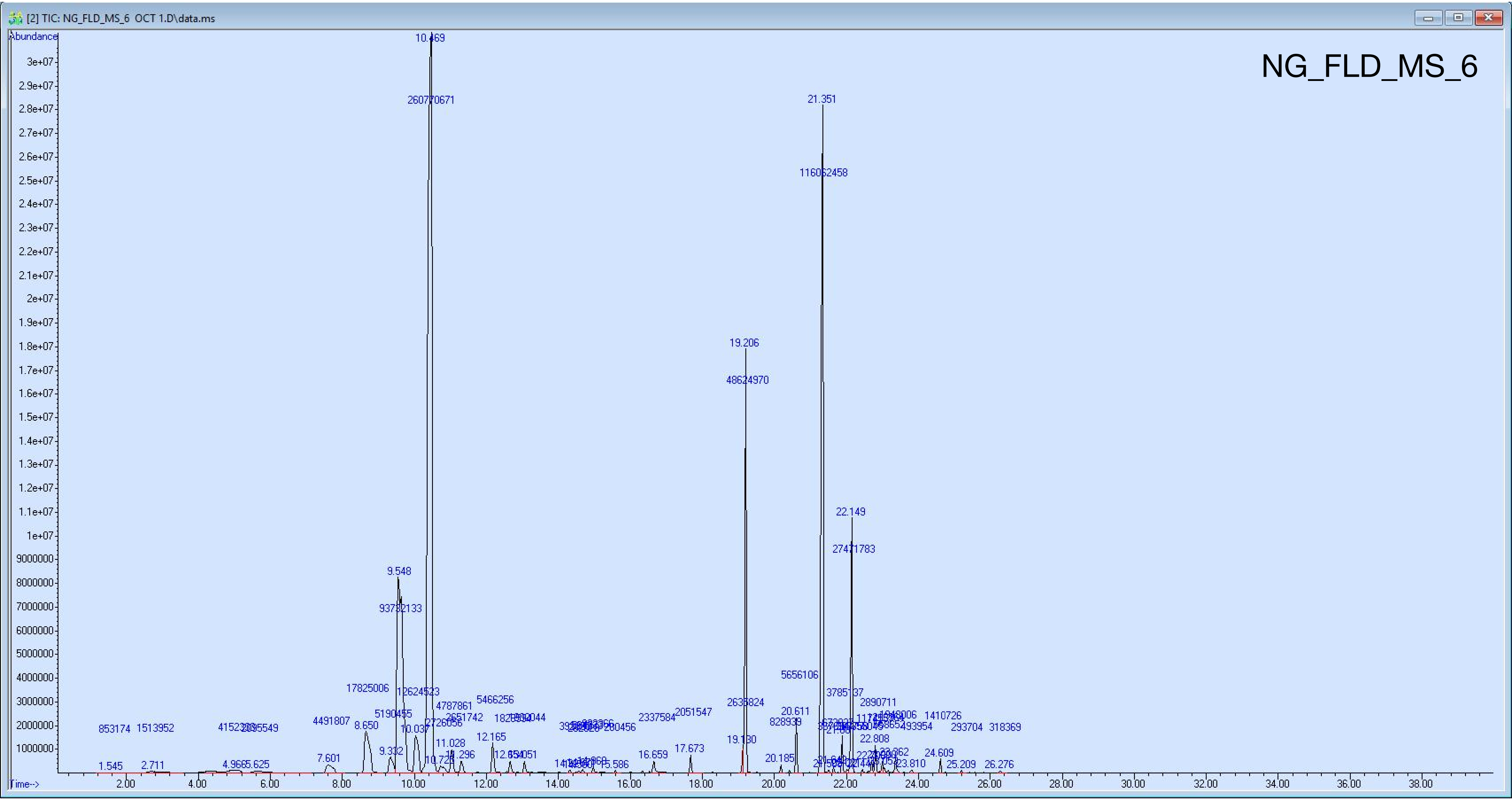
[2] TIC: NG_FLD_MS_6 OCT 1.D\data.ms
NG_FLD_MS_6
Abundance
Time-->
10.469
260770671
21.351
116062458
19.206
48624970
22.149
27471783
9.548
93732133

Mass Spectrums of major compounds identified

Mass Spectrum of **4-Carene** retrieved from CP_FLD_MS_1 at retention time 9.612 min

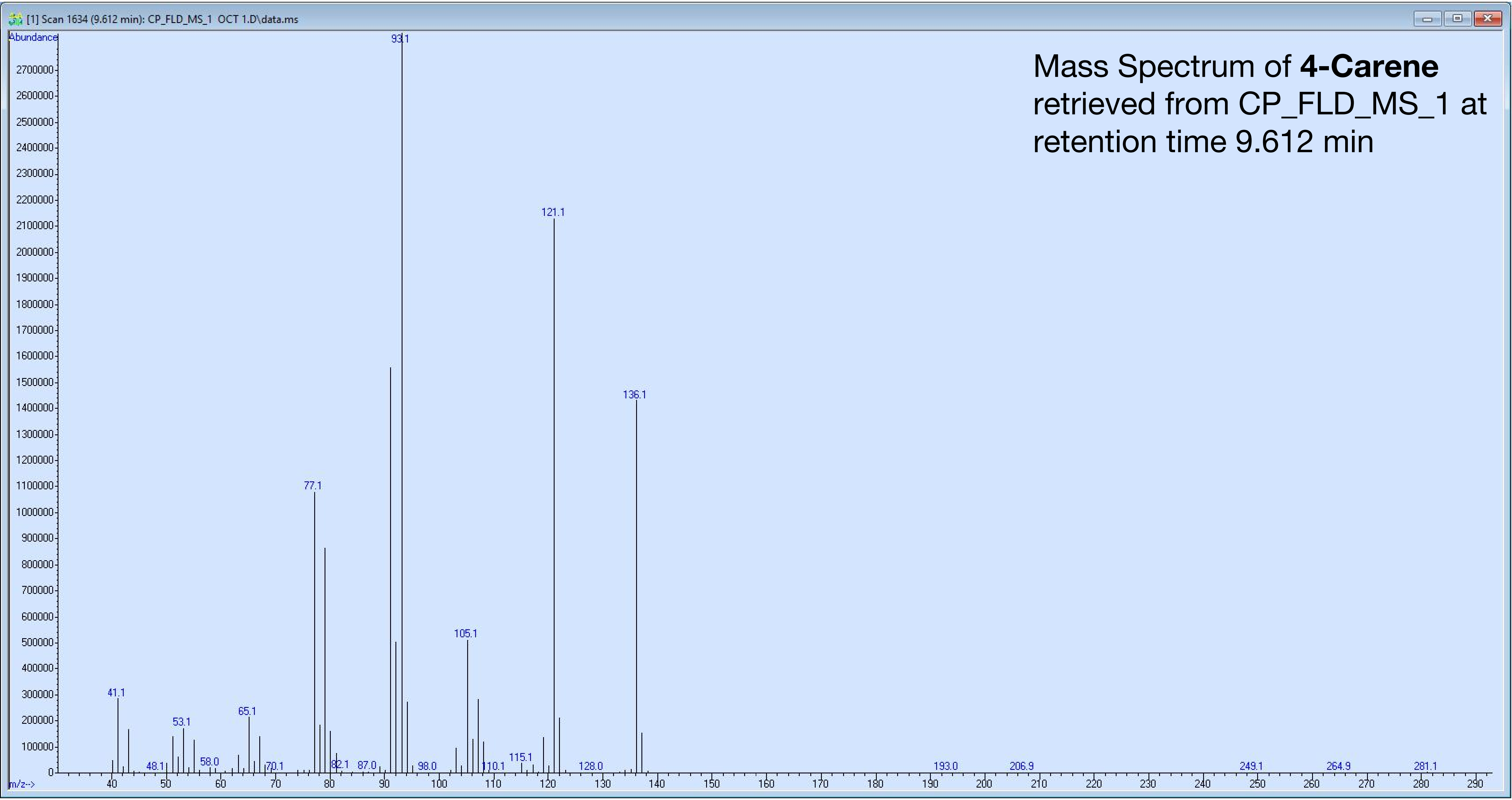

Mass Spectrum of **α-Pinene** retrieved from CP_FLD_MS_1 at retention time 7.461 min

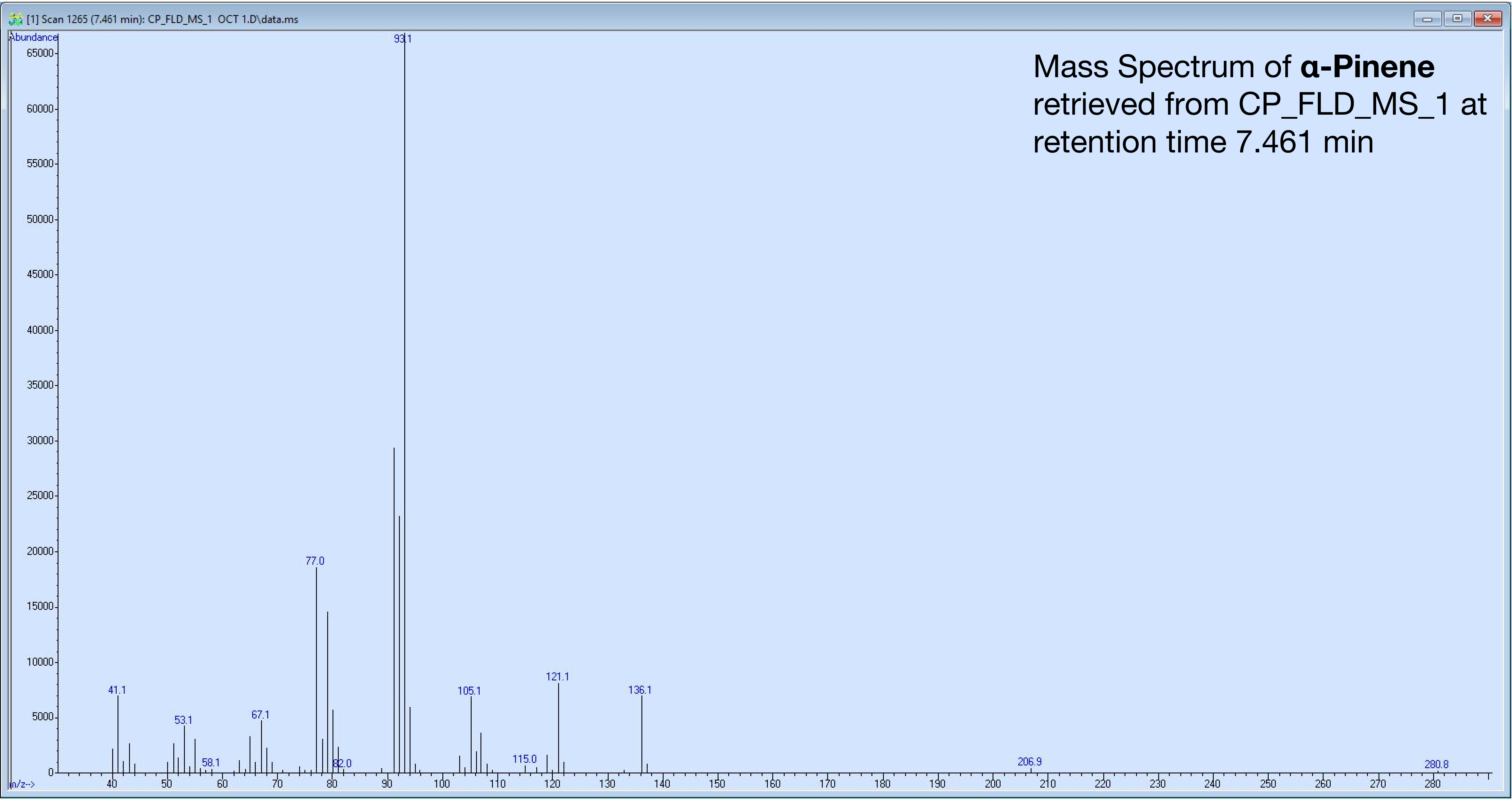

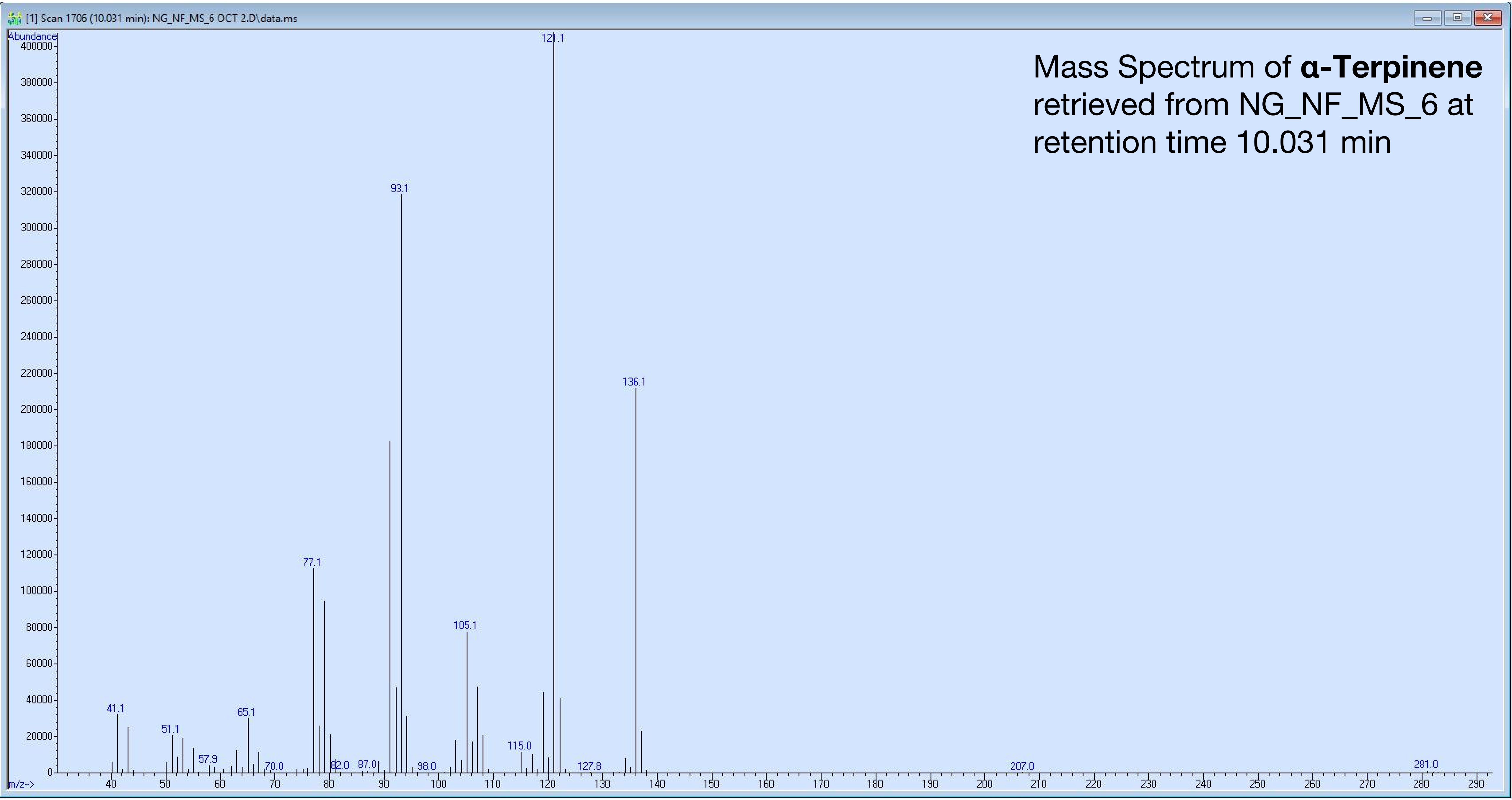
[1] Scan 1706 (10.031 min): NG_NF_MS_6 OCT 2.D\data.ms
Mass Spectrum of **α-Terpinene** retrieved from NG_NF_MS_6 at retention time 10.031 min
Abundance
m/z-->
121.1
93.1
136.1
77.1
105.1
41.1
51.1
65.1
57.9
70.0
82.0
87.0
98.0
115.0
127.8
207.0
281.0

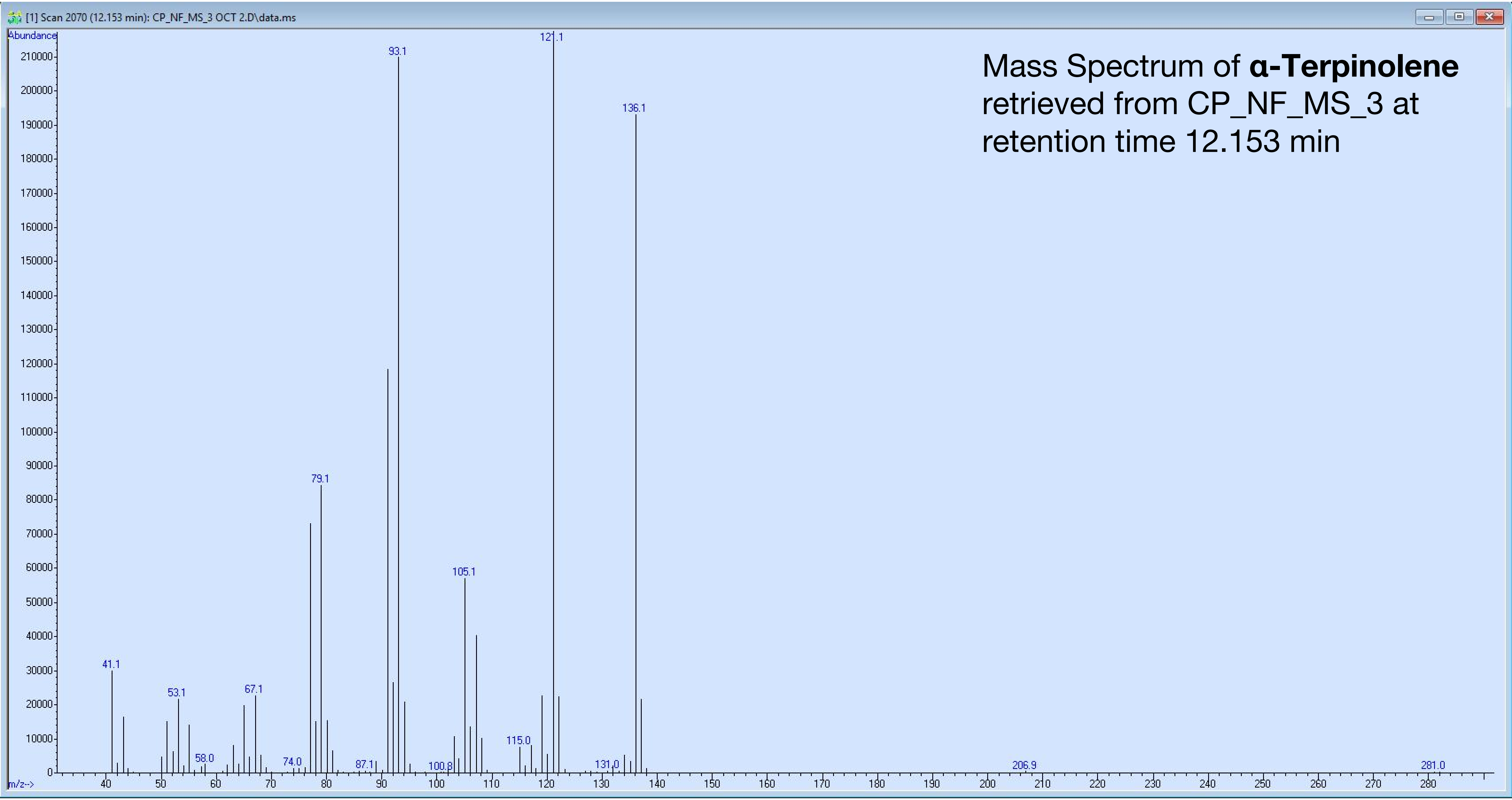

Mass Spectrum of **α-Terpinolene** retrieved from CP_NF_MS_3 at retention time 12.153 min

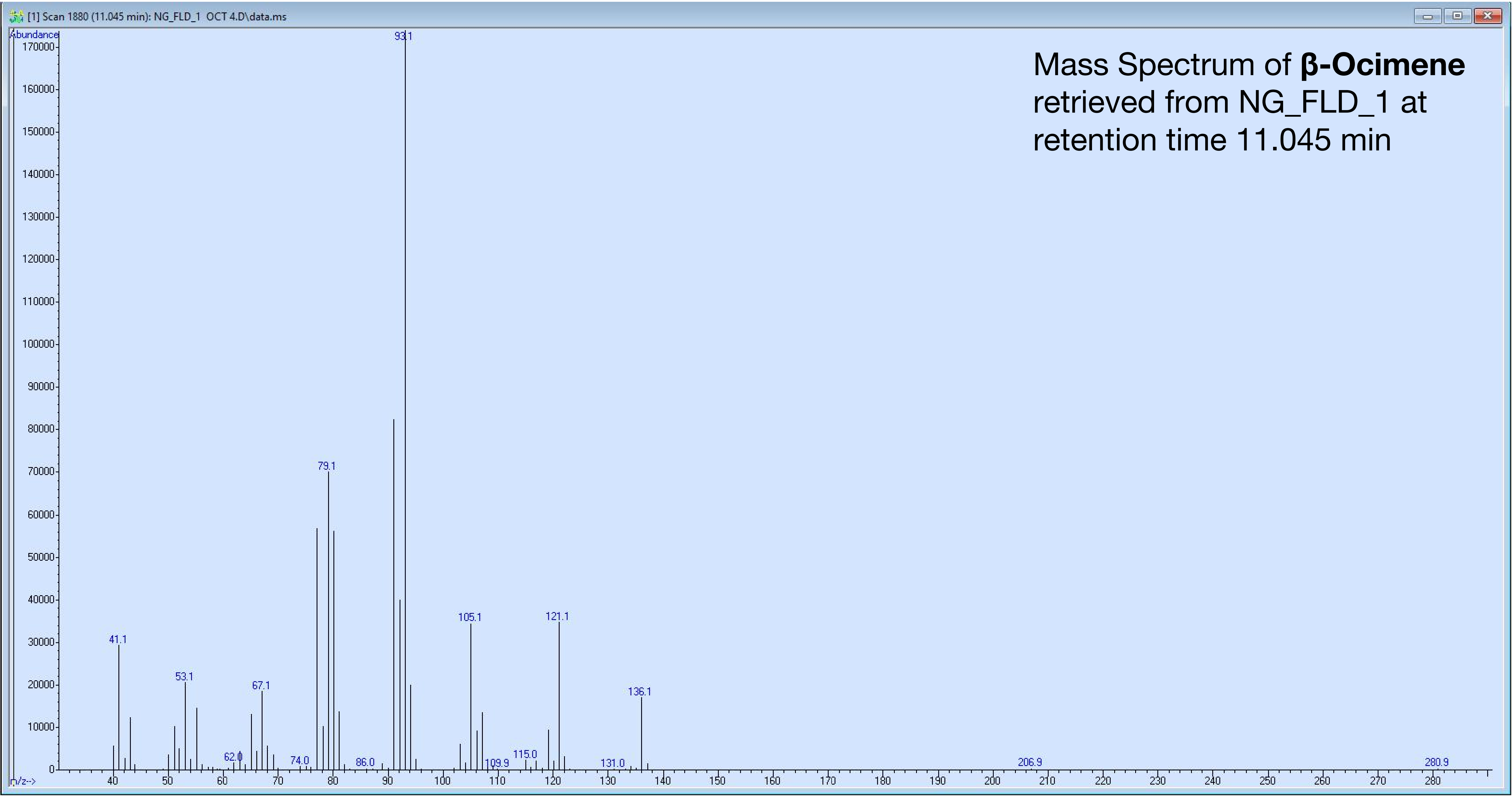

[1] Scan 1880 (11.045 min): NG_FLD_1 OCT 4.D\data.ms
Abundance
m/z-->
93.1
79.1
41.1
53.1
67.1
62.0
74.0
86.0
105.1
109.9
115.0
121.1
131.0
136.1
206.9
280.9
Mass Spectrum of **β-Ocimene** retrieved from NG_FLD_1 at retention time 11.045 min

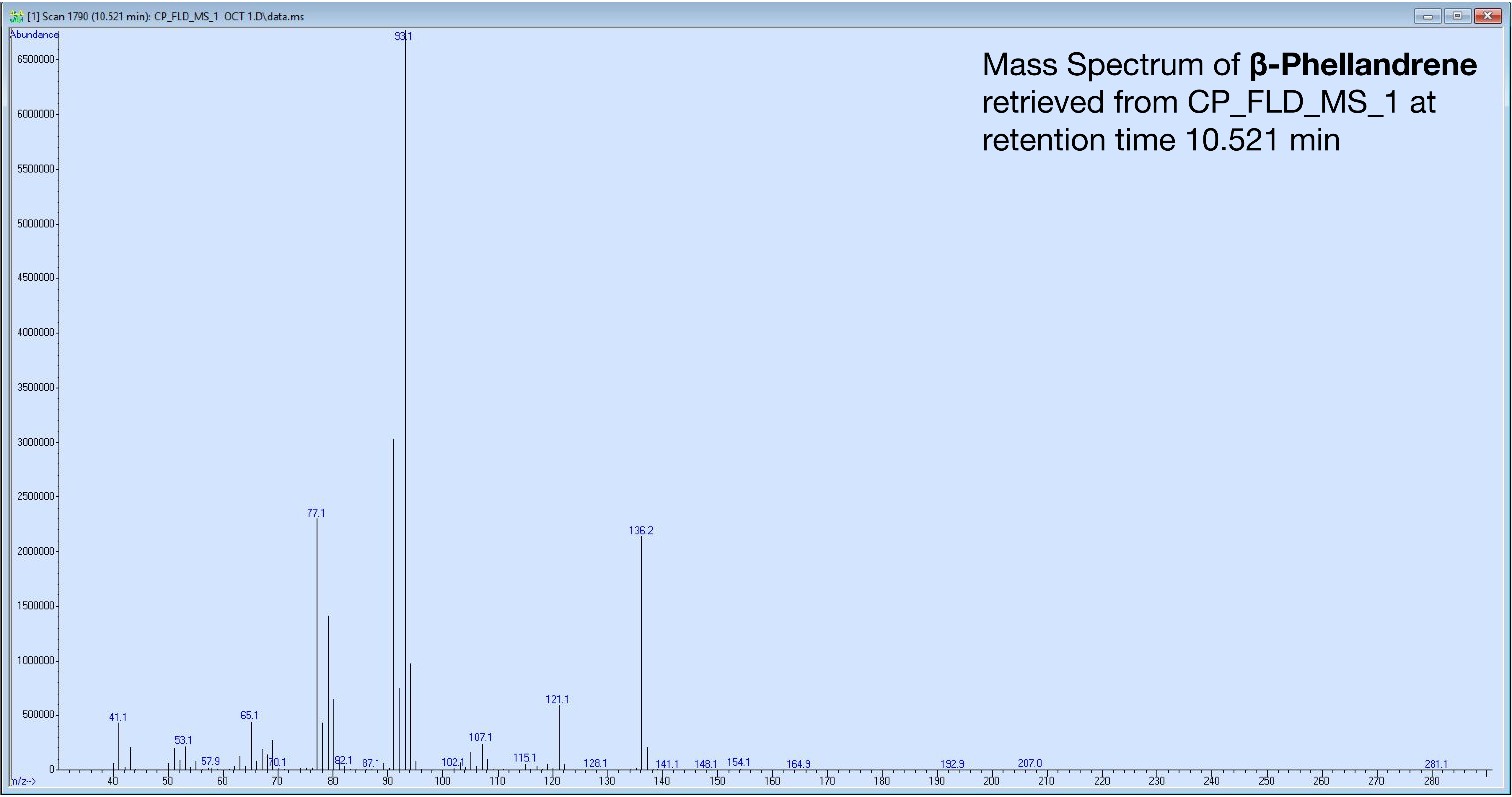
[1] Scan 1790 (10.521 min): CP_FLD_MS_1 OCT 1.D\data.ms
Abundance
m/z-->
93.1
77.1
136.2
121.1
107.1
65.1
41.1
53.1
57.9
70.1
82.1
87.1
102.1
115.1
128.1
141.1
148.1
154.1
164.9
192.9
207.0
281.1
Mass Spectrum of **β-Phellandrene**
retrieved from CP_FLD_MS_1 at
retention time 10.521 min

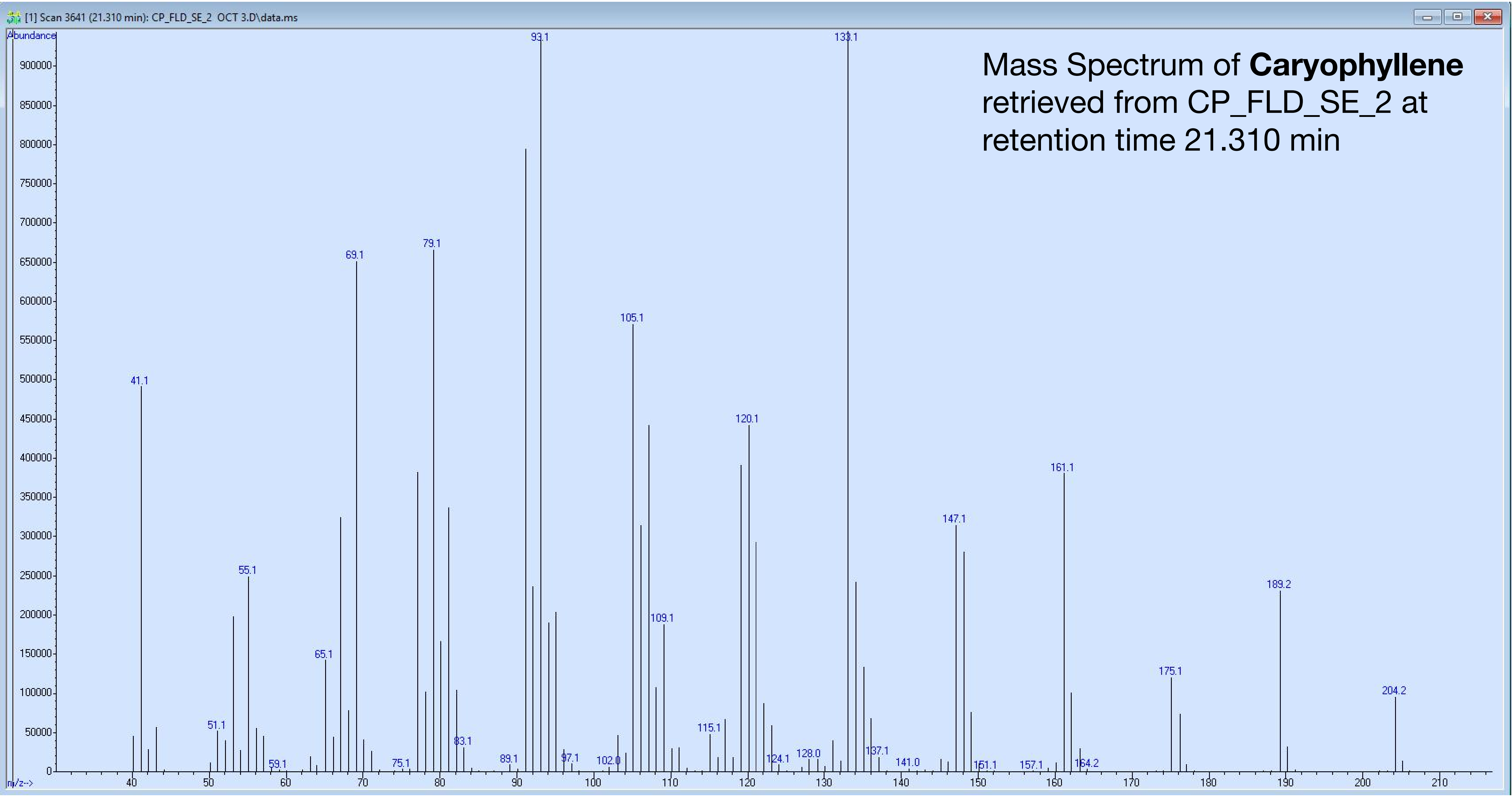
[1] Scan 3641 (21.310 min): CP_FLD_SE_2 OCT 3.D\data.ms
Abundance
m/z-->
Mass Spectrum of **Caryophyllene** retrieved from CP_FLD_SE_2 at retention time 21.310 min
41.1
51.1
55.1
59.1
65.1
69.1
75.1
79.1
83.1
89.1
93.1
97.1
102.0
105.1
109.1
115.1
120.1
124.1
128.0
133.1
137.1
141.0
147.1
151.1
157.1
161.1
164.2
175.1
189.2
204.2

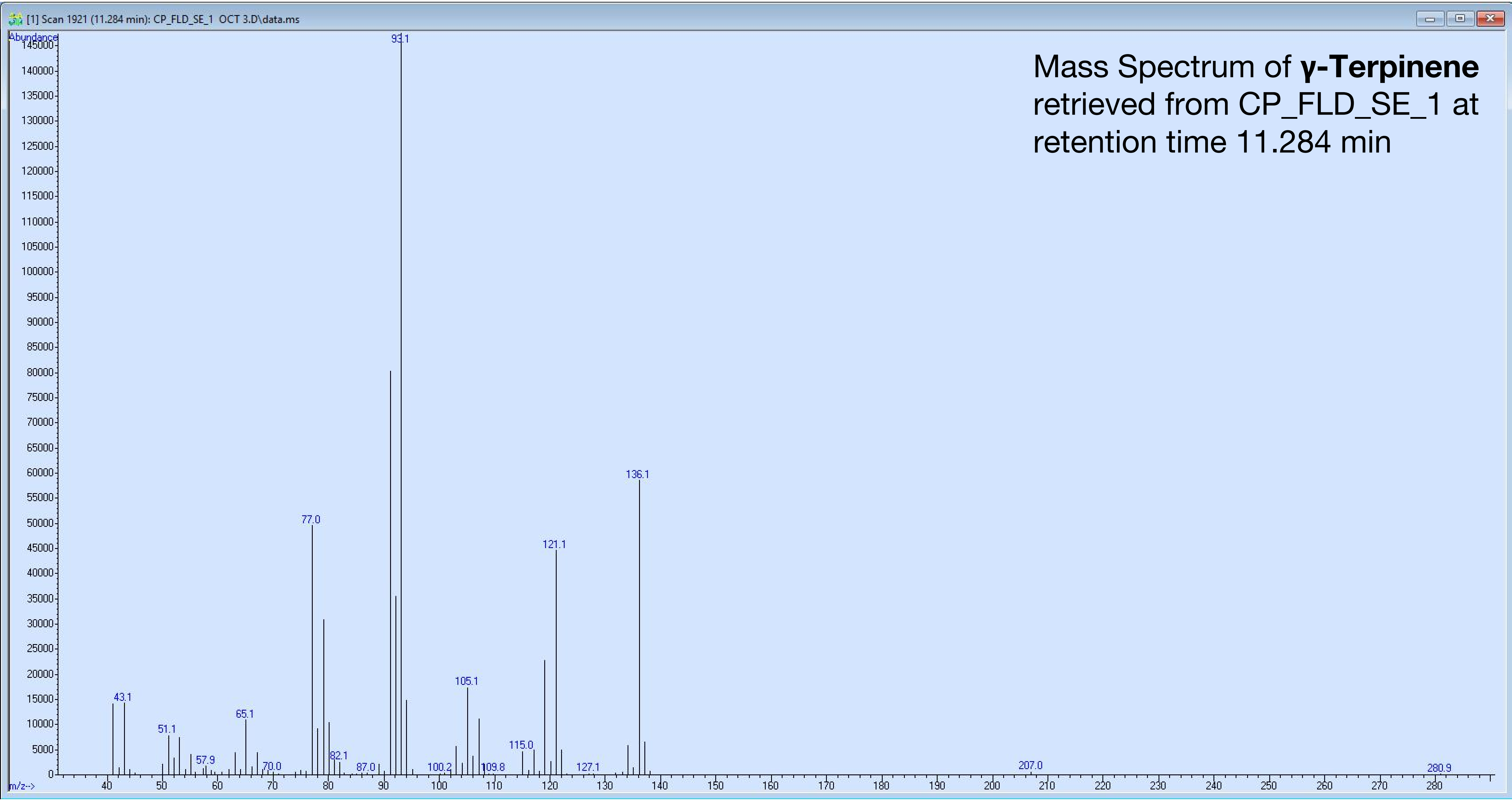
[1] Scan 1921 (11.284 min): CP_FLD_SE_1 OCT 3.D\data.ms
Abundance
m/z-->
93.1
136.1
77.0
121.1
105.1
43.1
65.1
51.1
115.0
57.9
82.1
70.0
87.0
100.2
109.8
127.1
207.0
280.9
Mass Spectrum of **γ-Terpinene** retrieved from CP_FLD_SE_1 at retention time 11.284 min

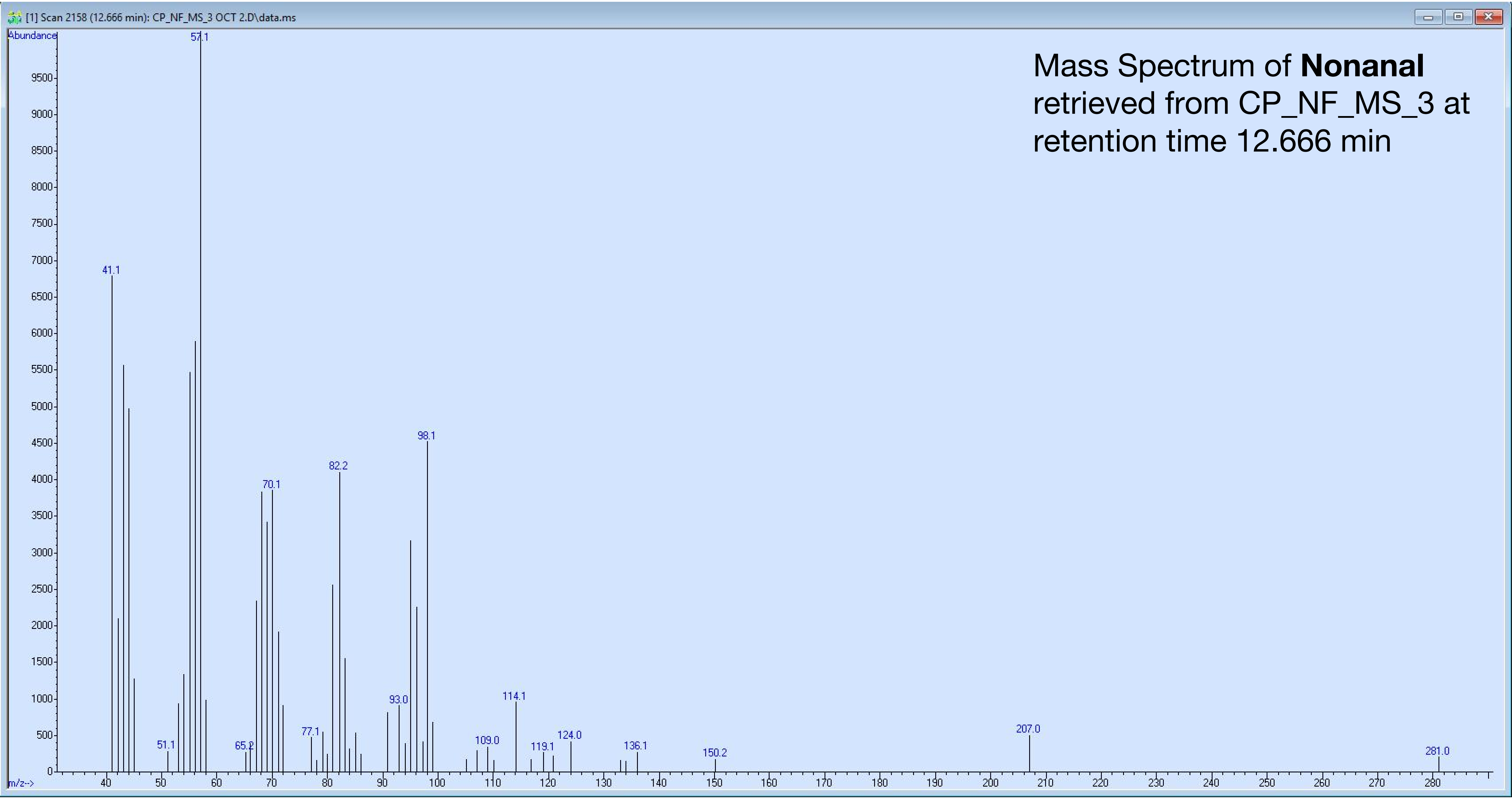
[1] Scan 2158 (12.666 min): CP_NF_MS_3 OCT 2.D\data.ms
Abundance
m/z-->
57.1
41.1
98.1
82.2
70.1
114.1
93.0
207.0
77.1
109.0
124.0
51.1
65.2
119.1
136.1
150.2
281.0
Mass Spectrum of **Nonanal** retrieved from CP_NF_MS_3 at retention time 12.666 min

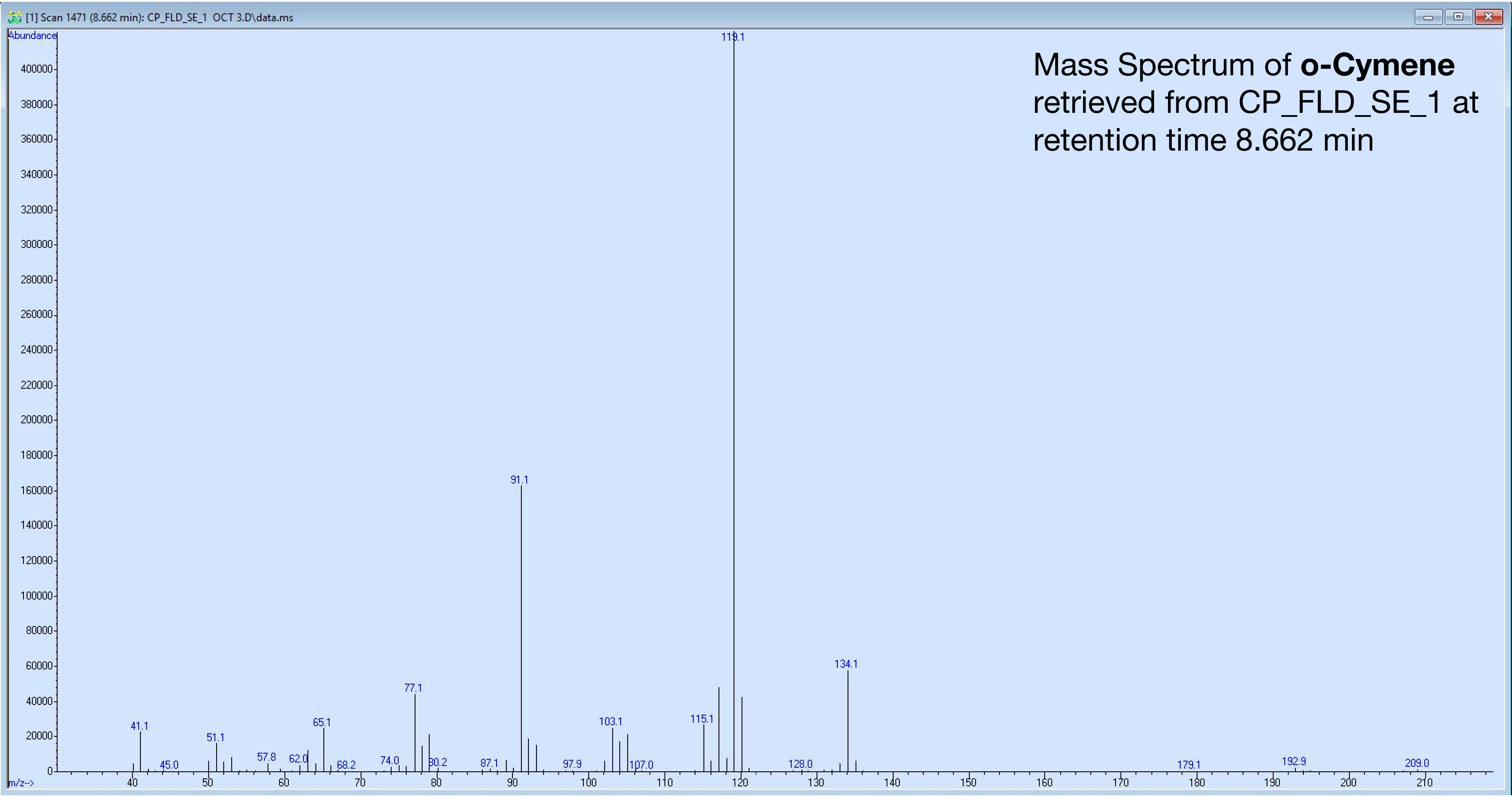
[1] Scan 1471 (8.662 min): CP_FLD_SE_1 OCT 3.D\data.ms
Abundance
m/z-->
119.1
91.1
134.1
77.1
41.1
51.1
65.1
103.1
115.1
45.0
57.8
62.0
68.2
74.0
80.2
87.1
97.9
107.0
128.0
179.1
192.9
209.0
Mass Spectrum of **o-Cymene** retrieved from CP_FLD_SE_1 at retention time 8.662 min
