# Supplementary material for: Metabolic and Biochemical Responses of Heirloom and Hybrid Tomato (Solanum lycopersicum) Under Flooding, Specialist, and Generalist Insect Herbivory, and their Stress Combination
Source: J Chem Ecol. 2026 Apr 2;52(2):32. doi: 10.1007/s10886-026-01703-9 (PMC13046590; doi:10.1007/s10886-026-01703-9)

Supplementary Figure 3

-Pictures of plants in different treatments

-Experiment scheme

Pictures of Plants in Different Treatments

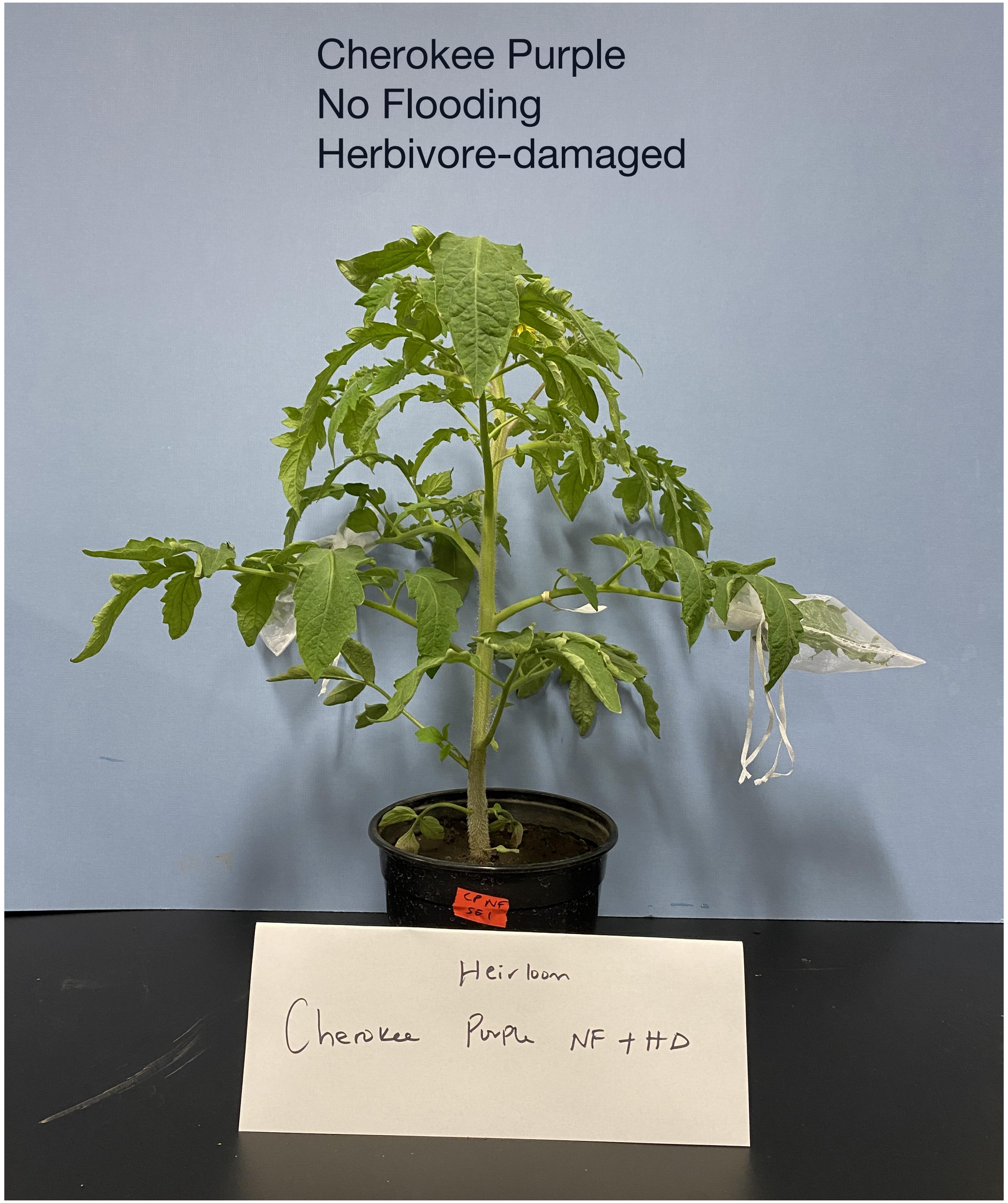
Cherokee Purple
No Flooding
Herbivore-damaged
Heirloom
Cherokee Purple NF + HD

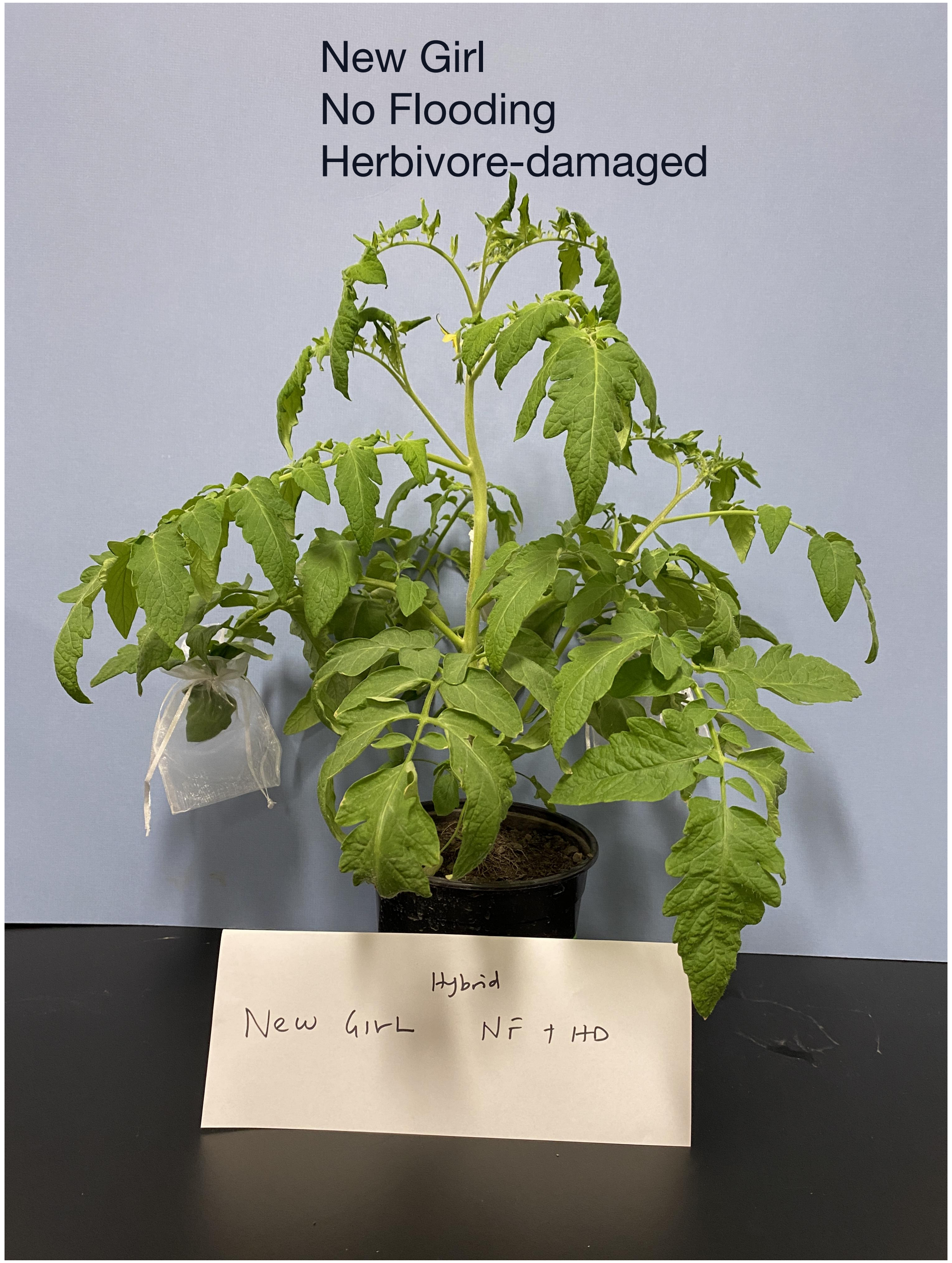
New Girl
No Flooding
Herbivore-damaged
Hybrid
New Girl NF + HD

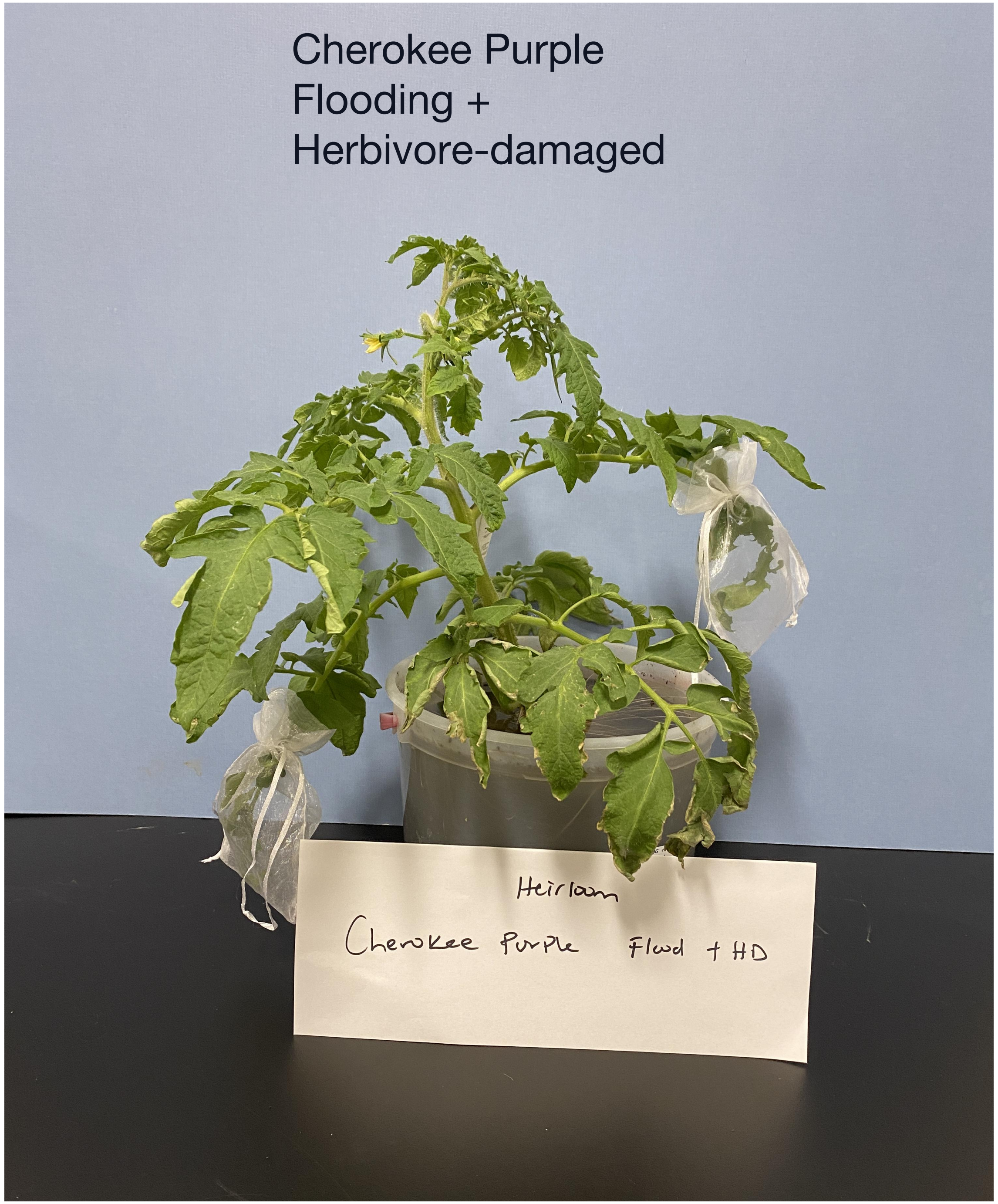
Cherokee Purple
Flooding +
Herbivore-damaged
Heirloom
Cherokee Purple Flood + HD

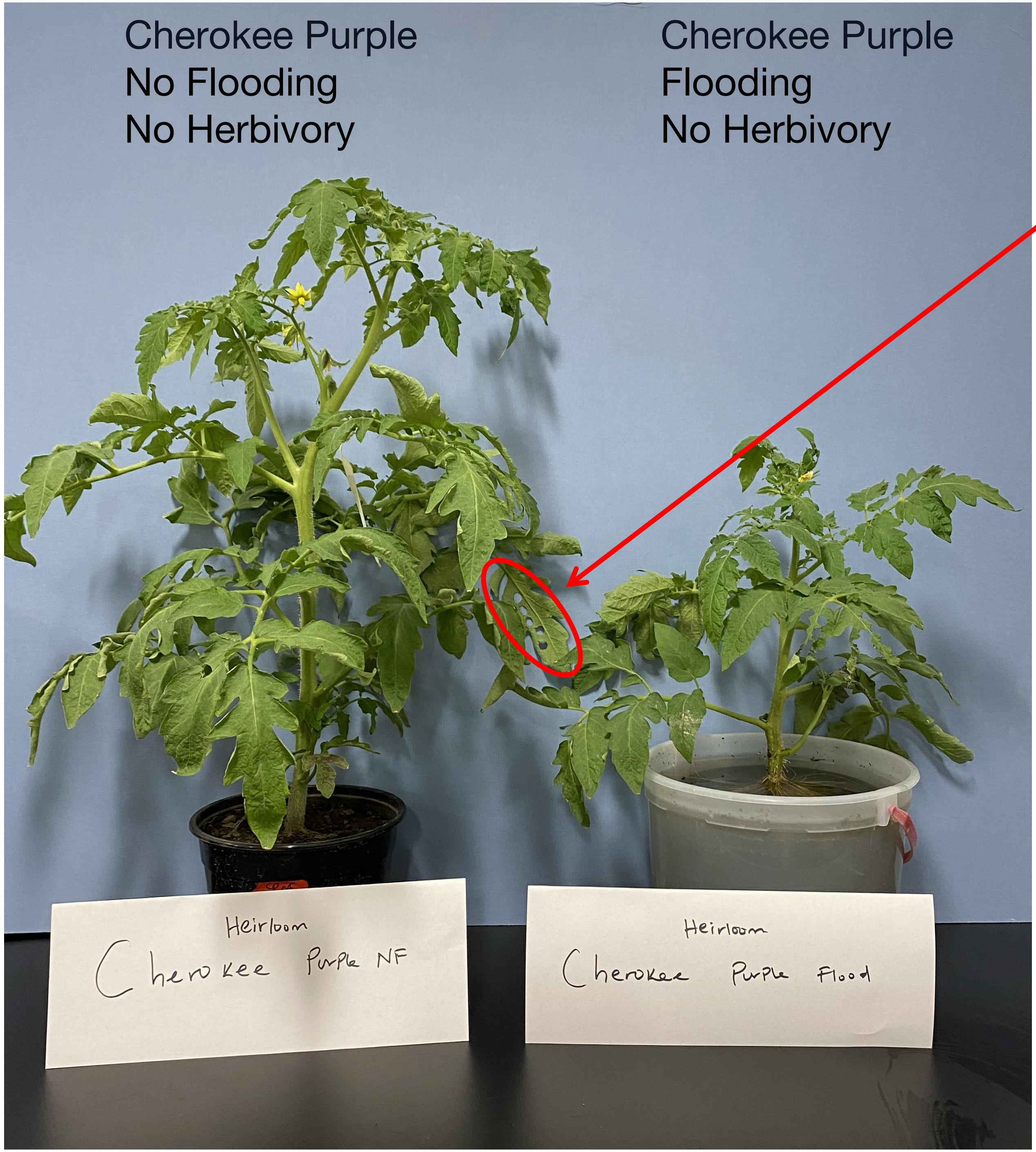

Leaves that had been collected tissue for metabolomic analysis

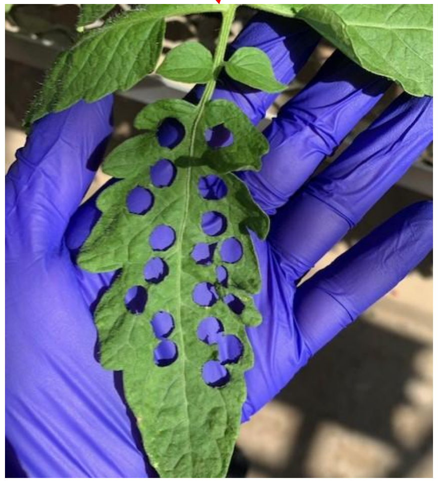

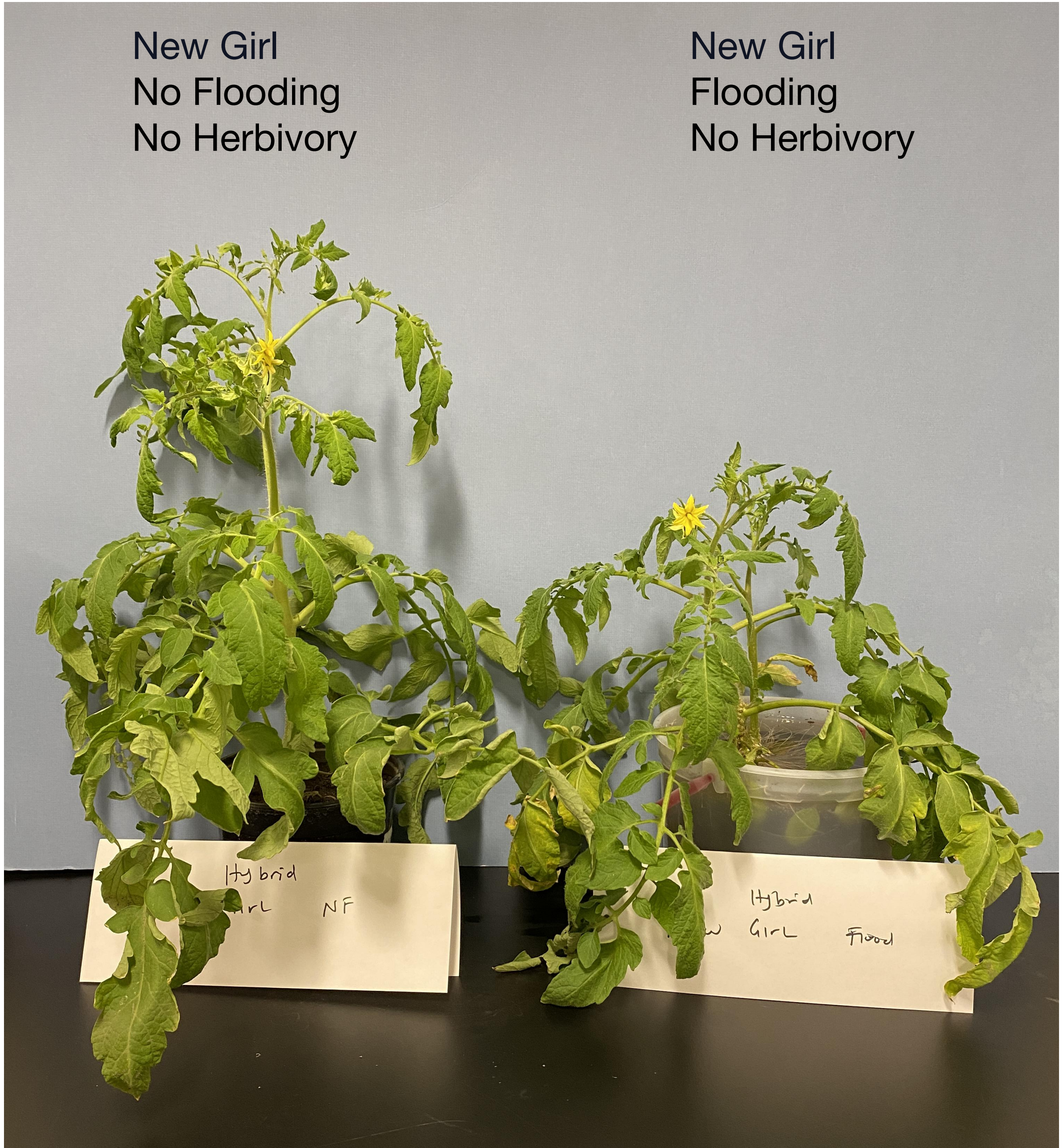
New Girl
No Flooding
No Herbivory
New Girl
Flooding
No Herbivory
Hybrid
NF
Hybrid
GirL
Flood

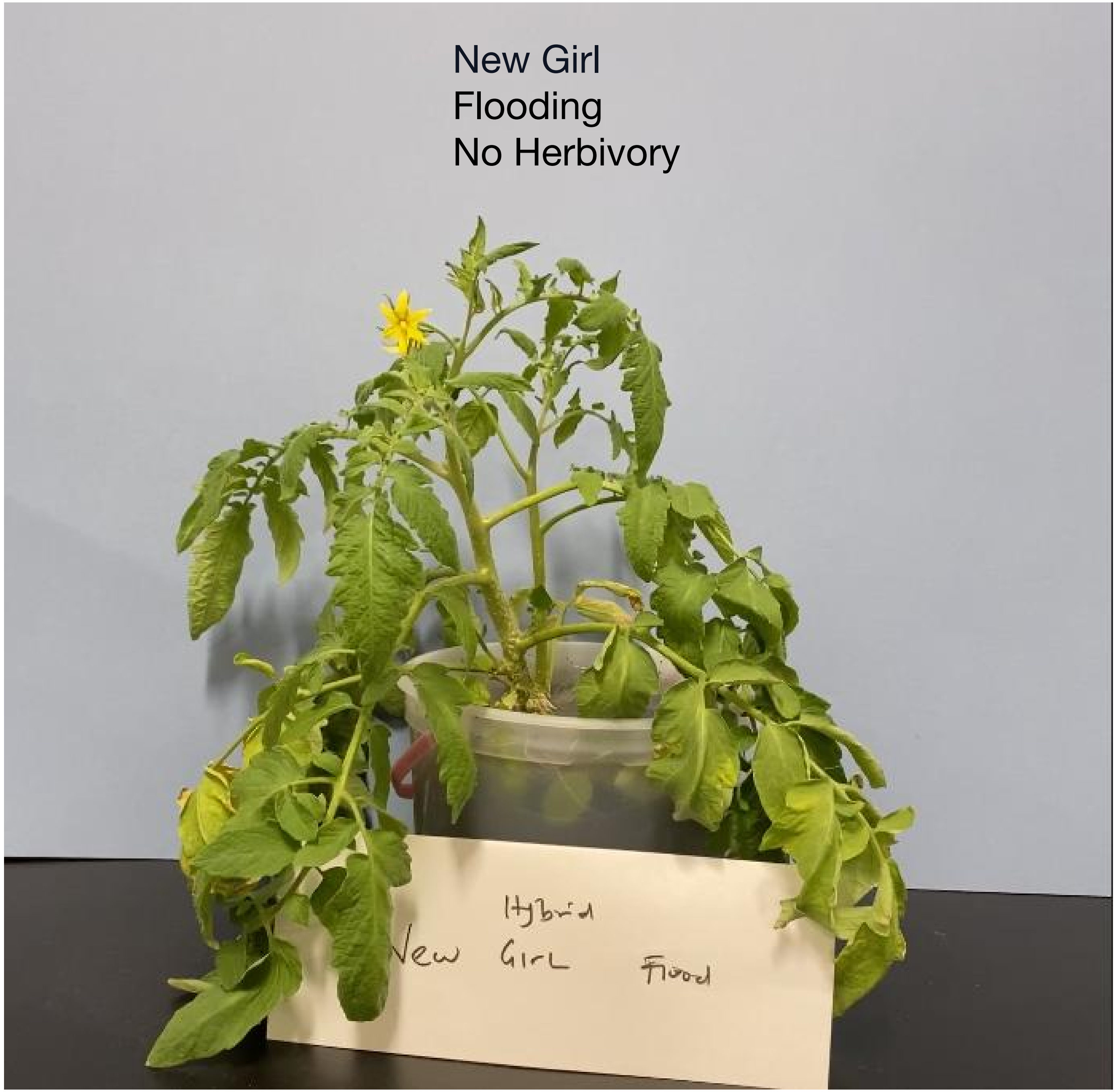
New Girl
Flooding
No Herbivory
Hybrid
New Girl Flood

Plant arrangement in the greenhouse

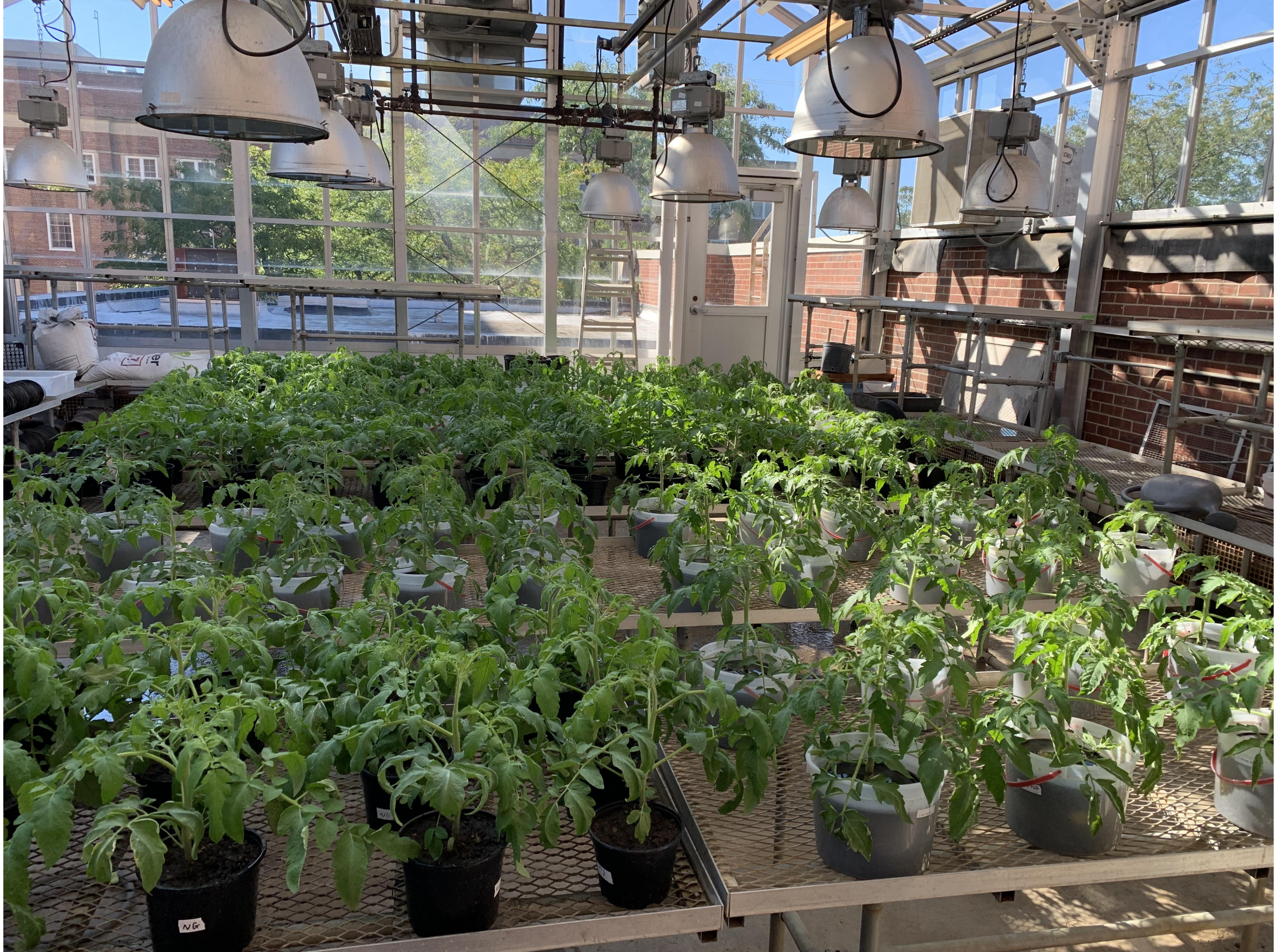

Overall Scheme of the Experiment

The 6 treatments: 1) Flooding; 2) Flooding+*Spodoptera exigua*-damged; 3) Flooding+*Manduca setxa*-damaged; 4) No flooding; 5) No flooing+*Spodoptera exigua*-damged; 6) No flooing+*Manduca setxa*-damged

-Same 6 treatments are applied to both heirloom (Cherokee Purple) and hybrid (New Girl) tomato variety

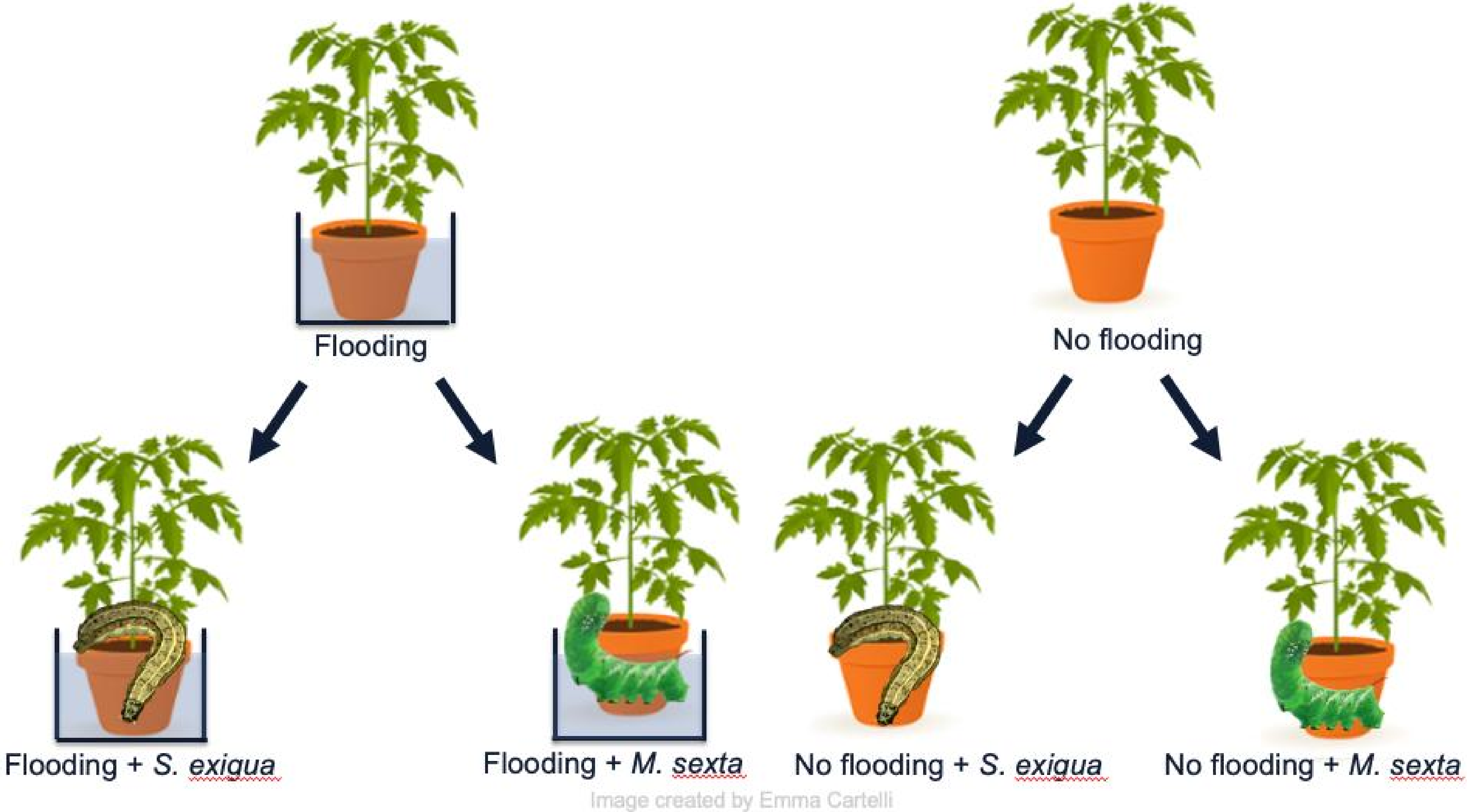

Supplement: Supplementary file 3 — Supplementary Fig. 3. Pictures of plants under different treatments and the overall experimental scheme. [file 10886_2026_1703_MOESM3_ESM.pdf]
