# Supplementary figures and images for: Metabolic and Biochemical Responses of Heirloom and Hybrid Tomato (Solanum lycopersicum) Under Flooding, Specialist, and Generalist Insect Herbivory, and their Stress Combination
Source: J Chem Ecol. 2026 Apr 2;52(2):32. doi: 10.1007/s10886-026-01703-9 (PMC13046590; doi:10.1007/s10886-026-01703-9)

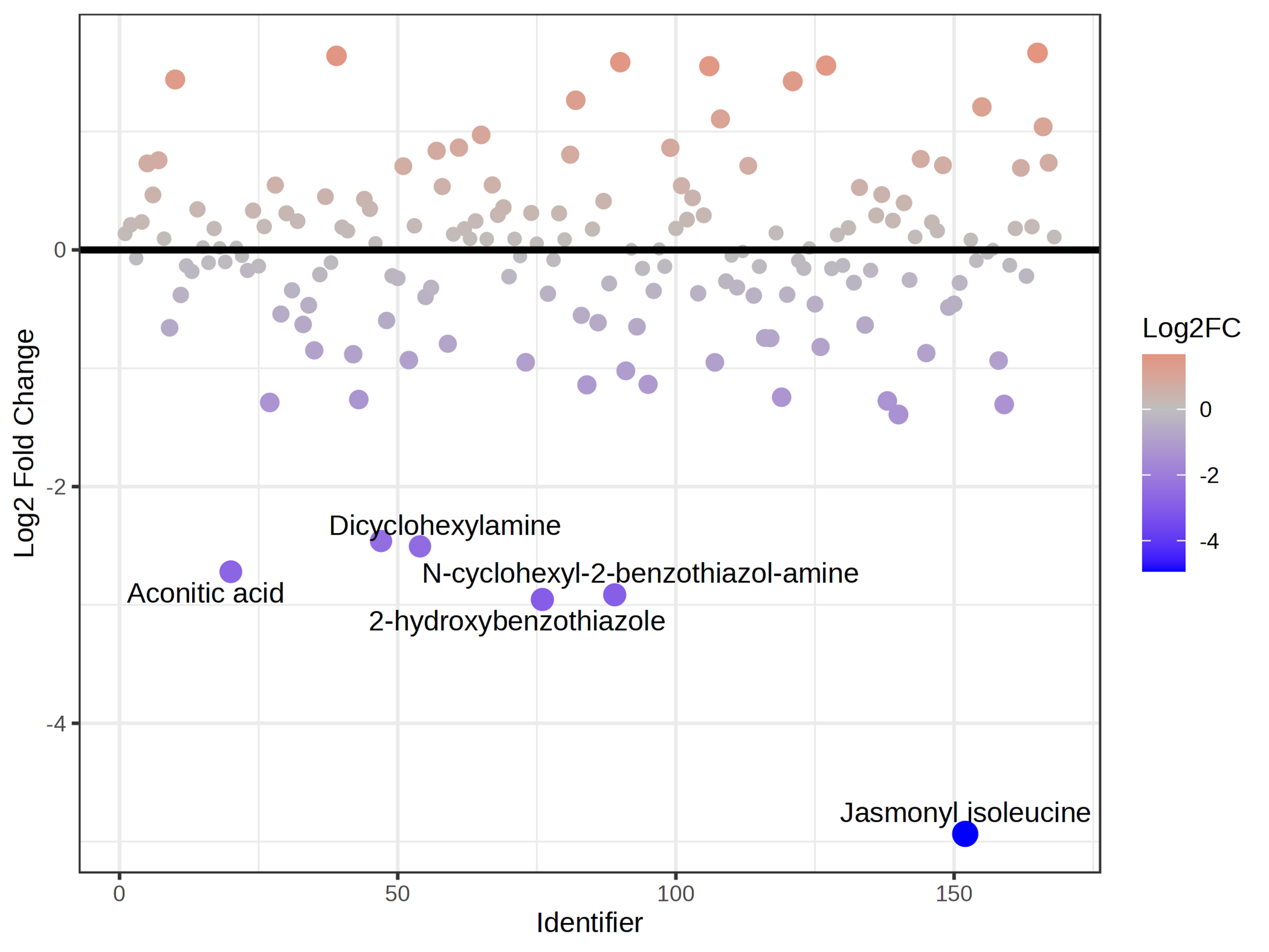

Supplement: Supplementary file 4 — Supplementary figure 4 (PNG 2.08 MB) [file 10886_2026_1703_Fig4_ESM.png]

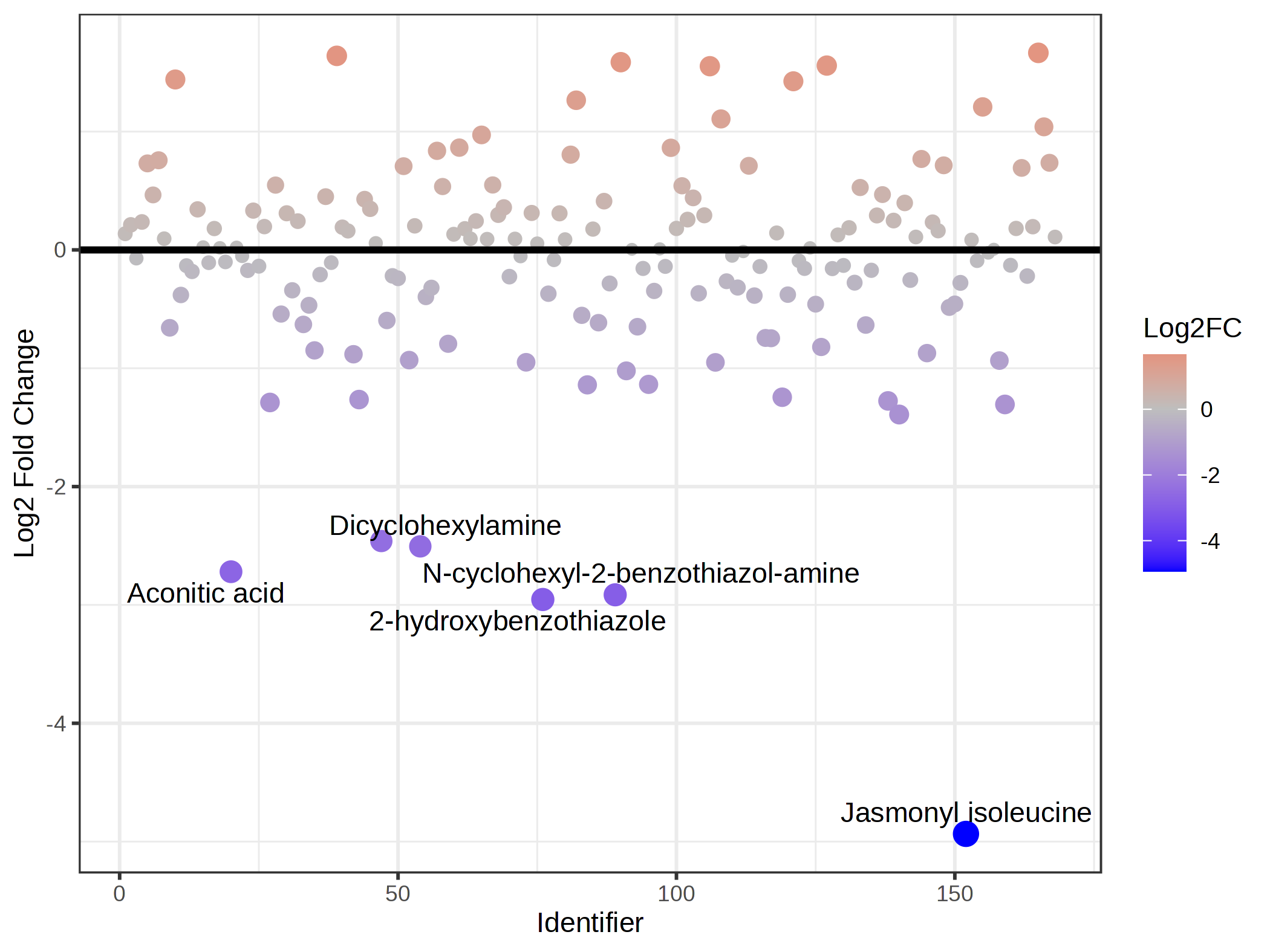

Supplement: Supplementary file 5 — Number of up-and down-accumulated metabolites in Cherokee Purple in response to feeding by the specialist caterpillar, Manduca sexta High Resolution Image (TIF 244 KB) [file 10886_2026_1703_MOESM4_ESM.tiff]

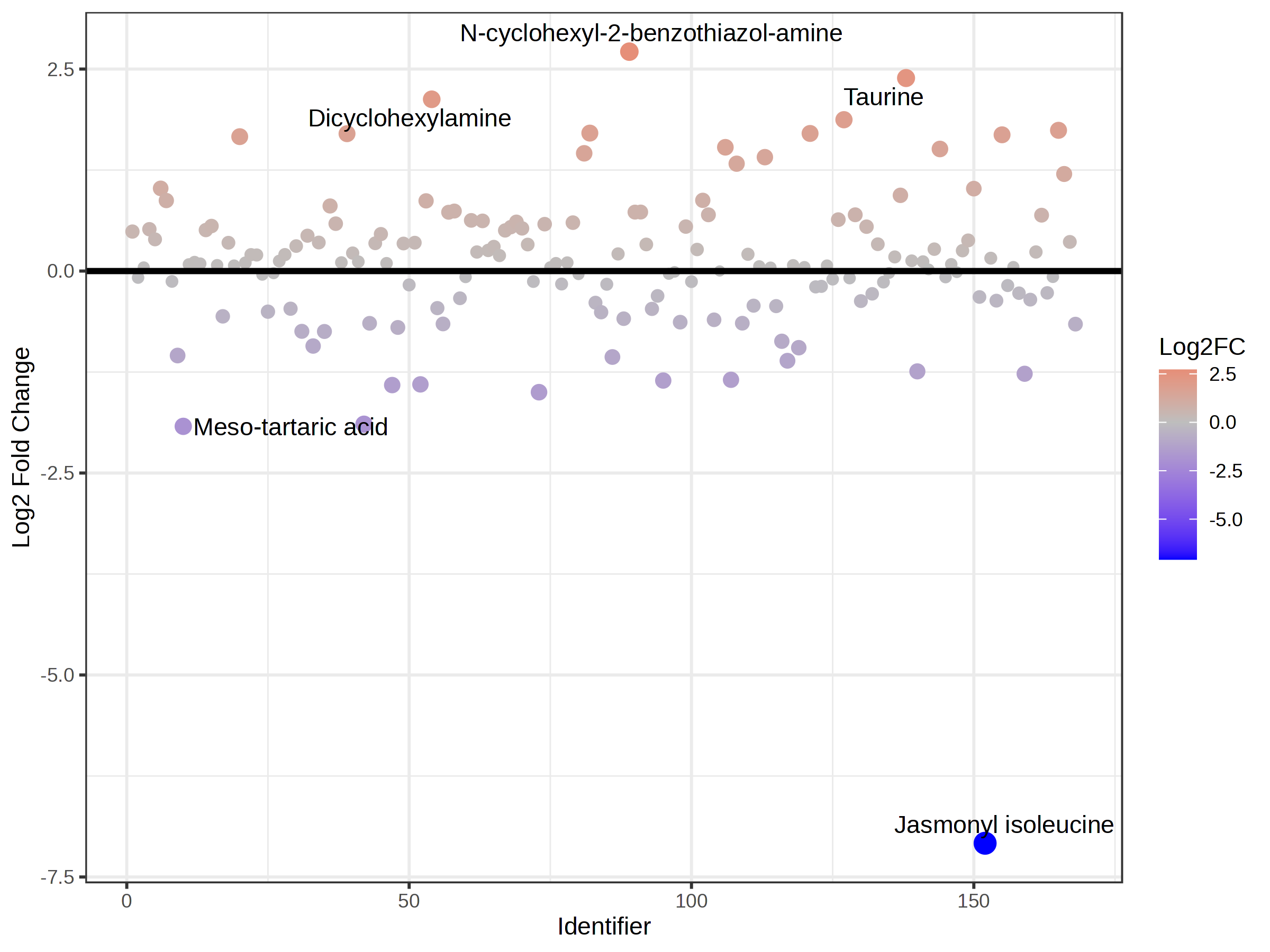

Supplement: Supplementary file 6 — Supplementary Fig. 5. Number of up-and down-accumulated metabolites in Cherokee Purple in response to feeding by the generalist caterpillar, Spodoptera exigua [file 10886_2026_1703_MOESM5_ESM.png]

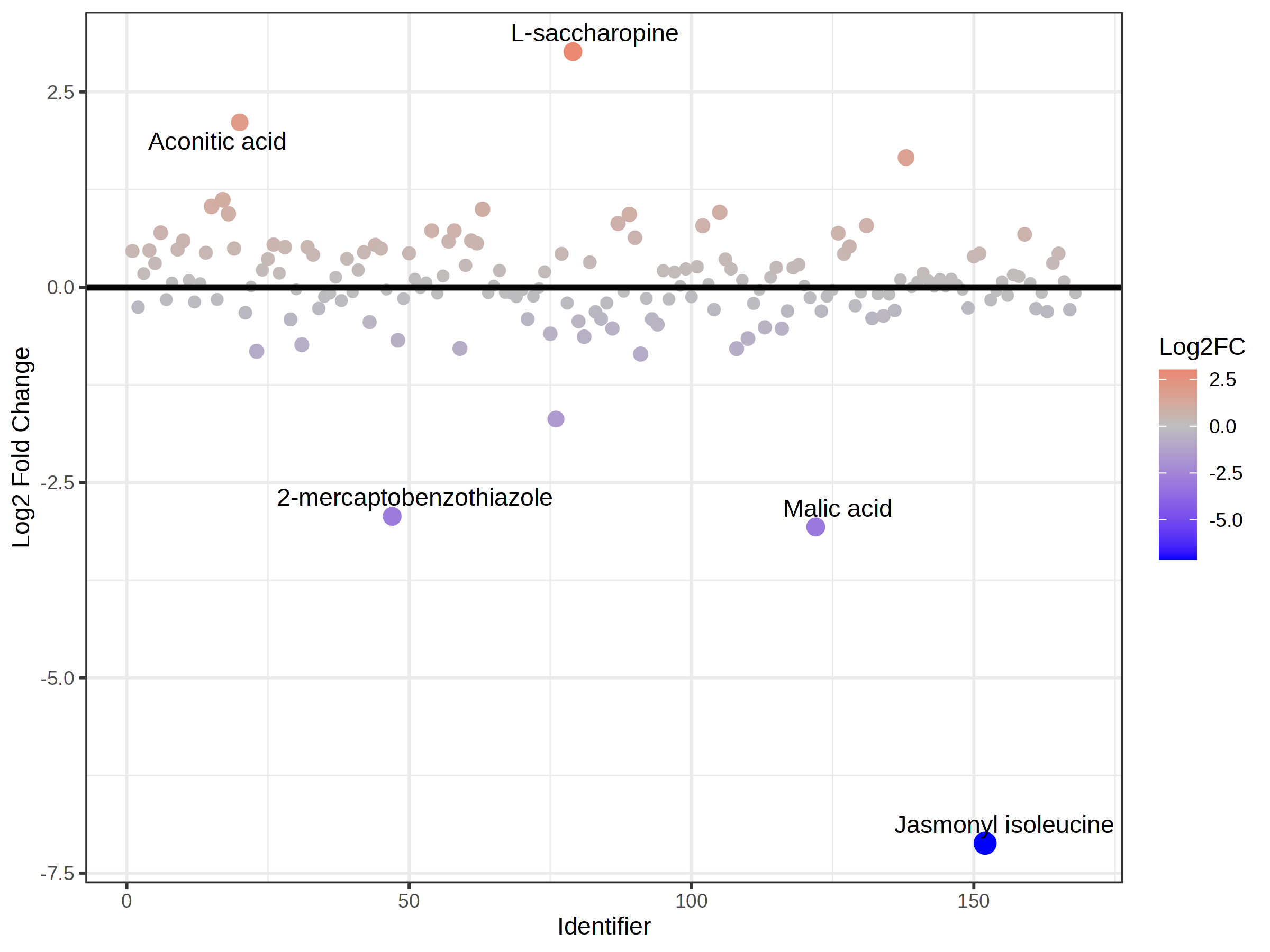

Supplement: Supplementary file 7 — Supplementary Fig. 6. Number of up-and down-accumulated metabolites in New Girl in response to feeding by the specialist caterpillar, Manduca sexta [file 10886_2026_1703_MOESM6_ESM.png]

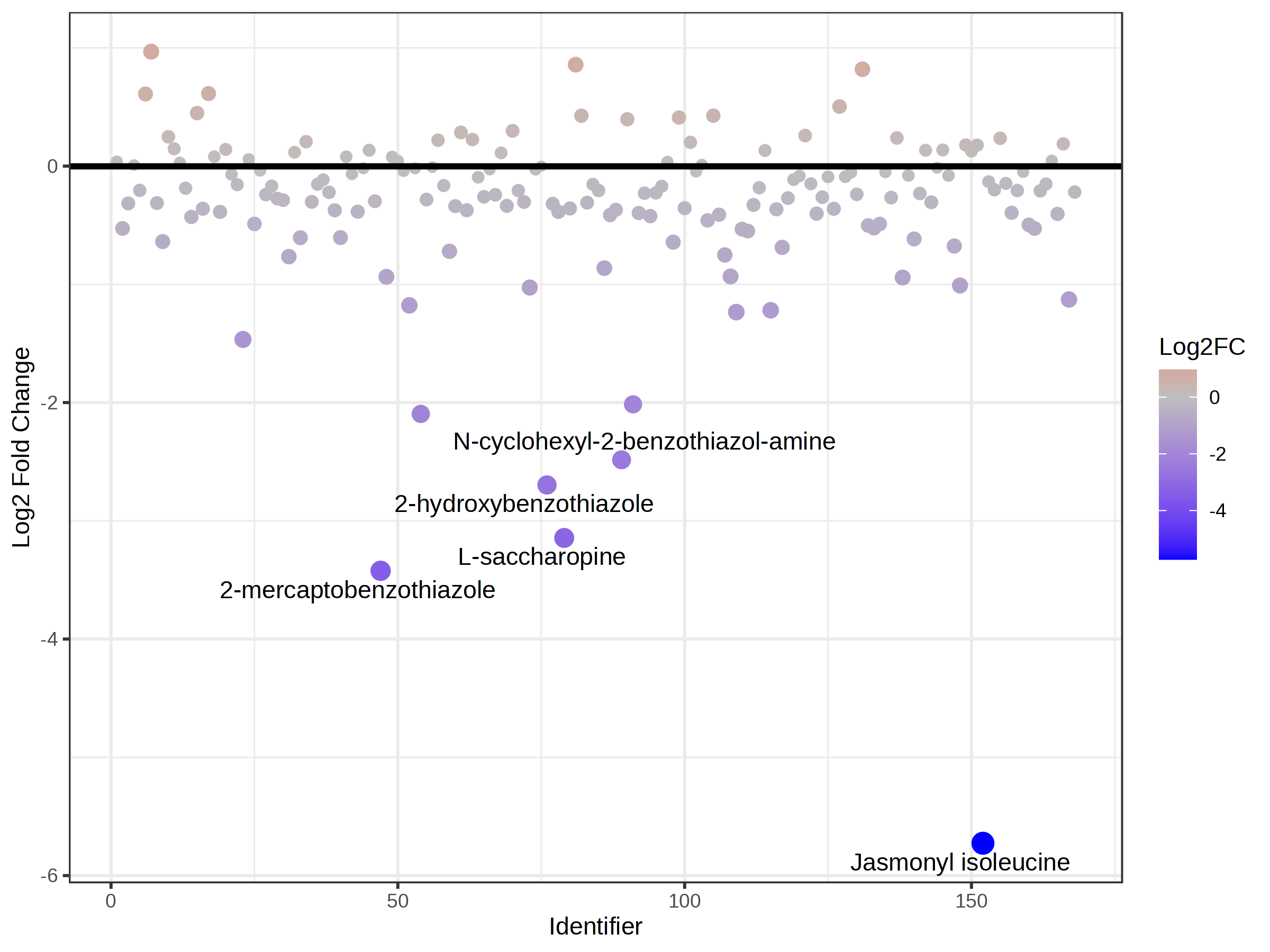

Supplement: Supplementary file 8 — Supplementary Fig. 7. Number of up-and down-accumulated metabolites in New Girl in response to feeding by the generalist caterpillar, Spodoptera exigua [file 10886_2026_1703_MOESM7_ESM.png]
